# Supplementary figures and images for: Cluster randomised trial of a health system strengthening approach applying person-centred communication for the prevention of female genital mutilation in Guinea, Kenya and Somalia
Source: BMJ Open. 2024 Jul 4;14(7):e078771. doi: 10.1136/bmjopen-2023-078771 (PMC11227771; doi:10.1136/bmjopen-2023-078771)

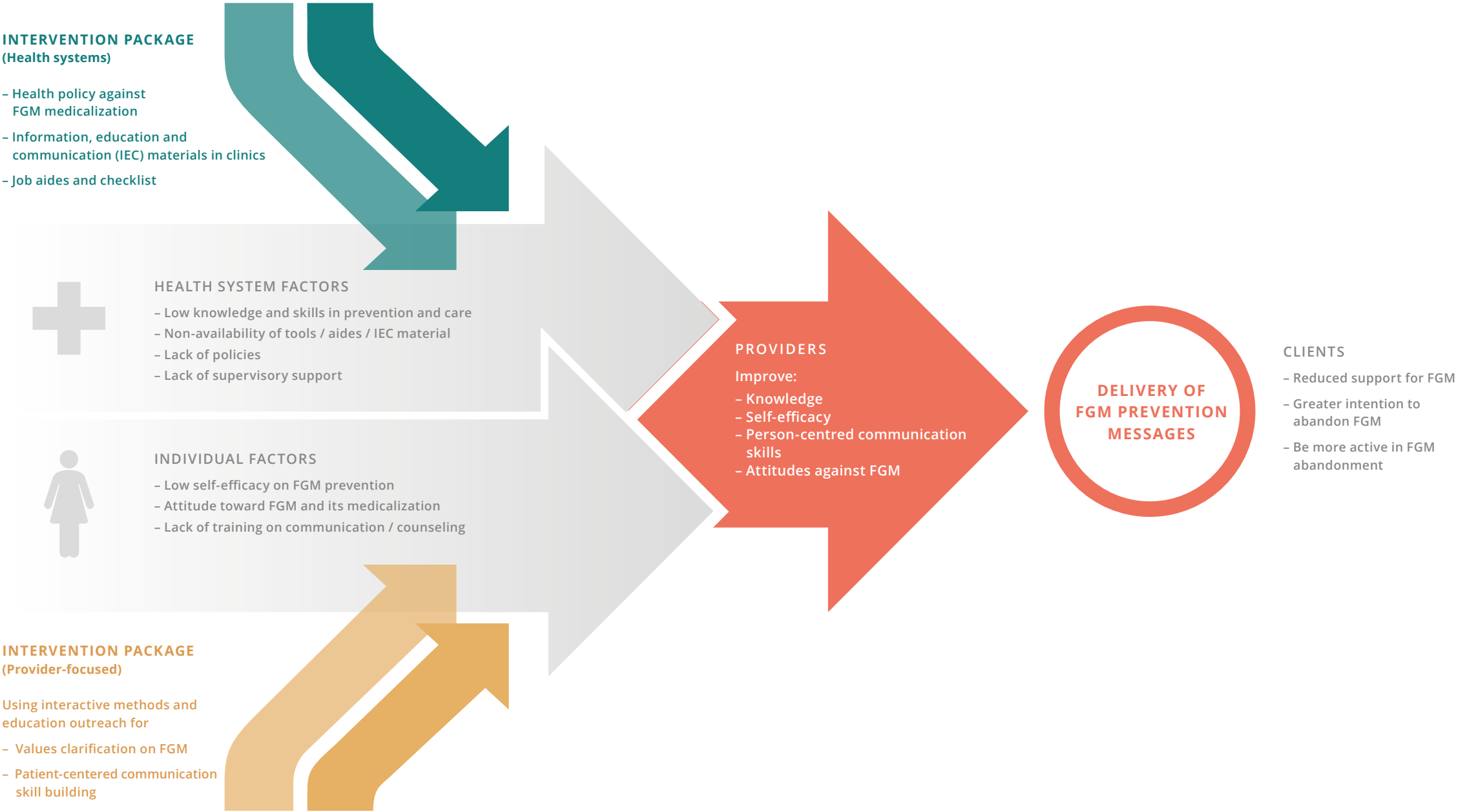

Supplement: Supplementary data [file bmjopen-2023-078771supp001.pdf]
