# Supplementary material for: Cluster randomised trial of a health system strengthening approach applying person-centred communication for the prevention of female genital mutilation in Guinea, Kenya and Somalia
Source: BMJ Open. 2024 Jul 4;14(7):e078771. doi: 10.1136/bmjopen-2023-078771 (PMC11227771; doi:10.1136/bmjopen-2023-078771)
Supplement: Supplementary data [file bmjopen-2023-078771supp002.pdf]

Supplementary file 2 : Measurement of study outcomes

1. ***Primary Outcome:*** *Health facility preparedness to provide FGM prevention and care services.*

Outcome definition*: Cumulative score based on affirmative responses to Q9a, Q10a, Q11a & Q12a on the CHK form (see below).*

Q9. Is there an MoH policy on FGM posted on the wall?
Yes
No
Q9a. If yes, is it placed where health care providers can see/read it e.g., bulletin board?
Yes
No
Q10. Are there WHO FGM prevention posters on the wall of the consultation room and/or waiting room?
Yes
No
Q10a. If yes, are they placed in a place where ANC clients can see them?
Yes
No
Q11. Is there a WHO clinical handbook in the ANC consultation room?
Yes
No
Q11a If yes, is it placed where ANC providers can see/use it?
Yes
No
Q12. Is there an FGM ABCD guide in the ANC consultation room?
Yes
No
Q12a. If yes, is it placed where ANC providers can see/use it?
Yes
No

2. ***Primary outcome: ANC provider utilization of Level 1 package components***

Outcome definition*: Affirmative response on Q40 of HCP form (see below).*

Q40. Have you referred to the WHO Clinical Handbook on FGM?
Yes
No, available but not referred
No, not available
Don't know

3. ***Primary outcome: Provision of FGM-related care after PCC training***

Outcome definition: *Cumulative score based on affirmative responses (Provision of FGM-related care (after PCC training) either 'Always' or 'Often') on Q22, Q24 & Q25 on the HCP form (see below).*

Q22. How often do you discourage a pregnant woman expecting to have a girl, or one having a girl at the age of cutting, from having her daughter cut?
Always
Often
Sometimes
Rarely

Never
Rarely
Refused to answer

Q24. How often do you look for female genital mutilation when performing a gynecological examination of the vulva?

Always
Often
Sometimes
Rarely
Never
Rarely
Refused to answer

Q25. How often do you record female genital mutilation in the woman's medical file if you are aware that she has undergone FGM?

Always
Often
Sometimes
Rarely
Never
Rarely
Refused to answer

4. Primary Outcome: Delivery of PCC 'ABCD' package

Outcome definition: *Cumulative score based on affirmative responses on Q5, Q7, Q8, Q9 & Q12 on the EXT form.*

Q5. Did the ANC provider ask if you have undergone FGM?

Yes
No
Don't know
Refused

Q7. Did the ANC provider ask about your personal belief regarding FGM?

Yes
No
Don't know
Refused

Q8. Did the ANC provider discuss why FGM should be prevented?

Yes
No
Don't know
Refused

Q9. Did the ANC provider discuss how FGM could be prevented?

Yes
No
Don't know
Refused

Q12. Are you satisfied with how FGM was addressed during your visit with your ANC provider today?

Yes
No
Don't know
Refused

5. ***Secondary Outcome: Improved knowledge about FGM***

Outcome definition: *Cumulative score based on correct responses to Q4 + affirmative responses to Q5 & Q7 of the HCP form.*

Q4. Please provide the WHO classification for the following images
Type I
Type II
Type III
Type IV
Don't Know
Other

Q5. Do you know of any health complications arising from female genital mutilation?
Yes
No

Q7. Are you aware of any existing WHO tools/guidance on FGM prevention and care?
Yes
No

6. ***Secondary Outcome: Improved interpersonal communication skills***

Outcome definition: *Cumulative score based on positive responses ("Always or Often') to Q34, Q35, Q36, Q37, Q38 on the HCP form.*

Now I will ask you about your communication skills

34. I can put myself in others shoes
Always
Often
Sometimes
Rarely
Never
Rarely
Refused to answer

35. I let others know that I understand what they say
Always
Often
Sometimes
Rarely
Never
Rarely
Refused to answer

36. In conversations with my colleagues, I perceive not only what they say but what they don't say
Always
Often
Sometimes
Rarely
Never
Rarely
Refused to answer

37. I communicate effectively
Always
Often

Sometimes
Rarely
Never
Rarely
Refused to answer

38. *I communicate with others as though they are my equals*
    Always
    Often
    Sometimes
    Rarely
    Never
    Rarely
    Refused to answer

7. Secondary outcome: Improved self-efficacy

Outcome definition: *Cumulative score based on positive responses (Agree or Strongly Agree) to Q26, Q27, Q28, Q29, Q30, Q31, Q32, Q33 on the HCP form.*

Now I would like to ask you a few questions about how you solve problems that you face. Please tell me how much you agree or disagree with the statements that I read to you

1 = Strongly disagree
2 = Disagree
3 = Neither agree nor disagree
4 = Agree
5 = Strongly agree

Q26. I will be able to achieve most of the goals that I have set for myself
Q27. When facing difficult tasks, I am certain that I will accomplish them
Q28. In general, I think that I can obtain outcomes that are important to me
Q29. I believe that I can succeed at almost any endeavor to which I set my mind
Q30. I will be able to successfully overcome many challenges
Q31. I am confident that I can perform effectively on many different tasks
Q32. Compared to other people, I can do most tasks very well
Q33. Even when things are tough, I can perform quite well

8. Secondary outcome: Improved attitudes towards FGM

Outcome definition*: Cumulative score based on positive responses to Q12, Q13, Q14, Q15, Q16, Q17, Q18 & Q19 on the HCP form.*

For each of the following statements please state if you:

1=Agree
2=Disagree
3=Don't know
4=Refused to answer

Q12. A girl who has not undergone FGM is unclean
Q13. A girl who has not undergone FGM cannot be married within her community
Q14. A girl who has not undergone FGM is a disgrace to her family's honor
Q15. Health care providers who provide FGM are violating FGM
Q16. Health care providers who provide FGM should be punished
Q17. FGM is a good practice
Q18. FGM is a violation of women and girls' rights
Q19. FGM is religious mandate

9. Tertiary outcome: ANC provider confidence in FGM knowledge to provide care

Outcome definition: *Positive responses ('Somewhat Confident' or 'Confident') to Q8 & Q9 on the HCP form*

Q8. When you treat or attend to a girl or woman with female genital mutilation, how confident are you that you have enough knowledge to provide good quality care?

1=Not confident
2=Somewhat confident
3=Confident
4=Refused to answer

Q9. How confident are you in your knowledge to communicate on FGM prevention?

1=Not confident
2=Somewhat confident
3=Confident
4=Refused to answer

10. Tertiary outcome: ANC provider support for FGM

Outcome definition: *Positive response ('Do not intend to cut her') to Q20 on the HCP form*

Q20. Pretend you had a daughter now who was at an age when cutting occurs, what would your intention to cut her be?

1=Intend to cut her
2=Do not intend to cut her
3=Don't know
4=Refused to answer

11. Tertiary outcome: ANC provider support for medicalized FGM

Outcome definition: *Correct response ('No') to Q21 on HCP form*

Q21. If a family brought their daughter to the clinic requesting genital cutting, for non-health reasons, would you perform it?

1=Yes
2=No
3=Don't know
4=Refused to answer

12. Tertiary outcome: ANC client change in support for FGM after ANC visit

Outcome definition: *Response to Q13 on EXT form*

Q13. What do you feel about FGM now as compared to before you came to the clinic today?

1= Same, no change
2=I feel more supportive of FGM now as compared to before I came
3=I feel less supportive of FGM now as compared to before I came
4=Don't know
5=Other
6=Refused to answer

13. Tertiary outcome: ANC client support or opposition to FGM

Outcome definition: *Response to Q14 on EXT form*

Q14. How supportive are you of female genital mutilation?

1=Strongly opposed
2=Somewhat opposed

3=Neutral
4=Somewhat supportive
5=Strongly supportive
6=Refused to answer

14. Tertiary outcome: ANC client intention to cut after ANC visit.

Outcome definition: *Response to Q16 on EXT form*

Q.16 Pretend you had a daughter now who was at an age where cutting occurs, what would your intention to cut her be?

1=Intend to cut her
2=Do not intend to cut her
3=Don't know
4=Refused to answer

15. Tertiary outcome: ANC client choice of who to cut their daughters.

Outcome definition: *Response to Q17 on EXT form*

Q17. If intending to cut, who would you prefer to do the cutting?

1=Traditional practitioner
2=Health care provider
3=Other
4=Refused to answer

16. Tertiary outcome: ANC client wish to be active in FGM prevention

Outcome definition: *Response to Q18 on EXT form*

Q.18 Do you wish/want to be active in preventing FGM?

1=Yes
2=No
3=Don't know
4=Refused to answer
