# Supplementary material for: Cluster randomised trial of a health system strengthening approach applying person-centred communication for the prevention of female genital mutilation in Guinea, Kenya and Somalia
Source: BMJ Open. 2024 Jul 4;14(7):e078771. doi: 10.1136/bmjopen-2023-078771 (PMC11227771; doi:10.1136/bmjopen-2023-078771)
Supplement: Supplementary data [file bmjopen-2023-078771supp003.pdf]

A HEALTH SYSTEMS APPROACH TO PREVENTION OF FEMALE GENITAL MUTILATION USING PERSON-CENTRED COMMUNICATION: IMPLEMENTATION RESEARCH PROJECT IN GUINEA, SOMALIA AND KENYA (A65993)

ANC PROVIDER SCREENING QUESTIONNAIRE (SCR)

Participant ID:

Project ID: Country ID: Facility ID:

A	6	5	9	9	3

Instructions: Observe and report findings from the health facility.

1. **MoH policy on FGM posted on the wall?**
   ☐ Yes
   ☐ No
   1a. If yes, is it placed where health care providers can see/read it e.g. bulletin board?
   ☐ Yes
   ☐ No
2. **Are there FGM prevention posters on the wall of the waiting room?** ☐ Yes
   ☐ No
   2a. If yes, is it placed in place where ANC clients can see it
   ☐ Yes
   ☐ No
3. **Is there WHO FGM Clinical Handbook in the ANC consultation room?** ☐ Yes
   ☐ No
   3a. If yes, is it placed where ANC provider can see /use it?
   ☐ Yes
   ☐ No
4. **Is there FGM ABCD guide in ANC consultation room?**
   ☐ Yes
   ☐ No
   4a. If yes, is it placed where ANC provider can see /use it
   ☐ Yes ☐
   No

Instructions: Assess health facility factors that may facilitate/constrain intervention delivery by reviewing health facility administrative records and notes and by meeting with the health facility manager.

5. **Number of ANC providers _________________**
6. **Average number of ANC clients per month_________________**
7. **Number of ANC providers trained on PCC on FGM prevention**
   ☐ All (specify number trained): ________
   ☐ Some (specify number trained): ________
   ☐ None
8. **Indicate the number of MoH supervisory visits to the clinic in the past year_________________**

BMJ Publishing Group Limited (BMJ) disclaims all liability and responsibility arising from any reliance placed on this supplemental material which has been supplied by the author(s)

A HEALTH SYSTEMS APPROACH TO PREVENTION OF FEMALE GENITAL MUTILATION USING PERSON-CENTRED COMMUNICATION: IMPLEMENTATION RESEARCH PROJECT IN GUINEA, SOMALIA AND KENYA (A65993)

ANC PROVIDER SCREENING QUESTIONNAIRE (SCR)

Participant ID:

Project ID: Country ID: Facility ID:

A	6	5	9	9	3

9. **How frequently are staff meetings held?**
   - ☐ Monthly
   - ☐ Every 2 to 4 months
   - ☐ Every 6 to 12months
   - ☐ Never
10. **What is the size of the population served by this facility? (*specify number)* ________________**
11. **Are there country/region-specific FGM laws that are enforced?**
    - ☐ Yes
    - ☐ No
12. **Are there anti-FGM activities that target the population served by this health facility?**
    - ☐ Yes
    - ☐ No
13. **Are there pro-FGM activities that target the population served by this health facility?**
    - ☐ Yes
    - ☐ No

Additional comments:

____
____
____
____

A HEALTH SYSTEMS APPROACH TO PREVENTION OF FEMALE GENITAL MUTILATION USING PERSON-CENTRED COMMUNICATION: IMPLEMENTATION RESEARCH PROJECT IN GUINEA, SOMALIA AND KENYA (A65993)

ANC PROVIDER SCREENING QUESTIONNAIRE (SCR)

Participant ID:

Project ID: Country ID: Facility ID:

A	6	5	9	9	3

1. **What is your age? _____________**
2. **What is your sex?**
   1. ☐ Female
   2. ☐ Male
3. **What is your religion?**
   1. ☐ Muslim
   2. ☐ Christian
   3. ☐ Other
   4. ☐ None
   5. ☐ Refused
4. **What is your occupation/designation?**
   1. ☐ Midwife
   2. ☐ Nurse
   3. ☐ Other, specify _____________________________________
5. **What is the highest education level of education you achieved?**
   1. ☐ Certificate
   2. ☐ Diploma
   3. ☐ Bachelors
   4. ☐ Masters or above
   5. ☐ Other, specify _____________________________________
6. **For how many years have you been working in your field? ________**
7. **During you clinical training, did you receive any formal training on female genital mutilation?**
   1. ☐ Yes.
   2. ☐ No. Go to section B
   3. ☐ I don’t know. Go to section B
8. **When did you receive the training?**
   1. ☐ During my studies (pre-service training)
   2. ☐ After graduation/at work (in-service training)
   3. ☐ Both
   4. ☐ I don’t know

   7. ☐ Not applicable .

A HEALTH SYSTEMS APPROACH TO PREVENTION OF FEMALE GENITAL MUTILATION USING PERSON-CENTRED COMMUNICATION: IMPLEMENTATION RESEARCH PROJECT IN GUINEA, SOMALIA AND KENYA (A65993)

ANC PROVIDER SCREENING QUESTIONNAIRE (SCR)

Participant ID:

Project ID: Country ID: Facility ID:

To be completed by data collector:

Data Collector ID:

Signature:

Date:

Day		Month			Year			
					2	0		

BMJ Publishing Group Limited (BMJ) disclaims all liability and responsibility arising from any reliance placed on this supplemental material which has been supplied by the author(s)

A HEALTH SYSTEMS APPROACH TO PREVENTION OF FEMALE GENITAL MUTILATION USING PERSON-CENTRED COMMUNICATION: IMPLEMENTATION RESEARCH PROJECT IN GUINEA, SOMALIA AND KENYA (A65993)

ANC PROVIDER SCREENING QUESTIONNAIRE (SCR)

Participant ID:

Project ID: Country ID: Facility ID:

A	6	5	9	9	3										

9. What was the format of the training? (Check all that apply)

1. ☐ Classroom lessons
2. ☐ Workshops
3. ☐ Digital format (E-learning videos; smart phone app)
4. ☐ During clinical practice under supervision of a mentor
5. ☐ Other, specify ____________________________________

7. ☐ Not applicable

10. During your pre- or post- graduate training, did you receive any formal training on communication or counselling?

1. ☐ Yes.
2. ☐ No.
3. ☐ I don't know

11. During you pre or post graduate training, did you receive any formal training on person-centred care?

1. ☐ Yes.
2. ☐ No.
3. ☐ I don't know

12. Have you ever cut the genitals of a girl (<=18 years old) for non-health reasons?

1. ☐ Yes.
2. ☐ No.
3. ☐ I don't know

.

Balde MD, *et al. BMJ Open* 2024; 14:e078771. doi: 10.1136/bmjopen-2023-078771

A HEALTH SYSTEMS APPROACH TO PREVENTION OF FEMALE GENITAL MUTILATION USING PERSON-CENTRED COMMUNICATION: IMPLEMENTATION RESEARCH PROJECT IN GUINEA, SOMALIA AND KENYA (A65993)

ANC PROVIDER QUESTIONNAIRE (HCP)

Participant ID:

Project ID: Country ID: Facility ID:

A	6	5	9	9	3

To be completed by data collector:

Data Collector ID:

Signature:

Date:

Day		Month			Year			
					2	0		

1. **Have you ever heard about female genital mutilation?**
   ☐ Yes
   ☐ No
2. **Do the women in your community undergo female genital mutilation?**
   ☐ Yes
   ☐ No
   ☐ I don't know
3. **Do you know of the WHO classification for female genital mutilation?**
   ☐ Yes
   ☐ No. Skip to Q5
4. **Please provide the WHO classification for the following FGM images (to include images)**
   a. **IMAGE of Type III FGM to be inserted here**
      i. ☐ **Type I**
      ii. ☐ **Type II**
      iii. ☐ **Type III**
      iv. ☐ **Type IV**
      v. ☐n**Don't know**
   b. **IMAGE of Type I FGM to be inserted here**
      i. ☐ **Type I**
      ii. ☐ **Type II**
      iii. ☐ **Type III**

To be completed by data collector:

Data Collector ID:

Signature:

Date:

Day		Month			Year			
					2	0		

A HEALTH SYSTEMS APPROACH TO PREVENTION OF FEMALE GENITAL MUTILATION USING PERSON-CENTRED COMMUNICATION: IMPLEMENTATION RESEARCH PROJECT IN GUINEA, SOMALIA AND KENYA (A65993)

ANC PROVIDER QUESTIONNAIRE (HCP)

Participant ID:

Project ID: Country ID: Facility ID:

iv. ☐ **Type IV**

v. ☐n**Don't know**

c. **IMAGE of Type II FGM to be inserted here**

i. ☐ **Type I** ii. ☐ **Type II**

A 6 5 9 9 3

iii. ☐ **Type III**

iv. ☐ **Type IV**

v. ☐n**Don't know**

d. **IMAGE of Type III FGM to be inserted here**

i. ☐ **Type I**

ii. ☐ **Type II**

iii. ☐ **Type III**

iv. ☐ **Type IV**

v. ☐n**Don't know**

e.

5. Do you know of any health complications arising from female genital mutilation?

☐ Yes

☐ No. Skip to Q6

6. Is female genital mutilation illegal in your country (specify actual study country)?

1. ☐ Yes
2. ☐ No
3. ☐ I don't know

7. Are you aware of any existing WHO tools/guidance on female genital mutilation and its complications?

1. ☐ Yes. *If yes, please specify*…………………………………………………………….
2. ☐ No

To be completed by data collector:

Data Collector ID:	*Date:*									
Signature:	Day		Month			Year				
						2	0			

A HEALTH SYSTEMS APPROACH TO PREVENTION OF FEMALE GENITAL MUTILATION USING PERSON-CENTRED COMMUNICATION: IMPLEMENTATION RESEARCH PROJECT IN GUINEA, SOMALIA AND KENYA (A65993)

ANC PROVIDER QUESTIONNAIRE (HCP)

Participant ID: | | | | |

Project ID: | A | 6 | 5 | 9 | 9 | 3 |

Country ID: | | | | |

Facility ID: | | | | | |

8. **When you treat or attend a girl or a woman with genital mutilation, how confident are you that you have enough knowledge to provide good quality health care?** *Rate between 1 – 4*

1. ☐ Not confident at all
2. ☐ Not very confident
3. ☐ Fairly confident
4. ☐ Fully confident

9. **How confident are you in your FGM knowledge to communicate on FGM prevention?** *Rate between 1 – 4*

1. ☐ Not confident at all
2. ☐ Not very confident
3. ☐ Fairly confident
4. ☐ Fully confident

For each of the following statements please state if you agree/disagree or don't know.

10. **A girl who has not undergone FGM is unclean.**

1. ☐ Agree
2. ☐ Disagree
3. ☐ I don't know

11. **A girl without FGM cannot be married within her community.**

1. ☐ Agree
2. ☐ Disagree
3. ☐ I don't know

12. **A girl who has not undergone FGM is a disgrace to her family's honour.**

1. ☐ Agree
2. ☐ Disagree

To be completed by data collector:

Data Collector ID: *Date:*

Signature:

Day		Month			Year			
					2	0		

A HEALTH SYSTEMS APPROACH TO PREVENTION OF FEMALE GENITAL MUTILATION USING PERSON-CENTRED COMMUNICATION: IMPLEMENTATION RESEARCH PROJECT IN GUINEA, SOMALIA AND KENYA (A65993)

ANC PROVIDER QUESTIONNAIRE (HCP)

Participant ID:

Project ID:						Country ID:			Facility ID:	
A	6	5	9	9	3					

3. ☐ I don't know

13. Health care providers who perform FGM are violating medical ethics.
1. ☐ Agree
2. ☐ Disagree
3. ☐ I don't know

14. Health care providers who perform FGM should be punished.
1. ☐ Agree
2. ☐ Disagree
3. ☐ I don't know

15. FGM is a good practice
1. ☐ Agree
2. ☐ Disagree
3. ☐ I don't know

16. FGM is a violation of women's and girls' rights
1. ☐ Agree
2. ☐ Disagree
3. ☐ I don't know

17. FGM is a religious mandate
1. ☐ Agree
2. ☐ Disagree
3. ☐ I don't know

18. Pretend you had a daughter now who was at an age when cutting occurs, what would your intention to cut her be?
1. ☐ Intend to cut her
2. ☐ Do not intend to cut her
3. ☐ Undecided
4. Refused to answer

19. If a family brought their daughter to the clinic requesting genital cutting for non-health reasons, would you perform it?

To be completed by data collector:

Data Collector ID: *Date:*

Signature:

Day		Month			Year			
					2	0		

A HEALTH SYSTEMS APPROACH TO PREVENTION OF FEMALE GENITAL MUTILATION USING PERSON-CENTRED COMMUNICATION: IMPLEMENTATION RESEARCH PROJECT IN GUINEA, SOMALIA AND KENYA (A65993)

ANC PROVIDER QUESTIONNAIRE (HCP)

Participant ID:

Project ID:

A	6 5	9	9	3	

Country ID:

Facility ID:

1. ☐ Yes
2. ☐ No
3. ☐ I don't know

Now I would like to ask you a few questions about how you solve problems that you face. Please state how much you agree or disagree with the statements that I read, , where 1=Strongly disagree; 2=Disagree; 3=Neither agree nor disagree; 4=Agree; 5=Strongly agree

20. I will be able to achieve most of the goals that I have set for myself.

1. ☐ **Strongly disagree**
2. ☐ **Disagree**
3. ☐ **Neither agree nor disagree**
4. ☐ **Agree**
5. ☐ **Strongly agree**
6. ☐ **Don't know**

21. When facing difficult tasks, I am certain that I will accomplish them.

1. ☐ **Strongly disagree**
2. ☐ **Disagree**
3. ☐ **Neither agree nor disagree**
4. ☐ **Agree**
5. ☐ **Strongly agree**
6. ☐ **Don't know**

22. In general, I think that I can obtain outcomes that are important to me.

1. ☐ **Strongly disagree**
2. ☐ **Disagree**
3. ☐ **Neither agree nor disagree**

To be completed by data collector:

Data Collector ID:

Signature:

Date:

Day		Month			Year			
					2	0		

A HEALTH SYSTEMS APPROACH TO PREVENTION OF FEMALE GENITAL MUTILATION USING PERSON-CENTRED COMMUNICATION: IMPLEMENTATION RESEARCH PROJECT IN GUINEA, SOMALIA AND KENYA (A65993)

ANC PROVIDER QUESTIONNAIRE (HCP)

Participant ID: | | | | |

Project ID: | | | | | | | Country ID: | | | | | Facility ID: | | | | | |

4. ☐ **Agree**
5. ☐ **Strongly agree**
6. ☐ **Don't know**

23. I believe I can succeed at most any endeavour to which I set my mind.

1. ☐ **Strongly disagree**
2. ☐ **Disagree**
3. ☐ **Neither agree nor disagree**
4. ☐ **Agree**
5. ☐ **Strongly agree**
6. ☐ **Don't know**

24. I will be able to successfully overcome many challenges.

1. ☐ **Strongly disagree**
2. ☐ **Disagree**
3. ☐ **Neither agree nor disagree**
4. ☐ **Agree**
5. ☐ **Strongly agree**

A 6 5 9 9 3

6. ☐ **Don't know**

25. I am confident that I can perform effectively on many different tasks.

1. ☐ **Strongly disagree**
2. ☐ **Disagree**
3. ☐ **Neither agree nor disagree**
4. ☐ **Agree**
5. ☐ **Strongly agree**
6. ☐ **Don't know**

To be completed by data collector:

Data Collector ID: *Date:*

Signature:

Day		Month			Year			
					2	0		

A HEALTH SYSTEMS APPROACH TO PREVENTION OF FEMALE GENITAL MUTILATION USING PERSON-CENTRED COMMUNICATION: IMPLEMENTATION RESEARCH PROJECT IN GUINEA, SOMALIA AND KENYA (A65993)

ANC PROVIDER QUESTIONNAIRE (HCP)

Participant ID:

Project ID: Country ID: Facility ID:

26. Compared to other people, I can do most tasks very well.

1. ☐ **Strongly disagree**
2. ☐ **Disagree**
3. ☐ **Neither agree nor disagree**
4. ☐ **Agree**
5. ☐ **Strongly agree**
6. ☐ **Don't know**

27. Even when things are tough, I can perform quite well.

1. ☐ **Strongly disagree**
2. ☐ **Disagree**
3. ☐ **Neither agree nor disagree**
4. ☐ **Agree**
5. ☐ **Strongly agree**
6. ☐ **Don't know**

A 6 5 9 9 3

28. Would you like to receive more training related to care for women and girls with FGM?

1. ☐ Yes
2. ☐ No
3. ☐ I don't know

29. If a pregnant woman is expected to have a girl, do you discourage her from having her daughter cut?

To be completed by data collector:

Data Collector ID: *Date:*

Signature:

Day		Month			Year			
					2	0		

A HEALTH SYSTEMS APPROACH TO PREVENTION OF FEMALE GENITAL MUTILATION USING PERSON-CENTRED COMMUNICATION: IMPLEMENTATION RESEARCH PROJECT IN GUINEA, SOMALIA AND KENYA (A65993)

ANC PROVIDER QUESTIONNAIRE (HCP)

Participant ID:

Project ID: Country ID: Facility ID:

1. ☐ Always
2. ☐ Often
3. ☐ Sometimes
4. ☐ Rarely
5. ☐ Never

30. If you heard of or saw a colleague performing female genital mutilation, what would you do? ***(Tick all that apply)***

1. ☐ I would report him/her to the authorities
2. ☐ I would discuss with him/her and explain to him/her that health care providers should not perform female genital mutilation
3. ☐ I would not get involved 4. ☐ I don't know

31. How often do you look for female genital cutting/excision when performing a gynecological examination of the vulva?

1. ☐ Always
2. ☐ Often
3. ☐ Sometimes
4. ☐ Rarely
5. ☐ Never

32. How often do you record the female genital mutilation in the women's medical file if you are aware that she has undergone FGM?

1. ☐ Always
2. ☐ Often
3. ☐ Sometimes
4. ☐ Rarely
5. ☐ Never

33. Would you like to receive more training on how to help patients to prevent FGM?

1. ☐ Yes
2. ☐ No
3. ☐ I don't know

A	6	5	9	9	3

34. I can put myself in others' shoes

1. ☐ Always
2. ☐ Often

To be completed by data collector:

Data Collector ID: *Date:*

Signature:

Day		Month			Year			
					2	0		

A HEALTH SYSTEMS APPROACH TO PREVENTION OF FEMALE GENITAL MUTILATION USING PERSON-CENTRED COMMUNICATION: IMPLEMENTATION RESEARCH PROJECT IN GUINEA, SOMALIA AND KENYA (A65993)

ANC PROVIDER QUESTIONNAIRE (HCP)

Participant ID:

Project ID: Country ID: Facility ID:

A	6	5	9	9	3

3. ☐ Sometimes
4. ☐ Rarely
5. ☐ Never

35. I let others know I understand what they say

1. ☐ Always
2. ☐ Often
3. ☐ Sometimes
4. ☐ Rarely
5. ☐ Never

36. In conversations with my colleagues, I perceive not only what they say but what they don't say

1. ☐ Always
2. ☐ Often
3. ☐ Sometimes
4. ☐ Rarely
5. ☐ Never

37. I communicate effectively

1. ☐ Always
2. ☐ Often
3. ☐ Sometimes
4. ☐ Rarely
5. ☐ Never

38. I communicate with others as though they are my equals

1. ☐ Always
2. ☐ Often
3. ☐ Sometimes
4. ☐ Rarely
5. ☐ Never

To be completed by data collector:

Data Collector ID: *Date:*

Signature:

Day		Month			Year			
					2	0		

A HEALTH SYSTEMS APPROACH TO PREVENTION OF FEMALE GENITAL MUTILATION USING PERSON-CENTRED COMMUNICATION: IMPLEMENTATION RESEARCH PROJECT IN GUINEA, SOMALIA AND KENYA (A65993)

ANC PROVIDER QUESTIONNAIRE (HCP)

Participant ID: | | | | |

| | | | | | | Project ID: | | | | | Country ID: | | | Facility ID: | |

These next questions relate to your clinic setting:

39. Have you seen the posters on FGM at the clinic?
1. ☐ Yes
2. ☐ No
3. ☐ I don't know

40. Have you referred to the clinical handbook on FGM that is available in your clinic?
1. ☐ No
2. ☐ I don't know

41. Do you think it is feasible to provide FGM prevention counselling during ANC visits?
1. ☐ Yes
2. ☐ No
3. ☐ I don't know

Comments

-
____
____
____
____
____

To be completed by data collector:

Data Collector ID: *Date:*

Signature:

Day		Month			Year			
					2	0		

A HEALTH SYSTEMS APPROACH TO PREVENTION OF FEMALE GENITAL MUTILATION USING PERSON-CENTRED COMMUNICATION: IMPLEMENTATION RESEARCH PROJECT IN GUINEA, SOMALIA AND KENYA (A65993)

Participant ID:

Project ID: Facility ID:

FIRST ANC CLIENT EXIT QUESTIONNAIRE (EXT)

Country ID:

A 6 5 9 9 3

1. **How old are you? (years)**_______________________________________
2. **What is your religion?**
   1. ☐ Muslim
   2. ☐ Christian
   3. ☐ Other
   4. ☐ None
   5. ☐ Refused
3. **What is the highest level of education you achieved?**
   1. ☐ None
   2. ☐ Primary
   3. ☐ Secondary
   4. ☐ University
   5. ☐ Other, s*pecify* _______________________________________
4. **Many women in your community have had their genitals cut when they were children, if you are comfortable telling me, can I ask if you have undergone this practice?**
   1. ☐ Yes
   2. ☐ No
   3. ☐ I don't know
   4. ☐ Refused
5. **How supportive are you of female genital mutilation?**
   1. ☐ Strongly opposed
   2. ☐ Somewhat opposed
   3. ☐ Neutral (Neither opposed or supportive)
   4. ☐ Somewhat supportive
   5. ☐ Strongly supportive

The following questions relate to your visit today. During your visit today:

6. **Did you see any FGM poster(s) in the waiting room?**
   1. ☐ Yes
   2. ☐ No
   3. ☐ I don't know

A HEALTH SYSTEMS APPROACH TO PREVENTION OF FEMALE GENITAL MUTILATION USING PERSON-CENTRED COMMUNICATION: IMPLEMENTATION RESEARCH PROJECT IN GUINEA, SOMALIA AND KENYA (A65993)

Participant ID:

Project ID: Facility ID:

7. Did the ANC provider ask if you have undergone FGM?

1. ☐ Yes
2. ☐ No
3. ☐ I don't know

8. Did the ANC provider explain how FGM can harm your health?

1. ☐ Yes
2. ☐ No

Country ID:

A 6 5 9 9 3

3. ☐ I don't know

9. Did the ANC provider ask about your personal belief regarding FGM?

1. ☐ Yes
2. ☐ No
3. ☐ I don't know

10. Did the ANC provider discuss why FGM should be prevented?

1. ☐ Yes
2. ☐ No
3. ☐ I don't know

11. Did the ANC provider discuss how FGM could be prevented?

1. ☐ Yes
2. ☐ No
3. ☐ I don't know

12. Did you have questions about FGM to ask the ANC provider?

1. ☐ Yes
2. ☐ No
3. ☐ I don't know

13. Did you feel encouraged to ask questions about FGM?

1. ☐ Yes
2. ☐ No
3. ☐ I don't know

14. Are you satisfied with how FGM was addressed during your visit with your ANC provider today?

A HEALTH SYSTEMS APPROACH TO PREVENTION OF FEMALE GENITAL MUTILATION USING PERSON-CENTRED COMMUNICATION: IMPLEMENTATION RESEARCH PROJECT IN GUINEA, SOMALIA AND KENYA (A65993)

Participant ID: ☐☐☐☐

☐☐☐☐☐☐ Project ID: ☐☐☐☐ Facility ID: ☐☐☐☐☐

1. ☐ Yes
2. ☐ No
3. ☐ I don't know

15. What do you feel about FGM now as compared to before you came to the clinic today?

1. ☐ Same, no change
2. ☐ I feel more supportive of FGM now as compared to before I came
3. ☐ I feel less supportive of FGM now as compared to before I came
4. ☐ I do not know
5. ☐ Other, s*pecify* ____________________________________

16. Pretend you had a daughter now who was at an age when cutting occurs, what would your intention to cut her be?

1. ☐ Intend to cut her
2. ☐ Do not intend to cut her

17. Do you wish/want to be active in preventing FGM?

1. ☐ Yes
2. ☐ No
3. ☐ I don't know
