# Supplementary material for: Cluster randomised trial of a health system strengthening approach applying person-centred communication for the prevention of female genital mutilation in Guinea, Kenya and Somalia
Source: BMJ Open. 2024 Jul 4;14(7):e078771. doi: 10.1136/bmjopen-2023-078771 (PMC11227771; doi:10.1136/bmjopen-2023-078771)
Supplement: Supplementary data [file bmjopen-2023-078771supp004.pdf]

Supplementary file 3: Additional analyses (appendices 1 – 3)

Appendix 1: Comparison baseline characteristics of ANC facilities

Characteristics	Facilities included in final analysis (n=163)	Facilities excluded* from final analysis (n=17)
Number of ANC providers	Mean 4 (SD: 3) Median 3 (1-14, IQR 3)	Mean 3 (SD: 3) Median 2 (1-9, IQR 1)
Average number of ANC clients/month	Mean 150 (SD: 127) Median 118 (3-664, IQR 141)	Mean 162 (SD: 147) Median 100 (25-600, IQR 200)
MoH supervisory visits in the past year	Mean 4 (SD: 3) Median 3 (0-18, IQR 2)	Mean 5 (SD: 4) Median 4 (0-12, IQR 4)
Size of catchment population served	Mean 36,754 (SD: 126,082) Median 15,972 (1,000-1,458,000, IQR 24,332)	Mean 11,737 (SD: 14,62) Median 7,800 (1,200-63,000, IQR 7,505
Presence of anti-FGM activities in the catchment area		
Yes	74 (45%)	9 (53%)
No	89 (55%)	8 (47%)
Presence of pro-FGM activities in the catchment area		
Yes	21 (13%)	2 (12%)
No	140 (86%)	15 (88%)
Don't Know	2 (1%)	-

*Total of ANC clinics not included: 16 clinics were excluded (7 intervention and 9 control) due to loss-to-follow up (LTFU) of ANC provider i.e., the clinics did not have at least one ANC provider present across all study time points while one ANC clinic in Kenya was never visited at subsequent time points due to issues with insecurity. An ANC provider from one of the clinics in Kenya that had been inaccessible due to insecurity attended the PCC training and was subsequently interviewed.

Appendix 2: Comparison of baseline characteristics of ANC providers

**Characteristics**	**Providers recruited at Baseline (n=436)**	**Providers enrolled with complete data at Month 6 (n=232)**	**Providers not enrolled with no data at Month 6 (n=204)**
Age	37 (20-65; SD: 10)	36 (20-65; SD: 10)	38 (21-62; SD: 10)
Years of professional experience	9 (1-39; SD: 7)	8 (1-39; SD: 7)	10 (1-36; SD: 8)
Sex			
Female	361 (83%)	193 (83%)	168 (82%)
Male	75 (17%)	39 (17%)	36 (18%)
Highest educational level			
Certificate	44 (3%)	21 (5%)	23 (11%)
Diploma	309 (71%)	158 (68%)	151 (74%)
Bachelors	64 (15%)	44 (19%)	20 (10%)
Masters & above	3 (0.7%)	1 (0.4%)	2 (1%)
Other[#]	16 (4%)	8 (3%)	8 (4%)
Current professional role/title			
Midwife	198 (45%)	103 (44%)	95 (47%)
Nurse	95 (22%)	51 (22%)	44 (22%)
Nurse-Midwife	94 (22%)	54 (23%)	40 (20%)
Other	49 (11%)	24 (10%)	25 (12%)
Received formal training on FGM during clinical training			
Yes	158 (36%)	85 (37%)	73 (36%)
No	275 (63%)	146 (63%)	129 (63%)
Don't Know	3 (0.7%)	1 (0.4%)	2 (1%)
Timing of clinical training on FGM			
Pre-service	63 (14%)	33 (14%)	30 (15%)
In-service	81 (19%)	45 (19%)	36 (18%)
Both pre- and in-service	14 (3%)	7 (3%)	7 (3%)
Received formal training on communication/counselling			
Yes	287 (66%)	149 (64%)	138 (68%)
No	149 (34%)	83 (36%)	66 (32%)
Received formal training on person-centered care			
Yes	227 (52%)	118 (51%)	109 (53%)
No	207 (47%)	131 (56%)	94 (46%)
Don't know	2 (0.5%)	1 (0.4%)	1 (0.5%)

Characteristics	Providers recruited at Baseline (n=436)	Providers enrolled with complete data at Month 6 (n=232)	Providers not enrolled with no data at Month 6 (n=204)
Undergone FGM			
Yes	226 (52%)	126 (54%)	100 (49%)
No	128 (29%)	63 (27%)	65 (32%)
Don't know	4 (0.9%)	2 (1%)	1 (0.5%)
Refused to answer	3 (0.7%)	2 (1%)	1 (0.55)
Conducted FGM			
Yes	35 (8%)	15 (7%)	20 (10%)
Conducted FGM on a girl <18 years			
Yes	32 (7%)	14 (6%)	18 (9%)

Appendix 3: Comparison of study outcomes between baseline vs. month 3 and month 3 vs. month 6 in the intervention arm

	**Baseline (Intervention only)**	**Month 3 (Intervention only)**	**P-value**	**Month 3 (Intervention only)**	**Month 6 (Intervention only)**	**P-value**
**Primary Outcomes**						
**ANC clients reporting that their provider implemented components of PCC for FGM prevention**						
Provider asked client if they have undergone FGM	48 (6%)	298 (37%)	<0.0001	298 (37%)	694 (78%)	<0.0001
Provider asked client about their (client's) personal beliefs regarding FGM	38 (5%)	239 (29%)	<0.0001	239 (29%)	616 (76%)	<0.0001
Provider discussed with client why FGM should be prevented	56 (7%)	243 (30%)	<0.0001	243 (30%)	629 (77%)	<0.0001
Provider discussed with client how FGM could be prevented	48 (6%)	224 (28%)	<0.0001	224 (28%)	592 (73%)	<0.0001
Client satisfied with how FGM was addressed by provider during clinic visit	176 (21%)	346 (43%)	<0.0001	346 (43%)	684 (84%)	<0.0001
Mean score of PCC approach (out of 5)	0.5 (0.4-0.5)	1.7 (1.5-1.8)	<0.0001	1.7 (1.5-1.8)	3.9 (3.8-4.0)	<0.0001
Mean score of PCC + appropriate FGM prevention & care (out of 8)	1.8 (1.6-2.1)	3.3 (2.8-3.8)	<0.0001	3.3 (2.8-3.8)	6.2 (5.9 – 6.6)	<0.0001
**ANC clinic preparedness to offer FGM prevention and care services**						
Clinics with ALL correct answers for facility preparedness	0 (0%)	42 (52%)	<0.0001	42 (52%)	56 (69%)	<0.01
Mean score of clinic preparedness (out of 4)	0.1 (0.01-0.2)	3.1 (2.9-3.4)	<0.0001	3.1 (2.9-3.4)	3.4 (3.2-3.6)	0.18
Providers using level 1 intervention package	1 (1%)	61 (58%)	<0.0001	61 (58%)	96 (91%)	<0.0001
Providers offering appropriate FGM-related prevention and care services	11 (11%)	20 (19%)	<0.0001	20 (19%)	52 (50%)	<0.0001
**Secondary Outcomes**						
Providers with correct FGM-related knowledge responses	0 (0%)	1 (3%)	0.47	1 (3%)	8 (8%)	0.06
Providers with appropriate interpersonal communication skills	49 (49%)	62 (59%)	0.08	62 (59%)	74 (70%)	0.11
Providers with high self-efficacy	85 (85%)	94 (90%)	0.18	94 (90%)	86 (82%)	0.17
Providers reporting less supportive attitudes towards FGM	67 (67%)	75 (71%)	0.26	75 (71%)	76 (72%)	0.50
Providers with high confidence scores	84 (83%)	81 (77%)	0.30	81 (77%)	103 (98%)	<0.001
Providers not supportive of FGM	91 (91%)	101 (96%)	0.16	101 (96%)	100 (96%)	1.0
Providers not supportive of medicalized FGM	98 (97%)	104 (99%)	0.36	104 (99%)	104 (99%)	0.75
**Other ANC Client Outcomes**						
Clients reporting less support for FGM after ANC clinic visit	194 (24%)	235 (29%)	0.01	235 (29%)	424 (52%)	<0.0001
Clients reporting that they were strongly opposed to FGM	367 (45%)	345 (43%)	0.38	345 (43%)	498 (61%)	<0.0001
Clients reporting that they intend to have their daughters cut	249 (30%)	184 (23%)	<0.0001	184 (23%)	96 (12%)	<0.0001
Clients reporting that they would prefer health care provider to cut daughters	141 (17%)	117 (14%)	0.003	117 (14%)	53 (7%)	<0.001
Clients wishing to be active in FGM prevention	530 (65%)	547 (68%)	0.22	547 (68%)	677 (83%)	<0.001
