# Supplementary material for: Cluster randomised trial of a health system strengthening approach applying person-centred communication for the prevention of female genital mutilation in Guinea, Kenya and Somalia
Source: BMJ Open. 2024 Jul 4;14(7):e078771. doi: 10.1136/bmjopen-2023-078771 (PMC11227771; doi:10.1136/bmjopen-2023-078771)
Supplement: Author's manuscript [file bmjopen-2023-078771.draft_revisions.pdf]

BMJ Open

BMJ Open is committed to open peer review. As part of this commitment we make the peer review history of every article we publish publicly available.

When an article is published we post the peer reviewers' comments and the authors' responses online. We also post the versions of the paper that were used during peer review. These are the versions that the peer review comments apply to.

The versions of the paper that follow are the versions that were submitted during the peer review process. They are not the versions of record or the final published versions. They should not be cited or distributed as the published version of this manuscript.

BMJ Open is an open access journal and the full, final, typeset and author-corrected version of record of the manuscript is available on our site with no access controls, subscription charges or pay-per-view fees (http://bmjopen.bmj.com).

If you have any questions on BMJ Open's open peer review process please email info.bmjopen@bmj.com

BMJ Open

A cluster randomized trial of a health system strengthening approach applying person-centered communication for the prevention of female genital mutilation in Guinea, Kenya, and Somalia

Journal:	*BMJ Open*
Manuscript ID	bmjopen-2023-078771
Article Type:	Original research
Date Submitted by the Author:	11-Aug-2023

Complete List of Authors:	Balde, Mamadou ; Cellulle de Recherche en Sante de la Reproduction en Guinee (CERREGUI)<br>Ndavi, Patrick; University of Nairobi College of Health Sciences, Department of Obstetrics & Gynecology<br>Oyaro, Vernon; World Health Organization, Department of Sexual and Reproductive Health and Research<br>Soumah, Anne-Marie; Cellulle de Recherche en Sante de la Reproduction en Guinee (CERREGUI)<br>Esho, Tammary; Amref International University<br>King'oo, James; Technical University of Kenya<br>Kemboi , Jackline; Amref Health Africa<br>Sall, Alpha; Cellulle de Recherche en Sante de la Reproduction en Guinee (CERREGUI)<br>Diallo, Aissatou; Cellulle de Recherche en Sante de la Reproduction en Guinee (CERREGUI)<br>Ahmed, Wisal; World Health Organization, Department of Sexual and Reproductive Health and Research<br>Stein, Karin; World Health Organization<br>Nosirov, Khurshed; World Health Organization, Department of Sexual and Reproductive Health and Research<br>Thwin, Soe Soe; World Health Organization, Department of Sexual and Reproductive Health and Research, including UNDP/UNFPA/UNICEF/WHO/World Bank Special Programme of Research, Development and Research Training in Human Reproduction (HRP)<br>Petzold, Max; University of Gothenburg Sahlgrenska Academy, Public Health and Community Medicine<br>Ahmed, Muna; Somaliland Central Statistics Department<br>Diriye, Ahmed; Data and Research Solutions<br>Pallitto, C ; World Health Organization, Department of Sexual and Reproductive Health and Research

Keywords:	EDUCATION & TRAINING (see Medical Education & Training), Patient-Centered Care, Primary Care < Primary Health Care, Primary Prevention, PUBLIC HEALTH, Behavior

I, the Submitting Author has the right to grant and does grant on behalf of all authors of the Work (as defined in the below author licence), an exclusive licence and/or a non-exclusive licence for contributions from authors who are: i) UK Crown employees; ii) where BMJ has agreed a CC-BY licence shall apply, and/or iii) in accordance with the terms applicable for US Federal Government officers or employees acting as part of their official duties; on a worldwide, perpetual, irrevocable, royalty-free basis to BMJ Publishing Group Ltd ("BMJ") its licensees and where the relevant Journal is co-owned by BMJ to the co-owners of the Journal, to publish the Work in this journal and any other BMJ products and to exploit all rights, as set out in our licence.

The Submitting Author accepts and understands that any supply made under these terms is made by BMJ to the Submitting Author unless you are acting as an employee on behalf of your employer or a postgraduate student of an affiliated institution which is paying any applicable article publishing charge ("APC") for Open Access articles. Where the Submitting Author wishes to make the Work available on an Open Access basis (and intends to pay the relevant APC), the terms of reuse of such Open Access shall be governed by a Creative Commons licence – details of these licences and which Creative Commons licence will apply to this Work are set out in our licence referred to above.

Other than as permitted in any relevant BMJ Author's Self Archiving Policies, I confirm this Work has not been accepted for publication elsewhere, is not being considered for publication elsewhere and does not duplicate material already published. I confirm all authors consent to publication of this Work and authorise the granting of this licence.

For peer review only

A cluster randomized trial of a health system strengthening approach applying person-centered communication for the prevention of female genital mutilation in Guinea, Kenya, and Somalia

Authors: *Prof. Mamadou Balde, MD[1*], Prof. Patrick Ndavi, MMed[2*], Dr. Vernon Mochache, PhD[3] Dr. Anne-Marie Soumah, MSc[1], Prof. Tammary Esho, PhD[4], James Munyao King'oo, MSc[5], Jackline Kemboi, MSc[2], Alpha Oumar Sall, MSc[1], Aissatou Diallo, MSc[1], Dr. Wisal Ahmed, PhD[3], Dr. Karin Stein, MD[3], Khurshed Nosirov, MCS[3], Dr. Soe Soe Thwin, PhD[3], Prof. Max Petzold, PhD[6], Muna Abdi Ahmed, MSc[7], Ahmed Diriye, MA[8], Dr. Christina Pallitto, PhD[3]*

Institutional Affiliations: [1]Centre for Research in Reproductive Health in Guinea, Conakry, Guinea; [2]Department of Obstetrics and Gynecology, University of Nairobi, Nairobi, Kenya; [3]Department of Sexual and Reproductive Health and Research, and the UNDP/UNFPA/UNICEF/WHO/World Bank Special Programme of Research, Development and Research Training in Human Reproduction (HRP), World Health Organization, Geneva, Switzerland; [4]Amref International University, Nairobi, Kenya; [5]Technical University of Kenya, Nairobi, Kenya; [6]School of Public Health and Community Medicine, Institute of Medicine, University of Gothenburg, Gothenburg, Sweden; [7] Somaliland Central Statistics Department, Hargeisa, Somalia; [8]Data and Research Solutions, Hargeisa, Somalia

** Joint first authors*

Correspondence to:

Dr. Christina Pallitto,

World Health Organization,

Department of Sexual and Reproductive Health and Research

20 Avenue Appia, 1211, Geneva, Switzerland

Telephone: +41 22 791 4745

For peer review only

Email: pallittoc@who.int

Abstract word count: 286

Text word count: 5,048

Tables: 3

Figures: 2

For peer review only

ABSTRACT

Introduction: There is limited evidence on effective health systems interventions for preventing female genital mutilation (FGM). This study tested a two-level health system strengthening approach at primary care level to apply person-centred communication (PCC) for FGM prevention.

Methods: Between August 2020 and September 2021, a cluster randomized trial was conducted in 180 antenatal care (ANC) clinics in Guinea, Kenya, and Somalia. At baseline, all clinics received guidance and materials on FGM prevention and care, while at month three, ANC providers at intervention sites received PCC training. Data were collected from clinic managers, ANC providers and clients at baseline, months three and six. Logistic regression models were used to analyze the effect of the intervention on study outcomes.

Results: Complete data were collected from 232 ANC providers in 163 clinics. Compared to providers in the control arm, those in the intervention arm had higher odds of being confident in their FGM-related knowledge (OR: 6.3, 95% CI: 1.4-28.9; p=0.02) and to communicate effectively about FGM prevention (OR: 1.7; 95% CI: 1.0-3.0; p=0.06). Additionally, ANC clients in the intervention arm had higher odds of being less supportive of FGM (OR: 2.4, 95% CI: 2.0-3.0; p<0.001) and wanting to be actively engaged in FGM prevention (OR: 2.2, 95% CI: 1.8-2.9; p<0.001) after speaking with their provider. They also had higher odds of being strongly opposed to FGM (OR: 1.7, 95% CI: 1.4-2.1; p<0.001), lower odds of intending to have their daughters undergo FGM (OR: 0.4, 95% CI: 0.3-0.5; p<0.001) or seeking medicalized FGM (OR: 0.4, 95% CI: 0.3-0.5; p<0.001).

For peer review only

Conclusion: This is the first randomized trial to provide evidence of an effective intervention to promote FGM prevention in primary care settings that could be scaled up in high prevalence countries.

SUMMARY BOX

- The health sector has the potential to complement existing efforts to prevent female genital mutilation (FGM) through its large primary care service network. Health workers can be influential in health promotion and behaviour change given their respected status in their communities, their access to at-risk populations and the support and access to capacity building they receive through the health system. There has been limited rigorous research examining effective interventions that empower health workers to communicate on FGM prevention that could be brought to scale to support global and national efforts for FGM abandonment.
- Based on concepts from social behavioural theory and person-centred care, as well as learnings from formative research conducted in Guinea and consultations with key stakeholders in high prevalence settings, we developed and pre-tested a training package to enable ANC providers working at primary care clinics in FGM prevalent settings to provide person-centered communication on FGM prevention to their clients. Results from the present study show that ANC providers effectively implemented this FGM counselling approach and their clients were significantly more satisfied by the care provided, had lower intentions to perform FGM on their daughters and greater willingness to be engaged in FGM abandonment efforts.

For peer review only

- To our knowledge, this is the first randomized controlled trial that showed the effectiveness of a health system intervention to promote FGM prevention communication in the context of routine primary care.
- Further research is needed to understand how to replicate and scale-up existing findings in other settings and how to reinforce prevention messages over the long-term. This study highlights the need for greater multi-sectoral coordination and complementarity in programming in high prevalence settings.

For peer review only

INTRODUCTION

Multi-sectoral efforts are needed to achieve Sustainable Development Goal (SDG) 5.3 to eliminate the harmful practice of female genital mutilation (FGM) by 2030 in line with the United Nation's (UN) General Assembly resolution 67/146 (1), the World Health Assembly Resolution 61.16 (2) and the 2008 Interagency Statement (3), which call upon UN Member States to enact comprehensive and multi-disciplinary national action plans and strategies towards the elimination of the practice. Identifying effective strategies across sectors is an important step in ending FGM

The health system, defined as all organizations, institutions and resources that produce actions whose primary purpose is to improve health (4), has an important role to play not only in managing complications of FGM but also in preventing the practice. Health care providers, specifically nurses and midwives who constitute most of the health workforce, are highly respected members of FGM practising communities and could positively contribute to abandonment efforts (5,6). However, there is currently limited evidence to guide health programming on FGM prevention (7). In addition, some health care providers are themselves

supportive of this harmful practice, and might even perform it (i.e., FGM medicalization), despite national laws and medical ethics forbidding it (8–11). Developing evidence-based tools to build skills of health care providers and address their underlying beliefs could contribute to FGM abandonment efforts and complement existing resources on management of complications (12,13) to ensure comprehensive and high quality care.

Three countries (Guinea, Somalia, and Kenya) participated in a cluster randomized trial to test the effectiveness and implementation of a health system strengthening approach to FGM, which included the testing of an intervention to build skills of health workers on applying person-centered communication (PCC) for the prevention of FGM (14). Study countries were selected based on their high national and/or sub-national FGM prevalence. The national prevalence of FGM among women and girls aged 15 - 49 years is 98% in Somalia, 97% in Guinea and 21% in Kenya according to national population-based surveys. There are 20 hotspot counties/sub-national administrative units in Kenya with a prevalence of >80% (15), and this study focused on three of these counties. Likewise, the study countries have high rates of medicalized FGM, performed primarily by midwives, who make up between 71% to 93% of primary care providers in the three study countries (16) hence the selection of nurses and midwives as the target group for this intervention.

The purpose of this study was to test a two-level intervention package to enable ANC providers to deliver person-centered FGM counseling to their clients.[1] This intervention package was informed by a theory of change that promotes health workers to be effective behavioral change agents because of their credibility (17) and positionality to influence the opinions, attitudes, beliefs, motivations and behaviors of their clients (18). We hypothesized that if ANC providers gained the necessary knowledge and skills to provide person-centered counseling

For peer review only - http://bmjopen.bmj.com/site/about/guidelines.xhtml

(Level 2) and were given the opportunity to question their beliefs and attitudes together with an enabling environment (Level 1), they could positively influence the knowledge and attitudes of their clients to abandon the practice (Figure 1).

The level one intervention consisted of making available national policy directives on the role of health care providers in providing FGM prevention and care services, WHO's FGM guidelines and clinical handbook as well as information, education, and communication (IEC) materials. These materials were distributed without any capacity building to accompany their distribution. Level two consisted of an interactive training specifically targeting ANC providers to build their knowledge on FGM, enable them to question their FGM-related values and attitudes and build their skills on counseling for FGM prevention using person-centred communication (19), a component of person-centred care, which ensures that the perspectives and preferences of individuals, carers, families and communities are at the center of decisions and that they have the information and support needed to make decisions (20). ANC providers were trained to apply a series of structured steps in which they would: 'Assess' their client's views on FGM, address and challenge her 'Beliefs', encourage 'Change' and together with the client, 'Discuss and Decide' (ABCD).

METHODS

Study Design

This cluster randomized trial applied a type 2 hybrid, effectiveness-implementation design to test the effectiveness of the delivery of a phased intervention package (Levels 1 and 2) on knowledge, attitudes and practices among ANC health workers and their clients. The methodology, analysis plan and reporting conformed to the 2010 Consolidated Standards of Reporting Trial (CONSORT) checklist (21). Ethical approval for the master protocol was

For peer review only

obtained from the World Health Organization (WHO) Ethical Review Committee (ERC) (#P151/03/2014). Each study country submitted country-specific protocols to local institutional review boards. Ethical approval was obtained in Kenya from the Kenyatta National Hospital/University of Nairobi ERC (P805/09/2019) and the National Commission for Science, Technology, and Innovation (NACOSTI/P/20/5721); in Somalia from the Department of Planning, Policy and Strategic Information, Unit of Research (MOHD/DG: 2/11526/2019); and in Guinea from the *Comité National d'Ethique Pour la Recherche en Santé* (CNERS) (105/CNERS/19).

Participants

Within each study country, two or three sub-national units (regions/counties) were purposively selected according to the following eligibility criteria: (1) FGM prevalence >50% among females 15 - 49 years old; (2) more than 15 ANC clinics, seeing on average 30 new ANC clients per month and (3) accessibility in terms of security. The unit of randomization was the ANC clinic to avoid having ANC providers in the same clinic in different study arms, which could lead to contamination. In intervention sites, all providers on duty were pre-screened. To ensure participation and follow-up throughout the trial, between one and three ANC providers on duty were enrolled based on a six-month clinic rotation schedule provided by the clinic manager. Ten new clients exiting their first ANC consultation with a participating provider were recruited at each data collection point.

Individual study participants gave verbal informed consent. Data collectors collected data from the ANC providers and their clients in a private and confidential setting. While personally identifiable information was collected from ANC providers to facilitate tracking during the follow-up data collection time points, data were de-identified prior to analysis. No personally

For peer review only

identifiable information was collected from ANC clients who were unique at each time point. Participating ANC clients received the equivalent of 5 USD to compensate for their transport costs recognizing that participants consenting to participate might have changed their plans to accommodate the interviews. Given insecurity in carrying cash in Somalia, a mobile phone application was used to transfer the money to participants, an amendment to the original protocol, which was submitted to the ethical review committees.

Randomization and blinding

Based on Ministry of Health (MoH) facility administrative records of all public, primary care facilities (i.e., dispensaries and/or health centers) offering ANC services in the selected regions/counties, the average number of new ANC clients seen in November and December 2019 was compiled to create ordered listings of client loads at each of the sites by region/county. Clinics were matched into pairs based on client load so the two busiest would be randomized to different arms and so on. A uniform distribution was used for randomization using the uniform random number function in STATA 17 (StataCorp Inc., College Station, TX, USA). The clinic managers, providers and clients were blinded as to study arm allocation. Intervention clinics might have inferred they were in the intervention arm. However, both arms received the level one intervention at baseline so clinics in the control arm might have also assumed they were in the intervention group.

Procedures

Implementation of the study interventions and data collection occurred between August 2020 and September 2021 and was staggered by countries. In the intervention arm, data collection was undertaken at three time points, i.e., at baseline prior to implementing the level one intervention component; at month three, prior to implementing the level two intervention

For peer review only

component and at month six. In the control arm, data collection was done at two time points, i.e., at baseline and at month six. Study instruments included one for ANC clients, one for health workers and a health facility checklist completed by clinic managers. The intervention was pretested among health workers in Kenya in 2019. Instruments were pretested among ANC clients and providers from non-participating sites in all countries, and country teams provided feedback on the structure and appropriateness of each question prior to finalizing the instruments.

A web-interface electronic data capture system was developed on the Kobo toolbox core system architecture (Kobo Toolbox, Harvard Humanitarian Initiative, Boston, Massachusetts, USA). User accounts were password-protected, and data sent to the server was encrypted in transit using SHA256 with RSA encryption that met the data security requirements. Personally identifiable information was not collected, and all records were anonymized with unique study numbers. Study instruments for ANC clients were translated from English into ten languages by research team members in consultation with language experts (French, Somali, Swahili, Soussou, Poular, Malinké, Keiyo, Maasai, Marakwet and Tugen) while those for ANC providers and clinic managers were translated into two languages (French and Somali). No backtranslation was performed. Field data collectors and their supervisors spoke the languages in which the questionnaires were administered. Data collection teams participated in a standardized training with WHO/HRP and the research institutions in each country. The level two intervention was implemented by master trainers in each country who had been trained remotely over a three-day period following the WHO PCC for FGM prevention facilitator's manual.

Outcomes

For peer review only

The primary study outcomes included the delivery of the “ABCD” approach by ANC providers measured by responses from client and provider instruments developed for this study, using validated instruments where possible, including four constructs of the operational definition of person-centered communication (22). The secondary self-efficacy outcome was assessed based on a score calculated from a validated tool for measuring general self-efficacy (23), while knowledge, attitudes, and practice (KAP) on FGM prevention and care were measured using an unvalidated KAP questionnaire similar to one used in formative research in Guinea. Questions for the health facility preparedness composite score were developed for this study and assessed availability and use of FGM prevention and care resources. (See supplementary materials).

Statistical analysis

To have sufficient power (80%) to detect a difference (significance level 5%) between intervention and control arms on the primary study outcome of delivery of the PCC intervention for FGM prevention, 180 ANC clinics, equally divided across the three study countries were recruited and randomized with 1800 new ANC clients (10 per clinic) recruited at baseline and 1800 at six-month follow-up. While similar interventions have resulted in 20% difference between groups (24), a 10% difference was applied to ensure sufficient power to detect a difference if the intervention was less effective than expected and considering the minimal levels of clinical efficacy for such an intervention to be practical. This sample size also allowed for a 10% non-response and/or loss to follow-up rate and accounted for a clustering effect (ICC=0.20) on the clinic level. A relatively high level of clustering was assumed in the sample size calculations to not underestimated the needed sample size. Region/county level was not included in the multilevel model due to the low number of included regions/counties per country (Kenya

For peer review only

3, Guinea 2, Somalia 3) and since it would not allow for an accurate estimate of the variance between clusters.

Data were analyzed using STATA 17 software following a per-protocol approach. Data from ANC providers and their clients were analyzed if the clinic had at least one provider with follow up data at all study time points, and in the intervention arm, if the ANC provider present had undergone training on PCC for FGM prevention at month three. Clinics where providers were lost to follow-up were not included in the final analyses. All facility checklists and ANC client exit interviews were conducted as intended except at sites not accessible due to security issues or closed or converted for care of COVID-19 patients during the pandemic.

The study was designed to pre-screen providers and include in the analytic sample only those who would be available at 3 and 6 months at the clinic. Therefore, an intention-to-treat approach was not feasible. Key characteristics of the participating facilities, providers and clients were summarized. Continuous variables are presented using mean values, and standard deviation (SD) while categorical variables were summarized as counts (N) with percentages (%). Differences in proportions were analysed for dichotomous outcomes using Fischer's exact test. For outcomes measured as summary scores, comparisons of mean scores are presented across study arms using t-tests.

Initial analyses showed that the clustering was negligible, probably due to having many small-sized clusters with only 10 patients per ANC clinic. To avoid convergence issues in the statistical analyses, we chose to not use multilevel mixed-effect modelling in the final analyses but ordinary regression models. Estimates were compared to the corresponding estimates from multilevel regression models and were found to be almost equal. To compare intervention and

For peer review only

control arms, logistic regression models were fitted. Linearity was assessed for the continuous covariates included in the regression models.

At month six, a comparison of study outcomes between the intervention and control arms was used to determine the combined effect of both levels of the intervention package. The multiple variable logistic regression analyses for ANC provider outcomes were adjusted for their sex, years of service, FGM status, FGM-related training, any specific training on communication/counseling and PCC, and whether the provider had conducted FGM in the past. Analyses related to ANC client outcomes were adjusted for their age, educational level, FGM status and exposure to level one IEC materials. These variables were determined a priori based on previously published literature. Unadjusted analyses are presented for outcomes that relate to composite measures based on ANC provider and client responses.

In-country data managers monitored data quality. Periodic data audits were conducted by the WHO/HRP Quantitative Assessment and Data Management team to identify any data collection gaps and data discrepancies requiring follow up by in-country teams. Weekly data monitoring meetings were held between the in-country research teams and WHO/HRP staff during data collection periods to identify, document and resolve any data discrepancies. These were virtual due to the COVID-19 pandemic. Given that there was no prospective follow-up of clients, a Data Safety and Monitoring Board was not established. Instead, local research teams documented and reported any unintended harms and/or protocol deviations to the WHO/HRP study coordination team.

Patient and public involvement statement

Health care providers and members of communities where the practice of FGM is prevalent in the study countries were actively involved in the design and implementation of this

For peer review only

study intervention. This included through formative research conducted in Guinea, during which interviews and focus group discussions were conducted with ANC clients, male community members, health workers, health systems managers and other key stakeholders. Health workers are considered integral members of FGM practicing communities who understand local community beliefs and norms with the potential to be change agents. The formative research also found that health workers can be supported by incorporating FGM content within their pre- and in-service trainings, ensuring accountability to legal and policy standards and recognition and promotion of FGM abandonment within the health sector as part of a multi-sectoral approach. These findings informed the development of the PCC training, which was subsequently pilot tested among ANC providers in Kenya before being rolled out as part of the multi-country study.

In Kenya, community health volunteers in the study counties talked about the study during their community sensitization sessions and invited pregnant women to attend routine ANC sessions where they could be approached for participation in the study. Prior to providing informed consent, health workers and pregnant women received information about the study, including the time commitment, any risks involved in their participation, and the voluntary nature of their participation.

Study dissemination meetings were conducted in Kenya and Guinea with the MoH and other stakeholders including representatives of health care providers and community members where the study was implemented. In these meetings, the in-country research partners have led the development of policy briefs identifying country-specific results relevant for local research needs, policy development and practice.

Role of the funders

For peer review only

Apart from WHO/HRP, the study funders had no role in study design or implementation. WHO/HRP, in collaboration with in-country research teams, developed the study protocol, provided data management and analytic support, and contributed to interpretation and manuscript writing. An author reflexity checklist has been included (Annex 1). This trial was registered: PACTR201906696419769 (June 3, 2019).

RESULTS

Recruitment and retention

Between August 2020 and September 2021, a total of 180 ANC clinics (i.e., 60 clinics per study country) were enrolled and randomized to intervention and control arms. There was some natural staggering of the start and subsequent data collection dates due to factors such as weather, COVID-19, Ramadan, and national elections. Data collection periods ranged from three to six weeks in each country at each time point. The time elapsed between the end of one data collection period to the beginning of the next data collection period ranged from three to five months.

In the intervention arm, 230 providers and 900 clients (i.e.., 10 per clinic) were interviewed. Based on a review of clinic rotation schedule to ensure participation of at least one provider from each study clinic throughout the trial, 133 providers were enrolled in the trial. In the control arm, 240 providers and 900 clients were interviewed. (*Figure 2*). At month three, data were collected at 98% (n=88) of the intervention clinics as two clinics in Kenya were inaccessible due to insecurity. One hundred and thirty (98%) ANC providers (at least one from each site) and 880 first visit ANC clients completed the month three questionnaires prior to implementing the Level 2 PCC intervention. No data collection was conducted at the control sites. At month six, 91% (n=163) of ANC clinics (81, intervention and 82, control) had at least

For peer review only

one ANC provider (intervention n=110 and control n=122) on duty who was previously enrolled in the study. The client questionnaire was applied to 819 and 810 first visit ANC clients, respectively in the intervention and control sites.

Characteristics of study sites and participants

The 163 ANC clinics with complete follow-up date at the end of the study had a mean of four ANC providers (standard deviation, SD: 3) and served on average 155 new ANC clients per month (SD: 127) with a mean catchment population of 36,754 people (SD: 126,082). In 55% (n=89) of clinics, the clinic manager reported that there were no activities promoting FGM prevention in the facilities' catchment area (*Table 1*). These characteristics were not different from the 17 ANC clinics that were enrolled at baseline but that subsequently were lost to follow-up.

Of the 232 ANC providers who contributed data for analysis at month six, 83% (n=193) were female and their mean age was 36 years (SD: 10 years). They had an average of eight years professional experience (SD: 7 years) and 68% (n=158) had studied up to Diploma level (generally 3 years post-secondary education) with 90% (n=208) identifying as either midwives, nurses, or nurse-midwives. Health cadres were defined by national licensing requirements in each country. Among these providers, at baseline, 63% (n=146) reported that they had not previously received formal clinical training on FGM prevention and care (*Table 1*). Almost two-thirds (64%, n=14) reported that they had received training on communication/counselling while half (51%, n=118) had received training on person-centered care. Further, 54% (n=126) of female providers reported that they had undergone FGM while overall, 94% (n=217) of providers reported that they had never performed FGM. These characteristics were not different when compared to the ANC providers who were on duty in the 180 ANC clinics enrolled at

For peer review only

baseline. The mean age of the 1,800 clients exiting their first ANC visits at baseline was 26 years (SD: 6 years), 47% (n=846) reported not having received any education, and 73% (n=1,320) reported that they had undergone FGM. These characteristics were similar to the 880 and 1,630 first visit ANC clients interviewed at month three (intervention arm only) and month six, respectively (*Table 2*).

To evaluate potential bias from differential selection of providers receiving the intervention, we assessed differences in baseline characteristics between the 133 ANC providers from intervention facilities who were screened at baseline and received PCC training at month three (i.e., included in the analysis sample) versus the 97 who were screened and did not receive the intervention (i.e., excluded from analysis sample). These groups were similar in terms of sex, educational level, professional cadre, as well as whether they had undergone or recently performed FGM, however, included providers tended to be slightly younger (by two years on average) and less likely to be of Muslim religion, although the question on religion was not administered for the Somalia sample (all were assumed to be Muslim).

Health facility preparedness

At month six, ANC clinics in the intervention arm had a significantly higher mean score for health facility preparedness compared to the control arm (3.4 (95% CI: 3.2-3.6) vs. 2.6 (95% CI: 2.4-2.9; p<0.001)).

Utilization of level one intervention components

A higher proportion of ANC providers in the intervention arm reported having utilized the level one intervention package components compared to those in the control arm (91% vs. 56%, p<0.001). In multiple variable analyses, ANC providers in the intervention arm had nine

For peer review only

times the odds of having utilized the level one intervention package components as compared to those in the control arm (AOR: 9.3, 95% CI: 4.2-20.8; P<0.001).

Delivery of FGM care and ABCD components

At month six, based on a cumulative score to specific questions on correct prevention and care service provision, including on the ABCD elements, a higher proportion of ANC providers in the intervention arm provided FGM prevention and care services correctly as compared to those in the control arm (50% vs. 34%, p=0.03). Additionally, a higher proportion of ANC providers in the intervention arm asked their clients if they had undergone FGM (78% vs. 31%, p<0.001), asked their clients' personal beliefs regarding FGM (76% vs. 27%, p<0.001) and discussed with their clients why (77% vs. 30%, p<0.001) and how (73% vs. 29%, p<0.001) FGM could be prevented. Furthermore, a higher proportion of ANC clients in the intervention compared to the control arms reported that they were satisfied with how FGM-related prevention and care services had been addressed during the visit (84% vs. 44%, p<0.001).

ANC providers' confidence, self-efficacy, and communication skills

A higher proportion of ANC providers in the intervention arm reported being confident in their knowledge to provide FGM prevention and care services compared to those in the control arm (98% vs. 89%, p=0.005). In multiple variable analysis, ANC providers in the intervention arm had more than six times the odds of reporting being confident in their knowledge to provide FGM prevention and care services compared to those in the control arm (AOR: 6.3, 95% CI: 1.4-28.9; p=0.02). Self-efficacy was generally high (scores 7.4 – 7.8 out of 8) with no significant difference in high scores between study arms (85% vs. 82%, p=0.36 and OR: 0.8, 95% CI: 0.4-1.6); p= 0.50).

ANC providers' knowledge and attitudes

For peer review only

The mean scores for FGM-related knowledge were higher among ANC providers in the intervention arm compared to the control arm (2.5, 95% CI: 2.2-2.8 vs. 1.9, 95% CI: 1.7-2.2; p=0.005). Overall scores were generally low, ranging from 1.6 to 2.5 out of 6. Providers had similarly unsupportive attitudes towards FGM in both groups (73% vs. 72%, p=0.54). and similar levels of support for FGM and/or medicalized FGM with most providers reporting that they did not support FGM and/or medicalized FGM at any time point (96% - 99%).

ANC clients' support for FGM

Compared to those in the control arm, a higher proportion of ANC clients in the intervention arm reported being less supportive of FGM after their month six clinic visit (52% vs. 29%, p<0.001). In multiple variable analysis, ANC clients in the intervention arm had nearly twice the odds of reporting being strongly opposed to FGM (AOR: 1.7, 95% CI: 1.4-2.1; p<0.001). When asked about their support for FGM after the ANC visit compared to before, clients in the intervention arm had more than twice the odds of being less supportive of FGM compared to those in the control arm (OR: 2.4, 95% CI: 2.0-3.0; p<0.001). ANC clients in the intervention clinics had lower odds of intending to have their daughters undergo FGM (OR: 0.4, 95% CI: 0.3-0.5; p<0.001) or of wanting a health care provider to perform FGM (OR: 0.4, 95% CI: 0.3-0.5; p<0.001) and higher odds of reporting that they wished to be active in FGM prevention (OR: 2.2, 95% CI: 1.8-2.9, p<0.001).

DISCUSSION

The results of this cluster randomized trial show that an intervention to strengthen health facility preparedness while building skills of ANC providers to communicate using a person-centred counselling technique on FGM prevention was effective. ANC providers exposed to the intervention had increased confidence in their communication, improved FGM-related

For peer review only

knowledge, and effective delivery of FGM prevention and care services. Additionally, ANC clients who had received care from these providers were less supportive of FGM and had reduced intentions to perform FGM on their daughters. This study provides evidence of a practical intervention to engage health care providers in FGM abandonment efforts whilst also providing quality care to FGM survivors. This study provides evidence of how to effectively build the capacity of health care providers at primary care to address FGM (25), an area identified as a critical gap during the formative research.

The PCC training modules not only strengthened ANC providers' knowledge and skills on FGM prevention and care but also addressed their beliefs and attitudes, which are key drivers of FGM (26). We did not find notable changes in knowledge and attitudes among ANC providers. Exposure to the intervention package also did not improve ANC providers' self-efficacy towards FGM prevention and care. This may be related to the lack of support for FGM and/or its medicalization and high self-efficacy among nearly all providers at baseline in both study arms, a finding that was also noted in formative research conducted in Guinea (27,28). In the formative phase, while the vast majority of health workers were opposed to the practice, 38% also felt that FGM limited promiscuity and 7% believed that it was a good practice, showing ambivalence and complexity in attitudes about FGM among health providers. Other studies have found that some providers support the perpetuation of the practice and even planned to have their own daughters undergo FGM or to perform it on their clients (29).

The findings in this study underscore the importance of addressing values and attitudes of both providers and clients as a means of achieving positive behavioral change. Changes observed among ANC providers were sustained across the study duration and ultimately, and importantly, resulted in reported changes in attitudes and intentions of their clients. However, this study

For peer review only

design did not allow us to determine whether the attitudinal changes observed among ANC clients were sustained after their clinic visit or translated into positive change in FGM prevention.

The application of these study results into programming will need to consider several factors. Firstly, the study sites were primary care facilities located in high FGM prevalence settings. The results of this intervention may not be generalizable to settings where FGM is less prevalent or to settings other than primary care. Secondly, first ANC visits are not typical of other health visits since the consultation is generally longer with a greater focus on health promotion messaging. While this is an ideal setting for implementing such an intervention, its application to other health settings and among other population groups is not known. During scale up, if the PCC approach is applied among clients seeking other sexual and reproductive health services or parents bringing their children to child immunization and wellness visits, it will be important to consider time requirements for the delivery of the 'ABCD' steps, especially in high volume clinic settings.

Thirdly, while the study found a positive impact of the PCC training on health care providers' delivery of person-centred FGM prevention counselling, the continuity and quality of FGM prevention counselling in the long-term is not known. Specifically, it will be important to assess subsequently whether providers will continue to provide prevention counselling on an ongoing basis, whether they will share their learnings with family and community members and whether clients will follow through with their intentions to not have their daughters undergo FGM. It may be important to include a supervisory mentorship component to ensure implementation of this intervention (30) in order to strengthen PCC communication practice and quality.

For peer review only

Limitations

The implementation of this multi-country study was not without challenges and limitations. First, initiation of field data collection activities was delayed by the global COVID-19 pandemic and required some modification to trainings of the data collection teams, the master trainers and the ANC providers receiving the PCC intervention. This may have impacted the overall effectiveness of the intervention.

Second, to attempt to ensure participation of at least one provider at each site, all providers were pre-screened at baseline and clinic rotation schedules determined enrollment into the study. Selection bias might have been introduced through this process. The exploratory analysis to assess for selection and attrition bias from the pre-screen step and per protocol analysis was limited, and it is possible that differences in other unmeasured factors related to the clinics and providers might have biased the results. Findings from a process evaluation conducted as part of this study will provide additional insights on the feasibility, acceptability, appropriateness, and fidelity of the intervention implementation in these contextual settings to inform further implementation and scale up.

Third, we did not perform adjustment for multiple testing in our analysis given that the different tests are interpreted and presented separately, therefore the overall type one error rate could be higher than the individual test level of 0.05.

Finally, we acknowledge that there are many factors that could impact FGM-related decision-making: a positive and impactful interaction with a respected health care provider might not be sufficient to lead to actual changes in individual behavior and community norms. However, the study design enabled us to compare similar sites to identify the relative effect of

For peer review only

this new approach since both intervention and control sites would be exposed to similar factors, and clients at these sites would face similar complexities in decision-making.

Conclusion

In conclusion, this study highlights the importance of addressing the values and beliefs of health care providers working at primary care level, who are subject to social norms around FGM that may conflict with medical ethics and national laws and policies as an intermediary step in preventing FGM. Empowering these health care providers with communication skills and engaging them as opinion leaders can be impactful in changing their clients' attitudes towards FGM. In conjunction with FGM prevention activities in other sectors, this intervention can contribute to positive change if brought to scale.

For peer review only

DECLARATIONS

Contributors

WA and CP conceptualized the study and prepared the protocol in collaboration with VM, KS, PN, TE, MDB, AMS, AD and MAA. MDB, AMS, AOS, PN, TE, JMK, AD and MAA provided oversight over study implementation while AD, JK and SA monitored data quality in countries and KN and SST monitored data quality across countries. VM prepared the first draft of the manuscript with input from WA and CP, the responsible officer of the study at WHO/HRP. MP developed the statistical analysis plan and conducted data analysis. KS coordinated the development of the PCC for FGM prevention training. KS, PN, TE, JMK, JK, MDB, AMS, AOS, AD, AD, SA, and MAA contributed to and reviewed the manuscript for proper intellectual content. All authors read and approved the final draft of this manuscript.

Declaration of interests

The authors declare that they have no competing interests.

Data sharing

De-identified dataset will be retained in the WHO HRP electronic archival system. Any use of the de-identified analytic dataset for secondary research purposes will be governed by the WHO data use regulation. Request for data dictionary and for dataset may be sent to pallittoc@who.int

Acknowledgements

The authors would like to acknowledge the funders, the WHO country office colleagues, Dr Bernadette Dramou, Dr Cécé-Vieux Kolie, Dr Joyce Lavussa, Ms Matilda Cherono, Ms Asia Ahmed Osman in the study countries as well as MoH and national FGM stakeholders in the development of the research protocol, implementation of study interventions and field data collection. We also thank Professor Joanna Schellenberg of the London School of Hygiene and Tropical Medicine and Dr Christina Atchison of Imperial College London for their input in

For peer review only

conceptualizing the study as well as Dr Leyla Hussein for supporting pilot testing of the PCC intervention. This work received funding from the Governments of Norway and the United Kingdom of Great Britain and Northern Ireland as well as the UNDP-UNFPA-UNICEF-WHO-World Bank Special Programme of Research, Development and Research Training in Human Reproduction (HRP), a cosponsored programme executed by the World Health Organization (WHO).

Disclaimer

The named authors alone are responsible for the views expressed in this publication and do not necessarily represent the decisions or the policies of the UNDP-UNFPA-UNICEF-WHO-World Bank Special Programme of Research, Development and Research Training in Human Reproduction (HRP) or the World Health Organization (WHO).

For peer review only

REFERENCES

1. United Nations General Assembly, Intensifying global efforts for the elimination of female genital mutilations : resolution/ / adopted by the United Nations General Assembly, 5 March 2013, A/RES/67/146, available at: https://www.refworld.org/docid/51e67bc64.html [accessed 4 May 2022]
2. World Health Assembly, WHA resolution 61.16. 2008. United Nations: New York. 2008. Available at https://apps.who.int/gb/ebwha/pdf_files/WHA61-REC1/A61_REC1-en.pdf , New York. 2008.
3. Eliminating female genital mutilation: an interagency statement-OHCHR, UNAIDS, UNDP, UNECA, UNESCO, UNFPA, UNHCR, UNICEF, UNIFEM, WHO. WHO: Geneva.World Health Organization, 2008.
4. World Health Organization. The World Health Report: 2000 :health systems : improving performance [Internet]. Geneva: World Health Organization; 2000. Available from: https://apps.who.int/iris/handle/10665/42281
5. Pallitto CC, Ahmed W. The role of the health sector in contributing to the abandonment of female genital mutilation. Med (N Y) [Internet]. 2021 May 14 [cited 2023 May 15];2(5):485–9. Available from: https://pubmed.ncbi.nlm.nih.gov/35590230/
6. Kimani S, Esho T, Kimani V, Muniu S, Kamau J, Kigondu C, et al. Female Genital Mutilation/Cutting: Innovative Training Approach for Nurse-Midwives in High Prevalent Settings. Obstet Gynecol Int [Internet]. 2018 [cited 2023 May 15];2018. Available from: https://pubmed.ncbi.nlm.nih.gov/29736171/
7. Johansen REB, Diop NJ, Laverack G, Leye E. What works and what does not: a discussion of popular approaches for the abandonment of female genital mutilation. Obstet Gynecol Int [Internet]. 2013 [cited 2023 May 15];2013:1–10. Available from: https://pubmed.ncbi.nlm.nih.gov/23737795/
8. Njue C, Askew I. Medicalization of female genital cutting among the Abagusii in Nyanza Province, Kenya. Reprod Health [Internet]. 2004 Jan 1 [cited 2023 May 15]; Available from: https://knowledgecommons.popcouncil.org/departments_sbsr-rh/32
9. Shell-Duncan B, Gathara D, Moore Z. Female genital mutilation/cutting in Kenya: Is change taking place? Descriptive statistics from four waves of Demographic and Health Surveys. Reprod Health [Internet]. 2017 Jan 1 [cited 2023 May 15]; Available from: https://knowledgecommons.popcouncil.org/departments_sbsr-rh/596
10. Umar AS, Oche OM. Medicalization of female genital mutilation among professional health care workers in a referral hospital, north-western Nigeria. J Reprod Biol Health. 2014;2(1):2.
11. Doucet MH, Pallitto C, Groleau D. Understanding the motivations of health-care providers in performing female genital mutilation: an integrative review of the literature. Reprod Health [Internet]. 2017 Mar 23 [cited 2023 May 15];14(1):46. Available from: https://pubmed.ncbi.nlm.nih.gov/28335771/
12. World Health Organization. Female Genital Mutilation: A Resource Kitkit for the Health Sector. WHO: Geneva, 2021, available at https://srhr.org/fgmresources/https://srhr.org/fgmresources/ - Google Search [Internet]. [cited 2023 May 15].
13. World Health Organization. Reproductive Health and Research. WHO guidelines on the management of health complications from female genital mutilation. :47.

For peer review only

14. Ahmed W, Mochache V, Stein K, Ndavi P, Esho T, Balde MD, et al. A hybrid, effectiveness-implementation research study protocol targeting antenatal care providers to provide female genital mutilation prevention and care services in Guinea, Kenya and Somalia. BMC Health Serv Res [Internet]. 2021 Dec 1 [cited 2023 May 15];21(1). Available from: https://pubmed.ncbi.nlm.nih.gov/33522926/
15. National Bureau of Statistics. Republic of Kenya. Kenya Demographic and Health Survey 2014. 2015 [cited 2023 May 15]; Available from: www.DHSprogram.com.
16. NHWA Web portal [Internet]. [cited 2023 May 15]. Available from: https://apps.who.int/nhwaportal/Home/Welcome?ReturnUrl=%2Fnhwaportal%2FHome%2FIndex
17. Boone Tim, Reilly Anthony J., Sashkin M. SOCIAL LEARNING THEORY Albert Bandura Englewood Cliffs, N.J.: Prentice-Hall, 1977. 247 pp., paperbound. Group & Organization Studies [Internet]. 1977 Sep 1;2(3):384–5. Available from: https://doi.org/10.1177/105960117700200317
18. Valente TW, Pumpuang P. Identifying opinion leaders to promote behavior change. Health Educ Behav [Internet]. 2007 Dec [cited 2023 May 15];34(6):881–96. Available from: https://pubmed.ncbi.nlm.nih.gov/17602096/
19. Person-centred communication for female genital mutilation prevention: facilitator's manual. WHO World Health Organization: Geneva, 2022
20. World Health Organization. WHO global strategy on people-centred and integrated health services: interim report [Internet]. Geneva: World Health Organization; 2015. Available from: https://apps.who.int/iris/handle/10665/155002
21. Schulz KF, Altman DG, Moher D. CONSORT 2010 statement: updated guidelines for reporting parallel group randomised trials. BMJ [Internet]. 2010 Mar 27 [cited 2023 May 15];340(7748):698–702. Available from: https://pubmed.ncbi.nlm.nih.gov/20332509/
22. Epstein RM, Franks P, Fiscella K, Shields CG, Meldrum SC, Kravitz RL, et al. Measuring patient-centered communication in patient-physician consultations: theoretical and practical issues. Soc Sci Med [Internet]. 2005 Oct [cited 2023 May 15];61(7):1516–28. Available from: https://pubmed.ncbi.nlm.nih.gov/16005784/
23. Chen G, Gully SM, Eden D. Validation of a New General Self-Efficacy Scale. Organ Res Methods [Internet]. 2001 Jan 1;4(1):62–83. Available from: https://doi.org/10.1177/109442810141004
24. Keeley RD, Burke BL, Brody D, Dimidjian S, Engel M, Emsermann C, et al. Training to use motivational interviewing techniques for depression: a cluster randomized trial. J Am Board Fam Med [Internet]. 2014 Sep 1 [cited 2023 May 15];27(5):621–36. Available from: https://pubmed.ncbi.nlm.nih.gov/25201932/
25. Balfour J, Abdulcadir J, Say L, Hindin MJ. Interventions for healthcare providers to improve treatment and prevention of female genital mutilation: a systematic review. BMC Health Serv Res [Internet]. 2016 Aug 19 [cited 2023 May 15];16(1). Available from: https://pubmed.ncbi.nlm.nih.gov/27542732/
26. Kimani S, Okondo C, Muteshi-Strachan J, Guyo J. Quality of services offered to women with female genital mutilation across health facilities in a Kenyan County. BMC Health Serv Res [Internet]. 2022 Dec 1 [cited 2023 May 15];22(1). Available from: https://pubmed.ncbi.nlm.nih.gov/35525954/
27. Balde MD, O'Neill S, Sall AO, Balde MB, Soumah AM, Diallo BA, et al. Attitudes of health care providers regarding female genital mutilation and its medicalization in Guinea.

For peer review only

PLoS One [Internet]. 2021 May 1 [cited 2023 May 15];16(5). Available from: https://pubmed.ncbi.nlm.nih.gov/33983949/

28. Balde MD, Soumah AM, Diallo A, Sall AO, Mochache V, Ahmed W, et al. Involving the health sector in the prevention and care of female genital mutilation: results from formative research in Guinea. Reprod Health. 2022 Dec 1;19(1).

29. Kaplan A, Hechavarría S, Bernal M, Bonhoure I. Knowledge, attitudes and practices of female genital mutilation/cutting among health care professionals in The Gambia: a multiethnic study. BMC Public Health [Internet]. 2013 [cited 2023 May 15];13(1). Available from: https://pubmed.ncbi.nlm.nih.gov/24040762/

30. Pantoja T, Opiyo N, Lewin S, Paulsen E, Ciapponi A, Wiysonge CS, et al. Implementation strategies for health systems in low-income countries: an overview of systematic reviews. Cochrane Database Syst Rev [Internet]. 2017 Sep 12 [cited 2023 May 15];9(9). Available from: https://pubmed.ncbi.nlm.nih.gov/28895659/

For peer review only

Table 1: Characteristics of ANC clinics and providers included in month six analyses

**ANC Clinics**			
**Characteristics**	**Overall (n=163*)**	**Intervention (n=82)**	**Control (n=81)**
Number of ANC providers	Mean 4 (SD: 3) Median 3 (1-14, IQR 3)	Mean 4 (SD: 2) Median 3 (1-11, IQR 3)	Mean 4 (SD: 3) Median 3 (1-14, IQR 4)
Average number of ANC clients/month	Mean 150 (SD: 127) Median 118 (3-664, IQR 141)	Mean 148 (SD: 121) Median 117 (3-500, IQR 143)	Mean 152 (SD: 133) Median 120 (3-664, IQR 140)
MoH supervisory visits in the past year	Mean 4 (SD: 3) Median 3 (0-18, IQR 2)	Mean 4 (SD: 3) Median 4 (1-18, IQR 1)	Mean 4 (SD: 3) Median 3 (0-12, IQR 2
Size of catchment population served	Mean 36,754 (SD: 126,082) Median 15,972 (1,000-1,458,000, IQR 24,332)	Mean 23,649 (SD: 35,873) Median 16,022 (1,000-290,000, IQR 22,361	Mean 50,020 (SD: 174,739) Median 15,551 (1,000-1,458,000, IQR 25,544
Presence of anti-FGM activities in the catchment area			
Yes	74 (45%)	43 (52%)	31 (38%)
No	89 (55%)	39 (48%)	50 (62%)
Presence of pro-FGM activities in the catchment area			
Yes	21 (13%)	12 (15%)	9 (11%)
No	140 (86%)	68 (83%)	72 (89%)
Don't Know	2 (1%)	2 (2%)	0 (0%)
**ANC Providers**			
	**Overall**	**Intervention**	**Control**

For peer review only

**Characteristics**	**(n=232)**	**(n= 115)**	**(n=117)**
Age	Mean 36 (SD: 10) Median 34 (20-65, IQR 15)	Mean 35 (SD: 10) Median 33 (20-59, IQR 14)	Mean 37 (SD:11) Median 35 (20-65, IQR 16)
Years of professional experience	Mean 8 (SD: 7) Median 6 (1-39, IQR 7)	Mean 8 (SD:7) Median 6 (1-30, IQR 8)	Mean 8 (SD:7) Median 6 (1-39, IQR 7)
Sex			
Female	193 (83%)	95 (83%)	98 (84%)
Highest educational level			
Certificate	21 (5%)	12 (10%)	9 (8%)
Diploma	158 (68%)	72 (63%)	86 (74%)
Bachelors	44 (19%)	27 (24%)	17 (15%)
Masters & above	1 (0.4%)	0 (0%)	1 (1%)
Other[#]	8 (3%)	4 (3%)	4 (3%)
Current professional role/title			
Midwife	103 (44%)	53 (46%)	50 (43%)
Nurse	51 (22%)	25 (22%)	26 (22%)
Nurse-Midwife	54 (23%)	27 (24%)	27 (23%)
Other	24 (10%)	10 (9%)	14 (12%)
Received formal training on FGM during clinical training			
Yes	85 (37%)	44 (38%)	41 (35%)
No	146 (63%)	71 (62%)	75 (64%)
Don't Know	1 (0.4%)	0 (0%)	1 (1%)

For peer review only

Timing of clinical training on FGM			
Pre-service	33 (14%)	18 (16%)	15 (13%)
In-service	45 (19%)	22 (19%)	23 (20%)
Both pre- and in-service	7 (3%)	4 (4%)	3 (3%)
Received formal training on communication/counselling			
Yes	149 (64%)	76 (66%)	73 (62%)
No	83 (36%)	39 (34%)	44 (38%)
Received formal training on person-centered care			
Yes	118 (51%)	58 (50%)	60 (51%)
No	113 (56%)	56 (49%)	57 (49%)
Don't know	1 (0.4%)	1 (1%)	0 (0%)
Undergone FGM			
Yes	126 (54%)	65 (57%)	61 (52%)
No	63 (27%)	27 (24%)	36 (31%)
Don't know	2 (1%)	2 (2%)	0 (0%)
Refused to answer	2 (1%)	1 (1%)	1 (1%)
Conducted FGM			
Yes	15 (7%)	9 (8%)	6 (5%)
Conducted FGM on a girl <18 years			
Yes	14 (6%)	8 (7%)	6 (5%)

* Total of 17 ANC clinics not included: 16 clinics were excluded (7 intervention and 9 control) due to loss-to-follow up (LTFU) of ANC provider i.e., the clinics did not have at least one ANC provider present across all study time points while one ANC clinic in

Kenya was never visited at subsequent time points due to issues with insecurity. An ANC provider from one of the clinics in Kenya that had been inaccessible due to insecurity attended the PCC training and was subsequently interviewed.

Table 2: Characteristics of ANC clients interviewed at each time point

**Characteristics**	**ANC clients interviewed at Baseline**			**ANC clients interviewed at Month 3**	**ANC clients interviewed at Month 6**		
	**Overall (n=1800)**	**Intervention (n=900)**	**Control (n=900)**	**Intervention only (n=880)**	**Overall (n=1759)**	**Intervention (n=879)**	**Control (n=880)**
Age	Mean 26 (SD: 6) Median 25 (15-45, IQR 10)	Mean 25 (SD: 6) Median 25 (15-45, IQR 10)	Mean 26 (SD: 6) Median 25 (15-45, IQR 10)	Mean 26 (SD 6) Median 25 (15-45, IQR 10)	Mean 26 (SD: 6) Median 25 (15-45, IQR 9)	Mean 26 (SD: 6) Median 25 (15-45, IQR 9)	Mean 26 (SD: 6) Median 25 (15-45, IQR 10)
Highest educational level							
None	840 (47%)	407 (45%)	433 (48%)	439 (50%)	806 (46%)	384 (44%)	422 (47%)
Primary	484 (27%)	231 (26%)	253 (28%)	239 (27%)	553 (31%)	278 (32%)	275 (31%)
Secondary	331 (18%)	171 (19%)	160 (18%)	157 (18%)	306 (17%)	160 (18%)	146 (16%)
University	95 (5%)	61 (7%)	34 (4%)	25 (3%)	67 (4%)	34 (4%)	33 (4%)
Other[#]	50 (3%)	30 (3%)	20 (25)	20 (2%)	37 (2%)	23 (3%)	14 (2%)
Have you undergone FGM?							
Yes	1320 (73%)	677 (75%)	643 (71%)	645 (73%)	1321 (75%)	655 (75%)	666 (75%)
No	452 (25%)	209 (23%)	243 (27%)	224 (25%)	420 (24%)	206 (23%)	214 (24%)
Don't know	12 (1%)	10 (1%)	2 (0.2%)	5 (1%)	21 (1%)	13 (2%)	8 (1%)
Refused to answer	16 (1%)	4 (0.4%)	12 (1%)	6 (1%)	7 (0.4%)	5 (1%)	2 (0.2%)

For peer review only - http://bmjopen.bmj.com/site/about/guidelines.xhtml

Table 3: Results of study outcomes

	Month 6 (Intervention vs control)	P-value	Adjusted OR* (95% CI)	P-value
**ANC facility preparedness (Intervention n=82, Control n=81)**				
Clinics with ALL correct answers for facility preparedness	56 (69%) vs. 22 (27%)	<0.001		
Facility preparedness mean score (0 – 4)	3.4 (3.2-3.6) vs. 2.6 (2.4-2.9)	<0.001		
**ANC provider outcomes** (Intervention n=115, Control n=117)**				
Using level 1 intervention package	96 (91%) vs. 65 (56%)	<0.001	9.3 (4.2-20.8)	<0.001
Providing appropriate FGM-related prevention and care services	52 (50%) vs. 40 (34%)	0.03		
With correct FGM-related knowledge responses	8 (8%) vs. 1 (2%)	0.09		
ANC providers mean knowledge score (0 – 6)	2.5 (2.2-2.8) vs. 1.9 (1.7-2.2)	0.005		
With appropriate interpersonal communication skills	74 (70%) vs. 68 (58%)	0.04	1.7 (1.0-3.0)	0.06
ANC provider communication skills mean score (0 – 5)	4.7 (4.5-4.8) vs. 4.4 (4.2-4.5)	0.003		
With high self-efficacy	86 (82%) vs. 99 (85%)	0.36	0.8 (0.4-1.6)	0.50
ANC providers self-efficacy mean score (0 – 8)	7.6 (7.3-7.8) vs. 7.6 (7.4-7.8)	0.94		
Reporting less supportive attitudes towards FGM	76 (72%) vs. 85 (73%)	0.54	1.0 (0.5-1.8)	0.90
ANC provider FGM attitude mean score (0 – 8) *	7.6 (7.5-7.8) vs. 7.5 (7.4-7.7)	0.57		
With high confidence scores	103 (98%) vs. 104 (89%)	0.005	6.3 (1.4-28.9)	0.02
Not supportive of FGM	100 (96%) vs. 114	0.44	0.8 (0.2-	0.73

For peer review only - http://bmjopen.bmj.com/site/about/guidelines.xhtml

	(97%)		3.7)	
Not supportive of medicalized FGM	104 (99%) vs. 116 (99%)	0.72	1.1 (0.1-22.1)	0.94
**ANC provider implementation of PCC for FGM prevention approach (as reported by clients) (Intervention n=819, Control n=810)**				
Provider asked client if they have undergone FGM	694 (78%) vs. 245 (31%)	<0.001		
Provider asked client about their (client's) personal beliefs regarding FGM	616 (76%) vs. 217 (27%)	<0.001		
Provider discussed with client why FGM should be prevented	629 (77%) vs. 244 (30%)	<0.001		
Provider discussed with client how FGM could be prevented	592 (73%) vs. 232 (29%)	<0.001		
Client satisfied with how FGM was addressed by provider during clinic visit	684 (84%) vs. 384 (44%)	<0.001		
Mean score of PCC approach (0 – 5)	3.9 (3.8-4.0) vs. 1.6 (1.5-1.8)	<0.001		
Mean score of PCC + appropriate FGM prevention & care (0 – 8)	6.2 (5.9-6.6) vs. 3.7 (3.2-4.1)	<0.001		
**ANC client outcomes*** (Intervention n=819, Control n=810)**				
Clients reporting less support for FGM after ANC clinic visit	424 (52%) vs. 237 (29%)	<0.001	2.4 (2.0-3.0)	<0.001
Clients reporting that they were strongly opposed to FGM	498 (61%) vs. 382 (47%)	<0.001	1.7 (1.4-2.1)	<0.001
Clients reporting that they intend to have their daughters cut	96 (12%) vs. 209 (26%)	<0.001	0.4 (0.3-0.5)	<0.001
Clients reporting that they would prefer health care provider to cut daughters	53 (7%) vs. 139 (17%)	<0.01	0.4 (0.3-0.5)	<0.001
Clients wishing to be active in FGM prevention	677 (83%) vs. 535 (66%)	<0.01	2.2 (1.8-2.9)	<0.001

For peer review only - http://bmjopen.bmj.com/site/about/guidelines.xhtml

*Higher score indicates less supportive attitude

** Adjusted for sex, years of service, FGM status, FGM-related training, any specific training on communication/counseling and PCC, and whether the provider had conducted FGM in the past

*** Adjusted for age, educational level, FGM status and exposure to level one IEC materials

For peer review only

For peer review only - http://bmjopen.bmj.com/site/about/guidelines.xhtml

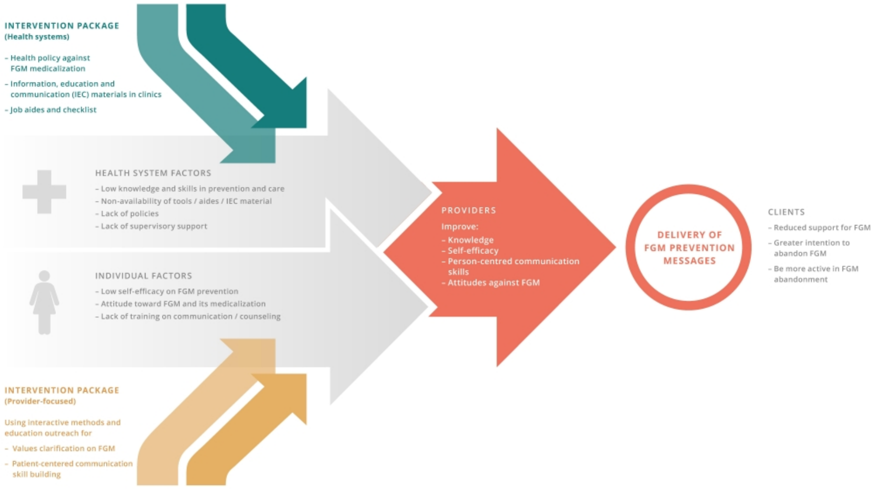

381x245mm (600 x 600 DPI)

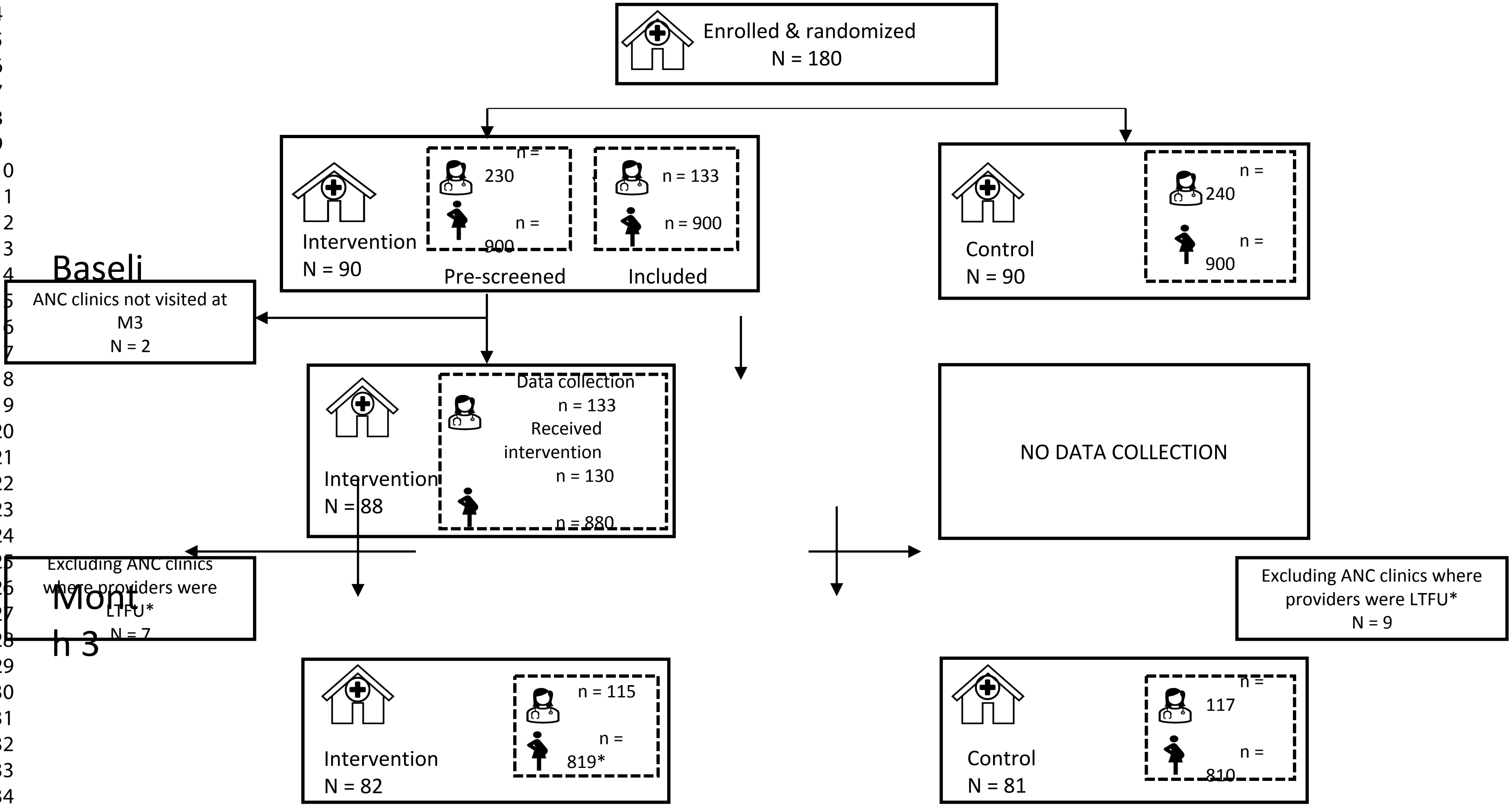
Enrolled & randomized
N = 180
Intervention
N = 90
n = 230
n = 900
Pre-screened
n = 133
n = 900
Included
Control
N = 90
n = 240
n = 900
Baseli
ANC clinics not visited at M3
N = 2
Intervention
N = 88
Data collection
n = 133
Received intervention
n = 130
n = 880
NO DATA COLLECTION
Excluding ANC clinics where providers were LTFU*
N = 7
Mont
h 3
Excluding ANC clinics where providers were LTFU*
N = 9
Intervention
N = 82
n = 115
n = 819*
Control
N = 81
n = 117
n = 810

World Health Organization

A Health Systems Approach to Prevention of Female Genital Mutilation using Person-Centered Communication: A Multi-Country Implementation Research Project

Health Facility Checklist (CHK)

CHK
Page 1/1
V1.1 (20Jul2020)

Project ID A 6 5 9 9 3 **Centre ID** ☐☐☐☐ - **Region/County ID** ☐ - **Facility ID** ☐☐☐

Study period: ☐
0 = Baseline, 2 = Six Months

SECTION A - FACILITY FUNCTIONALITY

The following questions should be directed to the health facility manager.

I am going to ask you a few questions about this health facility

1. Number of ANC providers: ☐

2. Average number of ANC clients per month: ☐☐☐

3. Number of ANC providers who received training as part of the study ☐
   1 = All
   2 = Some
   3 = None (***Skip to Q8***)

   3a) If ***All*** or ***Some***, specify number trained ☐

4. Number of MoH supervisory visits to the clinic in the past 12 months: ☐☐

5. How frequently are staff meetings held? ☐
   1 = Monthly
   2 = Every 2 to 4 months
   3 = Every 6 to 12 months
   4 - More than 12 months
   5 = Never

6. What is the size of the population served by this facility? (***specify number***) ☐☐☐☐

SECTION B - FACILITY CONTEXT

I am going to ask you about anti or pro FGM activities in the area served by this health facility

7. Are there anti-FGM activities that target the population served by this health facility? ☐
   1 = Yes
   2 = No
   3 = I don't know

   7a) If ***Yes***, specify: ____________________

   ____

8. Are there pro-FGM activities that target the population served by this health facility? ☐
   1 = Yes
   2 = No
   3 = I don't know

   8a) If ***Yes***, specify: ____________________

   ____

SECTION C - FACILITY OBSERVATION

Check around the facility for the following:

9. Is there an MoH policy on FGM posted on the wall? ☐
   1 = Yes
   2 = No

   9a) If ***Yes***, is it placed where health care providers can see/read it e.g. bulletin board? ☐
   1 = Yes 2 = No

10. Are there WHO FGM prevention posters on the wall of the consultation and/or waiting room? ☐
   1 = Yes 2 = No

   10a) If ***Yes***, are they placed in a place where ANC clients can see them? ☐
   1 = Yes 2 = No

11. Is there a WHO FGM Clinical Handbook in the ANC consultation room? ☐
   1 = Yes 2 = No

   11a) If ***Yes***, is it placed where ANC providers can see it/use it? ☐
   1 = Yes 2 = No

12. Is there an FGM ABCD guide in the ANC consultation room? ☐
   1 = Yes 2 = No

   12a) If ***Yes***, is it placed where ANC providers can see it/use it? ☐
   1 = Yes 2 = No

COMMENTS

____

Data Collector name: ***Signature:***

____

Date:

Day		Month		Year			

Time questionnaire completed (00:00 - 23:59): ☐☐ : ☐☐
hours **minutes**

For peer review only

BMJ Open

World Health Organization

A Health Systems Approach to Prevention of Female Genital Mutilation using Person-Centered Communication: A Multi-Country Implementation Research Project

ANC Provider Screening Questionnaire (SCR)

SCR
Page 1/1
V1.1 (20Jul2020)

Project ID	Centre ID	Region/ County ID	Facility ID	Provider ID
A 6 5 9 9 3	☐☐☐☐ -	☐ -	☐☐☐ -	☐

SECTION A - SOCIO-DEMOGRAPHIC INFORMATION

I am going to ask you a few questions about yourself

1. How old are you (years)? *('99' if unknown)* ☐☐

2. What is your sex? (***Observe and document***) ☐
   - 1 = Female
   - 2 = Male

For Somali study site, skip to Q4

3. What is your religion? ☐
   - 0 = None
   - 1 = Muslim
   - 2 = Christian
   - 3 = Other
   - 4 = Refused to answer
   - 3a) If ***Other*** , specify: ____________________

4. What is the highest education level you achieved? ☐
   - 1 = Certificate
   - 2 = Diploma
   - 3 = Bachelors
   - 4 = Masters or above
   - 5 = Other
   - 4a) If ***Other*** , specify: ____________________

5. What is your current professional title? ☐
   - 1 = Midwife — 3 = Nurse - Midwife
   - 2 = Nurse — 4 = Other
   - 5a) If ***Other*** , specify: ____________________

6. For how many years have you been working in your current professional title?*('99' if unknown)* ☐☐

SECTION B - TRAINING

Now, I am going to ask you a few questions about specific trainings you may have received

7. During your clinical training, did you receive any formal training on female genital mutilation? ☐
   - 1 = Yes — 3 = I don't know (***Skip to Q10***)
   - 2 = No (***Skip to Q10***)

8. When did you receive this training? ☐
   - 1 = During my studies (pre-service training)
   - 2 = After graduation/at work (in-service training)
   - 3 = Both options 1 and 2

9. What was the format of this training?
   - 1 = Yes — 3 = I don't know
   - 2 = No
   - 9a) Classroom lessons ☐
   - 9b) Workshops ☐

9. ***Continued***
   - 1 = Yes — 3 = I don't know
   - 2 = No
   - 9c) Digital format (E-learning videos; smart phone app) ☐
   - 9d) During clinical practice under supervision of a mentor ☐
   - 9e) Other ☐
   - 9es) If ***Other*** , specify: ____________________

10. During your pre- or post- graduate training, did you receive any formal training on communication or counselling? ☐
   - 1 = Yes — 3 = I don't know
   - 2 = No

11. During you pre or post graduate training, did you receive any formal training on person-centered care? ☐
   - 1 = Yes — 3 = I don't know
   - 2 = No

SECTION C - FGM HISTORY

Now, I will ask you a few personal questions about FGM

If Male provider, Skip to Q13

12. Many women in your community have had their genitals cut when they were children. If you are comfortable telling me, can I ask if you have undergone this practice? ☐
   - 1 = Yes — 3 = I don't know
   - 2 = No — 4 = Refused to answer

13. Have you ever cut the genitals of a girl or a woman for non-health reasons? ☐
   - 1 = Yes — 3 = Refused to answer
   - 2 = No
   - 13a) If ***Yes*** , have you ever cut a girl <18 years? ☐
     - 1 = Yes — 3 = Refused to answer
     - 2 = No

COMMENTS

____

Data Collector name: ***Signature:***

Date:

Day		Month		Year			

Time questionnaire completed (00:00 - 23:59): ☐☐ : ☐☐
hours minutes

For peer review only - http://bmjopen.bmj.com/site/about/guidelines.xhtml

For peer review only

World Health Organization

A Health Systems Approach to Prevention of Female Genital Mutilation using Person-Centered Communication: A Multi-Country Implementation Research Project

ANC Provider questionnaire (HCP)

HCP
Page 1/3
V1.1 (20Jul2020)

Project ID A 6 5 9 9 3 — **Centre ID** — **Region/ County ID** — **Facility ID** — **Provider ID** — **Study period:** ☐
0 = Baseline, 1 = Three months, 2 = Six Months

SECTION A - FGM KNOWLEDGE

I am going to ask you a few questions regarding FGM

1. Have you ever heard about female genital mutilation? ☐
   1 = Yes
   2 = No

2. Do the women in your community undergo female genital mutilation? ☐
   1 = Yes 3 = I don't know
   2 = No

3. Do you know of the WHO classification for female genital mutilation? ☐
   1 = Yes
   2 = No (***Skip to Q5***)

4. Please provide the WHO classification for the following FGM images (***to include images***)
   1 = Type I 4 = Type IV
   2 = Type II 5 = I don't know
   3 = Type III 6 = Other

   4a) IMAGE of Type IV FGM to be inserted here ☐
   4as) If ***Other***, specify: ____________

   4b) IMAGE of Type I FGM to be inserted here ☐
   4bs) If ***Other***, specify: ____________

   4c) IMAGE of Type II FGM to be inserted here ☐
   4cs) If ***Other***, specify: ____________

   4d) IMAGE of Type III FGM to be inserted here ☐
   4ds) If ***Other***, specify: ____________

5. Do you know of any health complications arising from female genital mutilation? ☐
   1 = Yes 2 = No

6. Is female genital mutilation illegal in your country ***(specify actual study country)?*** ☐
   1 = Yes 3 = I don't know
   2 = No

7. Are you aware of any existing WHO tools/guidance on FGM prevention and care? ☐
   1 = Yes 2 = No
   7a) If ***Yes***, please specify: ____________

8. When you treat or attend to a girl or a woman with female genital mutilation, how confident are you that you have enough knowledge to provide good quality health care? ☐
   1 = Not confident
   2 = Somewhat confident
   3 = Confident
   4 = Refused to answer

9. How confident are you in your knowledge to communicate on FGM prevention? ☐
   1 = Not confident
   2 = Somewhat confident
   3 = Confident
   4 = Refused to answer

10. Would you like to receive more training related to care for women and girls with FGM? ☐
   1 = Yes 2 = No

11. Would you like to receive more training on how to help patients prevent FGM? ☐
   1 = Yes 2 = No

SECTION B - FGM ATTITUDE

For each of the following statements please state if you agree/disagree or don't know.
1 = Agree 3 = I Don't know
2 = Disagree 4 = Refused to answer

12. A girl who has not undergone FGM is unclean ☐
13. A girl who has not undergone FGM cannot be married within her community ☐
14. A girl who has not undergone FGM is a disgrace to her family's honour ☐
15. Health care providers who perform FGM are violating medical ethics ☐
16. Health care providers who perform FGM should be punished ☐
17. FGM is a good practice ☐
18. FGM is a violation of women's and girls' rights ☐
19. FGM is a religious mandate ☐

For peer review only

World Health Organization

A Health Systems Approach to Prevention of Female Genital Mutilation using Person-Centered Communication: A Multi-Country Implementation Research Project

ANC Provider questionnaire (HCP)

HCP
Page 2/3
V1.1 (20Jul2020)

**Project ID**	**Centre ID**	**Region/ County ID**	**Facility ID**	**Provider ID**	**Study period:**
A 6 5 9 9 3		-	-	-	**0 = Baseline, 1 = Three months, 2 = Six Months**

SECTION C - FGM PRACTICE

Now, I am going to ask what you will do in specific situations regarding FGM

20. Pretend you had a daughter now who was at an age when cutting occurs, what would your intention to cut her be?
    1 = Intend to cut her
    2 = Do not intend to cut her
    3 = I don't know
    4 = Refused to answer

21. If a family brought their daughter to the clinic requesting genital cutting for non-health reasons, would you perform it?
    1 = Yes
    2 = No
    3 = I don't know
    4 = Refused to answer

22. How often do you discourage a pregnant woman expecting to have a girl, or one having a girl at the age of cutting, from having her daughter cut?
    1 = Always
    2 = Often
    3 = Sometimes
    4 = Rarely
    5 = Never
    6 = Refused to answer

23. If you became aware of a colleague performing female genital mutilation, will you …
    1 = Yes
    2 = No
    3 = I don't know
    4 = Refused to answer

    23a) Report him/her?

    23b) Explain to him/her that health care providers should not perform female genital mutilation?

24. How often do you look for female genital mutilation when performing a gynecological examination of the vulva?
    1 = Always 4 = Rarely
    2 = Often 5 = Never
    3 = Sometimes

25. How often do you record female genital mutilation in the woman's medical file if you are aware that she has undergone FGM?
    1 = Always 4 = Rarely
    2 = Often 5 = Never
    3 = Sometimes

SECTION D - CONFIDENCE

Now I would like to ask you a few questions about how you solve problems that you face. Please tell me how much you agree or disagree with the statements that I read to you

1 = Strongly disagree
2 = Disagree
3 = Neither agree nor disagree
4 = Agree
5 = Strongly agree

26. I will be able to achieve most of the goals that I have set for myself

27. When facing difficult tasks, I am certain that I will accomplish them

28. In general, I think that I can obtain outcomes that are important to me

29. I believe I can succeed at almost any endeavour to which I set my mind

30. I will be able to successfully overcome many challenges

31. I am confident that I can perform effectively on many different tasks

32. Compared to other people, I can do most tasks very well

33. Even when things are tough, I can perform quite well

For peer review only

World Health Organization	A Health Systems Approach to Prevention of Female Genital Mutilation using Person-Centered Communication: A Multi-Country Implementation Research Project<br>ANC Provider questionnaire (HCP)	HCP<br>Page 3/3<br>V1.1 (20Jul2020)

Project ID A 6 5 9 9 3 **Centre ID** - **Region/ County ID** - **Facility ID** - **Provider ID** **Study period:** 0 = Baseline, 1 = Three months, 2 = Six Months

SECTION E - COMMUNICATION SKILLS

Now, I will ask you questions about your communication skills

34. I can put myself in others' shoes
    1 = Always
    2 = Often
    3 = Sometimes
    4 = Rarely
    5 = Never

35. I let others know I understand what they say
    1 = Always
    2 = Often
    3 = Sometimes
    4 = Rarely
    5 = Never

36. In conversations with my colleagues, I perceive not only what they say but what they don't say
    1 = Always
    2 = Often
    3 = Sometimes
    4 = Rarely
    5 = Never

37. I communicate effectively
    1 = Always
    2 = Often
    3 = Sometimes
    4 = Rarely
    5 = Never

38. I communicate with others as though they are my equals
    1 = Always
    2 = Often
    3 = Sometimes
    4 = Rarely
    5 = Never

SECTION F - HEALTH FACILITY READINESS

These next questions relate to your clinic setting:

39. Have you seen any FGM posters at the clinic?
    1 = Yes
    2 = No
    3 = I don't know

40. Have you referred to the WHO Clinical Handbook on FGM?
    1 = Yes
    2 = No, available but not referred
    3 = No, not available
    4 = I don't know

41. Do you think it is feasible to provide FGM prevention counselling during ANC visits?
    1 = Yes
    2 = No
    3 = I don't know

COMMENTS

Data Collector name: ***Signature:***

Date:

Day	Month	Year

Time questionnaire completed (00:00 - 23:59): __ __ : __ __
hours minutes

For peer review only

World Health Organization

A Health Systems Approach to Prevention of Female Genital Mutilation using Person-Centered Communication: A Multi-Country Implementation Research Project

First ANC Client Exit Questionnaire (EXT)

EXT
Page 1/1
V1.1 (20Jul2020)

Project ID A 6 5 9 9 3 | **Centre ID** ____ - **Region/ County ID** __ - **Facility ID** ___ - **Participant ID** ____

Study period: ☐
0 = Baseline, 1 = Three months, 2 = Six Months

SECTION A - SOCIO-DEMOGRAPHIC INFORMATION

I am going to ask you a few personal questions

1. How old are you (years)? *('99' if unknown)* ☐☐

For Somali study sites, skip to Q3

2. What is your religion? ☐
   - 0 = None
   - 1 = Muslim
   - 2 = Christian
   - 3 = Other
   - 4 = Refused to answer
   - 2a) If **Other**, specify: ____________

3. What is the highest level of education you achieved? ☐
   - 0 = None
   - 1 = Primary
   - 2 = Secondary
   - 3 = University
   - 4 = Other
   - 3a) If **Other**, specify: ____________

SECTION B - CLINIC EXPERIENCE

The following questions relate to your clinic visit today.

During your visit today:

1 = Yes | 2 = No | 3 = I don't know | 4 = Refused to answer

4. Did you see any FGM poster(s) in the consultation and/or waiting room? ☐
5. Did the ANC provider ask if you have undergone FGM? ☐
6. Did the ANC provider explain how FGM can harm your health? ☐
7. Did the ANC provider ask about your personal belief regarding FGM? ☐
8. Did the ANC provider discuss why FGM should be prevented? ☐
9. Did the ANC provider discuss how FGM could be prevented? ☐
10. Did you have any questions about FGM to ask the ANC provider? ☐
11. Did you feel encouraged to ask questions about FGM? ☐
12. Are you satisfied with how FGM was addressed during your visit with your ANC provider today? ☐

SECTION C - FGM ATTITUDE AND PRACTICE

Now, I am going to ask your opinions regarding FGM

13. What do you feel about FGM now as compared to before you came to the clinic today? ☐
    - 1 = Same, no change
    - 2 = I feel more supportive of FGM now as compared to before I came
    - 3 = I feel less supportive of FGM now as compared to before I came
    - 4 = I don't know
    - 5 = Other
    - 6 = Refused to answer
    - 13a) If **Other**, specify: ____________

14. How supportive are you of female genital mutilation? ☐
    - 1 = Strongly opposed
    - 2 = Somewhat opposed
    - 3 = Neutral (neither opposed nor supportive)
    - 4 = Somewhat supportive
    - 5 = Strongly supportive
    - 6 = Refused to answer

15. Many women in your community have had their genitals cut when they were children. If you are comfortable telling me, can I ask if you have undergone this practice? ☐
    - 1 = Yes
    - 2 = No
    - 3 = I don't know
    - 4 = Refused to answer

16. Pretend you had a daughter now who was at an age when cutting occurs, what would your intention to cut her be? ☐
    - 1 = Intend to cut her
    - 2 = Do not intend to cut her (***Skip to Q18***)
    - 3 = I don't know (***Skip to Q18***)
    - 4 = Refused to answer (***Skip to Q18***)

17. If Intending to cut, who would you prefer to do the cutting?
    - 1 = Traditional practitioner
    - 2 = Health care provider
    - 3 = Other
    - 4 = Refused to answer
    - 17a) If **Other**, specify: ____________

18. Do you wish/want to be active in preventing FGM? ☐
    - 1 = Yes
    - 2 = No
    - 3 = I don’t know
    - 4 = Refused to answer

COMMENTS

Data Collector name: ***Signature:***

Date: Day | Month | Year

Time questionnaire completed (00:00 - 23:59): ☐☐ : ☐☐
hours **minutes**

For peer review only - http://bmjopen.bmj.com/site/about/guidelines.xhtml

1. ***Primary Outcome:** Health facility preparedness to provide FGM prevention and care services.*

***Outcome definition**: Cumulative score based on affirmative responses to Q9a, Q10a, Q11a & Q12a on the CHK form (see below).*

Q9. Is there an MoH policy on FGM posted on the wall?
Yes
No
Q9a. If yes, is it placed where health care providers can see/read it e.g., bulletin board?
Yes
No
Q10. Are there WHO FGM prevention posters on the wall of the consultation room and/or waiting room?
Yes
No
Q10a. If yes, are they placed in a place where ANC clients can see them?
Yes
No
Q11. Is there a WHO clinical handbook in the ANC consultation room?
Yes
No
Q11a If yes, is it placed where ANC providers can see/use it?
Yes
No
Q12. Is there an FGM ABCD guide in the ANC consultation room?
Yes
No
Q12a. If yes, is it placed where ANC providers can see/use it?
Yes
No

2. ***Primary outcome: ANC provider utilization of Level 1 package components***

***Outcome definition**: Affirmative response on Q40 of HCP form (see below).*

Q40. Have you referred to the WHO Clinical Handbook on FGM?
Yes
No, available but not referred
No, not available
Don't know

3. ***Primary outcome: Provision of FGM-related care after PCC training***

***Outcome definition:** Cumulative score based on affirmative responses (Provision of FGM-related care (after PCC training) either 'Always' or 'Often') on Q22, Q24 & Q25 on the HCP form (see below).*

Q22. How often do you discourage a pregnant woman expecting to have a girl, or one having a girl at the age of cutting, from having her daughter cut?
Always
Often
Sometimes
Rarely
Never
Rarely

For peer review only

Refused to answer

Q24. How often do you look for female genital mutilation when performing a gynecological examination of the vulva?

Always
Often
Sometimes
Rarely
Never
Rarely
Refused to answer

Q25. How often do you record female genital mutilation in the woman's medical file if you are aware that she has undergone FGM?

Always
Often
Sometimes
Rarely
Never
Rarely
Refused to answer

4. *Primary Outcome: Delivery of PCC 'ABCD' package*

Outcome definition: *Cumulative score based on affirmative responses on Q5, Q7, Q8, Q9 & Q12 on the EXT form.*

Q5. Did the ANC provider ask if you have undergone FGM?

Yes
No
Don't know
Refused

Q7. Did the ANC provider ask about your personal belief regarding FGM?

Yes
No
Don't know
Refused

Q8. Did the ANC provider discuss why FGM should be prevented?

Yes
No
Don't know
Refused

Q9. Did the ANC provider discuss how FGM could be prevented?

Yes
No
Don't know
Refused

Q12. Are you satisfied with how FGM was addressed during your visit with your ANC provider today?

Yes
No
Don't know
Refused

For peer review only

5. ***Secondary Outcome: Improved knowledge about FGM***

Outcome definition: *Cumulative score based on correct responses to Q4 + affirmative responses to Q5 & Q7 of the HCP form.*

Q4. Please provide the WHO classification for the following images
Type I
Type II
Type III
Type IV
Don't Know
Other

Q5. Do you know of any health complications arising from female genital mutilation?
Yes
No

Q7. Are you aware of any existing WHO tools/guidance on FGM prevention and care?
Yes
No

6. ***Secondary Outcome: Improved interpersonal communication skills***

Outcome definition: *Cumulative score based on positive responses ("Always or Often') to Q34, Q35, Q36, Q37, Q38 on the HCP form.*

Now I will ask you about your communication skills

34. I can put myself in others shoes
Always
Often
Sometimes
Rarely
Never
Rarely
Refused to answer

35. I let others know that I understand what they say
Always
Often
Sometimes
Rarely
Never
Rarely
Refused to answer

36. In conversations with my colleagues, I perceive not only what they say but what they don't say
Always
Often
Sometimes
Rarely
Never
Rarely
Refused to answer

37. I communicate effectively
Always
Often
Sometimes
Rarely

For peer review only

Never
Rarely
Refused to answer

38. I communicate with others as though they are my equals

Always
Often
Sometimes
Rarely
Never
Rarely
Refused to answer

7. Secondary outcome: Improved self-efficacy

***Outcome definition:** Cumulative score based on positive responses (Agree or Strongly Agree) to Q26, Q27, Q28, Q29, Q30, Q31, Q32, Q33 on the HCP form.*

Now I would like to ask you a few questions about how you solve problems that you face. Please tell me how much you agree or disagree with the statements that I read to you

1 = Strongly disagree
2 = Disagree
3 = Neither agree nor disagree
4 = Agree
5 = Strongly agree

Q26. I will be able to achieve most of the goals that I have set for myself
Q27. When facing difficult tasks, I am certain that I will accomplish them
Q28. In general, I think that I can obtain outcomes that are important to me
Q29. I believe that I can succeed at almost any endeavor to which I set my mind
Q30. I will be able to successfully overcome many challenges
Q31. I am confident that I can perform effectively on many different tasks
Q32. Compared to other people, I can do most tasks very well
Q33. Even when things are tough, I can perform quite well

8. Secondary outcome: Improved attitudes towards FGM

***Outcome definition**: Cumulative score based on positive responses to Q12, Q13, Q14, Q15, Q16, Q17, Q18 & Q19 on the HCP form.*

For each of the following statements please state if you:

1=Agree
2=Disagree
3=Don't know
4=Refused to answer

Q12. A girl who has not undergone FGM is unclean
Q13. A girl who has not undergone FGM cannot be married within her community
Q14. A girl who has not undergone FGM is a disgrace to her family's honor
Q15. Health care providers who provide FGM are violating FGM
Q16. Health care providers who provide FGM should be punished
Q17. FGM is a good practice
Q18. FGM is a violation of women and girls' rights
Q19. FGM is religious mandate

For peer review only

9. ***Tertiary outcome: ANC provider confidence in FGM knowledge to provide care***

Outcome definition: *Positive responses ('Somewhat Confident' or 'Confident') to Q8 & Q9 on the HCP form*

Q8. When you treat or attend to a girl or woman with female genital mutilation, how confident are you that you have enough knowledge to provide good quality care?

1=Not confident
2=Somewhat confident
3=Confident
4=Refused to answer

Q9. How confident are you in your knowledge to communicate on FGM prevention?

1=Not confident
2=Somewhat confident
3=Confident
4=Refused to answer

10. ***Tertiary outcome: ANC provider support for FGM***

Outcome definition: *Positive response ('Do not intend to cut her') to Q20 on the HCP form*

Q20. Pretend you had a daughter now who was at an age when cutting occurs, what would your intention to cut her be?

1=Intend to cut her
2=Do not intend to cut her
3=Don't know
4=Refused to answer

11. ***Tertiary outcome: ANC provider support for medicalized FGM***

Outcome definition: *Correct response ('No') to Q21 on HCP form*

Q21. If a family brought their daughter to the clinic requesting genital cutting, for non-health reasons, would you perform it?

1=Yes
2=No
3=Don't know
4=Refused to answer

12. ***Tertiary outcome: ANC client change in support for FGM after ANC visit***

Outcome definition: *Response to Q13 on EXT form*

Q13. What do you feel about FGM now as compared to before you came to the clinic today?

1= Same, no change
2=I feel more supportive of FGM now as compared to before I came
3=I feel less supportive of FGM now as compared to before I came
4=Don't know
5=Other
6=Refused to answer

13. ***Tertiary outcome: ANC client support or opposition to FGM***

Outcome definition: *Response to Q14 on EXT form*

Q14. How supportive are you of female genital mutilation?

1=Strongly opposed
2=Somewhat opposed
3=Neutral
4=Somewhat supportive

For peer review only

5=Strongly supportive
6=Refused to answer

14. Tertiary outcome: ANC client intention to cut after ANC visit.

Outcome definition: *Response to Q16 on EXT form*

Q.16 Pretend you had a daughter now who was at an age where cutting occurs, what would your intention to cut her be?

1=Intend to cut her
2=Do not intend to cut her
3=Don't know
4=Refused to answer

15. Tertiary outcome: ANC client choice of who to cut their daughters.

Outcome definition: *Response to Q17 on EXT form*

Q17. If intending to cut, who would you prefer to do the cutting?

1=Traditional practitioner
2=Health care provider
3=Other
4=Refused to answer

16. Tertiary outcome: ANC client wish to be active in FGM prevention

Outcome definition: *Response to Q18 on EXT form*

Q.18 Do you wish/want to be active in preventing FGM?

1=Yes
2=No
3=Don't know
4=Refused to answer

For peer review only

Reporting checklist for randomised trial.

Based on the CONSORT guidelines.

Instructions to authors

Complete this checklist by entering the page numbers from your manuscript where readers will find each of the items listed below.

Your article may not currently address all the items on the checklist. Please modify your text to include the missing information. If you are certain that an item does not apply, please write "n/a" and provide a short explanation.

Upload your completed checklist as an extra file when you submit to a journal.

In your methods section, say that you used the CONSORTreporting guidelines, and cite them as:

Schulz KF, Altman DG, Moher D, for the CONSORT Group. CONSORT 2010 Statement: updated guidelines for reporting parallel group randomised trials

		Reporting Item	Page Number
**Title and Abstract**			
Title	#1a	Identification as a randomized trial in the title.	1
Abstract	#1b	Structured summary of trial design, methods, results, and conclusions	2
**Introduction**			

For peer review only

Background and objectives	#2a	Scientific background and explanation of rationale	5
Background and objectives	#2b	Specific objectives or hypothesis	6
**Methods**			
Trial design	#3a	Description of trial design (such as parallel, factorial) including allocation ratio.	7
Trial design	#3b	Important changes to methods after trial commencement (such as eligibility criteria), with reasons	7
Participants	#4a	Eligibility criteria for participants	8
Participants	#4b	Settings and locations where the data were collected	8
Interventions	#5	The experimental and control interventions for each group with sufficient details to allow replication, including how and when they were actually administered	9,10
Outcomes	#6a	Completely defined prespecified primary and secondary outcome measures, including how and when they were assessed	11
Sample size	#7a	How sample size was determined.	11
Sample size	#7b	When applicable, explanation of any interim analyses and stopping guidelines	N/A

For peer review only

Randomization - Sequence generation	#8a	Method used to generate the random allocation sequence.	
9			
Randomization - Sequence generation	#8b	Type of randomization; details of any restriction (such as blocking and block size)	
9			
Randomization - Allocation concealment mechanism	#9	Mechanism used to implement the random allocation sequence (such as sequentially numbered containers), describing any steps taken to conceal the sequence until interventions were assigned	9
Randomization - Implementation	#10	Who generated the allocation sequence, who enrolled participants, and who assigned participants to interventions	9
Blinding	#11a	If done, who was blinded after assignment to interventions (for example, participants, care providers, those assessing outcomes) and how.	9
Blinding	#11b	If relevant, description of the similarity of interventions	N/A
Statistical methods	#12a	Statistical methods used to compare groups for primary and secondary outcomes	12
Statistical methods	#12b	Methods for additional analyses, such as subgroup analyses and adjusted analyses	12

For peer review only

Outcomes	#6b	Any changes to trial outcomes after the trial commenced, with reasons	N/A
**Results**			
Participant flow diagram (strongly recommended)	#13a	For each group, the numbers of participants who were randomly assigned, received intended treatment, and were analysed for the primary outcome	13
Participant flow	#13b	For each group, losses and exclusions after randomization, together with reason	13
Recruitment	#14a	Dates defining the periods of recruitment and follow-up	13
Recruitment	#14b	Why the trial ended or was stopped	N/A
Baseline data	#15	A table showing baseline demographic and clinical characteristics for each group	28, 29, 30, 31
Numbers analysed	#16	For each group, number of participants (denominator) included in each analysis and whether the analysis was by original assigned groups	14
Outcomes and estimation	#17a	For each primary and secondary outcome, results for each group, and the estimated effect size and its precision (such as 95% confidence interval)	16,17,18
Outcomes and estimation	#17b	For binary outcomes, presentation of both absolute and relative effect sizes is recommended	16,17,18

For peer review only

Ancillary analyses	#18	Results of any other analyses performed, including subgroup analyses and adjusted analyses, distinguishing pre-specified from exploratory	16,17,18
Harms	#19	All important harms or unintended effects in each group (For specific guidance see CONSORT for harms)	N/A
**Discussion**			
Limitations	#20	Trial limitations, addressing sources of potential bias, imprecision, and, if relevant, multiplicity of analyses	20
Interpretation	#22	Interpretation consistent with results, balancing benefits and harms, and considering other relevant evidence	22
Registration	#23	Registration number and name of trial registry	3
Generalisability	#21	Generalisability (external validity, applicability) of the trial findings	21
**Other information**			
Interpretation	#22	Interpretation consistent with results, balancing benefits and harms, and considering other relevant evidence	22
Registration	#23	Registration number and name of trial registry	3
Protocol	#24	Where the full trial protocol can be accessed, if available	3

For peer review only

Funding	#25	Sources of funding and other support (such as supply of drugs), role of funders	24

Notes:

- 15: 28, 29, 30, 31 The CONSORT checklist is distributed under the terms of the Creative Commons Attribution License CC-BY. This checklist was completed on 12. May 2023 using https://www.goodreports.org/, a tool made by the EQUATOR Network in collaboration with Penelope.ai

For peer review only

A cluster randomized trial of a health system strengthening approach applying person-centered communication for the prevention of female genital mutilation in Guinea, Kenya, and Somalia

Journal:	*BMJ Open*
Manuscript ID	bmjopen-2023-078771.R1
Article Type:	Original research
Date Submitted by the Author:	02-Apr-2024
Complete List of Authors:	Balde, Mamadou ; Cellulle de Recherche en Sante de la Reproduction en Guinee (CERREGUI)<br>Ndavi, Patrick; University of Nairobi College of Health Sciences, Department of Obstetrics & Gynecology<br>Oyaro, Vernon; World Health Organization, Department of Sexual and Reproductive Health and Research<br>Soumah, Anne-Marie; Cellulle de Recherche en Sante de la Reproduction en Guinee (CERREGUI)<br>Esho, Tammary; Amref International University<br>King'oo, James; Technical University of Kenya<br>Kemboi , Jackline; Amref Health Africa<br>Sall, Alpha; Cellulle de Recherche en Sante de la Reproduction en Guinee (CERREGUI)<br>Diallo, Aissatou; Cellulle de Recherche en Sante de la Reproduction en Guinee (CERREGUI)<br>Ahmed, Wisal; World Health Organization, Department of Sexual and Reproductive Health and Research<br>Stein, Karin; World Health Organization<br>Nosirov, Khurshed; World Health Organization, Department of Sexual and Reproductive Health and Research<br>Thwin, Soe Soe; World Health Organization, Department of Sexual and Reproductive Health and Research, including UNDP/UNFPA/UNICEF/WHO/World Bank Special Programme of Research, Development and Research Training in Human Reproduction (HRP)<br>Petzold, Max; University of Gothenburg Sahlgrenska Academy, Public Health and Community Medicine<br>Ahmed, Muna; Ministry of Planning and National Development, Central Statistics Department; MUFEIS Multidisciplinary Consultancy Firm , CEO<br>Diriye, Ahmed; Data and Research Solutions<br>Pallitto, C ; World Health Organization, Department of Sexual and Reproductive Health and Research
**Primary Subject Heading**:	Health services research
Secondary Subject Heading:	Evidence based practice, Reproductive medicine, Research methods, Communication, Complementary medicine

Keywords:	EDUCATION & TRAINING (see Medical Education & Training), Patient-Centered Care, Primary Care < Primary Health Care, Primary Prevention, PUBLIC HEALTH, Behavior

SCHOLARONE™
Manuscripts

I, the Submitting Author has the right to grant and does grant on behalf of all authors of the Work (as defined in the below author licence), an exclusive licence and/or a non-exclusive licence for contributions from authors who are: i) UK Crown employees; ii) where BMJ has agreed a CC-BY licence shall apply, and/or iii) in accordance with the terms applicable for US Federal Government officers or employees acting as part of their official duties; on a worldwide, perpetual, irrevocable, royalty-free basis to BMJ Publishing Group Ltd ("BMJ") its licensees and where the relevant Journal is co-owned by BMJ to the co-owners of the Journal, to publish the Work in this journal and any other BMJ products and to exploit all rights, as set out in our licence.

The Submitting Author accepts and understands that any supply made under these terms is made by BMJ to the Submitting Author unless you are acting as an employee on behalf of your employer or a postgraduate student of an affiliated institution which is paying any applicable article publishing charge ("APC") for Open Access articles. Where the Submitting Author wishes to make the Work available on an Open Access basis (and intends to pay the relevant APC), the terms of reuse of such Open Access shall be governed by a Creative Commons licence – details of these licences and which Creative Commons licence will apply to this Work are set out in our licence referred to above.

Other than as permitted in any relevant BMJ Author's Self Archiving Policies, I confirm this Work has not been accepted for publication elsewhere, is not being considered for publication elsewhere and does not duplicate material already published. I confirm all authors consent to publication of this Work and authorise the granting of this licence.

For peer review only

A cluster randomized trial of a health system strengthening approach applying person-centered communication for the prevention of female genital mutilation in Guinea, Kenya, and Somalia

Authors*:* *Prof. Mamadou Balde, MD[1*], Prof. Patrick Ndavi, MMed[2*], Dr. Vernon Mochache, PhD[3] Dr. Anne-Marie Soumah, MSc[1], Prof. Tammary Esho, PhD[4], James Munyao King'oo, MSc[5], Jackline Kemboi, MSc[2], Alpha Oumar Sall, MSc[1], Aissatou Diallo, MSc[1], Dr. Wisal Ahmed, PhD[3], Dr. Karin Stein, MD[3], Khurshed Nosirov, MCS[3], Dr. Soe Soe Thwin, PhD[3], Prof. Max Petzold, PhD[6], Muna Abdi Ahmed, MSc[7], Ahmed Diriye, MA[8], Dr. Christina Pallitto, PhD[3]*

Institutional Affiliations*:* [1]Centre for Research in Reproductive Health in Guinea, Conakry, Guinea; [2]Department of Obstetrics and Gynecology, University of Nairobi, Nairobi, Kenya; [3]Department of Sexual and Reproductive Health and Research, and the UNDP/UNFPA/UNICEF/WHO/World Bank Special Programme of Research, Development and Research Training in Human Reproduction (HRP), World Health Organization, Geneva, Switzerland; [4]Amref International University, Nairobi, Kenya; [5]Technical University of Kenya, Nairobi, Kenya; [6]School of Public Health and Community Medicine, Institute of Medicine, University of Gothenburg, Gothenburg, Sweden; [7] Somaliland Central Statistics Department, Hargeisa, Somalia; [8]Data and Research Solutions, Hargeisa, Somalia

** Joint first authors*

Correspondence to:

Dr. Christina Pallitto,

World Health Organization,

Department of Sexual and Reproductive Health and Research

20 Avenue Appia, 1211, Geneva, Switzerland

Telephone: +41 22 791 4745

Email: pallittoc@who.int

For peer review only

For peer review only - http://bmjopen.bmj.com/site/about/guidelines.xhtml

Abstract word count: 293

Text word count: 5,314

Tables: 4

Figures: 1

For peer review only

For peer review only - http://bmjopen.bmj.com/site/about/guidelines.xhtml

ABSTRACT

Introduction: There is limited evidence on effective health systems interventions for preventing female genital mutilation (FGM). This study tested a two-level health system strengthening approach at primary care level to apply person-centred communication (PCC) for FGM prevention.

Methods: Between August 2020 and September 2021, a cluster randomized trial was conducted in 180 antenatal care (ANC) clinics in Guinea, Kenya, and Somalia. At baseline, all clinics received guidance and materials on FGM prevention and care, while at month three, ANC providers at intervention sites received PCC training. Data were collected from clinic managers, ANC providers and clients at baseline, months three and six. Multi-level and single-level logistic regression models were used to analyze the effect of the intervention on study outcomes.

Results: Complete data were collected from 232 ANC providers in 163 clinics. Compared to providers in the control arm, those in the intervention arm had higher odds of being confident in their FGM-related knowledge (OR: 6.3, 95% CI: 1.4-28.9; p=0.02) and to communicate effectively about FGM prevention (OR: 1.7; 95% CI: 1.0-3.0; p=0.06). Additionally, ANC clients in the intervention arm had higher odds of being less supportive of FGM (AOR: 5.4, 95% CI: 2.4-12.4; p<0.001] and wanting to be actively engaged in FGM prevention (AOR: 3.2, 95% CI: 1.6-6.2; p=0.001) after speaking with their provider. They also had higher odds of being strongly opposed to FGM (AOR: 2.4, 95% CI: 1.1-5.2; p=0.023), lower odds of intending to have their daughters undergo FGM (AOR: 0.3, 95% CI: 0.1-0.7; p=0.004) or seeking medicalized FGM (AOR: 0.2, 95% CI: 0.1-0.5; p<0.001).

Conclusion: This is the first randomized trial to provide evidence of an effective intervention to promote FGM prevention that can be delivered in primary care settings and scaled up in high prevalence countries.

For peer review only - http://bmjopen.bmj.com/site/about/guidelines.xhtml

SUMMARY BOX

- This hybrid-effectiveness implementation research study conducted in primary care public health facilities in three countries with high prevalence of female genital mutilation (FGM) assessed the role of health workers in providing FGM prevention communication in the context of routine antenatal care (ANC).
- It will be important to assess the effectiveness of the person-centred communication approach in other service delivery points, e.g., child immunization, and with other cadres of health workers, e.g., community health workers, to assess its effectiveness beyond ANC care.
- Many factors influence FGM-related decision-making, and while primary care health workers were found to be effective communicators, and the randomized design controlled for some external factors, the impact of a health sector intervention in conjunction with multi-sectoral initiatives requires futher investigation.
- To ensure participation of at least one ANC provider at each site through each time point, eligibility of health workers was based on clinic rotation schedules, which may have introduced a selection bias although the included and excluded providers did not appear to differ significantly.

For peer review only

For peer review only - http://bmjopen.bmj.com/site/about/guidelines.xhtml

INTRODUCTION

Multi-sectoral efforts are needed to achieve Sustainable Development Goal (SDG) 5.3 to eliminate the harmful practice of female genital mutilation (FGM) by 2030 in line with the United Nation's (UN) General Assembly resolution 67/146 (1), the World Health Assembly Resolution 61.16 (2) and the 2008 Interagency Statement (3), which call upon UN Member States to enact comprehensive and multi-disciplinary national action plans and strategies towards the elimination of the practice. Identifying effective strategies across sectors is an important step in ending FGM.

The health system, defined as all organizations, institutions and resources that produce actions whose primary purpose is to improve health(4), has an important role to play not only in managing complications of FGM but also in preventing the practice. Health care providers, specifically nurses and midwives who constitute most of the health workforce, are highly respected members of FGM practising communities and could positively contribute to abandonment efforts (5,6). However, there is currently limited evidence to guide health programming on FGM prevention (7). In addition, some health care providers are themselves supportive of this harmful practice, and might even perform it (i.e., FGM medicalization), despite national laws and medical ethics forbidding it (8–11). Developing evidence-based tools to build skills of health care providers and address their underlying beliefs could contribute to FGM abandonment efforts and complement existing resources on management of complications (12,13) to ensure comprehensive and high quality care.

Three countries (Guinea, Somalia, and Kenya) participated in a cluster randomized trial to test the effectiveness and implementation of a health system strengthening approach to FGM, which included the testing of an intervention to build skills of health workers on applying person-centered communication

For peer review only - http://bmjopen.bmj.com/site/about/guidelines.xhtml

(PCC) for the prevention of FGM (14). Study countries were selected based on their high national and/or sub-national FGM prevalence. The national prevalence of FGM among women and girls aged 15 - 49 years is 98% in Somalia, 97% in Guinea and 21% in Kenya according to national population-based surveys. There are 20 hotspot counties/sub-national administrative units in Kenya with a prevalence of >80% (15), and this study focused on three of these counties. Likewise, the study countries have high rates of medicalized FGM, performed primarily by midwives, who make up between 71% to 93% of primary health care providers in the three study countries (16) hence the selection of nurses and midwives as the target group for this intervention.

The purpose of this study was to test a two-level intervention package to enable ANC providers to deliver person-centered FGM counseling to their clients.[1] This intervention package was informed by a theory of change that promotes health workers to be effective behavioral change agents because of their credibility (17) and positionality to influence the opinions, attitudes, beliefs, motivations and behaviors of their clients (18). We hypothesized that if ANC providers gained the necessary knowledge and skills to provide person-centered counseling (Level 2) and were given the opportunity to question their beliefs and attitudes together with an enabling environment (Level 1), they could positively influence the knowledge and attitudes of their clients to abandon the practice (Supplementary file 1).

The level one intervention consisted of making available national policy directives on the role of health care providers in providing FGM prevention and care services, WHO's FGM guidelines and clinical handbook as well as information, education, and communication (IEC) materials. These materials were distributed without any capacity building to accompany their distribution. Level two consisted of an interactive training specifically targeting ANC providers to build their knowledge on FGM, enable them to question their FGM-related values and attitudes and build their skills on counseling for FGM prevention using person-centred communication (19), a component of person-

For peer review only - http://bmjopen.bmj.com/site/about/guidelines.xhtml

centred care, which ensures that the perspectives and preferences of individuals, carers, families and communities are at the center of decisions and that they have the information and support needed to make decisions (20). ANC providers were trained to apply a series of structured steps in which they would: 'Assess' their client's views on FGM, address and challenge her 'Beliefs', encourage 'Change' and together with the client, 'Discuss and Decide' (ABCD).

METHODS

Study Design

This cluster randomized trial applied a type 2 hybrid, effectiveness-implementation design (21) to test the effectiveness of the delivery of a phased intervention package (Level 1 and 2) on knowledge, attitudes and practices among ANC health workers and their clients. This type of implementation research design assesses the effectiveness of the intervention and implementation factors in real world settings. The methodology, analysis plan and reporting conformed to the 2010 Consolidated Standards of Reporting Trial (CONSORT) checklist (22). Ethical approval for the master protocol was obtained from the World Health Organization (WHO) Ethical Review Committee (ERC) (#P151/03/2014). Each study country submitted country-specific protocols to local institutional review boards. Ethical approval was obtained in Kenya from the Kenyatta National Hospital/University of Nairobi ERC (P805/09/2019) and the National Commission for Science, Technology, and Innovation (NACOSTI/P/20/5721); in Somalia from the Department of Planning, Policy and Strategic Information, Unit of Research (MOHD/DG: 2/11526/2019); and in Guinea from the *Comité National d'Ethique Pour la Recherche en Santé* (CNERS) (105/CNERS/19).

Participants

Within each study country, two or three sub-national units (regions/counties) were purposively selected according to the following eligibility criteria: (1) FGM prevalence >50% among females 15 -

For peer review only - http://bmjopen.bmj.com/site/about/guidelines.xhtml

49 years old; (2) more than 15 ANC clinics, seeing on average 30 new ANC clients per month and (3) accessibility in terms of security. The unit of randomization was the ANC clinic to avoid having ANC providers in the same clinic in different study arms, which could lead to contamination. In intervention sites, all providers on duty were pre-screened. To ensure participation and follow-up throughout the trial, between one and three ANC providers on duty were enrolled based on a six-month clinic rotation schedule provided by the clinic manager. Ten new clients exiting their first ANC consultation with a participating provider were recruited at each data collection point.

Individual study participants gave verbal informed consent. Data collectors collected data from the ANC providers and their clients in a private and confidential setting. While personally identifiable information was collected from ANC providers to facilitate tracking during the follow-up data collection time points, data were de-identified prior to analysis. No personally identifiable information was collected from ANC clients who were unique at each time point. Participating ANC clients received the equivalent of 5 USD to compensate for their transport costs recognizing that participants consenting to participate might have changed their plans to accommodate the interviews. Given insecurity in carrying cash in Somalia, a mobile phone application was used to transfer the money to participants, an amendment to the original protocol, which was submitted to the ethical review committees.

Randomization and blinding

Based on Ministry of Health (MoH) facility administrative records, all public, primary care facilities (i.e., dispensaries and/or health centers) offering ANC services in the selected regions/counties the average number of new ANC clients seen in November and December 2019 was compiled to create ordered listings of client loads at each of the sites by region/county. Clinics were matched into pairs based on client load so the two busiest would be randomized to different arms and so on. A uniform distribution was used for randomization using the uniform random number function in STATA 17

For peer review only

For peer review only - http://bmjopen.bmj.com/site/about/guidelines.xhtml

(StataCorp Inc., College Station, TX, USA). Study teams organized data collection and intervention trainings based on the randomization lists. Attempts were made to blind clinic managers, ANC providers and their clients to study arm allocation. Since both study arms received the level one intervention component at baseline, and the providers and managers at control sites were unaware of the training that took place at intervention sites, it is conceivable that they were not aware of their study arm. Presumably, intervention clients would assume they were the intervention arm, but they were also not aware of what might have been offered to other sites. ANC clients, however, were completely blinded as to study arm allocation since a distinct set of clients was interviewed at each time point, and they would not be aware of the training the provider had had. Field data collectors were also blinded to study arm allocation as much as possible, although some might have determined intervention arm during the study.

Procedures

Implementation of the study interventions and data collection occurred between August 2020 and September 2021 and was staggered by countries. In the intervention arm, data collection was undertaken at three time points, i.e., at baseline prior to implementing the level one intervention component; at month three, prior to implementing the level two intervention component and at month six. In the control arm, data collection was done at two time points, i.e., at baseline and at month six. Study instruments included one for ANC clients, one for health workers and a health facility checklist completed by clinic managers. Instruments were pretested among ANC clients and providers from non-participating sites in all countries, and country teams provided feedback on the structure and appropriateness of each question prior to finalizing the instruments.

A web-interface electronic data capture system was developed on the Kobo toolbox core system architecture (Kobo Toolbox, Harvard Humanitarian Initiative, Boston, Massachusetts, USA). User accounts were password-protected, and data sent to the server was encrypted in transit using SHA256

For peer review only - http://bmjopen.bmj.com/site/about/guidelines.xhtml

with RSA encryption that met the data security requirements. Personally identifiable information was not collected, and all records were anonymized with unique study numbers. Study instruments for ANC clients were translated from English into ten languages by research team members in consultation with language experts (French, Somali, Swahili, Soussou, Poular, Malinké, Keiyo, Maasai, Marakwet and Tugen) while those for ANC providers and clinic managers were translated into two languages (French and Somali). No backtranslation was performed. Field data collectors and their supervisors spoke the languages in which the questionnaires were administered. Data collection teams participated in a standardized training with WHO/HRP and the research institutions in each country. The level two intervention was implemented by master trainers in each country who had been trained remotely over a three-day period following the WHO PCC for FGM prevention facilitator's manual.

Outcomes

The primary study outcome was delivery of the "ABCD" approach by ANC providers measured by responses from their client using tools developed for this study based on previously validated instruments, including four constructs of the operational definition of person-centered communication (23). We also assessed ANC provider delivery of FGM care services and their utilization of the level one intervention components. Health facility preparedness to offer FGM prevention and care was assessed using a composite score developed for this study. (Supplementary file 2).The secondary self-efficacy outcome was assessed based on a score calculated from a validated tool for measuring general self-efficacy (24) while knowledge, attitudes, and practice (KAP) on FGM prevention and care were measured using an unvalidated KAP questionnaire similar to one used in formative research in Guinea. Study instruments can be found in Supplementary file 3.

Statistical analysis

For peer review only - http://bmjopen.bmj.com/site/about/guidelines.xhtml

To have sufficient power (80%) to detect a difference (significance level 5%) between intervention and control arms on the primary study outcome of delivery of the PCC intervention for FGM prevention, 180 ANC clinics, equally divided across the three study countries were recruited and randomized with 1800 new ANC clients (10 per clinic) recruited at baseline and 1800 at six-month follow-up. While similar interventions have resulted in 20% difference between groups (25), a 10% difference (based on an assumed 20% in the control arm and 30% in the intervention arm) was applied to ensure sufficient power to detect a 10% difference and considering the minimal levels of clinical efficacy for such an intervention to be practical. This sample size also allowed for a 10% non-response and/or loss to follow-up rate and accounted for a clustering effect of (ICC=0.20) at clinic level. A relatively high level of clustering was assumed in the sample size calculations to not underestimate the needed sample size. Region/county level was not included in the multilevel model due to the low number of included regions/counties per country (Kenya 3, Guinea 2, Somalia 3) and it would then not be possible to get an accurate estimate of the variance between clusters.

Data were analyzed using STATA 17 software following a per-protocol approach. Data from ANC providers and their clients were analyzed if the clinic had at least one provider with follow up data at all study time points, and in the intervention arm, if the ANC provider present had undergone training on PCC for FGM prevention at month three. Clinics where providers were lost to follow-up were not included in the final analyses. All facility checklists and ANC client exit interviews were conducted as intended except at sites not accessible due to security issues or closed or converted for care of COVID-19 patients during the pandemic. As the study was designed to pre-screen ANC providers at baseline and include in the final analytic sample only those clinics and providers who were available at 3 and 6 months, an intention-to-treat approach was not feasible. Key characteristics of the participating facilities,

For peer review only - http://bmjopen.bmj.com/site/about/guidelines.xhtml

providers and clients were summarized. Providers and clinics that were screened but not eligible are compared in Supplementary file 4.

Continuous variables are presented using mean values, and standard deviation (SD) while categorical variables are summarized as counts (N) with percentages (%). Differences in proportions were analysed for dichotomous outcomes using Fischer's exact test. For outcomes measured as summary scores, comparisons of mean scores are presented across study arms using t-test.

Initial analyses showed that the clustering was negligible at the provider level since most sites only included one provider in the study. Therefore, multilevel regression models were not used to compare outcomes among providers in intervention vs. control arms. However, analyses based on client level outcomes applied multilevel mixed effect logistic regression models to assess differences between the study arms. Multilevel analyses were attempted for the models in which ANC clients reported on provider actions, but given the complexity of the models, convergence problems arose leading to unreliable results. In these cases, results of ordinary models are presented. Linearity was assessed for the continuous covariates included in the regression models using the Box-Tidwell test in Stata.

At month six, a comparison of study outcomes between the intervention and control arms was used to determine the combined effect of both levels of the intervention package. Multilevel multivariable logistic regression analyses for ANC provider outcomes were adjusted for their sex, years of service, FGM status, FGM-related training, any specific training on communication/counseling and PCC, and whether the provider had conducted FGM in the past. Analyses related to ANC client outcomes were adjusted for their age, educational level, FGM status and exposure to level one IEC materials. These variables were determined a priori based on previously published literature. Analyses related to provider actions as reported by clients were adjusted for client characteristics as it was not possible to definitively link a client with a particular provider. Unadjusted analyses are presented for

For peer review only - http://bmjopen.bmj.com/site/about/guidelines.xhtml

outcomes that relate to composite measures based on ANC provider and client responses (e.g., provision of FGM prevention and care services).

To determine the separate effect of the two levels of the intervention package, additional sub-analyses were conducted restricted to the intervention arm. Changes from baseline to month 3 within the intervention arm were used to determine the effect of the level one intervention component while changes from month 3 to month 6 within the same study arm were used to determine the effect of the level two intervention component. The study was not powered for these sub-analyses, however, and these results are presented in Supplementary file 4.

In-country data managers monitored data quality. Periodic data audits were conducted by the WHO/HRP Quantitative Assessment and Data Management team to identify any data collection gaps and data discrepancies requiring follow up by in-country teams. Weekly data monitoring meetings were held between the in-country research teams and WHO/HRP staff during data collection periods to identify, document and resolve any data discrepancies. These were virtual due to the COVID-19 pandemic. Given that there was no prospective follow-up of clients, a Data Safety and Monitoring Board was not established. Instead, local research teams documented and reported any unintended harms and/or protocol deviations to the WHO/HRP study coordination team.

Patient and public involvement statement

Health care providers and members of communities where the practice of FGM is prevalent in the study countries were actively involved in the design and implementation of this study intervention. This included the formative research conducted in Guinea, which identified health care providers as integral members of FGM practicing communities who understand local community beliefs and norms, making them effective change agents. The formative research also found that the health sector can support these health care providers to be effective change agents by incorporating FGM content within

For peer review only - http://bmjopen.bmj.com/site/about/guidelines.xhtml

their training, ensuring accountability to legal and policy standards and promoting FGM abandonment as part of a multi-sectoral approach. Based on this formative work, the PCC training was developed and subsequently piloted among ANC providers in Kenya before being rolled out as part of the multi-country study.

Additionally, the research partners in Guinea, Kenya and Somalia actively engaged health care providers and community members as part of their in-country work towards FGM prevention. In Kenya, as part of mobilization of study participants, community health volunteers in the study counties talked about the study during their community sensitization sessions and invited pregnant women to attend routine ANC sessions where they could be approached for participation in the study. Both health care providers and pregnant women were provided with information about the study, including the burden of the intervention as to time, any risks involved in their participation, the voluntary nature of their participation, and were recruited only after providing informed consent.

At present, study dissemination meetings have been conducted in Kenya and Guinea that have involved the MoH, other stakeholders as well as representatives of health care providers and community members where the study was implemented. In these meetings, the in-country research partners have led the development of policy briefs identifying country-specific results relevant for local research needs, policy development and practice.

Role of the funders

Apart from WHO/HRP, the study funders had no role in study design or implementation. WHO/HRP, in collaboration with in-country research teams, developed the study protocol, provided data management and analytic support, and contributed to interpretation and manuscript writing. WHO/HRP coordinated the successful implementation of this study. The data collection platform was developed and maintained by an outsourced vendor (First Data, LLC, Kenya); data management was

For peer review only - http://bmjopen.bmj.com/site/about/guidelines.xhtml

coordinated by the local implementing partners (CERREGUI, DARS and University of Nairobi) and statistical data analysis was conducted by an external statistician (Dr. Max Petzold, Gothenburg University). All these functions were conducted with utmost integrity following ICH-GCP guidelines. This trial was registered: PACTR201906696419769 (June 3, 2019).

RESULTS

Recruitment and retention

Between August 2020 and September 2021, a total of 180 ANC clinics (i.e, 60 clinics per study country) were enrolled and randomized to intervention and control arms. There was some natural staggering of the start and subsequent data collection dates due to factors, such as weather, COVID-19, Ramadan, and national elections. Data collection periods ranged from three to six weeks in each country at each time point. The time elapsed between the end of one data collection period to the beginning of the next data collection period ranged from three to five months.

In the intervention arm, 216 providers and 900 clients (i.e.., 10 per clinic) were interviewed. Based on a review of clinic rotation schedule to ensure participation of at least one provider from each study clinic throughout the trial, 133 providers were enrolled. In the control arm, 220 providers and 900 clients were interviewed. (Figure 1). At month three, data were collected at 98% (n=88) of the intervention clinics as two clinics in Kenya were inaccessible due to insecurity. One hundred and thirty (98%) ANC providers (at least one from each site) and 880 first visit ANC clients completed the month three questionnaires prior to implementing the Level 2 intervention PCC. No data collection was conducted at the control sites. At month six, 91% (n=163) of ANC clinics (81, intervention and 82, control) had at least one ANC provider (intervention n=110 and control n=122) on duty who was previously enrolled in the study. The client questionnaire was applied to 819 and 810 first visit ANC clients, respectively in the intervention and control sites.

For peer review only - http://bmjopen.bmj.com/site/about/guidelines.xhtml

Characteristics of study sites and participants

The 163 ANC clinics retained to the end of the study, had a mean of four ANC providers (standard deviation, SD: 3) and served on average 155 new ANC clients per month (SD: 127) with a mean catchment population of 36,754 people (SD: 126,082). In 55% (n=89) of clinics, the clinic manager reported that there were no activities promoting FGM prevention in the facilities' catchment area (*Table 1*). These characteristics were not different from the 17 ANC clinics that were enrolled at baseline but that subsequently were not included in the final analysis (Annex 1).

Of the 232 ANC providers who contributed data for analysis at month six, 83% (n=193) were female and their mean age was 36 years (SD: 10 years). They had an average of eight years professional experience (SD: 7 years) and 68% (n=158) had studied up to Diploma level (generally 3 years post-secondary education) with 90% (n=208) identifying as either midwives, nurses, or nurse-midwives. Health cadres were defined by national licensing requirements in each country. Among these providers, at baseline, 63% (n=146) reported that they had not received formal clinical training on FGM prevention and care (*Table 2*). Almost two-thirds (64%, n=149) reported that they had received training on communication/counselling while half (51%, n=118) had received training on person-centered care. Further, 54% (n=126) of female providers reported that they had undergone FGM while overall, 94% (n=217) of providers reported that they had never performed FGM. These characteristics were not different when compared to the ANC providers who were on duty in the 180 ANC clinics enrolled at baseline (Annex 2). The mean age of the 1,800 clients exiting their first ANC visits at baseline was 26 years (SD: 6 years), 47% (n=846) reported not having received any education, and 73% (n=1,320) reported that they had undergone FGM. These characteristics were similar to the 880 and 1,630 first visit ANC clients interviewed at month three (intervention arm only) and month six, respectively (*Table 3*).

For peer review only - http://bmjopen.bmj.com/site/about/guidelines.xhtml

To evaluate potential bias from differential selection of providers receiving the intervention, we assessed differences in baseline characteristics between the 133 ANC providers from intervention facilities who were screened at baseline and received PCC training at month three (i.e., included in the analytic sample) versus the 97 who were screened and did not receive the intervention (i.e., excluded from analytic sample). The reasons for this included the fact that some of the providers had been transferred from the study clinics or could not be released to attend the training so as not to affect service delivery. Both groups were similar in terms of sex, educational level, professional cadre, as well as whether they had undergone or recently performed FGM. However, included providers tended to be slightly younger (by two years on average) and less likely to be of Muslim religion, although the question on religion was not administered for the Somalia sample since all respondents were assumed to be Muslim (Annex 3).

ANC providers implementation of ABCD elements of the PCC approach

Table 4 presents the analysis of study outcomes by arm at month six. Compared to ANC providers in the control arm, those in the intervention arm were nearly nine times as likely to ask their clients if they had undergone FGM (OR: 8.9, 95% CI: 6.9-11.5; $p<0.001$), nearly ten times as likely to ask their clients' personal beliefs regarding FGM (OR: 9.7, 95% CI: 7.5-12.5; $p<0.001$), more than nine times as likely to discuss with their clients why FGM should be prevented (OR: 9.2, 95% CI: 7.1-11.9; $p<0.001$) and nearly eight times as likely to discuss with their clients how FGM could be prevented (OR: 7.7, 95% CI: 6.0-9.9; $p<0.001$). Further, ANC clients in the intervention arm were nearly seven times as likely to report that they were satisfied with how FGM had been addressed by their provider during the clinic visit compared to those in the control arm (OR: 6.6, 95% CI: 5.1-8.4; $p<0.001$). In the intervention arm, the mean score of implementing the ABCD elements of the PCC approach was more

For peer review only - http://bmjopen.bmj.com/site/about/guidelines.xhtml

than twice as likely (OR: 2.1, 95% CI: 1.6-2.6; p<0.001) to be higher in the intervention [3.9 (3.8-4.0)] compared to the control arm [1.6 (1.5-1.8)].

ANC clinic preparedness to provide FGM prevention and care services

A significantly higher proportion of ANC clinics in the intervention arm had all correct repornses related to facility preparedness to provide FGM prevention and care services compared to those in the control arm (68% vs. 27%, p<0.001). Additionally, ANC clinics in the intervention arm had a significantly higher mean score for preparedness compared to those in the control arm [3.4 (95% CI: 3.2-3.6) vs. 2.6 (95% CI: 2.4-2.9; p<0.001)].

ANC providers utilizing level one intervention components

A higher proportion of ANC providers in the intervention arm reported having utilized the level one intervention package components compared to those in the control arm (83% vs. 56%, p<0.001). In multivariable analyses, ANC providers in the intervention arm were nine times as likely to report having utilized the level one intervention package components compared to those in the control arm (AOR: 9.3, 95% CI: 4.2-20.8; P<0.001).

ANC providers offering appropriate FGM prevention and care services

At month six, based on a cumulative score to specific questions on provision of appropriate FGM-related prevention and care services, a higher proportion of ANC providers in the intervention arm reported that they had provided FGM prevention and care services correctly compared to those in the control arm (45% vs. 34%, p=0.03).

ANC providers' confidence, self-efficacy, and communication skills

A higher proportion of ANC providers in the intervention arm reported being confident in their knowledge to provide FGM prevention and care services compared to those in the control arm (98% vs. 89%, p=0.005). In multivariable analysis, ANC providers in the intervention arm had more than six

For peer review only - http://bmjopen.bmj.com/site/about/guidelines.xhtml

times the odds of reporting being confident in their knowledge to provide FGM prevention and care services compared to those in the control arm (AOR: 6.3, 95% CI: 1.4-28.9; p=0.02). Self-efficacy was generally high (scores 7.4 – 7.8 out of 8) with no significant difference in high scores between study arms (85% vs. 82%, p=0.36 and OR: 0.8, 95% CI: 0.4-1.6); p= 0.50).

ANC providers' knowledge, attitudes and support for FGM/medicalized FGM

The mean correct scores for FGM-related knowledge were higher among ANC providers in the intervention arm compared to the control arm (2.5, 95% CI: 2.2-2.8 vs. 1.9, 95% CI: 1.7-2.2; p=0.005) but 8% vs. 2% (p=0.16) had correct responses on the FGM-related knowledge questions, showing low knowledge overall, and particularly on the FGM typology. Providers had similarly unsupportive attitudes towards FGM in both groups and similarly unsupportive attitudes about medicalized FGM with most providers reporting that they did not support FGM (82% vs. 85%, p=0.73) and/or medicalized FGM (72% vs. 73, p=0.94%).

ANC clients' support for FGM, intention to have their daughters undergo FGM and being involved in FGM prevention efforts

Compared to those in the control arm, a higher proportion of ANC clients in the intervention arm reported being less supportive of FGM after their month six clinic visit (52% vs. 29%, p<0.001). In multivariable analysis, ANC clients in the intervention arm had more than twice the odds of reporting that they were strongly opposed to FGM (AOR: 2.4, 95% CI: 1.1-5.2; p=0.023, ICC: 0.61). When asked about their support for FGM after the ANC visit compared to before, clients in the intervention arm had more than five times the odds of being less supportive of FGM compared to those in the control arm (OR: 5.4, 95% CI: 2.4-12.4; p<0.001, ICC:0.66). ANC clients in the intervention clinics had lower odds of intending to have their daughters undergo FGM (OR: 0.3, 95% CI: 0.1-0.7; p=0.004, ICC: 0.60) or of wanting a health care provider to perform FGM (OR: 0.2, 95% CI: 0.1-0.5; p<0.001, ICC: 0.54) and

For peer review only - http://bmjopen.bmj.com/site/about/guidelines.xhtml

higher odds of reporting that they wished to be active in FGM prevention (OR: 3.2, 95% CI: 1.6-6.2, p=0.001, ICC: 0.50).

To understand the impact of the level one intervention relative to the level two intervention, a comparison of study outcomes restricted to the intervention arm was done between baseline and month three and between months three and six (Annex 3). Although not statistically powered for this analyses, we found that a significantly higher proportion of ANC clients in the intervention arm reported that their provider had asked about the different PCC components at month three versus basleine and at month six versus month three. Similarly, a significantly higher proportion of ANC clinics in the intervention arm were prepared to provide FGM-related prevention and care services at month three compared to baseline and at month six compared to month three. No statistically significant differences were seen in the proportion of ANC providers with the secondary outcomes apart from high confidence scores seen between month six and month three. Finally, ANC client outcomes were significantly higher among intervention clients in month three versus baseline and in month six versus month three.

DISCUSSION

The results of this cluster randomized trial show that an intervention to strengthen health facility preparedness while building skills of ANC providers to communicate using a person-centred counselling technique on FGM prevention was effective. ANC providers exposed to the intervention had increased confidence, improved FGM-related knowledge, and effective delivery of FGM prevention and care services. Additionally, ANC clients who had received care from these providers were less supportive of FGM and had reduced intentions to perform FGM on their daughters. This study provides evidence of a practical intervention to engage health care providers in FGM abandonment efforts whilst also providing quality care to FGM survivors. This study provides evidence of how to effectively build the capacity of

For peer review only - http://bmjopen.bmj.com/site/about/guidelines.xhtml

health care providers at primary care to address FGM(26), an area identified as a critical gap during the formative research.

The PCC training modules strengthened ANC providers' skills on FGM prevention and care and helped to clarify their beliefs and attitudes, which are key drivers of FGM (27). We did not find notable changes in knowledge and attitudes among ANC providers. The knowledge scores overall were low, and upon further investigation, it appears that questions on typology captured through visually drawn images on a tablet device were consistently answered incorrectly. These results perhaps show measurement and knowledge limitations but do not necessarily relate to service provision or quality of care. Attitudes in the intervention and control groups were generally unsupportive of FGM and do not appear to be heavily impacted by the training intervention. Exposure to the intervention package also did not improve ANC providers' self-efficacy towards FGM prevention and care. This may be related to the lack of support for FGM and/or its medicalization and high self-efficacy among nearly all providers throughout the study in both study arms, a finding that was also noted in formative research conducted in Guinea (28,29). In the formative phase, while the vast majority of health workers were opposed to the practice, 38% also felt that FGM limited promiscuity and 7% believed that it was a good practice, showing ambivalence and complexity in attitudes about FGM among health providers. Other studies have found that some providers support the perpetuation of the practice and even planned to have their own daughters undergo FGM or to perform it on their clients (30).

The findings in this study underscore the importance of addressing values and attitudes of both providers and clients as a means of achieving positive behavioral change. Changes observed among ANC providers were sustained across the study duration and ultimately, and importantly, resulted in reported changes in attitudes and intentions of their clients. However, this study design did not allow us

For peer review only - http://bmjopen.bmj.com/site/about/guidelines.xhtml

to determine whether the attitudinal changes observed among ANC clients were sustained after their clinic visit or translated into positive change in FGM prevention.

The application of these study results into programming will need to consider several factors. Firstly, the study sites were primary care facilities located in high FGM prevalence settings. The results of this intervention may not be generalizable to settings where FGM is less prevalent or to settings other than primary care. Secondly, first ANC visits are not typical of other health visits since the consultation is generally longer with a greater focus on health promotion messaging. While this is an ideal setting for implementing such an intervention, its application to other health settings and among other population groups is not known. During scale up, if the PCC approach is applied among clients seeking other sexual and reproductive health services or parents bringing their children to child immunization and wellness visits, it will be important to consider time requirements for the delivery of the 'ABCD' steps, especially in high volume clinic settings.

Thirdly, while the study found a positive impact of the PCC training on health care providers' delivery of person-centred FGM prevention counselling, the continuity and quality of FGM prevention counselling in the long-term is not known. Specifically, it will be important to assess subsequently whether providers will continue to provide prevention counselling on an ongoing basis, whether they will share their learnings with family and community members and whether clients will follow through with their intentions to not have their daughters undergo FGM. It may be important to include a supervisory mentorship component to ensure implementation of this intervention (31) in order to strengthen PCC communication practice and quality.

Limitations

For peer review only - http://bmjopen.bmj.com/site/about/guidelines.xhtml

The implementation of this multi-country study was not without challenges and limitations. First, initiation of field data collection activities was delayed by the global COVID-19 pandemic in 2020 – 2021 and required some modification to trainings of the data collection teams, the master trainers and the ANC providers receiving the PCC intervention. This may have impacted the overall effectiveness of the intervention.

Second, to attempt to ensure participation of at least one provider at each site, all providers were pre-screened at baseline and clinic rotation schedules determined enrollment into the study. Selection bias might have been introduced through this process. The exploratory analysis to assess for selection and attrition bias from the pre-screen step, did not reveal significant differences between included and excluded health workers except for slightly lower age (Supplementary file 4), and a per protocol analysis was required, but it is possible that differences in other unmeasured factors related to the clinics and providers might have biased the results. Findings from a process evaluation conducted as part of this study will provide additional insights on the feasibility, acceptability, appropriateness, and fidelity of the intervention implementation in these contextual settings to inform further implementation and scale up.

Third, we did not perform adjustment for multiple testing in our analysis given that the different tests are interpreted separately and no overall conclusion will be stated. Given that the null hypotheses of no differences are true, we estimate that the overall type one error rate is higher than the individual test level of 0.05.

Finally, we acknowledge that there are many factors that could impact FGM-related decision-making and a positive and impactful interaction with a respected health care provider might not be sufficient to lead to actual changes in community behavior. However, the study design enabled us to compare similar sites to identify the relative effect of this approach since both intervention and control

For peer review only - http://bmjopen.bmj.com/site/about/guidelines.xhtml

sites would be exposed to similar factors, and clients at these sites would face similar complexities in decision-making.

Conclusion

In conclusion, this study highlights the importance of addressing the values and beliefs of health care providers working at primary care level, who are subject to social norms around FGM that may conflict with medical ethics and national laws and policies as an intermediary step in preventing FGM. Empowering these health care providers with communication skills and engaging them as opinion leaders can be impactful in changing their clients' attitudes towards FGM. In conjunction with FGM prevention activities in other sectors, this intervention can contribute to positive change if brought to scale.

For peer review only

For peer review only - http://bmjopen.bmj.com/site/about/guidelines.xhtml

DECLARATIONS

Contributors

WA and CP conceptualized the study and prepared the protocol in collaboration with VM, KS, PN, TE, MDB, AMS, AD(1) and MAA. MDB, AMS, AOS, PN, TE, JMK, AD(1) and MAA provided oversight over study implementation while AD(2), JK and SA monitored data quality in countries and KN and SST monitored data quality across countries. VM prepared the first draft of the manuscript with input from WA and CP, the responsible officer of the study at WHO/HRP. MP developed the statistical analysis plan and conducted data analysis. KS coordinated the development of the PCC for FGM prevention training. KS, PN, TE, JMK, JK, MDB, AMS, AOS, AD(1), AD(2), SA, and MAA contributed to and reviewed the manuscript for proper intellectual content. All authors read and approved the final draft of this manuscript.

Declaration of interests

The authors declare that they have no competing interests.

Data sharing

De-identified dataset will be retained in the WHO HRP electronic archival system. Any use of the de-identified analytic dataset for secondary research purposes will be governed by the WHO data use regulation. Request for data dictionary and for dataset may be sent to pallittoc@who.int

Acknowledgements

The authors would like to acknowledge the funders, the WHO country office colleagues, Dr Bernadette Dramou, Dr Cécé-Vieux Kolie, Dr Joyce Lavussa, Ms Matilda Cherono, Ms Asia Ahmed Osman in the study countries as well as MoH and national FGM stakeholders in the development of the research protocol, implementation of study interventions and field data collection. We also thank Professor Joanna Schellenberg of the London School of Hygiene and Tropical Medicine and Dr Christina

For peer review only - http://bmjopen.bmj.com/site/about/guidelines.xhtml

Atchison of Imperial College London for their input in conceptualizing the study as well as Dr Leyla Hussein for supporting pilot testing of the PCC intervention.

Funding statement

This work received funding from the Governments of Norway and the United Kingdom of Great Britain and Northern Ireland as well as the UNDP-UNFPA-UNICEF-WHO-World Bank Special Programme of Research, Development and Research Training in Human Reproduction (HRP), a cosponsored programme executed by the World Health Organization (WHO).

Ethics approval:

The following research ethics committees approved the protocol:

1. World Health Organization (WHO) Ethical Review Committee (ERC) (#P151/03/2014).

2. Kenya: Kenyatta National Hospital/University of Nairobi ERC (P805/09/2019) and the National Commission for Science, Technology, and Innovation (NACOSTI/P/20/5721)

3. Somalia: the Department of Planning, Policy and Strategic Information, Unit of Research (MOHD/DG: 2/11526/2019)

4. Guinea: the Comité National d'Ethique Pour la Recherche en Santé (CNERS) (105/CNERS/19).

Disclaimer

The named authors alone are responsible for the views expressed in this publication and do not necessarily represent the decisions or the policies of the UNDP-UNFPA-UNICEF-WHO-World Bank Special Programme of Research, Development and Research Training in Human Reproduction (HRP) or the World Health Organization (WHO).

For peer review only - http://bmjopen.bmj.com/site/about/guidelines.xhtml

For peer review only

For peer review only - http://bmjopen.bmj.com/site/about/guidelines.xhtml

REFERENCES

1. United Nations General Assembly, Intensifying global efforts for the elimination of female genital mutilations : resolution/ / adopted by the United Nations General Assembly, 5 March 2013, A/RES/67/146, available at: https://www.refworld.org/docid/51e67bc64.html [accessed 4 May 2022]
2. World Health Assembly, WHA resolution 61.16. 2008. United Nations: New York. 2008. Available at https://apps.who.int/gb/ebwha/pdf_files/WHA61-REC1/A61_REC1-en.pdf , New York. 2008.
3. Eliminating female genital mutilation: an interagency statement-OHCHR, UNAIDS, UNDP, UNECA, UNESCO, UNFPA, UNHCR, UNICEF, UNIFEM, WHO. WHO: Geneva.World Health Organization, 2008.
4. World Health Organization. The World Health Report: 2000 :health systems : improving performance [Internet]. Geneva: World Health Organization; 2000. Available from: https://apps.who.int/iris/handle/10665/42281
5. Pallitto CC, Ahmed W. The role of the health sector in contributing to the abandonment of female genital mutilation. Med (N Y) [Internet]. 2021 May 14 [cited 2023 May 15];2(5):485–9. Available from: https://pubmed.ncbi.nlm.nih.gov/35590230/
6. Kimani S, Esho T, Kimani V, Muniu S, Kamau J, Kigondu C, et al. Female Genital Mutilation/Cutting: Innovative Training Approach for Nurse-Midwives in High Prevalent Settings. Obstet Gynecol Int [Internet]. 2018 [cited 2023 May 15];2018. Available from: https://pubmed.ncbi.nlm.nih.gov/29736171/
7. Johansen REB, Diop NJ, Laverack G, Leye E. What works and what does not: a discussion of popular approaches for the abandonment of female genital mutilation. Obstet Gynecol Int [Internet]. 2013 [cited 2023 May 15];2013:1–10. Available from: https://pubmed.ncbi.nlm.nih.gov/23737795/
8. Njue C, Askew I. Medicalization of female genital cutting among the Abagusii in Nyanza Province, Kenya. Reprod Health [Internet]. 2004 Jan 1 [cited 2023 May 15]; Available from: https://knowledgecommons.popcouncil.org/departments_sbsr-rh/32
9. Shell-Duncan B, Gathara D, Moore Z. Female genital mutilation/cutting in Kenya: Is change taking place? Descriptive statistics from four waves of Demographic and Health Surveys. Reprod Health [Internet]. 2017 Jan 1 [cited 2023 May 15]; Available from: https://knowledgecommons.popcouncil.org/departments_sbsr-rh/596
10. Umar AS, Oche OM. Medicalization of female genital mutilation among professional health care workers in a referral hospital, north-western Nigeria. J Reprod Biol Health. 2014;2(1):2.
11. Doucet MH, Pallitto C, Groleau D. Understanding the motivations of health-care providers in performing female genital mutilation: an integrative review of the literature. Reprod Health [Internet]. 2017 Mar 23 [cited 2023 May 15];14(1):46. Available from: https://pubmed.ncbi.nlm.nih.gov/28335771/
12. World Health Organization. Female Genital Mutilation: A Resource Kitkit for the Health Sector. WHO: Geneva, 2021, available at https://srhr.org/fgmresources/https://srhr.org/fgmresources/ - Google Search [Internet]. [cited 2023 May 15].
13. World Health Organization. Reproductive Health and Research. WHO guidelines on the management of health complications from female genital mutilation. :47.
14. Ahmed W, Mochache V, Stein K, Ndavi P, Esho T, Balde MD, et al. A hybrid, effectiveness-implementation research study protocol targeting antenatal care providers to provide female

For peer review only - http://bmjopen.bmj.com/site/about/guidelines.xhtml

genital mutilation prevention and care services in Guinea, Kenya and Somalia. BMC Health Serv Res [Internet]. 2021 Dec 1 [cited 2023 May 15];21(1). Available from: https://pubmed.ncbi.nlm.nih.gov/33522926/
15. National Bureau of Statistics. Republic of Kenya. Kenya Demographic and Health Survey 2014. 2015 [cited 2023 May 15]; Available from: www.DHSprogram.com.
16. NHWA Web portal [Internet]. [cited 2023 May 15]. Available from: https://apps.who.int/nhwaportal/Home/Welcome?ReturnUrl=%2Fnhwaportal%2FHome%2FIndex
17. Boone Tim, Reilly Anthony J., Sashkin M. SOCIAL LEARNING THEORY Albert Bandura Englewood Cliffs, N.J.: Prentice-Hall, 1977. 247 pp., paperbound. Group & Organization Studies [Internet]. 1977 Sep 1;2(3):384–5. Available from: https://doi.org/10.1177/105960117700200317
18. Valente TW, Pumpuang P. Identifying opinion leaders to promote behavior change. Health Educ Behav [Internet]. 2007 Dec [cited 2023 May 15];34(6):881–96. Available from: https://pubmed.ncbi.nlm.nih.gov/17602096/
19. Person-centred communication for female genital mutilation prevention: facilitator's manual. WHO World Health Organization: Geneva, 2022
20. World Health Organization. WHO global strategy on people-centred and integrated health services: interim report [Internet]. Geneva: World Health Organization; 2015. Available from: https://apps.who.int/iris/handle/10665/155002

21. Peters DH, Adam T, Alonge O, Agyepong IA, Tran N. Republished research: Implementation research: what it is and how to do it. Br J Sports Med. 2014 Apr 23;48(8):731–6.
22. Schulz KF, Altman DG, Moher D. CONSORT 2010 statement: updated guidelines for reporting parallel group randomised trials. BMJ [Internet]. 2010 Mar 27 [cited 2023 May 15];340(7748):698–702. Available from: https://pubmed.ncbi.nlm.nih.gov/20332509/
23. Epstein RM, Franks P, Fiscella K, Shields CG, Meldrum SC, Kravitz RL, et al. Measuring patient-centered communication in patient-physician consultations: theoretical and practical issues. Soc Sci Med [Internet]. 2005 Oct [cited 2023 May 15];61(7):1516–28. Available from: https://pubmed.ncbi.nlm.nih.gov/16005784/
24. Chen G, Gully SM, Eden D. Validation of a New General Self-Efficacy Scale. Organ Res Methods [Internet]. 2001 Jan 1;4(1):62–83. Available from: https://doi.org/10.1177/109442810141004
25. Keeley RD, Burke BL, Brody D, Dimidjian S, Engel M, Emsermann C, et al. Training to use motivational interviewing techniques for depression: a cluster randomized trial. J Am Board Fam Med [Internet]. 2014 Sep 1 [cited 2023 May 15];27(5):621–36. Available from: https://pubmed.ncbi.nlm.nih.gov/25201932/
26. Balfour J, Abdulcadir J, Say L, Hindin MJ. Interventions for healthcare providers to improve treatment and prevention of female genital mutilation: a systematic review. BMC Health Serv Res [Internet]. 2016 Aug 19 [cited 2023 May 15];16(1). Available from: https://pubmed.ncbi.nlm.nih.gov/27542732/
27. Kimani S, Okondo C, Muteshi-Strachan J, Guyo J. Quality of services offered to women with female genital mutilation across health facilities in a Kenyan County. BMC Health Serv Res [Internet]. 2022 Dec 1 [cited 2023 May 15];22(1). Available from: https://pubmed.ncbi.nlm.nih.gov/35525954/
28. Balde MD, O'Neill S, Sall AO, Balde MB, Soumah AM, Diallo BA, et al. Attitudes of health care providers regarding female genital mutilation and its medicalization in Guinea. PLoS One

For peer review only

[Internet]. 2021 May 1 [cited 2023 May 15];16(5). Available from: https://pubmed.ncbi.nlm.nih.gov/33983949/

29. Balde MD, Soumah AM, Diallo A, Sall AO, Mochache V, Ahmed W, et al. Involving the health sector in the prevention and care of female genital mutilation: results from formative research in Guinea. Reprod Health. 2022 Dec 1;19(1).

30. Kaplan A, Hechavarría S, Bernal M, Bonhoure I. Knowledge, attitudes and practices of female genital mutilation/cutting among health care professionals in The Gambia: a multiethnic study. BMC Public Health [Internet]. 2013 [cited 2023 May 15];13(1). Available from: https://pubmed.ncbi.nlm.nih.gov/24040762/

31. Pantoja T, Opiyo N, Lewin S, Paulsen E, Ciapponi A, Wiysonge CS, et al. Implementation strategies for health systems in low-income countries: an overview of systematic reviews. Cochrane Database Syst Rev [Internet]. 2017 Sep 12 [cited 2023 May 15];9(9). Available from: https://pubmed.ncbi.nlm.nih.gov/28895659/

For peer review only

For peer review only - http://bmjopen.bmj.com/site/about/guidelines.xhtml

TABLES & FIGURES:

Figure 1: Study CONSORT Diagram

Table 1: Characteristics of ANC clinics included in month six analyses

Table 2: Characteristics of ANC providers included in the month six analyses

Table 3: Characteristics of ANC clients interviewed at each time point

Table 4: Analysis of study outcomes

SUPPLEMENTARY FILES

Supplementary file 1: Theory of change framework

Supplementary file 2: Measurement of study outcomes

Supplementary file 3: Data collection instruments

Supplementary file 4: Additional analyses (Appendices 1 – 3)

For peer review only

For peer review only - http://bmjopen.bmj.com/site/about/guidelines.xhtml

Table 1: Characteristics of ANC clinics included in month six analyses

Characteristics	Overall (n=163*)	Intervention (n=82)	Control (n=81)
Number of ANC providers	Mean 4 (SD: 3) Median 3 (1-14, IQR 3)	Mean 4 (SD: 2) Median 3 (1-11, IQR 3)	Mean 4 (SD: 3) Median 3 (1-14, IQR 4)
Average number of ANC clients/month	Mean 150 (SD: 127) Median 118 (3-664, IQR 141)	Mean 148 (SD: 121) Median 117 (3-500, IQR 143)	Mean 152 (SD: 133) Median 120 (3-664, IQR 140)
MoH supervisory visits in the past year	Mean 4 (SD: 3) Median 3 (0-18, IQR 2)	Mean 4 (SD: 3) Median 4 (1-18, IQR 1)	Mean 4 (SD: 3) Median 3 (0-12, IQR 2
Size of catchment population served	Mean 36,754 (SD: 126,082) Median 15,972 (1,000-1,458,000, IQR 24,332)	Mean 23,649 (SD: 35,873) Median 16,022 (1,000-290,000, IQR 22,361	Mean 50,020 (SD: 174,739) Median 15,551 (1,000-1,458,000, IQR 25,544
Presence of anti-FGM activities in the catchment area			
Yes	74 (45%)	43 (52%)	31 (38%)
No	89 (55%)	39 (48%)	50 (62%)
Presence of pro-FGM activities in the catchment area			
Yes	21 (13%)	12 (15%)	9 (11%)
No	140 (86%)	68 (83%)	72 (89%)
Don't Know	2 (1%)	2 (2%)	0 (0%)

* Total of 17 ANC clinics not included: 16 clinics were excluded (7 intervention and 9 control) due to loss-to-follow up (LTFU) of ANC provider i.e., the clinics did not have at least one ANC provider present across all study time points while one ANC clinic in Kenya was never visited at subsequent time points due to issues with insecurity. An ANC provider from one of the clinics in Kenya that had been inaccessible due to insecurity attended the PCC training and was subsequently interviewed.

For peer review only

For peer review only - http://bmjopen.bmj.com/site/about/guidelines.xhtml

Table 2: Characteristics of ANC providers included in the month six analyses

Characteristics	Overall (n=232)	Intervention (n= 115)	Control (n=117)
Age	Mean 36 (SD: 10) Median 34 (20-65, IQR 15)	Mean 35 (SD: 10) Median 33 (20-59, IQR 14)	Mean 37 (SD:11) Median 35 (20-65, IQR 16)
Years of professional experience	Mean 8 (SD: 7) Median 6 (1-39, IQR 7)	Mean 8 (SD:7) Median 6 (1-30, IQR 8)	Mean 8 (SD:7) Median 6 (1-39, IQR 7)
Sex			
Female	193 (83%)	95 (83%)	98 (84%)
Highest educational level			
Certificate	21 (5%)	12 (10%)	9 (8%)
Diploma	158 (68%)	72 (63%)	86 (74%)
Bachelors	44 (19%)	27 (24%)	17 (15%)
Masters & above	1 (0.4%)	0 (0%)	1 (1%)
Other[#]	8 (3%)	4 (3%)	4 (3%)
Current professional role/title			
Midwife	103 (44%)	53 (46%)	50 (43%)
Nurse	51 (22%)	25 (22%)	26 (22%)
Nurse-Midwife	54 (23%)	27 (24%)	27 (23%)
Other	24 (10%)	10 (9%)	14 (12%)
Received formal training on FGM during clinical training			
Yes	85 (37%)	44 (38%)	41 (35%)
No	146 (63%)	71 (62%)	75 (64%)
Don't Know	1 (0.4%)	0 (0%)	1 (1%)
Timing of clinical training on FGM			
Pre-service	33 (14%)	18 (16%)	15 (13%)
In-service	45 (19%)	22 (19%)	23 (20%)
Both pre- and in-service	7 (3%)	4 (4%)	3 (3%)
Received formal training on communication/counselling			
Yes	149 (64%)	76 (66%)	73 (62%)
No	83 (36%)	39 (34%)	44 (38%)
Received formal training on person-centered care			
Yes	118 (51%)	58 (50%)	60 (51%)
No	113 (56%)	56 (49%)	57 (49%)
Don't know	1 (0.4%)	1 (1%)	0 (0%)
Undergone FGM			

For peer review only

For peer review only - http://bmjopen.bmj.com/site/about/guidelines.xhtml

Yes	126 (54%)	65 (57%)	61 (52%)
No	63 (27%)	27 (24%)	36 (31%)
Don't know	2 (1%)	2 (2%)	0 (0%)
Refused to answer	2 (1%)	1 (1%)	1 (1%)
Conducted FGM			
Yes	15 (7%)	9 (8%)	6 (5%)
Conducted FGM on a girl <18 years			
Yes	14 (6%)	8 (7%)	6 (5%)

For peer review only

For peer review only - http://bmjopen.bmj.com/site/about/guidelines.xhtml

Table 3: Characteristics of ANC clients interviewed at each time point

Characteristics	ANC clients interviewed at Baseline			ANC clients interviewed at Month 3	ANC clients interviewed at Month 6		
	**Overall (n=1800)**	**Intervention (n=900)**	**Control (n=900)**	**Intervention only (n=880)**	**Overall (n=1759)**	**Intervention (n=879)**	**Control (n=880)**
Age	Mean 26 (SD: 6) Median 25 (15-45, IQR 10)	Mean 25 (SD: 6) Median 25 (15-45, IQR 10)	Mean 26 (SD: 6) Median 25 (15-45, IQR 10)	Mean 26 (SD 6) Median 25 (15-45, IQR 10)	Mean 26 (SD: 6) Median 25 (15-45, IQR 9)	Mean 26 (SD: 6) Median 25 (15-45, IQR 9)	Mean 26 (SD: 6) Median 25 (15-45, IQR 10)
Highest educational level							
None	840 (47%)	407 (45%)	433 (48%)	439 (50%)	806 (46%)	384 (44%)	422 (47%)
Primary	484 (27%)	231 (26%)	253 (28%)	239 (27%)	553 (31%)	278 (32%)	275 (31%)
Secondary	331 (18%)	171 (19%)	160 (18%)	157 (18%)	306 (17%)	160 (18%)	146 (16%)
University	95 (5%)	61 (7%)	34 (4%)	25 (3%)	67 (4%)	34 (4%)	33 (4%)
Other[#]	50 (3%)	30 (3%)	20 (25)	20 (2%)	37 (2%)	23 (3%)	14 (2%)
Have you undergone FGM?							
Yes	1320 (73%)	677 (75%)	643 (71%)	645 (73%)	1321 (75%)	655 (75%)	666 (75%)
No	452 (25%)	209 (23%)	243 (27%)	224 (25%)	420 (24%)	206 (23%)	214 (24%)
Don't know	12 (1%)	10 (1%)	2 (0.2%)	5 (1%)	21 (1%)	13 (2%)	8 (1%)
Refused to answer	16 (1%)	4 (0.4%)	12 (1%)	6 (1%)	7 (0.4%)	5 (1%)	2 (0.2%)

For peer review only - http://bmjopen.bmj.com/site/about/guidelines.xhtml

For peer review only

For peer review only - http://bmjopen.bmj.com/site/about/guidelines.xhtml

Table 4: Analysis of study outcomes

**Primary Outcomes**					
**ANC clients reporting that their provider implemented components of PCC for FGM prevention approach**					
	**Intervention (n=819)**	**Control (n=810)**	**Adjusted OR# (95% CI)**	**P-value**	**ICC**
Provider asked client if they have undergone FGM	634 (77%)	245 (30%)	8.9 (6.9-11.5)	<0.001	N/A
Provider asked client about the client's personal beliefs regarding FGM	616 (75%)	217 (27%)	9.7 (7.5-12.5)	<0.001	N/A
Provider discussed with client why FGM should be prevented	629 (77%)	244 (30%)	9.2 (7.1-11.9)	<0.001	N/A
Provider discussed with client how FGM could be prevented	592 (72%)	232 (29%)	7.7 (6.0-9.9)	<0.001	N/A
Client satisfied with how FGM was addressed by provider during clinic visit	684 (84%)	348 (43%)	6.6 (5.1-8.4)	<0.001	N/A
			Difference in mean scores (95% CI)		
Mean score of implementing PCC approach (out of 5)	3.9 (3.8-4.0)	1.6 (1.5-1.7)	2.3 (2.1-2.5)	<0.001	N/A
Mean score of PCC + appropriate FGM prevention and care (out of 8)	6.2 (5.9-6.6)	3.7 (3.2-4.1)	2.6 (2.0-3.2)	<0.001	N/A
**ANC clinic preparedness to offer FGM prevention and care services**					
	**Intervention (n=82)**	**Control (n=81)**	**Adjusted OR[&] (95% CI)**	**P-value**	**ICC**
Clinics with ALL correct responses for preparedness	56 (68%)	22 (27%)	-	<0.001	N/A
Mean score of clinic preparedness (out of 4)	3.4 (3.2-3.6)	2.6 (2.4-2.9)	-	<0.001	N/A
	**Intervention (n=115)**	**Control (n=117)**	**Adjusted OR[&] (95% CI)**	**P-value**	**ICC**
Providers using level 1 intervention package	96 (83%)	65 (56%)	9.3 (4.2-20.8)	<0.001	N/A
**Secondary Outcomes***					
Providers with appropriate interpersonal communication skills	74 (64%)	68 (58%)	1.7 (1.0-3.0)	0.060	N/A
Providers with high self-efficacy	86 (75%)	99 (85%)	0.8 (0.4-1.6)	0.453	N/A
Providers reporting less supportive attitudes towards FGM	76 (66%)	85 (73%)	1.0 (0.5-1.8)	0.901	N/A
Providers with high confidence scores	103 (90%)	104 (89%)	6.3 (1.4-28.9)	0.018	N/A
Providers not supportive of FGM	100 (87%)	114 (97%)	0.8 (0.2-3.7)	0.726	N/A
Providers not supportive of medicalized FGM	104 (90%)	116 (99%)	1.1 (0.1-22.1)	0.938	N/A
Providers with correct FGM-related knowledge responses	8 (8%)	1 (2%)	5.0 (0.5-47.8)	0.16	N/A
Mean score of FGM-related knowledge (out of 6)	2.5 (2.2-2.8)	1.9 (1.7-2.2)	-	0.005	N/A
**Other ANC client outcomes****					
	**Intervention (n=819)**	**Control (n=810)**	**Adjusted OR[&] (95% CI)**	**P-value**	**ICC**
Clients reporting less support for FGM after ANC clinic visit	424 (52%)	237 (29%)	5.4 (2.4-12.4)	<0.001	0.66
Clients reporting that they were strongly opposed to FGM	498 (61%)	382 (47%)	2.4 (1.1-5.2)	0.023	0.62

For peer review only - http://bmjopen.bmj.com/site/about/guidelines.xhtml

Clients reporting that they intend to have their daughters cut	96 (12%)	209 (26%)	0.3 (0.1-0.7)	0.004	0.60
Clients reporting that they would prefer health care provider to cut daughters	53 (7%)	139 (17%)	0.2 (0.1-0.5)	<0.001	0.54
Clients wishing to be active in FGM prevention	677 (83%)	535 (66%)	3.2 (1.6-6.2)	0.001	0.50

ICC = Intra-cluster Correlation Coefficient
[#]Single-level multi-variable adjusted models
[&]Multi-level multi-variable adjusted models
*Provider outcomes adjusted for sex, years of service, FGM status, FGM-related training, any specific training on communication/counseling and PCC, and whether the provider had conducted FGM in the past
** Client outcomes adjusted for age, educational level, FGM status and exposure to level one IEC materials

For peer review only

For peer review only - http://bmjopen.bmj.com/site/about/guidelines.xhtml

For peer review only

For peer review only - http://bmjopen.bmj.com/site/about/guidelines.xhtml

Figure 1: CONSORT diagram

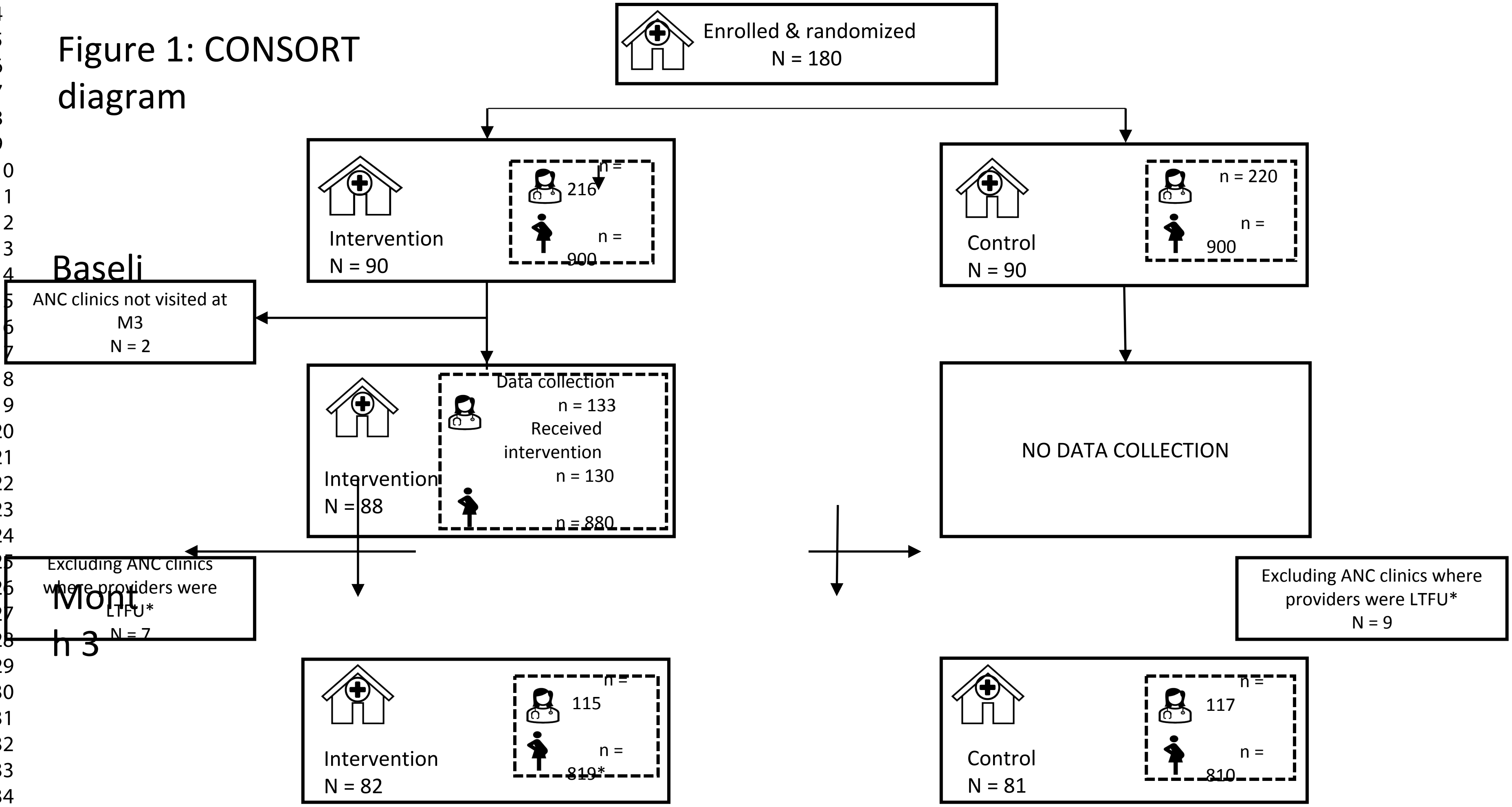

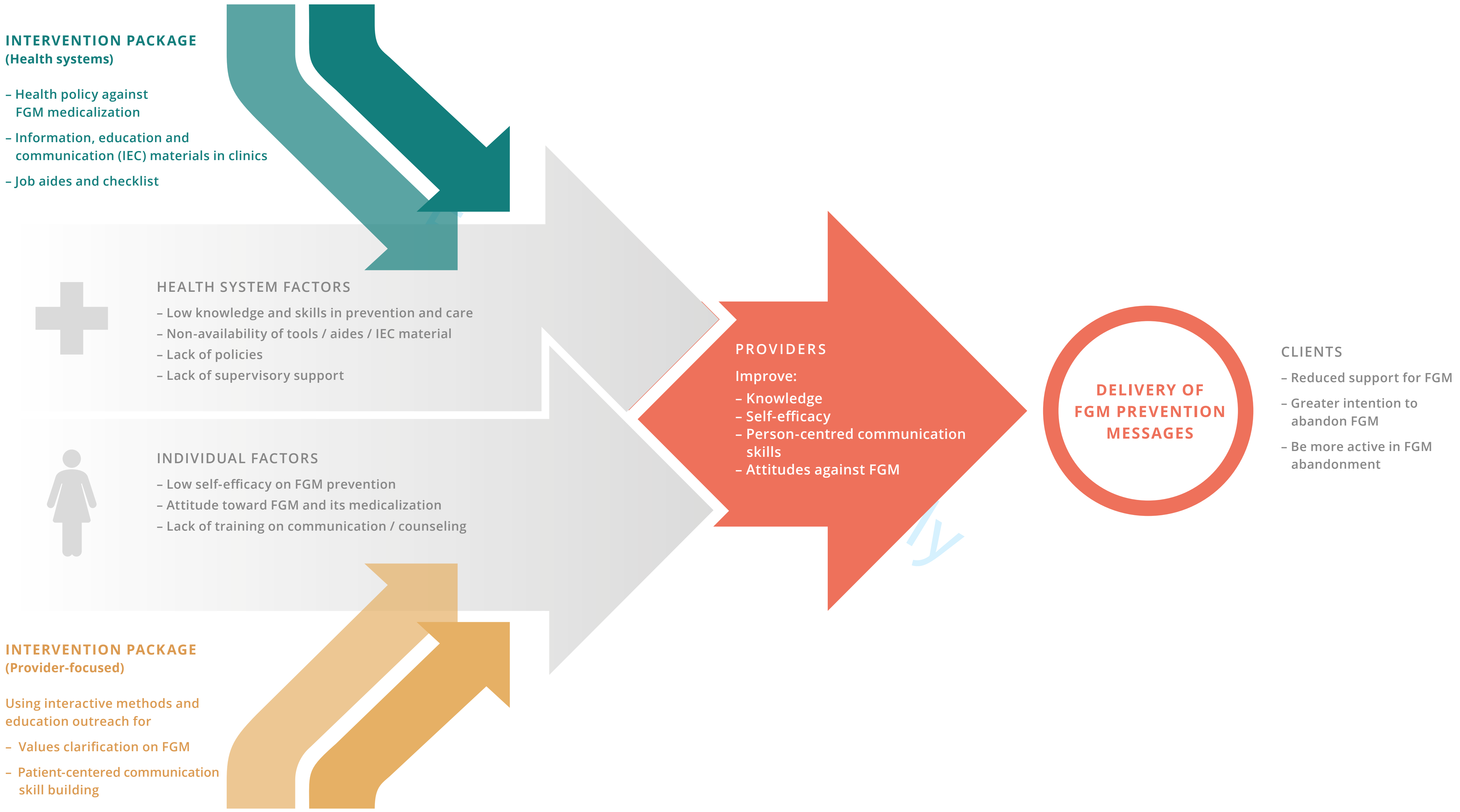
INTERVENTION PACKAGE
(Health systems)
– Health policy against FGM medicalization
– Information, education and communication (IEC) materials in clinics
– Job aides and checklist
HEALTH SYSTEM FACTORS
– Low knowledge and skills in prevention and care
– Non-availability of tools / aides / IEC material
– Lack of policies
– Lack of supervisory support
INDIVIDUAL FACTORS
– Low self-efficacy on FGM prevention
– Attitude toward FGM and its medicalization
– Lack of training on communication / counseling
INTERVENTION PACKAGE
(Provider-focused)
Using interactive methods and education outreach for
– Values clarification on FGM
– Patient-centered communication skill building
PROVIDERS
Improve:
– Knowledge
– Self-efficacy
– Person-centred communication skills
– Attitudes against FGM
DELIVERY OF FGM PREVENTION MESSAGES
CLIENTS
– Reduced support for FGM
– Greater intention to abandon FGM
– Be more active in FGM abandonment

Supplementary file 2 : Measurement of study outcomes

1. ***Primary Outcome:*** *Health facility preparedness to provide FGM prevention and care services.*

Outcome definition*: Cumulative score based on affirmative responses to Q9a, Q10a, Q11a & Q12a on the CHK form (see below).*

Q9. Is there an MoH policy on FGM posted on the wall?
Yes
No
Q9a. If yes, is it placed where health care providers can see/read it e.g., bulletin board?
Yes
No
Q10. Are there WHO FGM prevention posters on the wall of the consultation room and/or waiting room?
Yes
No
Q10a. If yes, are they placed in a place where ANC clients can see them?
Yes
No
Q11. Is there a WHO clinical handbook in the ANC consultation room?
Yes
No
Q11a If yes, is it placed where ANC providers can see/use it?
Yes
No
Q12. Is there an FGM ABCD guide in the ANC consultation room?
Yes
No
Q12a. If yes, is it placed where ANC providers can see/use it?
Yes
No

2. ***Primary outcome: ANC provider utilization of Level 1 package components***

Outcome definition*: Affirmative response on Q40 of HCP form (see below).*

Q40. Have you referred to the WHO Clinical Handbook on FGM?
Yes
No, available but not referred
No, not available
Don't know

3. ***Primary outcome: Provision of FGM-related care after PCC training***

Outcome definition: *Cumulative score based on affirmative responses (Provision of FGM-related care (after PCC training) either 'Always' or 'Often') on Q22, Q24 & Q25 on the HCP form (see below).*

Q22. How often do you discourage a pregnant woman expecting to have a girl, or one having a girl at the age of cutting, from having her daughter cut?
Always
Often
Sometimes
Rarely

For peer review only

Never
Rarely
Refused to answer

Q24. How often do you look for female genital mutilation when performing a gynecological examination of the vulva?

Always
Often
Sometimes
Rarely
Never
Rarely
Refused to answer

Q25. How often do you record female genital mutilation in the woman's medical file if you are aware that she has undergone FGM?

Always
Often
Sometimes
Rarely
Never
Rarely
Refused to answer

4. Primary Outcome: Delivery of PCC 'ABCD' package

Outcome definition: *Cumulative score based on affirmative responses on Q5, Q7, Q8, Q9 & Q12 on the EXT form.*

Q5. Did the ANC provider ask if you have undergone FGM?

Yes
No
Don't know
Refused

Q7. Did the ANC provider ask about your personal belief regarding FGM?

Yes
No
Don't know
Refused

Q8. Did the ANC provider discuss why FGM should be prevented?

Yes
No
Don't know
Refused

Q9. Did the ANC provider discuss how FGM could be prevented?

Yes
No
Don't know
Refused

Q12. Are you satisfied with how FGM was addressed during your visit with your ANC provider today?

Yes
No
Don't know
Refused

For peer review only

5. Secondary Outcome: Improved knowledge about FGM

Outcome definition: *Cumulative score based on correct responses to Q4 + affirmative responses to Q5 & Q7 of the HCP form.*

Q4. Please provide the WHO classification for the following images

Type I
Type II
Type III
Type IV
Don't Know
Other

Q5. Do you know of any health complications arising from female genital mutilation?

Yes
No

Q7. Are you aware of any existing WHO tools/guidance on FGM prevention and care?

Yes
No

6. Secondary Outcome: Improved interpersonal communication skills

Outcome definition: *Cumulative score based on positive responses ("Always or Often') to Q34, Q35, Q36, Q37, Q38 on the HCP form.*

Now I will ask you about your communication skills

34. I can put myself in others shoes

Always
Often
Sometimes
Rarely
Never
Rarely
Refused to answer

35. I let others know that I understand what they say

Always
Often
Sometimes
Rarely
Never
Rarely
Refused to answer

36. In conversations with my colleagues, I perceive not only what they say but what they don't say

Always
Often
Sometimes
Rarely
Never
Rarely
Refused to answer

37. I communicate effectively

Always
Often

For peer review only

Sometimes
Rarely
Never
Rarely
Refused to answer

38. *I communicate with others as though they are my equals*
Always
Often
Sometimes
Rarely
Never
Rarely
Refused to answer

7. *Secondary outcome: Improved self-efficacy*

Outcome definition: *Cumulative score based on positive responses (Agree or Strongly Agree) to Q26, Q27, Q28, Q29, Q30, Q31, Q32, Q33 on the HCP form.*

Now I would like to ask you a few questions about how you solve problems that you face. Please tell me how much you agree or disagree with the statements that I read to you

1 = Strongly disagree
2 = Disagree
3 = Neither agree nor disagree
4 = Agree
5 = Strongly agree

Q26. I will be able to achieve most of the goals that I have set for myself
Q27. When facing difficult tasks, I am certain that I will accomplish them
Q28. In general, I think that I can obtain outcomes that are important to me
Q29. I believe that I can succeed at almost any endeavor to which I set my mind
Q30. I will be able to successfully overcome many challenges
Q31. I am confident that I can perform effectively on many different tasks
Q32. Compared to other people, I can do most tasks very well
Q33. Even when things are tough, I can perform quite well

8. *Secondary outcome: Improved attitudes towards FGM*

Outcome definition*: Cumulative score based on positive responses to Q12, Q13, Q14, Q15, Q16, Q17, Q18 & Q19 on the HCP form.*

For each of the following statements please state if you:

1=Agree
2=Disagree
3=Don't know
4=Refused to answer

Q12. A girl who has not undergone FGM is unclean
Q13. A girl who has not undergone FGM cannot be married within her community
Q14. A girl who has not undergone FGM is a disgrace to her family's honor
Q15. Health care providers who provide FGM are violating FGM
Q16. Health care providers who provide FGM should be punished
Q17. FGM is a good practice
Q18. FGM is a violation of women and girls' rights
Q19. FGM is religious mandate

For peer review only

9. Tertiary outcome: ANC provider confidence in FGM knowledge to provide care

Outcome definition: *Positive responses ('Somewhat Confident' or 'Confident') to Q8 & Q9 on the HCP form*

Q8. When you treat or attend to a girl or woman with female genital mutilation, how confident are you that you have enough knowledge to provide good quality care?

1=Not confident
2=Somewhat confident
3=Confident
4=Refused to answer

Q9. How confident are you in your knowledge to communicate on FGM prevention?

1=Not confident
2=Somewhat confident
3=Confident
4=Refused to answer

10. Tertiary outcome: ANC provider support for FGM

Outcome definition: *Positive response ('Do not intend to cut her') to Q20 on the HCP form*

Q20. Pretend you had a daughter now who was at an age when cutting occurs, what would your intention to cut her be?

1=Intend to cut her
2=Do not intend to cut her
3=Don't know
4=Refused to answer

11. Tertiary outcome: ANC provider support for medicalized FGM

Outcome definition: *Correct response ('No') to Q21 on HCP form*

Q21. If a family brought their daughter to the clinic requesting genital cutting, for non-health reasons, would you perform it?

1=Yes
2=No
3=Don't know
4=Refused to answer

12. Tertiary outcome: ANC client change in support for FGM after ANC visit

Outcome definition: *Response to Q13 on EXT form*

Q13. What do you feel about FGM now as compared to before you came to the clinic today?

1= Same, no change
2=I feel more supportive of FGM now as compared to before I came
3=I feel less supportive of FGM now as compared to before I came
4=Don't know
5=Other
6=Refused to answer

13. Tertiary outcome: ANC client support or opposition to FGM

Outcome definition: *Response to Q14 on EXT form*

Q14. How supportive are you of female genital mutilation?

1=Strongly opposed
2=Somewhat opposed

For peer review only

3=Neutral
4=Somewhat supportive
5=Strongly supportive
6=Refused to answer

14. Tertiary outcome: ANC client intention to cut after ANC visit.
Outcome definition: *Response to Q16 on EXT form*

Q.16 Pretend you had a daughter now who was at an age where cutting occurs, what would your intention to cut her be?

1=Intend to cut her
2=Do not intend to cut her
3=Don't know
4=Refused to answer

15. Tertiary outcome: ANC client choice of who to cut their daughters.
Outcome definition: *Response to Q17 on EXT form*

Q17. If intending to cut, who would you prefer to do the cutting?

1=Traditional practitioner
2=Health care provider
3=Other
4=Refused to answer

16. Tertiary outcome: ANC client wish to be active in FGM prevention
Outcome definition: *Response to Q18 on EXT form*

Q.18 Do you wish/want to be active in preventing FGM?

1=Yes
2=No
3=Don't know
4=Refused to answer

For peer review only

A HEALTH SYSTEMS APPROACH TO PREVENTION OF FEMALE GENITAL MUTILATION USING PERSON-CENTRED COMMUNICATION: IMPLEMENTATION RESEARCH PROJECT IN GUINEA, SOMALIA AND KENYA (A65993)

ANC PROVIDER SCREENING QUESTIONNAIRE (SCR)

Participant ID:

A	6	5	9	9	3

Project ID:

Country ID: Facility ID:

Instructions: Observe and report findings from the health facility.

1. **MoH policy on FGM posted on the wall?**
   ☐ Yes
   ☐ No
   1a. If yes, is it placed where health care providers can see/read it e.g. bulletin board?
   ☐ Yes
   ☐ No
2. **Are there FGM prevention posters on the wall of the waiting room?** ☐ Yes
   ☐ No
   2a. If yes, is it placed in place where ANC clients can see it
   ☐ Yes
   ☐ No
3. **Is there WHO FGM Clinical Handbook in the ANC consultation room?** ☐ Yes
   ☐ No
   3a. If yes, is it placed where ANC provider can see /use it?
   ☐ Yes
   ☐ No
4. **Is there FGM ABCD guide in ANC consultation room?**
   ☐ Yes
   ☐ No
   4a. If yes, is it placed where ANC provider can see /use it
   ☐ Yes ☐
   No

Instructions: Assess health facility factors that may facilitate/constrain intervention delivery by reviewing health facility administrative records and notes and by meeting with the health facility manager.

5. **Number of ANC providers** __________________
6. **Average number of ANC clients per month**__________________
7. **Number of ANC providers trained on PCC on FGM prevention**
   ☐ All (specify number trained): _________
   ☐ Some (specify number trained): _________
   ☐ None
8. **Indicate the number of MoH supervisory visits to the clinic in the past year**__________________

A HEALTH SYSTEMS APPROACH TO PREVENTION OF FEMALE GENITAL MUTILATION USING PERSON-CENTRED COMMUNICATION: IMPLEMENTATION RESEARCH PROJECT IN GUINEA, SOMALIA AND KENYA (A65993)

ANC PROVIDER SCREENING QUESTIONNAIRE (SCR)

Participant ID: ☐☐☐☐

Project ID: Country ID: Facility ID:

☐☐☐☐☐☐ ☐☐☐☐ ☐☐☐☐☐

A 6 5 9 9 3

9. **How frequently are staff meetings held?**
   - ☐ Monthly
   - ☐ Every 2 to 4 months
   - ☐ Every 6 to 12months
   - ☐ Never
10. **What is the size of the population served by this facility? (*specify number)* ________________**
11. **Are there country/region-specific FGM laws that are enforced?**
    - ☐ Yes
    - ☐ No
12. **Are there anti-FGM activities that target the population served by this health facility?**
    - ☐ Yes
    - ☐ No
13. **Are there pro-FGM activities that target the population served by this health facility?**
    - ☐ Yes
    - ☐ No

Additional comments:

____

____

____

____

For peer review only

A HEALTH SYSTEMS APPROACH TO PREVENTION OF FEMALE GENITAL MUTILATION USING PERSON-CENTRED COMMUNICATION: IMPLEMENTATION RESEARCH PROJECT IN GUINEA, SOMALIA AND KENYA (A65993)

ANC PROVIDER SCREENING QUESTIONNAIRE (SCR)

Participant ID: ☐☐☐☐

Project ID: ☐☐☐☐☐☐ Country ID: ☐☐☐☐ Facility ID: ☐☐☐☐☐

Version 2 – 18th October 2019 2

A 6 5 9 9 3

1. **What is your age?** ______________
2. **What is your sex?**
   1. ☐ Female
   2. ☐ Male
3. **What is your religion?**
   1. ☐ Muslim
   2. ☐ Christian
   3. ☐ Other
   4. ☐ None
   5. ☐ Refused
4. **What is your occupation/designation?**
   1. ☐ Midwife
   2. ☐ Nurse
   3. ☐ Other, specify _______________________________________
5. **What is the highest education level of education you achieved?**
   1. ☐ Certificate
   2. ☐ Diploma
   3. ☐ Bachelors
   4. ☐ Masters or above
   5. ☐ Other, specify _______________________________________
6. **For how many years have you been working in your field?** ________
7. **During you clinical training, did you receive any formal training on female genital mutilation?**
   1. ☐ Yes.
   2. ☐ No. Go to section B
   3. ☐ I don't know. Go to section B
8. **When did you receive the training?**
   1. ☐ During my studies (pre-service training)
   2. ☐ After graduation/at work (in-service training)
   3. ☐ Both
   4. ☐ I don't know

   7. ☐ Not applicable

For peer review only

For peer review only - http://bmjopen.bmj.com/site/about/guidelines.xhtml

A HEALTH SYSTEMS APPROACH TO PREVENTION OF FEMALE GENITAL MUTILATION USING PERSON-CENTRED COMMUNICATION: IMPLEMENTATION RESEARCH PROJECT IN GUINEA, SOMALIA AND KENYA (A65993)

ANC PROVIDER SCREENING QUESTIONNAIRE (SCR)

Participant ID:

Project ID:

Country ID:

Facility ID:

To be completed by data collector:

Data Collector ID:

Signature:

Date:

Day		Month			Year			
					2	0		

For peer review only

For peer review only

A HEALTH SYSTEMS APPROACH TO PREVENTION OF FEMALE GENITAL MUTILATION USING PERSON-CENTRED COMMUNICATION: IMPLEMENTATION RESEARCH PROJECT IN GUINEA, SOMALIA AND KENYA (A65993)

ANC PROVIDER SCREENING QUESTIONNAIRE (SCR)

Participant ID:

A	6	5	9	9	3

Project ID:

Country ID: Facility ID:

9. What was the format of the training? (Check all that apply)

1. ☐ Classroom lessons
2. ☐ Workshops
3. ☐ Digital format (E-learning videos; smart phone app)
4. ☐ During clinical practice under supervision of a mentor
5. ☐ Other, specify ____

7. ☐ Not applicable

10. During your pre- or post- graduate training, did you receive any formal training on communication or counselling?

1. ☐ Yes.
2. ☐ No.
3. ☐ I don't know

11. During you pre or post graduate training, did you receive any formal training on person-centred care?

1. ☐ Yes.
2. ☐ No.
3. ☐ I don't know

12. Have you ever cut the genitals of a girl (<=18 years old) for non-health reasons?

1. ☐ Yes.
2. ☐ No.
3. ☐ I don't know

For peer review only - http://bmjopen.bmj.com/site/about/guidelines.xhtml

A HEALTH SYSTEMS APPROACH TO PREVENTION OF FEMALE GENITAL MUTILATION USING PERSON-CENTRED COMMUNICATION: IMPLEMENTATION RESEARCH PROJECT IN GUINEA, SOMALIA AND KENYA (A65993)

ANC PROVIDER QUESTIONNAIRE (HCP)

Participant ID:

A	6	5	9	9	3

Project ID: Country ID: Facility ID:

To be completed by data collector:

Data Collector ID: *Date:*

Signature:

Day		Month			Year			
					2	0		

1. **Have you ever heard about female genital mutilation?**
   ☐ Yes
   ☐ No
2. **Do the women in your community undergo female genital mutilation?**
   ☐ Yes
   ☐ No
   ☐ I don't know
3. **Do you know of the WHO classification for female genital mutilation?**
   ☐ Yes
   ☐ No. Skip to Q5
4. **Please provide the WHO classification for the following FGM images (to include images)**
   a. **IMAGE of Type III FGM to be inserted here**
      i. ☐ **Type I**
      ii. ☐ **Type II**
      iii. ☐ **Type III**
      iv. ☐ **Type IV**
      v. ☐n**Don't know**
   b. **IMAGE of Type I FGM to be inserted here**
      i. ☐ **Type I**
      ii. ☐ **Type II**
      iii. ☐ **Type III**

To be completed by data collector:

Data Collector ID: *Date:*

Signature:

Day		Month			Year			
					2	0		

For peer review only

A HEALTH SYSTEMS APPROACH TO PREVENTION OF FEMALE GENITAL MUTILATION USING PERSON-CENTRED COMMUNICATION: IMPLEMENTATION RESEARCH PROJECT IN GUINEA, SOMALIA AND KENYA (A65993)

ANC PROVIDER QUESTIONNAIRE (HCP)

Participant ID: | | | | |

Project ID: | | | | | | | Country ID: | | | | | Facility ID: | | | | | |

iv. ☐ Type IV

v. ☐nDon't know

c. **IMAGE of Type II FGM to be inserted here**

i. ☐ Type I ii. ☐ Type II

A 6 5 9 9 3

iii. ☐ Type III

iv. ☐ Type IV

v. ☐nDon't know

d. **IMAGE of Type III FGM to be inserted here**

i. ☐ Type I

ii. ☐ Type II

iii. ☐ Type III

iv. ☐ Type IV

v. ☐nDon't know

e.

5. **Do you know of any health complications arising from female genital mutilation?**

☐ Yes

☐ No. Skip to Q6

6. **Is female genital mutilation illegal in your country (specify actual study country)?**

1. ☐ Yes
2. ☐ No
3. ☐ I don't know

7. **Are you aware of any existing WHO tools/guidance on female genital mutilation and its complications?**

1. ☐ Yes. *If yes, please specify*……………………………………………………….
2. ☐ No

To be completed by data collector:

Data Collector ID:	*Date:*		
Signature:	Day	Month	Year
			2 0

For peer review only - http://bmjopen.bmj.com/site/about/guidelines.xhtml

A HEALTH SYSTEMS APPROACH TO PREVENTION OF FEMALE GENITAL MUTILATION USING PERSON-CENTRED COMMUNICATION: IMPLEMENTATION RESEARCH PROJECT IN GUINEA, SOMALIA AND KENYA (A65993)

ANC PROVIDER QUESTIONNAIRE (HCP)

Participant ID: ☐☐☐☐

Project ID: ☐☐☐☐☐☐ Country ID: ☐☐☐☐ Facility ID: ☐☐☐☐☐

A 6 5 9 9 3

8. When you treat or attend a girl or a woman with genital mutilation, how confident are you that you have enough knowledge to provide good quality health care? *Rate between 1 – 4*

1. ☐ Not confident at all
2. ☐ Not very confident
3. ☐ Fairly confident
4. ☐ Fully confident

9. How confident are you in your FGM knowledge to communicate on FGM prevention? *Rate between 1 – 4*

1. ☐ Not confident at all
2. ☐ Not very confident
3. ☐ Fairly confident
4. ☐ Fully confident

For each of the following statements please state if you agree/disagree or don't know.

10. A girl who has not undergone FGM is unclean.

1. ☐ Agree
2. ☐ Disagree
3. ☐ I don't know

11. A girl without FGM cannot be married within her community.

1. ☐ Agree
2. ☐ Disagree
3. ☐ I don't know

12. A girl who has not undergone FGM is a disgrace to her family's honour.

1. ☐ Agree
2. ☐ Disagree

To be completed by data collector:

Data Collector ID: *Date:*

Signature:

Day		Month			Year			
					2	0		

For peer review only - http://bmjopen.bmj.com/site/about/guidelines.xhtml

A HEALTH SYSTEMS APPROACH TO PREVENTION OF FEMALE GENITAL MUTILATION USING PERSON-CENTRED COMMUNICATION: IMPLEMENTATION RESEARCH PROJECT IN GUINEA, SOMALIA AND KENYA (A65993)

ANC PROVIDER QUESTIONNAIRE (HCP)

Participant ID:

Project ID: Country ID: Facility ID:

3. ☐ I don't know

13. Health care providers who perform FGM are violating medical ethics.

1. ☐ Agree
2. ☐ Disagree
3. ☐ I don't know

14. Health care providers who perform FGM should be punished.

1. ☐ Agree
2. ☐ Disagree

A 6 5 9 9 3

3. ☐ I don't know

15. FGM is a good practice

1. ☐ Agree
2. ☐ Disagree
3. ☐ I don't know

16. FGM is a violation of women's and girls' rights

1. ☐ Agree
2. ☐ Disagree
3. ☐ I don't know

17. FGM is a religious mandate

1. ☐ Agree
2. ☐ Disagree
3. ☐ I don't know

18. Pretend you had a daughter now who was at an age when cutting occurs, what would your intention to cut her be? 1. ☐ Intend to cut her

2. ☐ Do not intend to cut her
3. ☐ Undecided
4. Refused to answer

19. If a family brought their daughter to the clinic requesting genital cutting for non-health reasons, would you perform it?

To be completed by data collector:

Data Collector ID: *Date:*

Signature:

Day		Month			Year			
					2	0		

For peer review only

For peer review only - http://bmjopen.bmj.com/site/about/guidelines.xhtml

A HEALTH SYSTEMS APPROACH TO PREVENTION OF FEMALE GENITAL MUTILATION USING PERSON-CENTRED COMMUNICATION: IMPLEMENTATION RESEARCH PROJECT IN GUINEA, SOMALIA AND KENYA (A65993)

ANC PROVIDER QUESTIONNAIRE (HCP)

Participant ID:

Project ID: Country ID: Facility ID:

1. ☐ Yes
2. ☐ No
3. ☐ I don't know

Now I would like to ask you a few questions about how you solve problems that you face. Please state how much you agree or disagree with the statements that I read, , where 1=Strongly disagree; 2=Disagree; 3=Neither agree nor disagree; 4=Agree; 5=Strongly agree

20. I will be able to achieve most of the goals that I have set for myself.

1. ☐ **Strongly disagree**
2. ☐ **Disagree**
3. ☐ **Neither agree nor disagree**
4. ☐ **Agree**

A 6 5 9 9 3

5. ☐ **Strongly agree**
6. ☐ **Don't know**

21. When facing difficult tasks, I am certain that I will accomplish them.

1. ☐ **Strongly disagree**
2. ☐ **Disagree**
3. ☐ **Neither agree nor disagree**
4. ☐ **Agree**
5. ☐ **Strongly agree**
6. ☐ **Don't know**

22. In general, I think that I can obtain outcomes that are important to me.

1. ☐ **Strongly disagree**
2. ☐ **Disagree**
3. ☐ **Neither agree nor disagree**

To be completed by data collector:

Data Collector ID: *Date:*

Signature:

Day		Month			Year			
					2	0		

For peer review only

A HEALTH SYSTEMS APPROACH TO PREVENTION OF FEMALE GENITAL MUTILATION USING PERSON-CENTRED COMMUNICATION: IMPLEMENTATION RESEARCH PROJECT IN GUINEA, SOMALIA AND KENYA (A65993)

ANC PROVIDER QUESTIONNAIRE (HCP)

Participant ID:

Project ID: Country ID: Facility ID:

4. ☐ **Agree**
5. ☐ **Strongly agree**
6. ☐ **Don't know**

23. I believe I can succeed at most any endeavour to which I set my mind.

1. ☐ **Strongly disagree**
2. ☐ **Disagree**
3. ☐ **Neither agree nor disagree**
4. ☐ **Agree**
5. ☐ **Strongly agree**
6. ☐ **Don't know**

24. I will be able to successfully overcome many challenges.

1. ☐ **Strongly disagree**
2. ☐ **Disagree**
3. ☐ **Neither agree nor disagree**
4. ☐ **Agree**
5. ☐ **Strongly agree**

A 6 5 9 9 3

6. ☐ **Don't know**

25. I am confident that I can perform effectively on many different tasks.

1. ☐ **Strongly disagree**
2. ☐ **Disagree**
3. ☐ **Neither agree nor disagree**
4. ☐ **Agree**
5. ☐ **Strongly agree**
6. ☐ **Don't know**

To be completed by data collector:

Data Collector ID:	*Date:*								
Signature:	Day		Month			Year			
						2	0		

For peer review only

A HEALTH SYSTEMS APPROACH TO PREVENTION OF FEMALE GENITAL MUTILATION USING PERSON-CENTRED COMMUNICATION: IMPLEMENTATION RESEARCH PROJECT IN GUINEA, SOMALIA AND KENYA (A65993)

ANC PROVIDER QUESTIONNAIRE (HCP)

Participant ID:

Project ID: Country ID: Facility ID:

26. Compared to other people, I can do most tasks very well.

1. ☐ **Strongly disagree**
2. ☐ **Disagree**
3. ☐ **Neither agree nor disagree**
4. ☐ **Agree**
5. ☐ **Strongly agree**
6. ☐ **Don't know**

For peer review only

27. Even when things are tough, I can perform quite well.

1. ☐ **Strongly disagree**
2. ☐ **Disagree**
3. ☐ **Neither agree nor disagree**
4. ☐ **Agree**
5. ☐ **Strongly agree**
6. ☐ **Don't know**

A 6 5 9 9 3

28. Would you like to receive more training related to care for women and girls with FGM?

1. ☐ Yes
2. ☐ No
3. ☐ I don't know

29. If a pregnant woman is expected to have a girl, do you discourage her from having her daughter cut?

To be completed by data collector:

Data Collector ID: *Date:*

Signature:

Day		Month			Year			
					2	0		

A HEALTH SYSTEMS APPROACH TO PREVENTION OF FEMALE GENITAL MUTILATION USING PERSON-CENTRED COMMUNICATION: IMPLEMENTATION RESEARCH PROJECT IN GUINEA, SOMALIA AND KENYA (A65993)

ANC PROVIDER QUESTIONNAIRE (HCP)

Participant ID: | | | | |

A	6	5	9	9	3

Project ID: | | | | |

Country ID: Facility ID: | | | | | |

1. ☐ Always
2. ☐ Often
3. ☐ Sometimes
4. ☐ Rarely
5. ☐ Never

30. If you heard of or saw a colleague performing female genital mutilation, what would you do? ***(Tick all that apply)***

1. ☐ I would report him/her to the authorities
2. ☐ I would discuss with him/her and explain to him/her that health care providers should not perform female genital mutilation
3. ☐ I would not get involved 4. ☐ I don't know

31. How often do you look for female genital cutting/excision when performing a gynecological examination of the vulva?

1. ☐ Always
2. ☐ Often
3. ☐ Sometimes
4. ☐ Rarely
5. ☐ Never

32. How often do you record the female genital mutilation in the women's medical file if you are aware that she has undergone FGM?

1. ☐ Always
2. ☐ Often
3. ☐ Sometimes
4. ☐ Rarely
5. ☐ Never

33. Would you like to receive more training on how to help patients to prevent FGM?

1. ☐ Yes
2. ☐ No
3. ☐ I don't know

34. I can put myself in others' shoes

1. ☐ Always
2. ☐ Often

To be completed by data collector:

Data Collector ID: *Date:*

Signature:

Day		Month			Year			
					2	0		

For peer review only - http://bmjopen.bmj.com/site/about/guidelines.xhtml

For peer review only

A HEALTH SYSTEMS APPROACH TO PREVENTION OF FEMALE GENITAL MUTILATION USING PERSON-CENTRED COMMUNICATION: IMPLEMENTATION RESEARCH PROJECT IN GUINEA, SOMALIA AND KENYA (A65993)

ANC PROVIDER QUESTIONNAIRE (HCP)

Participant ID:

Project ID: Country ID: Facility ID:

3. ☐ Sometimes
4. ☐ Rarely
5. ☐ Never

35. I let others know I understand what they say
1. ☐ Always
2. ☐ Often
3. ☐ Sometimes
4. ☐ Rarely
5. ☐ Never

36. In conversations with my colleagues, I perceive not only what they say but what they don't say
1. ☐ Always
2. ☐ Often
3. ☐ Sometimes
4. ☐ Rarely
5. ☐ Never

37. I communicate effectively
1. ☐ Always
2. ☐ Often
3. ☐ Sometimes
4. ☐ Rarely
5. ☐ Never

38. I communicate with others as though they are my equals
1. ☐ Always
2. ☐ Often
3. ☐ Sometimes
4. ☐ Rarely
5. ☐ Never

A	6	5	9	9	3

To be completed by data collector:

Data Collector ID: *Date:*

Signature:

Day		Month			Year			
					2	0		

For peer review only

A HEALTH SYSTEMS APPROACH TO PREVENTION OF FEMALE GENITAL MUTILATION USING PERSON-CENTRED COMMUNICATION: IMPLEMENTATION RESEARCH PROJECT IN GUINEA, SOMALIA AND KENYA (A65993)

ANC PROVIDER QUESTIONNAIRE (HCP)

Participant ID: | | | | |

Project ID: | | | | | Country ID: | | | | | Facility ID: | | | | | |

These next questions relate to your clinic setting:

39. Have you seen the posters on FGM at the clinic?

1. ☐ Yes
2. ☐ No
3. ☐ I don't know

40. Have you referred to the clinical handbook on FGM that is available in your clinic?

1. ☐ No
2. ☐ I don't know

41. Do you think it is feasible to provide FGM prevention counselling during ANC visits?

1. ☐ Yes
2. ☐ No
3. ☐ I don't know

Comments

-

____
____
____
____
____

To be completed by data collector:

Data Collector ID: *Date:*

Signature:

Day		Month			Year			
					2	0		

For peer review only

A HEALTH SYSTEMS APPROACH TO PREVENTION OF FEMALE GENITAL MUTILATION USING PERSON-CENTRED COMMUNICATION: IMPLEMENTATION RESEARCH PROJECT IN GUINEA, SOMALIA AND KENYA (A65993)

Participant ID:

Project ID: Facility ID:

FIRST ANC CLIENT EXIT QUESTIONNAIRE (EXT)

Country ID:

A 6 5 9 9 3

1. **How old are you? (years)**____________________________________
2. **What is your religion?**
   1. ☐ Muslim
   2. ☐ Christian
   3. ☐ Other
   4. ☐ None
   5. ☐ Refused
3. **What is the highest level of education you achieved?**
   1. ☐ None
   2. ☐ Primary
   3. ☐ Secondary
   4. ☐ University
   5. ☐ Other, *specify* ____________________________________
4. **Many women in your community have had their genitals cut when they were children, if you are comfortable telling me, can I ask if you have undergone this practice?**
   1. ☐ Yes
   2. ☐ No
   3. ☐ I don't know
   4. ☐ Refused
5. **How supportive are you of female genital mutilation?**
   1. ☐ Strongly opposed
   2. ☐ Somewhat opposed
   3. ☐ Neutral (Neither opposed or supportive)
   4. ☐ Somewhat supportive
   5. ☐ Strongly supportive

The following questions relate to your visit today. During your visit today:

6. **Did you see any FGM poster(s) in the waiting room?**
   1. ☐ Yes
   2. ☐ No
   3. ☐ I don't know

For peer review only

A HEALTH SYSTEMS APPROACH TO PREVENTION OF FEMALE GENITAL MUTILATION USING PERSON-CENTRED COMMUNICATION: IMPLEMENTATION RESEARCH PROJECT IN GUINEA, SOMALIA AND KENYA (A65993)

Participant ID:

Project ID: Facility ID:

Country ID:

A 6 5 9 9 3

7. Did the ANC provider ask if you have undergone FGM?

1. ☐ Yes
2. ☐ No
3. ☐ I don't know

8. Did the ANC provider explain how FGM can harm your health?

1. ☐ Yes
2. ☐ No
3. ☐ I don't know

9. Did the ANC provider ask about your personal belief regarding FGM?

1. ☐ Yes
2. ☐ No
3. ☐ I don't know

10. Did the ANC provider discuss why FGM should be prevented?

1. ☐ Yes
2. ☐ No
3. ☐ I don't know

11. Did the ANC provider discuss how FGM could be prevented?

1. ☐ Yes
2. ☐ No
3. ☐ I don't know

12. Did you have questions about FGM to ask the ANC provider?

1. ☐ Yes
2. ☐ No
3. ☐ I don't know

13. Did you feel encouraged to ask questions about FGM?

1. ☐ Yes
2. ☐ No
3. ☐ I don't know

14. Are you satisfied with how FGM was addressed during your visit with your ANC provider today?

For peer review only

For peer review only - http://bmjopen.bmj.com/site/about/guidelines.xhtml

A HEALTH SYSTEMS APPROACH TO PREVENTION OF FEMALE GENITAL MUTILATION USING PERSON-CENTRED COMMUNICATION: IMPLEMENTATION RESEARCH PROJECT IN GUINEA, SOMALIA AND KENYA (A65993)

Participant ID: | | | | |

Project ID: | | | | |

Facility ID: | | | | | |

1. ☐ Yes
2. ☐ No
3. ☐ I don't know

15. What do you feel about FGM now as compared to before you came to the clinic today?

1. ☐ Same, no change
2. ☐ I feel more supportive of FGM now as compared to before I came
3. ☐ I feel less supportive of FGM now as compared to before I came
4. ☐ I do not know
5. ☐ Other, *specify* ____

16. Pretend you had a daughter now who was at an age when cutting occurs, what would your intention to cut her be?

1. ☐ Intend to cut her
2. ☐ Do not intend to cut her

17. Do you wish/want to be active in preventing FGM?

1. ☐ Yes
2. ☐ No
3. ☐ I don't know

For peer review only

For peer review only - http://bmjopen.bmj.com/site/about/guidelines.xhtml

Supplementary file 3: Additional analyses (appendices 1 – 3)

Appendix 1: Comparison baseline characteristics of ANC facilities

**Characteristics**	**Facilities included in final analysis (n=163)**	**Facilities excluded* from final analysis (n=17)**
Number of ANC providers	Mean 4 (SD: 3) Median 3 (1-14, IQR 3)	Mean 3 (SD: 3) Median 2 (1-9, IQR 1)
Average number of ANC clients/month	Mean 150 (SD: 127) Median 118 (3-664, IQR 141)	Mean 162 (SD: 147) Median 100 (25-600, IQR 200)
MoH supervisory visits in the past year	Mean 4 (SD: 3) Median 3 (0-18, IQR 2)	Mean 5 (SD: 4) Median 4 (0-12, IQR 4)
Size of catchment population served	Mean 36,754 (SD: 126,082) Median 15,972 (1,000-1,458,000, IQR 24,332)	Mean 11,737 (SD: 14,62) Median 7,800 (1,200-63,000, IQR 7,505
Presence of anti-FGM activities in the catchment area		
Yes	74 (45%)	9 (53%)
No	89 (55%)	8 (47%)
Presence of pro-FGM activities in the catchment area		
Yes	21 (13%)	2 (12%)
No	140 (86%)	15 (88%)
Don't Know	2 (1%)	-

*Total of ANC clinics not included: 16 clinics were excluded (7 intervention and 9 control) due to loss-to-follow up (LTFU) of ANC provider i.e., the clinics did not have at least one ANC provider present across all study time points while one ANC clinic in Kenya was never visited at subsequent time points due to issues with insecurity. An ANC provider from one of the clinics in Kenya that had been inaccessible due to insecurity attended the PCC training and was subsequently interviewed.

For peer review only

Appendix 2: Comparison of baseline characteristics of ANC providers

Characteristics	Providers recruited at Baseline (n=436)	Providers enrolled with complete data at Month 6 (n=232)	Providers not enrolled with no data at Month 6 (n=204)
Age	37 (20-65; SD: 10)	36 (20-65; SD: 10)	38 (21-62; SD: 10)
Years of professional experience	9 (1-39; SD: 7)	8 (1-39; SD: 7)	10 (1-36; SD: 8)
Sex			
Female	361 (83%)	193 (83%)	168 (82%)
Male	75 (17%)	39 (17%)	36 (18%)
Highest educational level			
Certificate	44 (3%)	21 (5%)	23 (11%)
Diploma	309 (71%)	158 (68%)	151 (74%)
Bachelors	64 (15%)	44 (19%)	20 (10%)
Masters & above	3 (0.7%)	1 (0.4%)	2 (1%)
Other[#]	16 (4%)	8 (3%)	8 (4%)
Current professional role/title			
Midwife	198 (45%)	103 (44%)	95 (47%)
Nurse	95 (22%)	51 (22%)	44 (22%)
Nurse-Midwife	94 (22%)	54 (23%)	40 (20%)
Other	49 (11%)	24 (10%)	25 (12%)
Received formal training on FGM during clinical training			
Yes	158 (36%)	85 (37%)	73 (36%)
No	275 (63%)	146 (63%)	129 (63%)
Don't Know	3 (0.7%)	1 (0.4%)	2 (1%)
Timing of clinical training on FGM			
Pre-service	63 (14%)	33 (14%)	30 (15%)
In-service	81 (19%)	45 (19%)	36 (18%)
Both pre- and in-service	14 (3%)	7 (3%)	7 (3%)
Received formal training on communication/counselling			
Yes	287 (66%)	149 (64%)	138 (68%)
No	149 (34%)	83 (36%)	66 (32%)
Received formal training on person-centered care			
Yes	227 (52%)	118 (51%)	109 (53%)
No	207 (47%)	131 (56%)	94 (46%)
Don't know	2 (0.5%)	1 (0.4%)	1 (0.5%)

For peer review only

Characteristics	Providers recruited at Baseline (n=436)	Providers enrolled with complete data at Month 6 (n=232)	Providers not enrolled with no data at Month 6 (n=204)
Undergone FGM			
Yes	226 (52%)	126 (54%)	100 (49%)
No	128 (29%)	63 (27%)	65 (32%)
Don't know	4 (0.9%)	2 (1%)	1 (0.5%)
Refused to answer	3 (0.7%)	2 (1%)	1 (0.55)
Conducted FGM			
Yes	35 (8%)	15 (7%)	20 (10%)
Conducted FGM on a girl <18 years			
Yes	32 (7%)	14 (6%)	18 (9%)

For peer review only

Appendix 3: Comparison of study outcomes between baseline vs. month 3 and month 3 vs. month 6 in the intervention arm

	**Baseline (Intervention only)**	**Month 3 (Intervention only)**	**P-value**	**Month 3 (Intervention only)**	**Month 6 (Intervention only)**	**P-value**
**Primary Outcomes**						
**ANC clients reporting that their provider implemented components of PCC for FGM prevention**						
Provider asked client if they have undergone FGM	48 (6%)	298 (37%)	<0.0001	298 (37%)	694 (78%)	<0.0001
Provider asked client about their (client's) personal beliefs regarding FGM	38 (5%)	239 (29%)	<0.0001	239 (29%)	616 (76%)	<0.0001
Provider discussed with client why FGM should be prevented	56 (7%)	243 (30%)	<0.0001	243 (30%)	629 (77%)	<0.0001
Provider discussed with client how FGM could be prevented	48 (6%)	224 (28%)	<0.0001	224 (28%)	592 (73%)	<0.0001
Client satisfied with how FGM was addressed by provider during clinic visit	176 (21%)	346 (43%)	<0.0001	346 (43%)	684 (84%)	<0.0001
Mean score of PCC approach (out of 5)	0.5 (0.4-0.5)	1.7 (1.5-1.8)	<0.0001	1.7 (1.5-1.8)	3.9 (3.8-4.0)	<0.0001
Mean score of PCC + appropriate FGM prevention & care (out of 8)	1.8 (1.6-2.1)	3.3 (2.8-3.8)	<0.0001	3.3 (2.8-3.8)	6.2 (5.9 – 6.6)	<0.0001
**ANC clinic preparedness to offer FGM prevention and care services**						
Clinics with ALL correct answers for facility preparedness	0 (0%)	42 (52%)	<0.0001	42 (52%)	56 (69%)	<0.01
Mean score of clinic preparedness (out of 4)	0.1 (0.01-0.2)	3.1 (2.9-3.4)	<0.0001	3.1 (2.9-3.4)	3.4 (3.2-3.6)	0.18
Providers using level 1 intervention package	1 (1%)	61 (58%)	<0.0001	61 (58%)	96 (91%)	<0.0001
Providers offering appropriate FGM-related prevention and care services	11 (11%)	20 (19%)	<0.0001	20 (19%)	52 (50%)	<0.0001
**Secondary Outcomes**						
Providers with correct FGM-related knowledge responses	0 (0%)	1 (3%)	0.47	1 (3%)	8 (8%)	0.06
Providers with appropriate interpersonal communication skills	49 (49%)	62 (59%)	0.08	62 (59%)	74 (70%)	0.11
Providers with high self-efficacy	85 (85%)	94 (90%)	0.18	94 (90%)	86 (82%)	0.17
Providers reporting less supportive attitudes towards FGM	67 (67%)	75 (71%)	0.26	75 (71%)	76 (72%)	0.50
Providers with high confidence scores	84 (83%)	81 (77%)	0.30	81 (77%)	103 (98%)	<0.001
Providers not supportive of FGM	91 (91%)	101 (96%)	0.16	101 (96%)	100 (96%)	1.0
Providers not supportive of medicalized FGM	98 (97%)	104 (99%)	0.36	104 (99%)	104 (99%)	0.75
**Other ANC Client Outcomes**						
Clients reporting less support for FGM after ANC clinic visit	194 (24%)	235 (29%)	0.01	235 (29%)	424 (52%)	<0.0001
Clients reporting that they were strongly opposed to FGM	367 (45%)	345 (43%)	0.38	345 (43%)	498 (61%)	<0.0001
Clients reporting that they intend to have their daughters cut	249 (30%)	184 (23%)	<0.0001	184 (23%)	96 (12%)	<0.0001
Clients reporting that they would prefer health care provider to cut daughters	141 (17%)	117 (14%)	0.003	117 (14%)	53 (7%)	<0.001
Clients wishing to be active in FGM prevention	530 (65%)	547 (68%)	0.22	547 (68%)	677 (83%)	<0.001

For peer review only

CONSORT 2010 checklist of information to include when reporting a cluster randomised trial

Section/Topic	Item No	Standard Checklist item	Extension for cluster designs	Page No *
**Title and abstract**				
	1a	Identification as a randomised trial in the title	Identification as a cluster randomised trial in the title	1
	1b	Structured summary of trial design, methods, results, and conclusions (for specific guidance see CONSORT for abstracts)[1,2]	See table 2	3
**Introduction**				
**Background and objectives**	2a	Scientific background and explanation of rationale	Rationale for using a cluster design	5-6
	2b	Specific objectives or hypotheses	Whether objectives pertain to the cluster level, the individual participant level or both	7
**Methods**				
**Trial design**	3a	Description of trial design (such as parallel, factorial) including allocation ratio	Definition of cluster and description of how the design features apply to the clusters	7
	3b	Important changes to methods after trial commencement (such as eligibility criteria), with reasons		N/A
**Participants**	4a	Eligibility criteria for participants	Eligibility criteria for clusters	7-8
	4b	Settings and locations where the data were collected		6-7
**Interventions**	5	The interventions for each group with sufficient details to allow replication, including how and when they were actually administered	Whether interventions pertain to the cluster level, the individual participant level or both	6
**Outcomes**	6a	Completely defined pre-specified primary and	Whether outcome measures pertain to the cluster level, the	10

		secondary outcome measures, including how and when they were assessed	individual participant level or both	
	6b	Any changes to trial outcomes after the trial commenced, with reasons		N/A
**Sample size**	7a	How sample size was determined	Method of calculation, number of clusters(s) (and whether equal or unequal cluster sizes are assumed), cluster size, a coefficient of intra-cluster correlation (ICC or *k*), and an indication of its uncertainty	10-11
	7b	When applicable, explanation of any interim analyses and stopping guidelines		12
**Randomisation:**				
**Sequence generation**	8a	Method used to generate the random allocation sequence		8-9
	8b	Type of randomisation; details of any restriction (such as blocking and block size)	Details of stratification or matching if used	8-9
**Allocation concealment mechanism**	9	Mechanism used to implement the random allocation sequence (such as sequentially numbered containers), describing any steps taken to conceal the sequence until interventions were assigned	Specification that allocation was based on clusters rather than individuals and whether allocation concealment (if any) was at the cluster level, the individual participant level or both	9
**Implementation**	10	Who generated the random allocation sequence, who enrolled participants, and who assigned participants to interventions	Replace by 10a, 10b and 10c	
	10a		Who generated the random allocation sequence, who enrolled clusters, and who assigned clusters to interventions	8

	10b		Mechanism by which individual participants were included in clusters for the purposes of the trial (such as complete enumeration, random sampling)	8
	10c		From whom consent was sought (representatives of the cluster, or individual cluster members, or both), and whether consent was sought before or after randomisation	8
**Blinding**	11a	If done, who was blinded after assignment to interventions (for example, participants, care providers, those assessing outcomes) and how		8-9
	11b	If relevant, description of the similarity of interventions		8-9
**Statistical methods**	12a	Statistical methods used to compare groups for primary and secondary outcomes	How clustering was taken into account	10-13
	12b	Methods for additional analyses, such as subgroup analyses and adjusted analyses		12-13
**Results**				
**Participant flow (a diagram is strongly recommended)**	13a	For each group, the numbers of participants who were randomly assigned, received intended treatment, and were analysed for the primary outcome	For each group, the numbers of clusters that were randomly assigned, received intended treatment, and were analysed for the primary outcome	15
	13b	For each group, losses and exclusions after randomisation, together with reasons	For each group, losses and exclusions for both clusters and individual cluster members	Figure 2

For peer review only

**Recruitment**	14a	Dates defining the periods of recruitment and follow-up		15
	14b	Why the trial ended or was stopped		N/A
**Baseline data**	15	A table showing baseline demographic and clinical characteristics for each group	Baseline characteristics for the individual and cluster levels as applicable for each group	15-16
**Numbers analysed**	16	For each group, number of participants (denominator) included in each analysis and whether the analysis was by original assigned groups	For each group, number of clusters included in each analysis	15-16
**Outcomes and estimation**	17a	For each primary and secondary outcome, results for each group, and the estimated effect size and its precision (such as 95% confidence interval)	Results at the individual or cluster level as applicable and a coefficient of intra-cluster correlation (ICC or k) for each primary outcome	17-19
	17b	For binary outcomes, presentation of both absolute and relative effect sizes is recommended		19
**Ancillary analyses**	18	Results of any other analyses performed, including subgroup analyses and adjusted analyses, distinguishing pre-specified from exploratory		19
**Harms**	19	All important harms or unintended effects in each group (for specific guidance see CONSORT for harms[3])		N/A
**Discussion**				
**Limitations**	20	Trial limitations, addressing sources of potential bias, imprecision, and, if relevant, multiplicity of analyses		22-23
**Generalisability**	21	Generalisability (external validity, applicability) of the trial findings	Generalisability to clusters and/or individual participants (as relevant)	23

For peer review only

**Interpretation**	22	Interpretation consistent with results, balancing benefits and harms, and considering other relevant evidence	23-24
**Other information**			
**Registration**	23	Registration number and name of trial registry	15
**Protocol**	24	Where the full trial protocol can be accessed, if available	15
**Funding**	25	Sources of funding and other support (such as supply of drugs), role of funders	In Funding Statement

** Note: page numbers optional depending on journal requirements*

For peer review only

BMJ Open

A cluster randomized trial of a health system strengthening approach applying person-centered communication for the prevention of female genital mutilation in Guinea, Kenya, and Somalia

Journal:	*BMJ Open*
Manuscript ID	bmjopen-2023-078771.R2
Article Type:	Original research
Date Submitted by the Author:	02-May-2024
Complete List of Authors:	Balde, Mamadou ; Cellulle de Recherche en Sante de la Reproduction en Guinee (CERREGUI)<br>Ndavi, Patrick; University of Nairobi College of Health Sciences, Department of Obstetrics & Gynecology<br>Oyaro, Vernon; World Health Organization, Department of Sexual and Reproductive Health and Research<br>Soumah, Anne-Marie; Cellulle de Recherche en Sante de la Reproduction en Guinee (CERREGUI)<br>Esho, Tammary; Amref International University<br>King'oo, James; Technical University of Kenya<br>Kemboi , Jackline; Amref Health Africa<br>Sall, Alpha; Cellulle de Recherche en Sante de la Reproduction en Guinee (CERREGUI)<br>Diallo, Aissatou; Cellulle de Recherche en Sante de la Reproduction en Guinee (CERREGUI)<br>Ahmed, Wisal; World Health Organization, Department of Sexual and Reproductive Health and Research<br>Stein, Karin; World Health Organization<br>Nosirov, Khurshed; World Health Organization, Department of Sexual and Reproductive Health and Research<br>Thwin, Soe Soe; World Health Organization, Department of Sexual and Reproductive Health and Research, including UNDP/UNFPA/UNICEF/WHO/World Bank Special Programme of Research, Development and Research Training in Human Reproduction (HRP)<br>Petzold, Max; University of Gothenburg Sahlgrenska Academy, Public Health and Community Medicine<br>Ahmed, Muna; Ministry of Planning and National Development, Central Statistics Department; MUFEIS Multidisciplinary Consultancy Firm , CEO<br>Diriye, Ahmed; Data and Research Solutions<br>Pallitto, C ; World Health Organization, Department of Sexual and Reproductive Health and Research
**Primary Subject Heading**:	Health services research
Secondary Subject Heading:	Evidence based practice, Reproductive medicine, Research methods, Communication, Complementary medicine

Keywords:	EDUCATION & TRAINING (see Medical Education & Training), Patient-Centered Care, Primary Care < Primary Health Care, Primary Prevention, PUBLIC HEALTH, Behavior

SCHOLARONE™
Manuscripts

I, the Submitting Author has the right to grant and does grant on behalf of all authors of the Work (as defined in the below author licence), an exclusive licence and/or a non-exclusive licence for contributions from authors who are: i) UK Crown employees; ii) where BMJ has agreed a CC-BY licence shall apply, and/or iii) in accordance with the terms applicable for US Federal Government officers or employees acting as part of their official duties; on a worldwide, perpetual, irrevocable, royalty-free basis to BMJ Publishing Group Ltd ("BMJ") its licensees and where the relevant Journal is co-owned by BMJ to the co-owners of the Journal, to publish the Work in this journal and any other BMJ products and to exploit all rights, as set out in our licence.

The Submitting Author accepts and understands that any supply made under these terms is made by BMJ to the Submitting Author unless you are acting as an employee on behalf of your employer or a postgraduate student of an affiliated institution which is paying any applicable article publishing charge ("APC") for Open Access articles. Where the Submitting Author wishes to make the Work available on an Open Access basis (and intends to pay the relevant APC), the terms of reuse of such Open Access shall be governed by a Creative Commons licence – details of these licences and which Creative Commons licence will apply to this Work are set out in our licence referred to above.

Other than as permitted in any relevant BMJ Author's Self Archiving Policies, I confirm this Work has not been accepted for publication elsewhere, is not being considered for publication elsewhere and does not duplicate material already published. I confirm all authors consent to publication of this Work and authorise the granting of this licence.

For peer review only

A cluster randomized trial of a health system strengthening approach applying person-centered communication for the prevention of female genital mutilation in Guinea, Kenya, and Somalia

Authors*:* *Prof. Mamadou Balde, MD[1*], Prof. Patrick Ndavi, MMed[2*], Dr. Vernon Mochache, PhD[3] Dr. Anne-Marie Soumah, MSc[1], Prof. Tammary Esho, PhD[4], James Munyao King'oo, MSc[5], Jackline Kemboi, MSc[2], Alpha Oumar Sall, MSc[1], Aissatou Diallo, MSc[1], Dr. Wisal Ahmed, PhD[3], Dr. Karin Stein, MD[3], Khurshed Nosirov, MCS[3], Dr. Soe Soe Thwin, PhD[3], Prof. Max Petzold, PhD[6], Muna Abdi Ahmed, MSc[7], Ahmed Diriye, MA[8], Dr. Christina Pallitto, PhD[3]*

Institutional Affiliations: [1]Centre for Research in Reproductive Health in Guinea, Conakry, Guinea; [2]Department of Obstetrics and Gynecology, University of Nairobi, Nairobi, Kenya; [3]Department of Sexual and Reproductive Health and Research, and the UNDP/UNFPA/UNICEF/WHO/World Bank Special Programme of Research, Development and Research Training in Human Reproduction (HRP), World Health Organization, Geneva, Switzerland; [4]Amref International University, Nairobi, Kenya; [5]Technical University of Kenya, Nairobi, Kenya; [6]School of Public Health and Community Medicine, Institute of Medicine, University of Gothenburg, Gothenburg, Sweden; [7] Somaliland Central Statistics Department, Hargeisa, Somalia; [8]Data and Research Solutions, Hargeisa, Somalia

** Joint first authors*

Correspondence to:

Dr. Christina Pallitto,

World Health Organization,

Department of Sexual and Reproductive Health and Research

20 Avenue Appia, 1211, Geneva, Switzerland

Telephone: +41 22 791 4745

Email: pallittoc@who.int

For peer review only - http://bmjopen.bmj.com/site/about/guidelines.xhtml

Abstract word count: 299

Text word count: 6,084

Tables: 4

Figures: 1

For peer review only

For peer review only - http://bmjopen.bmj.com/site/about/guidelines.xhtml

ABSTRACT

Introduction: There is limited evidence on effective health systems interventions for preventing female genital mutilation (FGM). This study tested a two-level intervention package at primary care applying person-centred communication (PCC) for FGM prevention.

Methods: A cluster randomized trial was conducted in 2020 - 2021 in 180 antenatal care (ANC) clinics in Guinea, Kenya, and Somalia. At baseline, all clinics received guidance and materials on FGM prevention and care, while at month three, ANC providers at intervention sites received PCC training. Data were collected from clinic managers, ANC providers and clients at baseline, months three and six. Multi-level and single-level logistic regression models were used to analyze the effect of the intervention on study outcomes.

Results: Providers in the intervention arm were more likely to implement the PCC for FGM prevention approach compared to those in the control arm, including inquiring about clients' FGM status (OR: 8.9, 95% CI: 6.9-11.5; $p<0.001$) and FGM-related beliefs (OR: 9.7, 95% CI: 7.5-12.5; $p<0.001$) and discussing why (OR: 9.2, 95% CI: 7.1-11.9; $p<0.001$) or how (OR: 7.7, 95% CI: 6.0-9.9; $p<0.001$) FGM should be prevented. They were also more confident in their FGM-related knowledge (OR: 6.3, 95% CI: 1.4-28.9; $p=0.02$) and communication skills (OR: 1.7; 95% CI: 1.0-3.0; $p=0.06$). ANC clients in the intervention arm were less supportive of FGM (AOR: 5.4, 95% CI: 2.4-12.4; $p<0.001$], more interested in being actively engaged in FGM prevention efforts (AOR: 3.2, 95% CI: 1.6-6.2; $p=0.001$) and had lower intentions of having their daughters undergo FGM (AOR: 0.3, 95% CI: 0.1-0.7; $p=0.004$) or seeking medicalized FGM (AOR: 0.2, 95% CI: 0.1-0.5; $p<0.001$) compared to those in the control arm.

Conclusion: This is the first study to provide evidence of an effective intervention to promote FGM prevention that can be delivered in primary care setting in high prevalence countries.

For peer review only - http://bmjopen.bmj.com/site/about/guidelines.xhtml

SUMMARY BOX

- This hybrid-effectiveness implementation research study conducted in primary care public health facilities in three countries with high prevalence of female genital mutilation (FGM) assessed the role of health workers in providing FGM prevention communication in the context of routine antenatal care (ANC).
- It will be important to assess the effectiveness of the person-centred communication approach in other service delivery points, e.g., child immunization, and with other cadres of health workers, e.g., community health workers, to assess its effectiveness beyond ANC care.
- Many factors influence FGM-related decision-making, and while primary care health workers were found to be effective communicators, and the randomized design controlled for some external factors, the impact of a health sector intervention in conjunction with multi-sectoral initiatives requires futher investigation.
- To ensure participation of at least one ANC provider at each site through each time point, eligibility of health workers was based on clinic rotation schedules, which may have introduced a selection bias although the included and excluded providers did not appear to differ significantly.

For peer review only

For peer review only - http://bmjopen.bmj.com/site/about/guidelines.xhtml

INTRODUCTION

Multi-sectoral efforts are needed to achieve Sustainable Development Goal (SDG) 5.3 to eliminate the harmful practice of female genital mutilation (FGM) by 2030 in line with the United Nation's (UN) General Assembly resolution 67/146 (1), the World Health Assembly Resolution 61.16 (2) and the 2008 Interagency Statement (3), which call upon UN Member States to enact comprehensive and multi-disciplinary national action plans and strategies towards the elimination of the practice. Identifying effective strategies across sectors is an important step in ending FGM.

The health system, defined as all organizations, institutions and resources that produce actions whose primary purpose is to improve health(4), has an important role to play not only in managing complications of FGM but also in preventing the practice. Health care providers, specifically nurses and midwives who constitute most of the health workforce, are highly respected members of FGM practising communities and could positively contribute to abandonment efforts (5,6). However, there is currently limited evidence to guide health programming on FGM prevention (7). In addition, some health care providers are themselves supportive of this harmful practice, and might even perform it (i.e., FGM medicalization), despite national laws and medical ethics forbidding it (8–11). Developing evidence-based tools to build skills of health care providers and address their underlying beliefs could contribute to FGM abandonment efforts and complement existing resources on management of complications (12,13) to ensure comprehensive and high quality care.

Three countries (Guinea, Somalia, and Kenya) participated in a cluster randomized trial to test the effectiveness and implementation of a health system strengthening approach to FGM, which included the testing of an intervention to build skills of health workers on applying person-centered communication

For peer review only - http://bmjopen.bmj.com/site/about/guidelines.xhtml

(PCC) for the prevention of FGM (14). Study countries were selected based on their high national and/or sub-national FGM prevalence. The national prevalence of FGM among women and girls aged 15 - 49 years is 98% in Somalia, 97% in Guinea and 21% in Kenya according to national population-based surveys. There are 20 hotspot counties/sub-national administrative units in Kenya with a prevalence of >80% (15), and this study focused on three of these counties. Likewise, the study countries have high rates of medicalized FGM, performed primarily by midwives, who make up between 71% to 93% of primary health care providers in the three study countries (16) hence the selection of nurses and midwives as the target group for this intervention.

The purpose of this study was to test a two-level intervention package to enable ANC providers to deliver person-centered FGM counseling to their clients.[1] This intervention package was informed by a theory of change that promotes health workers to be effective behavioral change agents because of their credibility (17) and positionality to influence the opinions, attitudes, beliefs, motivations and behaviors of their clients (18). We hypothesized that if ANC providers gained the necessary knowledge and skills to provide person-centered counseling (Level 2) and were given the opportunity to question their beliefs and attitudes together with an enabling environment (Level 1), they could positively influence the knowledge and attitudes of their clients to abandon the practice (Supplementary file 1).

The level one intervention consisted of making available national policy directives on the role of health care providers in providing FGM prevention and care services, WHO's FGM guidelines and clinical handbook as well as information, education, and communication (IEC) materials. These materials were distributed without any capacity building to accompany their distribution. Level two consisted of an interactive training specifically targeting ANC providers to build their knowledge on FGM, enable them to question their FGM-related values and attitudes and build their skills on counseling for FGM prevention using person-centred communication (19), a component of person-

For peer review only - http://bmjopen.bmj.com/site/about/guidelines.xhtml

centred care, which ensures that the perspectives and preferences of individuals, carers, families and communities are at the center of decisions and that they have the information and support needed to make decisions (20). ANC providers were trained to apply a series of structured steps in which they would: 'Assess' their client's views on FGM, address and challenge her 'Beliefs', encourage 'Change' and together with the client, 'Discuss and Decide' (ABCD).

METHODS

Study Design

This cluster randomized trial applied a type 2 hybrid, effectiveness-implementation design (21) to test the effectiveness of the delivery of a phased intervention package (Level 1 and 2) on knowledge, attitudes and practices among ANC health workers and their clients. This type of implementation research design assesses the effectiveness of the intervention and implementation factors in real world settings. The methodology, analysis plan and reporting conformed to the Consolidated Standards of Reporting Trial (CONSORT) 2010 statement: extension for cluster randomized trials checklist (22). Ethical approval for the master protocol was obtained from the World Health Organization (WHO) Ethical Review Committee (ERC) (#P151/03/2014). Each study country submitted country-specific protocols to local institutional review boards. Ethical approval was obtained in Kenya from the Kenyatta National Hospital/University of Nairobi ERC (P805/09/2019) and the National Commission for Science, Technology, and Innovation (NACOSTI/P/20/5721); in Somalia from the Department of Planning, Policy and Strategic Information, Unit of Research (MOHD/DG: 2/11526/2019); and in Guinea from the *Comité National d'Ethique Pour la Recherche en Santé* (CNERS) (105/CNERS/19).

Participants

Within each study country, two or three sub-national units (regions/counties) were purposively selected according to the following eligibility criteria: (1) FGM prevalence >50% among females 15 -

For peer review only - http://bmjopen.bmj.com/site/about/guidelines.xhtml

49 years old; (2) more than 15 ANC clinics, seeing on average 30 new ANC clients per month and (3) accessibility in terms of security. The unit of randomization was the ANC clinic to avoid having ANC providers in the same clinic in different study arms, which could lead to contamination. In intervention sites, all providers on duty were pre-screened. To ensure participation and follow-up throughout the trial, between one and three ANC providers on duty were enrolled based on a six-month clinic rotation schedule provided by the clinic manager. Ten new clients exiting their first ANC consultation with a participating provider were recruited at each data collection point.

Individual study participants gave verbal informed consent. Data collectors collected data from the ANC providers and their clients in a private and confidential setting. While personally identifiable information was collected from ANC providers to facilitate tracking during the follow-up data collection time points, data were de-identified prior to analysis. No personally identifiable information was collected from ANC clients who were unique at each time point. Participating ANC clients received the equivalent of 5 USD to compensate for their transport costs recognizing that participants consenting to participate might have changed their plans to accommodate the interviews. Given insecurity in carrying cash in Somalia, a mobile phone application was used to transfer the money to participants, an amendment to the original protocol, which was submitted to the ethical review committees.

Randomization and blinding

Based on Ministry of Health (MoH) facility administrative records, all public, primary care facilities (i.e., dispensaries and/or health centers) offering ANC services in the selected regions/counties the average number of new ANC clients seen in November and December 2019 was compiled to create ordered listings of client loads at each of the sites by region/county. Clinics were matched into pairs based on client load so the two busiest would be randomized to different arms and so on. A uniform distribution was used for randomization using the uniform random number function in STATA 17

For peer review only - http://bmjopen.bmj.com/site/about/guidelines.xhtml

(StataCorp Inc., College Station, TX, USA). Study teams organized data collection and intervention trainings based on the randomization lists. Attempts were made to blind clinic managers, ANC providers and their clients to study arm allocation. Since both study arms received the level one intervention component at baseline, and the providers and managers at control sites were unaware of the training that took place at intervention sites, it is conceivable that they were not aware of their study arm. Presumably, intervention clients would assume they were the intervention arm, but they were also not aware of what might have been offered to other sites. ANC clients, however, were completely blinded as to study arm allocation since a distinct set of clients was interviewed at each time point, and they would not be aware of the training the provider had had. Field data collectors were also blinded to study arm allocation as much as possible, although some might have determined intervention arm during the study.

Procedures

Implementation of the study interventions and data collection occurred between August 2020 and September 2021 and was staggered by countries. In the intervention arm, data collection was undertaken at three time points, i.e., at baseline prior to implementing the level one intervention component; at month three, prior to implementing the level two intervention component and at month six. In the control arm, data collection was done at two time points, i.e., at baseline and at month six. Study instruments included one for ANC clients, one for health workers and a health facility checklist completed by clinic managers. Instruments were pretested among ANC clients and providers from non-participating sites in all countries, and country teams provided feedback on the structure and appropriateness of each question prior to finalizing the instruments.

A web-interface electronic data capture system was developed on the Kobo toolbox core system architecture (Kobo Toolbox, Harvard Humanitarian Initiative, Boston, Massachusetts, USA). User accounts were password-protected, and data sent to the server was encrypted in transit using SHA256

For peer review only - http://bmjopen.bmj.com/site/about/guidelines.xhtml

with RSA encryption that met the data security requirements. Personally identifiable information was not collected, and all records were anonymized with unique study numbers. Study instruments for ANC clients were translated from English into ten languages by research team members in consultation with language experts (French, Somali, Swahili, Soussou, Poular, Malinké, Keiyo, Maasai, Marakwet and Tugen) while those for ANC providers and clinic managers were translated into two languages (French and Somali). No backtranslation was performed. Field data collectors and their supervisors spoke the languages in which the questionnaires were administered. Data collection teams participated in a standardized training with WHO/HRP and the research institutions in each country. The level two intervention was implemented by master trainers in each country who had been trained remotely over a three-day period following the WHO PCC for FGM prevention facilitator's manual.

Outcomes

The primary study outcome was delivery of the "ABCD" approach by ANC providers measured by responses from their client using tools developed for this study based on previously validated instruments, including four constructs of the operational definition of person-centered communication (23). We also assessed ANC provider delivery of FGM care services and their utilization of the level one intervention components. Health facility preparedness to offer FGM prevention and care was assessed using a composite score developed for this study. (Supplementary file 2).The secondary self-efficacy outcome was assessed based on a score calculated from a validated tool for measuring general self-efficacy (24) while knowledge, attitudes, and practice (KAP) on FGM prevention and care were measured using an unvalidated KAP questionnaire similar to one used in formative research in Guinea. Study instruments can be found in Supplementary file 3

Statistical analysis

For peer review only - http://bmjopen.bmj.com/site/about/guidelines.xhtml

To have sufficient power (80%) to detect a difference (significance level 5%) between intervention and control arms on the primary study outcome of delivery of the PCC intervention for FGM prevention, 180 ANC clinics, equally divided across the three study countries were recruited and randomized with 1800 new ANC clients (10 per clinic) recruited at baseline and 1800 at six-month follow-up. While similar interventions have resulted in 20% difference between groups (25), a 10% difference (based on an assumed 20% in the control arm and 30% in the intervention arm) was applied to ensure sufficient power to detect a 10% difference and considering the minimal levels of clinical efficacy for such an intervention to be practical. This sample size also allowed for a 10% non-response and/or loss to follow-up rate and accounted for a clustering effect of (ICC=0.20) at clinic level. A relatively high level of clustering was assumed in the sample size calculations to not underestimate the needed sample size. Region/county level was not included in the multilevel model due to the low number of included regions/counties per country (Kenya 3, Guinea 2, Somalia 3) and it would then not be possible to get an accurate estimate of the variance between clusters.

Data were analyzed using STATA 17 software following a per-protocol approach. Data from ANC providers and their clients were analyzed if the clinic had at least one provider with follow up data at all study time points, and in the intervention arm, if the ANC provider present had undergone training on PCC for FGM prevention at month three. Clinics where providers were lost to follow-up were not included in the final analyses. All facility checklists and ANC client exit interviews were conducted as intended except at sites not accessible due to security issues or closed or converted for care of COVID-19 patients during the pandemic. As the study was designed to pre-screen ANC providers at baseline and include in the final analytic sample only those clinics and providers who were available at 3 and 6 months, an intention-to-treat approach was not feasible. Key characteristics of the participating facilities,

For peer review only - http://bmjopen.bmj.com/site/about/guidelines.xhtml

providers and clients were summarized. Providers and clinics that were screened but not eligible are compared in Supplementary file 4.

Continuous variables are presented using mean values, and standard deviation (SD) while categorical variables are summarized as counts (N) with percentages (%). Differences in proportions were analysed for dichotomous outcomes using Fischer's exact test. For outcomes measured as summary scores, comparisons of mean scores are presented across study arms using t-test.

Initial analyses showed that the clustering was negligible at the provider level since most sites only included one provider in the study. Therefore, multilevel regression models were not used to compare outcomes among providers in intervention vs. control arms. However, analyses based on client level outcomes applied multilevel mixed effect logistic regression models to assess differences between the study arms. Multilevel analyses were attempted for the models in which ANC clients reported on provider actions, but given the complexity of the models, convergence problems arose leading to unreliable results. In these cases, results of ordinary models are presented. Linearity was assessed for the continuous covariates included in the regression models using the Box-Tidwell test in Stata.

At month six, a comparison of study outcomes between the intervention and control arms was used to determine the combined effect of both levels of the intervention package. Multilevel multivariable logistic regression analyses for ANC provider outcomes were adjusted for their sex, years of service, FGM status, FGM-related training, any specific training on communication/counseling and PCC, and whether the provider had conducted FGM in the past. Analyses related to ANC client outcomes were adjusted for their age, educational level, FGM status and exposure to level one IEC materials. These variables were determined a priori based on previously published literature. Analyses related to provider actions as reported by clients were adjusted for client characteristics as it was not possible to definitively link a client with a particular provider. Unadjusted analyses are presented for

For peer review only - http://bmjopen.bmj.com/site/about/guidelines.xhtml

outcomes that relate to composite measures based on ANC provider and client responses (e.g., provision of FGM prevention and care services).

To determine the separate effect of the two levels of the intervention package, additional sub-analyses were conducted restricted to the intervention arm. Changes from baseline to month 3 within the intervention arm were used to determine the effect of the level one intervention component while changes from month 3 to month 6 within the same study arm were used to determine the effect of the level two intervention component. The study was not powered for these sub-analyses, however, and these results are presented in Supplementary file 4.

In-country data managers monitored data quality. Periodic data audits were conducted by the WHO/HRP Quantitative Assessment and Data Management team to identify any data collection gaps and data discrepancies requiring follow up by in-country teams. Weekly data monitoring meetings were held between the in-country research teams and WHO/HRP staff during data collection periods to identify, document and resolve any data discrepancies. These were virtual due to the COVID-19 pandemic. Given that there was no prospective follow-up of clients, a Data Safety and Monitoring Board was not established. Instead, local research teams documented and reported any unintended harms and/or protocol deviations to the WHO/HRP study coordination team.

Patient and public involvement statement

Health care providers and members of communities where the practice of FGM is prevalent in the study countries were actively involved in the design and implementation of this study intervention. This included the formative research conducted in Guinea, which identified health care providers as integral members of FGM practicing communities who understand local community beliefs and norms, making them effective change agents. The formative research also found that the health sector can support these health care providers to be effective change agents by incorporating FGM content within

For peer review only - http://bmjopen.bmj.com/site/about/guidelines.xhtml

their training, ensuring accountability to legal and policy standards and promoting FGM abandonment as part of a multi-sectoral approach. Based on this formative work, the PCC training was developed and subsequently piloted among ANC providers in Kenya before being rolled out as part of the multi-country study.

Additionally, the research partners in Guinea, Kenya and Somalia actively engaged health care providers and community members as part of their in-country work towards FGM prevention. In Kenya, as part of mobilization of study participants, community health volunteers in the study counties talked about the study during their community sensitization sessions and invited pregnant women to attend routine ANC sessions where they could be approached for participation in the study. Both health care providers and pregnant women were provided with information about the study, including the burden of the intervention as to time, any risks involved in their participation, the voluntary nature of their participation, and were recruited only after providing informed consent.

At present, study dissemination meetings have been conducted in Kenya and Guinea that have involved the MoH, other stakeholders as well as representatives of health care providers and community members where the study was implemented. In these meetings, the in-country research partners have led the development of policy briefs identifying country-specific results relevant for local research needs, policy development and practice.

Role of the funders

Apart from WHO/HRP, the study funders had no role in study design or implementation. WHO/HRP, in collaboration with in-country research teams, developed the study protocol, provided data management and analytic support, and contributed to interpretation and manuscript writing. WHO/HRP coordinated the successful implementation of this study. The data collection platform was developed and maintained by an outsourced vendor (First Data, LLC, Kenya); data management was

For peer review only - http://bmjopen.bmj.com/site/about/guidelines.xhtml

coordinated by the local implementing partners (CERREGUI, DARS and University of Nairobi) and statistical data analysis was conducted by an external statistician (Dr. Max Petzold, Gothenburg University). All these functions were conducted with utmost integrity following ICH-GCP guidelines. This trial was registered: PACTR201906696419769 (June 3, 2019).

RESULTS

Recruitment and retention

Between August 2020 and September 2021, a total of 180 ANC clinics (i.e, 60 clinics per study country) were enrolled and randomized to intervention and control arms. There was some natural staggering of the start and subsequent data collection dates due to factors, such as weather, COVID-19, Ramadan, and national elections. Data collection periods ranged from three to six weeks in each country at each time point. The time elapsed between the end of one data collection period to the beginning of the next data collection period ranged from three to five months.

In the intervention arm, 216 providers and 900 clients (i.e.., 10 per clinic) were interviewed. Based on a review of clinic rotation schedule to ensure participation of at least one provider from each study clinic throughout the trial, 133 providers were enrolled. In the control arm, 220 providers and 900 clients were interviewed. (Figure 1). At month three, data were collected at 98% (n=88) of the intervention clinics as two clinics in Kenya were inaccessible due to insecurity. One hundred and thirty (98%) ANC providers (at least one from each site) and 880 first visit ANC clients completed the month three questionnaires prior to implementing the Level 2 intervention PCC. No data collection was conducted at the control sites. At month six, 91% (n=163) of ANC clinics (81, intervention and 82, control) had at least one ANC provider (intervention n=110 and control n=122) on duty who was previously enrolled in the study. The client questionnaire was applied to 819 and 810 first visit ANC clients, respectively in the intervention and control sites.

For peer review only - http://bmjopen.bmj.com/site/about/guidelines.xhtml

Characteristics of study sites and participants

The 163 ANC clinics retained to the end of the study, had a mean of four ANC providers (standard deviation, SD: 3) and served on average 155 new ANC clients per month (SD: 127) with a mean catchment population of 36,754 people (SD: 126,082). In 55% (n=89) of clinics, the clinic manager reported that there were no activities promoting FGM prevention in the facilities' catchment area (*Table 1*). These characteristics were not different from the 17 ANC clinics that were enrolled at baseline but that subsequently were not included in the final analysis (Supplementary file 4).

Of the 232 ANC providers who contributed data for analysis at month six, 83% (n=193) were female and their mean age was 36 years (SD: 10 years). They had an average of eight years professional experience (SD: 7 years) and 68% (n=158) had studied up to Diploma level (generally 3 years post-secondary education) with 90% (n=208) identifying as either midwives, nurses, or nurse-midwives. Health cadres were defined by national licensing requirements in each country. Among these providers, at baseline, 63% (n=146) reported that they had not received formal clinical training on FGM prevention and care (*Table 2*). Almost two-thirds (64%, n=149) reported that they had received training on communication/counselling while half (51%, n=118) had received training on person-centered care. Further, 54% (n=126) of female providers reported that they had undergone FGM while overall, 94% (n=217) of providers reported that they had never performed FGM. These characteristics were not different when compared to the ANC providers who were on duty in the 180 ANC clinics enrolled at baseline (Supplementary file 4). The mean age of the 1,800 clients exiting their first ANC visits at baseline was 26 years (SD: 6 years), 47% (n=846) reported not having received any education, and 73% (n=1,320) reported that they had undergone FGM. These characteristics were similar to the 880 and 1,630 first visit ANC clients interviewed at month three (intervention arm only) and month six, respectively (*Table 3*).

For peer review only - http://bmjopen.bmj.com/site/about/guidelines.xhtml

To evaluate potential bias from differential selection of providers receiving the intervention, we assessed differences in baseline characteristics between the 133 ANC providers from intervention facilities who were screened at baseline and received PCC training at month three (i.e., included in the analytic sample) versus the 97 who were screened and did not receive the intervention (i.e., excluded from analytic sample). The reasons for this included the fact that some of the providers had been transferred from the study clinics or could not be released to attend the training so as not to affect service delivery. Both groups were similar in terms of sex, educational level, professional cadre, as well as whether they had undergone or recently performed FGM. However, included providers tended to be slightly younger (by two years on average) and less likely to be of Muslim religion, although the question on religion was not administered for the Somalia sample since all respondents were assumed to be Muslim (Supplementary file 4).

ANC providers implementation of ABCD elements of the PCC approach

Table 4 presents the analysis of study outcomes by arm at month six. Compared to ANC providers in the control arm, those in the intervention arm were nearly nine times as likely to ask their clients if they had undergone FGM (OR: 8.9, 95% CI: 6.9-11.5; p<0.001), nearly ten times as likely to ask their clients' personal beliefs regarding FGM (OR: 9.7, 95% CI: 7.5-12.5; p<0.001), more than nine times as likely to discuss with their clients why FGM should be prevented (OR: 9.2, 95% CI: 7.1-11.9; p<0.001) and nearly eight times as likely to discuss with their clients how FGM could be prevented (OR: 7.7, 95% CI: 6.0-9.9; p<0.001). Further, ANC clients in the intervention arm were nearly seven times as likely to report that they were satisfied with how FGM had been addressed by their provider during the clinic visit compared to those in the control arm (OR: 6.6, 95% CI: 5.1-8.4; p<0.001). In the intervention arm, the mean score of implementing the ABCD elements of the PCC approach was more

For peer review only - http://bmjopen.bmj.com/site/about/guidelines.xhtml

than twice as likely (OR: 2.1, 95% CI: 1.6-2.6; p<0.001) to be higher in the intervention [3.9 (3.8-4.0)] compared to the control arm [1.6 (1.5-1.8)].

ANC clinic preparedness to provide FGM prevention and care services

A significantly higher proportion of ANC clinics in the intervention arm had all correct repornses related to facility preparedness to provide FGM prevention and care services compared to those in the control arm (68% vs. 27%, p<0.001). Additionally, ANC clinics in the intervention arm had a significantly higher mean score for preparedness compared to those in the control arm [3.4 (95% CI: 3.2-3.6) vs. 2.6 (95% CI: 2.4-2.9; p<0.001)].

ANC providers utilizing level one intervention components

A higher proportion of ANC providers in the intervention arm reported having utilized the level one intervention package components compared to those in the control arm (83% vs. 56%, p<0.001). In multivariable analyses, ANC providers in the intervention arm were nine times as likely to report having utilized the level one intervention package components compared to those in the control arm (AOR: 9.3, 95% CI: 4.2-20.8; P<0.001).

ANC providers offering appropriate FGM prevention and care services

At month six, based on a cumulative score to specific questions on provision of appropriate FGM-related prevention and care services, a higher proportion of ANC providers in the intervention arm reported that they had provided FGM prevention and care services correctly compared to those in the control arm (45% vs. 34%, p=0.03).

ANC providers' confidence, self-efficacy, and communication skills

A higher proportion of ANC providers in the intervention arm reported being confident in their knowledge to provide FGM prevention and care services compared to those in the control arm (98% vs. 89%, p=0.005). In multivariable analysis, ANC providers in the intervention arm had more than six

For peer review only - http://bmjopen.bmj.com/site/about/guidelines.xhtml

times the odds of reporting being confident in their knowledge to provide FGM prevention and care services compared to those in the control arm (AOR: 6.3, 95% CI: 1.4-28.9; p=0.02). Self-efficacy was generally high (scores 7.4 – 7.8 out of 8) with no significant difference in high scores between study arms (85% vs. 82%, p=0.36 and OR: 0.8, 95% CI: 0.4-1.6); p= 0.50).

ANC providers' knowledge, attitudes and support for FGM/medicalized FGM

The mean correct scores for FGM-related knowledge were higher among ANC providers in the intervention arm compared to the control arm (2.5, 95% CI: 2.2-2.8 vs. 1.9, 95% CI: 1.7-2.2; p=0.005) but 8% vs. 2% (p=0.16) had correct responses on the FGM-related knowledge questions, showing low knowledge overall, and particularly on the FGM typology. Providers had similarly unsupportive attitudes towards FGM in both groups and similarly unsupportive attitudes about medicalized FGM with most providers reporting that they did not support FGM (82% vs. 85%, p=0.73) and/or medicalized FGM (72% vs. 73, p=0.94%).

ANC clients' support for FGM, intention to have their daughters undergo FGM and being involved in FGM prevention efforts

Compared to those in the control arm, a higher proportion of ANC clients in the intervention arm reported being less supportive of FGM after their month six clinic visit (52% vs. 29%, p<0.001). In multivariable analysis, ANC clients in the intervention arm had more than twice the odds of reporting that they were strongly opposed to FGM (AOR: 2.4, 95% CI: 1.1-5.2; p=0.023, ICC: 0.61). When asked about their support for FGM after the ANC visit compared to before, clients in the intervention arm had more than five times the odds of being less supportive of FGM compared to those in the control arm (OR: 5.4, 95% CI: 2.4-12.4; p<0.001, ICC:0.66). ANC clients in the intervention clinics had lower odds of intending to have their daughters undergo FGM (OR: 0.3, 95% CI: 0.1-0.7; p=0.004, ICC: 0.60) or of wanting a health care provider to perform FGM (OR: 0.2, 95% CI: 0.1-0.5; p<0.001, ICC: 0.54) and

For peer review only - http://bmjopen.bmj.com/site/about/guidelines.xhtml

higher odds of reporting that they wished to be active in FGM prevention (OR: 3.2, 95% CI: 1.6-6.2, p=0.001, ICC: 0.50).

To understand the impact of the level one intervention relative to the level two intervention, a comparison of study outcomes restricted to the intervention arm was done between baseline and month three and between months three and six (Supplementary file 4). Although not statistically powered for this analyses, we found that a significantly higher proportion of ANC clients in the intervention arm reported that their provider had asked about the different PCC components at month three versus basleine and at month six versus month three. Similarly, a significantly higher proportion of ANC clinics in the intervention arm were prepared to provide FGM-related prevention and care services at month three compared to baseline and at month six compared to month three. No statistically signiofiocant differences were seen in the proportion of ANC providers with the secondary outcomes apart from high confidence scores seen between month six and month three. Finally, ANC client outcomes were significantly higher among intervention clients in month three versus baseline and in month six versus month three.

DISCUSSION

The results of this cluster randomized trial show that an intervention to strengthen health facility preparedness while building skills of ANC providers to communicate using a person-centred counselling technique on FGM prevention was effective. ANC providers exposed to the intervention had increased confidence, improved FGM-related knowledge, and effective delivery of FGM prevention and care services. Additionally, ANC clients who had received care from these providers were less supportive of FGM and had reduced intentions to perform FGM on their daughters. This study provides evidence of a practical intervention to engage health care providers in FGM abandonment efforts whilst also providing quality care to FGM survivors. This study provides evidence of how to effectively build the capacity of

For peer review only - http://bmjopen.bmj.com/site/about/guidelines.xhtml

health care providers at primary care to address FGM(26), an area identified as a critical gap during the formative research.

The PCC training modules strengthened ANC providers' skills on FGM prevention and care and helped to clarify their beliefs and attitudes, which are key drivers of FGM (27). We did not find notable changes in knowledge and attitudes among ANC providers. The knowledge scores overall were low, and upon further investigation, it appears that questions on typology captured through visually drawn images on a tablet device were consistently answered incorrectly. These results perhaps show measurement and knowledge limitations but do not necessarily relate to service provision or quality of care. Attitudes in the intervention and control groups were generally unsupportive of FGM and do not appear to be heavily impacted by the training intervention. Exposure to the intervention package also did not improve ANC providers' self-efficacy towards FGM prevention and care. This may be related to the lack of support for FGM and/or its medicalization and high self-efficacy among nearly all providers throughout the study in both study arms, a finding that was also noted in formative research conducted in Guinea (28,29). In the formative phase, while the vast majority of health workers were opposed to the practice, 38% also felt that FGM limited promiscuity and 7% believed that it was a good practice, showing ambivalence and complexity in attitudes about FGM among health providers. Other studies have found that some providers support the perpetuation of the practice and even planned to have their own daughters undergo FGM or to perform it on their clients(30).

The findings in this study underscore the importance of addressing values and attitudes of both providers and clients as a means of achieving positive behavioral change. Changes observed among ANC providers were sustained across the study duration and ultimately, and importantly, resulted in reported changes in attitudes and intentions of their clients. However, this study design did not allow us

For peer review only - http://bmjopen.bmj.com/site/about/guidelines.xhtml

to determine whether the attitudinal changes observed among ANC clients were sustained after their clinic visit or translated into positive change in FGM prevention.

The application of these study results into programming will need to consider several factors. Firstly, the study sites were primary care facilities located in high FGM prevalence settings. The results of this intervention may not be generalizable to settings where FGM is less prevalent or to settings other than primary care. Secondly, first ANC visits are not typical of other health visits since the consultation is generally longer with a greater focus on health promotion messaging. While this is an ideal setting for implementing such an intervention, its application to other health settings and among other population groups is not known. During scale up, if the PCC approach is applied among clients seeking other sexual and reproductive health services or parents bringing their children to child immunization and wellness visits, it will be important to consider time requirements for the delivery of the 'ABCD' steps, especially in high volume clinic settings.

Thirdly, while the study found a positive impact of the PCC training on health care providers' delivery of person-centred FGM prevention counselling, the continuity and quality of FGM prevention counselling in the long-term is not known. Specifically, it will be important to assess subsequently whether providers will continue to provide prevention counselling on an ongoing basis, whether they will share their learnings with family and community members and whether clients will follow through with their intentions to not have their daughters undergo FGM. It may be important to include a supervisory mentorship component to ensure implementation of this intervention (31) in order to strengthen PCC communication practice and quality.

Limitations

For peer review only - http://bmjopen.bmj.com/site/about/guidelines.xhtml

The implementation of this multi-country study was not without challenges and limitations. First, initiation of field data collection activities was delayed by the global COVID-19 pandemic in 2020 – 2021 and required some modification to trainings of the data collection teams, the master trainers and the ANC providers receiving the PCC intervention. This may have impacted the overall effectiveness of the intervention.

Second, to attempt to ensure participation of at least one provider at each site, all providers were pre-screened at baseline and clinic rotation schedules determined enrollment into the study. Selection bias might have been introduced through this process. The exploratory analysis to assess for selection and attrition bias from the pre-screen step, did not reveal significant differences between included and excluded health workers except for slightly lower age (Supplementary file 4),and a per protocol analysis wasrequired, but it is possible that differences in other unmeasured factors related to the clinics and providers might have biased the results. Findings from a process evaluation conducted as part of this study will provide additional insights on the feasibility, acceptability, appropriateness, and fidelity of the intervention implementation in these contextual settings to inform further implementation and scale up.

Third, we did not perform adjustment for multiple testing in our analysis given that the different tests are interpreted separately and no overall conclusion will be stated. Given that the null hypotheses of no differences are true, we estimate that the overall type one error rate is higher than the individual test level of 0.05. In terms of assumptions regarding clustering, sample size was calculated based on an ICC of 0.20. However, the observed ICC:s were all above 0.50 leading to statistically conservative conclusions of the non-significant results due to being under-powered to find an association.

Finally, we acknowledge that there are many factors that could impact FGM-related decision-making and a positive and impactful interaction with a respected health care provider might not be

For peer review only - http://bmjopen.bmj.com/site/about/guidelines.xhtml

sufficient to lead to actual changes in community behavior. However, the study design enabled us to compare similar sites to identify the relative effect of this approach since both intervention and control sites would be exposed to similar factors, and clients at these sites would face similar complexities in decision-making.

Conclusion

In conclusion, this study highlights the importance of addressing the values and beliefs of health care providers working at primary care level, who are subject to social norms around FGM that may conflict with medical ethics and national laws and policies as an intermediary step in preventing FGM. Empowering these health care providers with communication skills and engaging them as opinion leaders can be impactful in changing their clients' attitudes towards FGM. In conjunction with FGM prevention activities in other sectors, this intervention can contribute to positive change if brought to scale.

For peer review only - http://bmjopen.bmj.com/site/about/guidelines.xhtml

DECLARATIONS

Contributors

WA and CP conceptualized the study and prepared the protocol in collaboration with VM, KS, PN, TE, MDB, AMS, AD(1) and MAA. MDB, AMS, AOS, PN, TE, JMK, AD(1) and MAA provided oversight over study implementation while AD(2), JK and SA monitored data quality in countries and KN and SST monitored data quality across countries. VM prepared the first draft of the manuscript with input from WA and CP, the responsible officer of the study at WHO/HRP. MP developed the statistical analysis plan and conducted data analysis. KS coordinated the development of the PCC for FGM prevention training. KS, PN, TE, JMK, JK, MDB, AMS, AOS, AD(1), AD(2), SA, and MAA contributed to and reviewed the manuscript for proper intellectual content. All authors read and approved the final draft of this manuscript.

Declaration of interests

The authors declare that they have no competing interests.

Data sharing

De-identified dataset will be retained in the WHO HRP electronic archival system. Any use of the de-identified analytic dataset for secondary research purposes will be governed by the WHO data use regulation. Request for data dictionary and for dataset may be sent to pallittoc@who.int

Acknowledgements

The authors would like to acknowledge the funders, the WHO country office colleagues, Dr Bernadette Dramou, Dr Cécé-Vieux Kolie, Dr Joyce Lavussa, Ms Matilda Cherono, Ms Asia Ahmed Osman in the study countries as well as MoH and national FGM stakeholders in the development of the research protocol, implementation of study interventions and field data collection. We also thank Professor Joanna Schellenberg of the London School of Hygiene and Tropical Medicine and Dr Christina

For peer review only - http://bmjopen.bmj.com/site/about/guidelines.xhtml

Atchison of Imperial College London for their input in conceptualizing the study as well as Dr Leyla Hussein for supporting pilot testing of the PCC intervention.

Funding

This work received funding from the Governments of Norway and the United Kingdom of Great Britain and Northern Ireland as well as the UNDP-UNFPA-UNICEF-WHO-World Bank Special Programme of Research, Development and Research Training in Human Reproduction (HRP), a cosponsored programme executed by the World Health Organization (WHO).

For peer review only

Ethics approval:

The following research ethics committees approved the protocol:

1. World Health Organization (WHO) Ethical Review Committee (ERC) (#P151/03/2014).

2. Kenya: Kenyatta National Hospital/University of Nairobi ERC (P805/09/2019) and the National Commission for Science, Technology, and Innovation (NACOSTI/P/20/5721)

3. Somalia: the Department of Planning, Policy and Strategic Information, Unit of Research (MOHD/DG: 2/11526/2019)

4. Guinea: the Comité National d'Ethique Pour la Recherche en Santé (CNERS) (105/CNERS/19).

Disclaimer

The named authors alone are responsible for the views expressed in this publication and do not necessarily represent the decisions or the policies of the UNDP-UNFPA-UNICEF-WHO-World Bank Special Programme of Research, Development and Research Training in Human Reproduction (HRP) or the World Health Organization (WHO).

For peer review only - http://bmjopen.bmj.com/site/about/guidelines.xhtml

For peer review only

For peer review only - http://bmjopen.bmj.com/site/about/guidelines.xhtml

REFERENCES

1. United Nations General Assembly, Intensifying global efforts for the elimination of female genital mutilations : resolution/ / adopted by the United Nations General Assembly, 5 March 2013, A/RES/67/146, available at: https://www.refworld.org/docid/51e67bc64.html [accessed 4 May 2022]
2. World Health Assembly, WHA resolution 61.16. 2008. United Nations: New York. 2008. Available at https://apps.who.int/gb/ebwha/pdf_files/WHA61-REC1/A61_REC1-en.pdf , New York. 2008.
3. Eliminating female genital mutilation: an interagency statement-OHCHR, UNAIDS, UNDP, UNECA, UNESCO, UNFPA, UNHCR, UNICEF, UNIFEM, WHO. WHO: Geneva.World Health Organization, 2008.
4. World Health Organization. The World Health Report: 2000 :health systems : improving performance [Internet]. Geneva: World Health Organization; 2000. Available from: https://apps.who.int/iris/handle/10665/42281
5. Pallitto CC, Ahmed W. The role of the health sector in contributing to the abandonment of female genital mutilation. Med (N Y) [Internet]. 2021 May 14 [cited 2023 May 15];2(5):485–9. Available from: https://pubmed.ncbi.nlm.nih.gov/35590230/
6. Kimani S, Esho T, Kimani V, Muniu S, Kamau J, Kigondu C, et al. Female Genital Mutilation/Cutting: Innovative Training Approach for Nurse-Midwives in High Prevalent Settings. Obstet Gynecol Int [Internet]. 2018 [cited 2023 May 15];2018. Available from: https://pubmed.ncbi.nlm.nih.gov/29736171/
7. Johansen REB, Diop NJ, Laverack G, Leye E. What works and what does not: a discussion of popular approaches for the abandonment of female genital mutilation. Obstet Gynecol Int [Internet]. 2013 [cited 2023 May 15];2013:1–10. Available from: https://pubmed.ncbi.nlm.nih.gov/23737795/
8. Njue C, Askew I. Medicalization of female genital cutting among the Abagusii in Nyanza Province, Kenya. Reprod Health [Internet]. 2004 Jan 1 [cited 2023 May 15]; Available from: https://knowledgecommons.popcouncil.org/departments_sbsr-rh/32
9. Shell-Duncan B, Gathara D, Moore Z. Female genital mutilation/cutting in Kenya: Is change taking place? Descriptive statistics from four waves of Demographic and Health Surveys. Reprod Health [Internet]. 2017 Jan 1 [cited 2023 May 15]; Available from: https://knowledgecommons.popcouncil.org/departments_sbsr-rh/596
10. Umar AS, Oche OM. Medicalization of female genital mutilation among professional health care workers in a referral hospital, north-western Nigeria. J Reprod Biol Health. 2014;2(1):2.
11. Doucet MH, Pallitto C, Groleau D. Understanding the motivations of health-care providers in performing female genital mutilation: an integrative review of the literature. Reprod Health [Internet]. 2017 Mar 23 [cited 2023 May 15];14(1):46. Available from: https://pubmed.ncbi.nlm.nih.gov/28335771/
12. World Health Organization. Female Genital Mutilation: A Resource Kitkit for the Health Sector. WHO: Geneva, 2021, available at https://srhr.org/fgmresources/https://srhr.org/fgmresources/ - Google Search [Internet]. [cited 2023 May 15].
13. World Health Organization. Reproductive Health and Research. WHO guidelines on the management of health complications from female genital mutilation. :47.
14. Ahmed W, Mochache V, Stein K, Ndavi P, Esho T, Balde MD, et al. A hybrid, effectiveness-implementation research study protocol targeting antenatal care providers to provide female

For peer review only - http://bmjopen.bmj.com/site/about/guidelines.xhtml

genital mutilation prevention and care services in Guinea, Kenya and Somalia. BMC Health Serv Res [Internet]. 2021 Dec 1 [cited 2023 May 15];21(1). Available from: https://pubmed.ncbi.nlm.nih.gov/33522926/

15. National Bureau of Statistics. Republic of Kenya. Kenya Demographic and Health Survey 2014. 2015 [cited 2023 May 15]; Available from: www.DHSprogram.com.
16. NHWA Web portal [Internet]. [cited 2023 May 15]. Available from: https://apps.who.int/nhwaportal/Home/Welcome?ReturnUrl=%2Fnhwaportal%2FHome%2FIndex
17. Boone Tim, Reilly Anthony J., Sashkin M. SOCIAL LEARNING THEORY Albert Bandura Englewood Cliffs, N.J.: Prentice-Hall, 1977. 247 pp., paperbound. Group & Organization Studies [Internet]. 1977 Sep 1;2(3):384–5. Available from: https://doi.org/10.1177/105960117700200317
18. Valente TW, Pumpuang P. Identifying opinion leaders to promote behavior change. Health Educ Behav [Internet]. 2007 Dec [cited 2023 May 15];34(6):881–96. Available from: https://pubmed.ncbi.nlm.nih.gov/17602096/
19. Person-centred communication for female genital mutilation prevention: facilitator's manual. WHO World Health Organization: Geneva, 2022
20. World Health Organization. WHO global strategy on people-centred and integrated health services: interim report [Internet]. Geneva: World Health Organization; 2015. Available from: https://apps.who.int/iris/handle/10665/155002
21. Peters DH, Adam T, Alonge O, Agyepong IA, Tran N. Republished research: Implementation research: what it is and how to do it. Br J Sports Med. 2014 Apr 23;48(8):731–6.
22. Campbell MK, Piaggio G, Elbourne DR, Altman DG. Consort 2010 statement: Extension to cluster randomised trials. BMJ (Online). 2012 Nov 3;345(7881).
23. Epstein RM, Franks P, Fiscella K, Shields CG, Meldrum SC, Kravitz RL, et al. Measuring patient-centered communication in patient-physician consultations: theoretical and practical issues. Soc Sci Med [Internet]. 2005 Oct [cited 2023 May 15];61(7):1516–28. Available from: https://pubmed.ncbi.nlm.nih.gov/16005784/
24. Chen G, Gully SM, Eden D. Validation of a New General Self-Efficacy Scale. Organ Res Methods [Internet]. 2001 Jan 1;4(1):62–83. Available from: https://doi.org/10.1177/109442810141004
25. Keeley RD, Burke BL, Brody D, Dimidjian S, Engel M, Emsermann C, et al. Training to use motivational interviewing techniques for depression: a cluster randomized trial. J Am Board Fam Med [Internet]. 2014 Sep 1 [cited 2023 May 15];27(5):621–36. Available from: https://pubmed.ncbi.nlm.nih.gov/25201932/
26. Balfour J, Abdulcadir J, Say L, Hindin MJ. Interventions for healthcare providers to improve treatment and prevention of female genital mutilation: a systematic review. BMC Health Serv Res [Internet]. 2016 Aug 19 [cited 2023 May 15];16(1). Available from: https://pubmed.ncbi.nlm.nih.gov/27542732/
27. Kimani S, Okondo C, Muteshi-Strachan J, Guyo J. Quality of services offered to women with female genital mutilation across health facilities in a Kenyan County. BMC Health Serv Res [Internet]. 2022 Dec 1 [cited 2023 May 15];22(1). Available from: https://pubmed.ncbi.nlm.nih.gov/35525954/
28. Balde MD, O'Neill S, Sall AO, Balde MB, Soumah AM, Diallo BA, et al. Attitudes of health care providers regarding female genital mutilation and its medicalization in Guinea. PLoS One

For peer review only - http://bmjopen.bmj.com/site/about/guidelines.xhtml

[Internet]. 2021 May 1 [cited 2023 May 15];16(5). Available from: https://pubmed.ncbi.nlm.nih.gov/33983949/

29. Balde MD, Soumah AM, Diallo A, Sall AO, Mochache V, Ahmed W, et al. Involving the health sector in the prevention and care of female genital mutilation: results from formative research in Guinea. Reprod Health. 2022 Dec 1;19(1).

30. Kaplan A, Hechavarría S, Bernal M, Bonhoure I. Knowledge, attitudes and practices of female genital mutilation/cutting among health care professionals in The Gambia: a multiethnic study. BMC Public Health [Internet]. 2013 [cited 2023 May 15];13(1). Available from: https://pubmed.ncbi.nlm.nih.gov/24040762/

31. Pantoja T, Opiyo N, Lewin S, Paulsen E, Ciapponi A, Wiysonge CS, et al. Implementation strategies for health systems in low-income countries: an overview of systematic reviews. Cochrane Database Syst Rev [Internet]. 2017 Sep 12 [cited 2023 May 15];9(9). Available from: https://pubmed.ncbi.nlm.nih.gov/28895659/

For peer review only

TABLES & FIGURES:

TABLES & FIGURES:

Figure 1: Study CONSORT Diagram

Table 1: Characteristics of ANC clinics included in month six analyses

Table 2: Characteristics of ANC providers included in the month six analyses

Table 3: Characteristics of ANC clients interviewed at each time point

Table 4: Analysis of study outcomes

SUPPLEMENTARY FILES

Supplementary file 1: Theory of change framework

Supplementary file 2: Measurement of study outcomes

Supplementary file 3: Data collection instruments

Supplementary file 4: Additional analyses (Appendices 1 – 3)

For peer review only

For peer review only - http://bmjopen.bmj.com/site/about/guidelines.xhtml

Table 1: Characteristics of ANC clinics included in month six analyses

Characteristics	Overall (n=163*)	Intervention (n=82)	Control (n=81)
Number of ANC providers	Mean 4 (SD: 3) Median 3 (1-14, IQR 3)	Mean 4 (SD: 2) Median 3 (1-11, IQR 3)	Mean 4 (SD: 3) Median 3 (1-14, IQR 4)
Average number of ANC clients/month	Mean 150 (SD: 127) Median 118 (3-664, IQR 141)	Mean 148 (SD: 121) Median 117 (3-500, IQR 143)	Mean 152 (SD: 133) Median 120 (3-664, IQR 140)
MoH supervisory visits in the past year	Mean 4 (SD: 3) Median 3 (0-18, IQR 2)	Mean 4 (SD: 3) Median 4 (1-18, IQR 1)	Mean 4 (SD: 3) Median 3 (0-12, IQR 2
Size of catchment population served	Mean 36,754 (SD: 126,082) Median 15,972 (1,000-1,458,000, IQR 24,332)	Mean 23,649 (SD: 35,873) Median 16,022 (1,000-290,000, IQR 22,361	Mean 50,020 (SD: 174,739) Median 15,551 (1,000-1,458,000, IQR 25,544
Presence of anti-FGM activities in the catchment area			
Yes	74 (45%)	43 (52%)	31 (38%)
No	89 (55%)	39 (48%)	50 (62%)
Presence of pro-FGM activities in the catchment area			
Yes	21 (13%)	12 (15%)	9 (11%)
No	140 (86%)	68 (83%)	72 (89%)
Don't Know	2 (1%)	2 (2%)	0 (0%)

* Total of 17 ANC clinics not included: 16 clinics were excluded (7 intervention and 9 control) due to loss-to-follow up (LTFU) of ANC provider i.e., the clinics did not have at least one ANC provider present across all study time points while one ANC clinic in Kenya was never visited at subsequent time points due to issues with insecurity. An ANC provider from one of the clinics in Kenya that had been inaccessible due to insecurity attended the PCC training and was subsequently interviewed.

For peer review only

For peer review only - http://bmjopen.bmj.com/site/about/guidelines.xhtml

Table 2: Characteristics of ANC providers included in the month six analyses

Characteristics	Overall (n=232)	Intervention (n= 115)	Control (n=117)
Age	Mean 36 (SD: 10) Median 34 (20-65, IQR 15)	Mean 35 (SD: 10) Median 33 (20-59, IQR 14)	Mean 37 (SD:11) Median 35 (20-65, IQR 16)
Years of professional experience	Mean 8 (SD: 7) Median 6 (1-39, IQR 7)	Mean 8 (SD:7) Median 6 (1-30, IQR 8)	Mean 8 (SD:7) Median 6 (1-39, IQR 7)
Sex			
Female	193 (83%)	95 (83%)	98 (84%)
Highest educational level			
Certificate	21 (5%)	12 (10%)	9 (8%)
Diploma	158 (68%)	72 (63%)	86 (74%)
Bachelors	44 (19%)	27 (24%)	17 (15%)
Masters & above	1 (0.4%)	0 (0%)	1 (1%)
Other[#]	8 (3%)	4 (3%)	4 (3%)
Current professional role/title			
Midwife	103 (44%)	53 (46%)	50 (43%)
Nurse	51 (22%)	25 (22%)	26 (22%)
Nurse-Midwife	54 (23%)	27 (24%)	27 (23%)
Other	24 (10%)	10 (9%)	14 (12%)
Received formal training on FGM during clinical training			
Yes	85 (37%)	44 (38%)	41 (35%)
No	146 (63%)	71 (62%)	75 (64%)
Don't Know	1 (0.4%)	0 (0%)	1 (1%)
Timing of clinical training on FGM			
Pre-service	33 (14%)	18 (16%)	15 (13%)
In-service	45 (19%)	22 (19%)	23 (20%)
Both pre- and in-service	7 (3%)	4 (4%)	3 (3%)
Received formal training on communication/counselling			
Yes	149 (64%)	76 (66%)	73 (62%)
No	83 (36%)	39 (34%)	44 (38%)
Received formal training on person-centered care			
Yes	118 (51%)	58 (50%)	60 (51%)
No	113 (56%)	56 (49%)	57 (49%)
Don't know	1 (0.4%)	1 (1%)	0 (0%)
Undergone FGM			

For peer review only - http://bmjopen.bmj.com/site/about/guidelines.xhtml

Yes	126 (54%)	65 (57%)	61 (52%)
No	63 (27%)	27 (24%)	36 (31%)
Don't know	2 (1%)	2 (2%)	0 (0%)
Refused to answer	2 (1%)	1 (1%)	1 (1%)
Conducted FGM			
Yes	15 (7%)	9 (8%)	6 (5%)
Conducted FGM on a girl <18 years			
Yes	14 (6%)	8 (7%)	6 (5%)

For peer review only

For peer review only - http://bmjopen.bmj.com/site/about/guidelines.xhtml

Table 3: Characteristics of ANC clients interviewed at each time point

Characteristics	ANC clients interviewed at Baseline			ANC clients interviewed at Month 3	ANC clients interviewed at Month 6		
	**Overall (n=1800)**	**Intervention (n=900)**	**Control (n=900)**	**Intervention only (n=880)**	**Overall (n=1759)**	**Intervention (n=879)**	**Control (n=880)**
Age	Mean 26 (SD: 6) Median 25 (15-45, IQR 10)	Mean 25 (SD: 6) Median 25 (15-45, IQR 10)	Mean 26 (SD: 6) Median 25 (15-45, IQR 10)	Mean 26 (SD 6) Median 25 (15-45, IQR 10)	Mean 26 (SD: 6) Median 25 (15-45, IQR 9)	Mean 26 (SD: 6) Median 25 (15-45, IQR 9)	Mean 26 (SD: 6) Median 25 (15-45, IQR 10)
Highest educational level							
None	840 (47%)	407 (45%)	433 (48%)	439 (50%)	806 (46%)	384 (44%)	422 (47%)
Primary	484 (27%)	231 (26%)	253 (28%)	239 (27%)	553 (31%)	278 (32%)	275 (31%)
Secondary	331 (18%)	171 (19%)	160 (18%)	157 (18%)	306 (17%)	160 (18%)	146 (16%)
University	95 (5%)	61 (7%)	34 (4%)	25 (3%)	67 (4%)	34 (4%)	33 (4%)
Other[#]	50 (3%)	30 (3%)	20 (25)	20 (2%)	37 (2%)	23 (3%)	14 (2%)
Have you undergone FGM?							
Yes	1320 (73%)	677 (75%)	643 (71%)	645 (73%)	1321 (75%)	655 (75%)	666 (75%)
No	452 (25%)	209 (23%)	243 (27%)	224 (25%)	420 (24%)	206 (23%)	214 (24%)
Don't know	12 (1%)	10 (1%)	2 (0.2%)	5 (1%)	21 (1%)	13 (2%)	8 (1%)
Refused to answer	16 (1%)	4 (0.4%)	12 (1%)	6 (1%)	7 (0.4%)	5 (1%)	2 (0.2%)

For peer review only

For peer review only

For peer review only - http://bmjopen.bmj.com/site/about/guidelines.xhtml

Table 4: Analysis of study outcomes

**Primary Outcomes**					
**ANC clients reporting that their provider implemented components of PCC for FGM prevention approach**					
	**Intervention (n=819)**	**Control (n=810)**	**Adjusted OR# (95% CI)**	**P-value**	**ICC**
Provider asked client if they have undergone FGM	634 (77%)	245 (30%)	8.9 (6.9-11.5)	<0.001	N/A
Provider asked client about the client's personal beliefs regarding FGM	616 (75%)	217 (27%)	9.7 (7.5-12.5)	<0.001	N/A
Provider discussed with client why FGM should be prevented	629 (77%)	244 (30%)	9.2 (7.1-11.9)	<0.001	N/A
Provider discussed with client how FGM could be prevented	592 (72%)	232 (29%)	7.7 (6.0-9.9)	<0.001	N/A
Client satisfied with how FGM was addressed by provider during clinic visit	684 (84%)	348 (43%)	6.6 (5.1-8.4)	<0.001	N/A
			Difference in mean scores (95% CI)		
Mean score of implementing PCC approach (out of 5)	3.9 (3.8-4.0)	1.6 (1.5-1.7)	2.3 (2.1-2.5)	<0.001	N/A
Mean score of PCC + appropriate FGM prevention and care (out of 8)	6.2 (5.9-6.6)	3.7 (3.2-4.1)	2.6 (2.0-3.2)	<0.001	N/A
**ANC clinic preparedness to offer FGM prevention and care services**					
	**Intervention (n=82)**	**Control (n=81)**	**Adjusted OR& (95% CI)**	**P-value**	**ICC**
Clinics with ALL correct responses for preparedness	56 (68%)	22 (27%)	-	<0.001	N/A
Mean score of clinic preparedness (out of 4)	3.4 (3.2-3.6)	2.6 (2.4-2.9)	-	<0.001	N/A
	**Intervention (n=115)**	**Control (n=117)**	**Adjusted OR& (95% CI)**	**P-value**	**ICC**
Providers using level 1 intervention package	96 (83%)	65 (56%)	9.3 (4.2-20.8)	<0.001	N/A
**Secondary Outcomes***					
Providers with appropriate interpersonal communication skills	74 (64%)	68 (58%)	1.7 (1.0-3.0)	0.060	N/A
Providers with high self-efficacy	86 (75%)	99 (85%)	0.8 (0.4-1.6)	0.453	N/A
Providers reporting less supportive attitudes towards FGM	76 (66%)	85 (73%)	1.0 (0.5-1.8)	0.901	N/A
Providers with high confidence scores	103 (90%)	104 (89%)	6.3 (1.4-28.9)	0.018	N/A
Providers not supportive of FGM	100 (87%)	114 (97%)	0.8 (0.2-3.7)	0.726	N/A
Providers not supportive of medicalized FGM	104 (90%)	116 (99%)	1.1 (0.1-22.1)	0.938	N/A
Providers with correct FGM-related knowledge responses	8 (8%)	1 (2%)	5.0 (0.5-47.8)	0.16	N/A
Mean score of FGM-related knowledge (out of 6)	2.5 (2.2-2.8)	1.9 (1.7-2.2)	-	0.005	N/A
**Other ANC client outcomes****					
	**Intervention (n=819)**	**Control (n=810)**	**Adjusted OR& (95% CI)**	**P-value**	**ICC**
Clients reporting less support for FGM after ANC clinic visit	424 (52%)	237 (29%)	5.4 (2.4-12.4)	<0.001	0.66
Clients reporting that they were strongly opposed to FGM	498 (61%)	382 (47%)	2.4 (1.1-5.2)	0.023	0.62

For peer review only - http://bmjopen.bmj.com/site/about/guidelines.xhtml

Clients reporting that they intend to have their daughters cut	96 (12%)	209 (26%)	0.3 (0.1-0.7)	0.004	0.60
Clients reporting that they would prefer health care provider to cut daughters	53 (7%)	139 (17%)	0.2 (0.1-0.5)	<0.001	0.54
Clients wishing to be active in FGM prevention	677 (83%)	535 (66%)	3.2 (1.6-6.2)	0.001	0.50

ICC = Intra-cluster Correlation Coefficient
[#]Single-level multi-variable adjusted models
[&]Multi-level multi-variable adjusted models
*Provider outcomes adjusted for sex, years of service, FGM status, FGM-related training, any specific training on communication/counseling and PCC, and whether the provider had conducted FGM in the past
** Client outcomes adjusted for age, educational level, FGM status and exposure to level one IEC materials

For peer review only

For peer review only

For peer review only - http://bmjopen.bmj.com/site/about/guidelines.xhtml

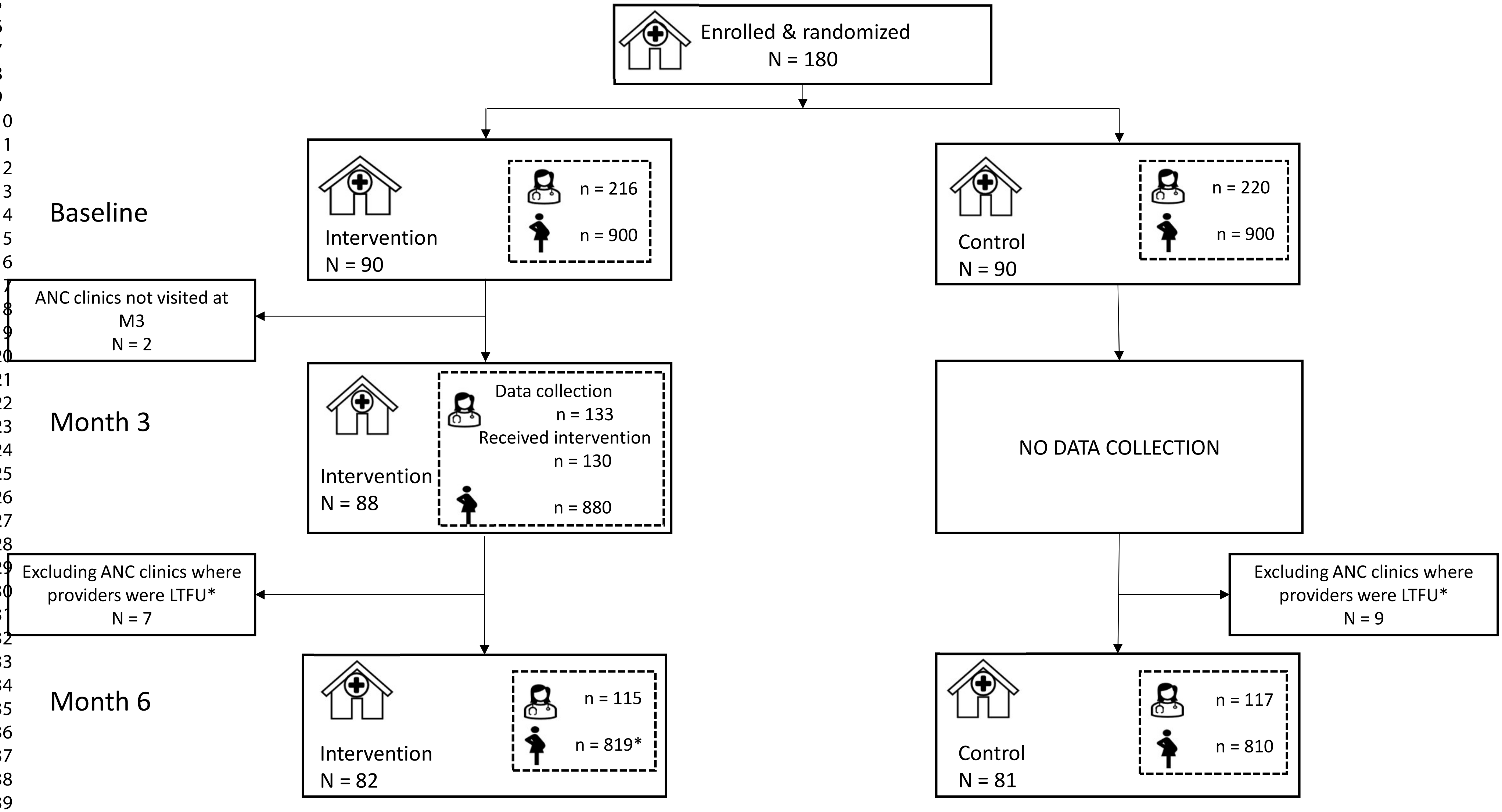
Enrolled & randomized
N = 180
Baseline
Intervention
N = 90
n = 216
n = 900
Control
N = 90
n = 220
n = 900
ANC clinics not visited at M3
N = 2
Month 3
Intervention
N = 88
Data collection
n = 133
Received intervention
n = 130
n = 880
NO DATA COLLECTION
Excluding ANC clinics where providers were LTFU*
N = 7
Excluding ANC clinics where providers were LTFU*
N = 9
Month 6
Intervention
N = 82
n = 115
n = 819*
Control
N = 81
n = 117
n = 810

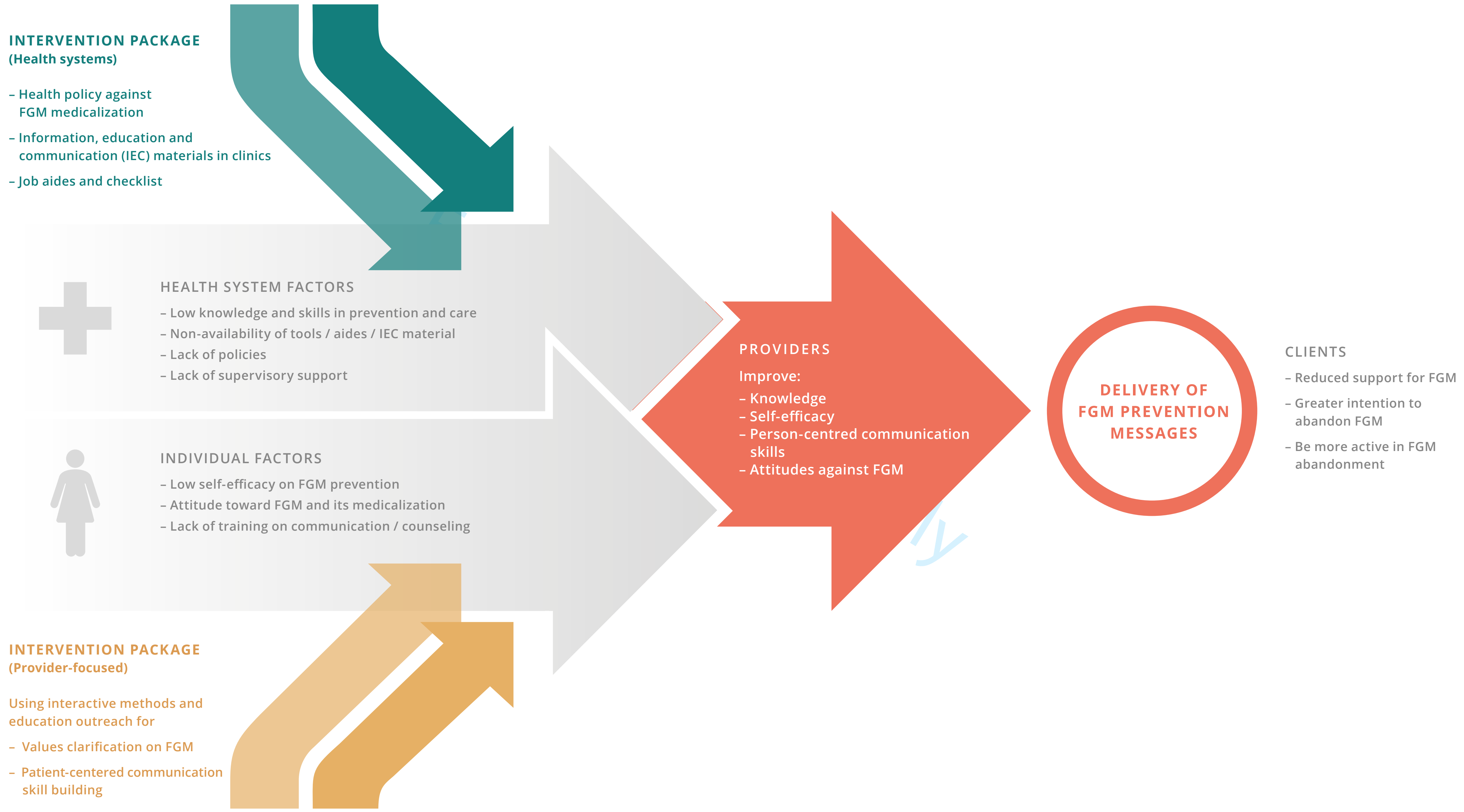
INTERVENTION PACKAGE
(Health systems)
– Health policy against FGM medicalization
– Information, education and communication (IEC) materials in clinics
– Job aides and checklist
HEALTH SYSTEM FACTORS
– Low knowledge and skills in prevention and care
– Non-availability of tools / aides / IEC material
– Lack of policies
– Lack of supervisory support
INDIVIDUAL FACTORS
– Low self-efficacy on FGM prevention
– Attitude toward FGM and its medicalization
– Lack of training on communication / counseling
INTERVENTION PACKAGE
(Provider-focused)
Using interactive methods and education outreach for
– Values clarification on FGM
– Patient-centered communication skill building
PROVIDERS
Improve:
– Knowledge
– Self-efficacy
– Person-centred communication skills
– Attitudes against FGM
DELIVERY OF FGM PREVENTION MESSAGES
CLIENTS
– Reduced support for FGM
– Greater intention to abandon FGM
– Be more active in FGM abandonment

Supplementary file 2 : Measurement of study outcomes

1. ***Primary Outcome:*** *Health facility preparedness to provide FGM prevention and care services.*

Outcome definition*: Cumulative score based on affirmative responses to Q9a, Q10a, Q11a & Q12a on the CHK form (see below).*

Q9. Is there an MoH policy on FGM posted on the wall?

Yes

No

Q9a. If yes, is it placed where health care providers can see/read it e.g., bulletin board?

Yes

No

Q10. Are there WHO FGM prevention posters on the wall of the consultation room and/or waiting room?

Yes

No

Q10a. If yes, are they placed in a place where ANC clients can see them?

Yes

No

Q11. Is there a WHO clinical handbook in the ANC consultation room?

Yes

No

Q11a If yes, is it placed where ANC providers can see/use it?

Yes

No

Q12. Is there an FGM ABCD guide in the ANC consultation room?

Yes

No

Q12a. If yes, is it placed where ANC providers can see/use it?

Yes

No

2. ***Primary outcome: ANC provider utilization of Level 1 package components***

Outcome definition*: Affirmative response on Q40 of HCP form (see below).*

Q40. Have you referred to the WHO Clinical Handbook on FGM?

Yes

No, available but not referred

No, not available

Don't know

3. ***Primary outcome: Provision of FGM-related care after PCC training***

Outcome definition: *Cumulative score based on affirmative responses (Provision of FGM-related care (after PCC training) either 'Always' or 'Often') on Q22, Q24 & Q25 on the HCP form (see below).*

Q22. How often do you discourage a pregnant woman expecting to have a girl, or one having a girl at the age of cutting, from having her daughter cut?

Always

Often

Sometimes

Rarely

For peer review only

Never
Rarely
Refused to answer

Q24. How often do you look for female genital mutilation when performing a gynecological examination of the vulva?

Always
Often
Sometimes
Rarely
Never
Rarely
Refused to answer

Q25. How often do you record female genital mutilation in the woman's medical file if you are aware that she has undergone FGM?

Always
Often
Sometimes
Rarely
Never
Rarely
Refused to answer

4. Primary Outcome: Delivery of PCC 'ABCD' package

Outcome definition: *Cumulative score based on affirmative responses on Q5, Q7, Q8, Q9 & Q12 on the EXT form.*

Q5. Did the ANC provider ask if you have undergone FGM?

Yes
No
Don't know
Refused

Q7. Did the ANC provider ask about your personal belief regarding FGM?

Yes
No
Don't know
Refused

Q8. Did the ANC provider discuss why FGM should be prevented?

Yes
No
Don't know
Refused

Q9. Did the ANC provider discuss how FGM could be prevented?

Yes
No
Don't know
Refused

Q12. Are you satisfied with how FGM was addressed during your visit with your ANC provider today?

Yes
No
Don't know
Refused

For peer review only

5. ***Secondary Outcome: Improved knowledge about FGM***

Outcome definition: *Cumulative score based on correct responses to Q4 + affirmative responses to Q5 & Q7 of the HCP form.*

Q4. Please provide the WHO classification for the following images

Type I
Type II
Type III
Type IV
Don't Know
Other

Q5. Do you know of any health complications arising from female genital mutilation?

Yes
No

Q7. Are you aware of any existing WHO tools/guidance on FGM prevention and care?

Yes
No

6. ***Secondary Outcome: Improved interpersonal communication skills***

Outcome definition: *Cumulative score based on positive responses ("Always or Often') to Q34, Q35, Q36, Q37, Q38 on the HCP form.*

Now I will ask you about your communication skills

34. I can put myself in others shoes

Always
Often
Sometimes
Rarely
Never
Rarely
Refused to answer

35. I let others know that I understand what they say

Always
Often
Sometimes
Rarely
Never
Rarely
Refused to answer

36. In conversations with my colleagues, I perceive not only what they say but what they don't say

Always
Often
Sometimes
Rarely
Never
Rarely
Refused to answer

37. I communicate effectively

Always
Often

For peer review only

Sometimes
Rarely
Never
Rarely
Refused to answer

38. I communicate with others as though they are my equals

Always
Often
Sometimes
Rarely
Never
Rarely
Refused to answer

7. Secondary outcome: Improved self-efficacy

Outcome definition: *Cumulative score based on positive responses (Agree or Strongly Agree) to Q26, Q27, Q28, Q29, Q30, Q31, Q32, Q33 on the HCP form.*

Now I would like to ask you a few questions about how you solve problems that you face. Please tell me how much you agree or disagree with the statements that I read to you

1 = Strongly disagree
2 = Disagree
3 = Neither agree nor disagree
4 = Agree
5 = Strongly agree

Q26. I will be able to achieve most of the goals that I have set for myself
Q27. When facing difficult tasks, I am certain that I will accomplish them
Q28. In general, I think that I can obtain outcomes that are important to me
Q29. I believe that I can succeed at almost any endeavor to which I set my mind
Q30. I will be able to successfully overcome many challenges
Q31. I am confident that I can perform effectively on many different tasks
Q32. Compared to other people, I can do most tasks very well
Q33. Even when things are tough, I can perform quite well

8. Secondary outcome: Improved attitudes towards FGM

Outcome definition*: Cumulative score based on positive responses to Q12, Q13, Q14, Q15, Q16, Q17, Q18 & Q19 on the HCP form.*

For each of the following statements please state if you:

1=Agree
2=Disagree
3=Don't know
4=Refused to answer

Q12. A girl who has not undergone FGM is unclean
Q13. A girl who has not undergone FGM cannot be married within her community
Q14. A girl who has not undergone FGM is a disgrace to her family's honor
Q15. Health care providers who provide FGM are violating FGM
Q16. Health care providers who provide FGM should be punished
Q17. FGM is a good practice
Q18. FGM is a violation of women and girls' rights
Q19. FGM is religious mandate

For peer review only

9. Tertiary outcome: ANC provider confidence in FGM knowledge to provide care

Outcome definition: *Positive responses ('Somewhat Confident' or 'Confident') to Q8 & Q9 on the HCP form*

Q8. When you treat or attend to a girl or woman with female genital mutilation, how confident are you that you have enough knowledge to provide good quality care?

1=Not confident
2=Somewhat confident
3=Confident
4=Refused to answer

Q9. How confident are you in your knowledge to communicate on FGM prevention?

1=Not confident
2=Somewhat confident
3=Confident
4=Refused to answer

10. Tertiary outcome: ANC provider support for FGM

Outcome definition: *Positive response ('Do not intend to cut her') to Q20 on the HCP form*

Q20. Pretend you had a daughter now who was at an age when cutting occurs, what would your intention to cut her be?

1=Intend to cut her
2=Do not intend to cut her
3=Don't know
4=Refused to answer

11. Tertiary outcome: ANC provider support for medicalized FGM

Outcome definition: *Correct response ('No') to Q21 on HCP form*

Q21. If a family brought their daughter to the clinic requesting genital cutting, for non-health reasons, would you perform it?

1=Yes
2=No
3=Don't know
4=Refused to answer

12. Tertiary outcome: ANC client change in support for FGM after ANC visit

Outcome definition: *Response to Q13 on EXT form*

Q13. What do you feel about FGM now as compared to before you came to the clinic today?

1= Same, no change
2=I feel more supportive of FGM now as compared to before I came
3=I feel less supportive of FGM now as compared to before I came
4=Don't know
5=Other
6=Refused to answer

13. Tertiary outcome: ANC client support or opposition to FGM

Outcome definition: *Response to Q14 on EXT form*

Q14. How supportive are you of female genital mutilation?

1=Strongly opposed
2=Somewhat opposed

For peer review only

3=Neutral
4=Somewhat supportive
5=Strongly supportive
6=Refused to answer

14. Tertiary outcome: ANC client intention to cut after ANC visit.
Outcome definition: *Response to Q16 on EXT form*

Q.16 Pretend you had a daughter now who was at an age where cutting occurs, what would your intention to cut her be?
1=Intend to cut her
2=Do not intend to cut her
3=Don't know
4=Refused to answer

15. Tertiary outcome: ANC client choice of who to cut their daughters.
Outcome definition: *Response to Q17 on EXT form*

Q17. If intending to cut, who would you prefer to do the cutting?
1=Traditional practitioner
2=Health care provider
3=Other
4=Refused to answer

16. Tertiary outcome: ANC client wish to be active in FGM prevention
Outcome definition: *Response to Q18 on EXT form*

Q.18 Do you wish/want to be active in preventing FGM?
1=Yes
2=No
3=Don't know
4=Refused to answer

For peer review only

A HEALTH SYSTEMS APPROACH TO PREVENTION OF FEMALE GENITAL MUTILATION USING PERSON-CENTRED COMMUNICATION: IMPLEMENTATION RESEARCH PROJECT IN GUINEA, SOMALIA AND KENYA (A65993)

ANC PROVIDER SCREENING QUESTIONNAIRE (SCR)

Participant ID:

A	6	5	9	9	3

Project ID:

Country ID: Facility ID:

Instructions: Observe and report findings from the health facility.

1. MoH policy on FGM posted on the wall?
☐ Yes
☐ No
1a. If yes, is it placed where health care providers can see/read it e.g. bulletin board?
☐ Yes
☐ No

2. Are there FGM prevention posters on the wall of the waiting room? ☐ Yes
☐ No
2a. If yes, is it placed in place where ANC clients can see it
☐ Yes
☐ No

3. Is there WHO FGM Clinical Handbook in the ANC consultation room? ☐ Yes
☐ No
3a. If yes, is it placed where ANC provider can see /use it?
☐ Yes
☐ No

4. Is there FGM ABCD guide in ANC consultation room?
☐ Yes
☐ No
4a. If yes, is it placed where ANC provider can see /use it
☐ Yes ☐
No

Instructions: Assess health facility factors that may facilitate/constrain intervention delivery by reviewing health facility administrative records and notes and by meeting with the health facility manager.

5. Number of ANC providers __________________

6. Average number of ANC clients per month_________________

7. Number of ANC providers trained on PCC on FGM prevention
☐ All (specify number trained): ________
☐ Some (specify number trained): ________
☐ None

8. Indicate the number of MoH supervisory visits to the clinic in the past year_________________

For peer review only

A HEALTH SYSTEMS APPROACH TO PREVENTION OF FEMALE GENITAL MUTILATION USING PERSON-CENTRED COMMUNICATION: IMPLEMENTATION RESEARCH PROJECT IN GUINEA, SOMALIA AND KENYA (A65993)

ANC PROVIDER SCREENING QUESTIONNAIRE (SCR)

Participant ID: | | | | |

A	6	5	9	9	3

Project ID: | | | | |

Country ID: | | | | | |

Facility ID:

9. How frequently are staff meetings held?

☐ Monthly

☐ Every 2 to 4 months

☐ Every 6 to 12months

☐ Never

10. What is the size of the population served by this facility? (*specify number)* ________________

11. Are there country/region-specific FGM laws that are enforced?

☐ Yes

☐ No

12. Are there anti-FGM activities that target the population served by this health facility?

☐ Yes

☐ No

13. Are there pro-FGM activities that target the population served by this health facility?

☐ Yes

☐ No

Additional comments:

____

____

____

____

For peer review only

A HEALTH SYSTEMS APPROACH TO PREVENTION OF FEMALE GENITAL MUTILATION USING PERSON-CENTRED COMMUNICATION: IMPLEMENTATION RESEARCH PROJECT IN GUINEA, SOMALIA AND KENYA (A65993)

ANC PROVIDER SCREENING QUESTIONNAIRE (SCR)

Participant ID: ☐☐☐☐

Project ID: ☐☐☐☐ Country ID: ☐☐☐☐☐ Facility ID:

☐☐☐☐☐☐

A 6 5 9 9 3

1. **What is your age? ______________**
2. **What is your sex?**
   1. ☐ Female
   2. ☐ Male
3. **What is your religion?**
   1. ☐ Muslim
   2. ☐ Christian
   3. ☐ Other
   4. ☐ None
   5. ☐ Refused
4. **What is your occupation/designation?**
   1. ☐ Midwife
   2. ☐ Nurse
   3. ☐ Other, specify ____
5. **What is the highest education level of education you achieved?**
   1. ☐ Certificate
   2. ☐ Diploma
   3. ☐ Bachelors
   4. ☐ Masters or above
   5. ☐ Other, specify ____
6. **For how many years have you been working in your field? ________**
7. **During you clinical training, did you receive any formal training on female genital mutilation?**
   1. ☐ Yes.
   2. ☐ No. Go to section B
   3. ☐ I don't know. Go to section B
8. **When did you receive the training?**
   1. ☐ During my studies (pre-service training)
   2. ☐ After graduation/at work (in-service training)
   3. ☐ Both
   4. ☐ I don't know

   7. ☐ Not applicable

For peer review only

For peer review only - http://bmjopen.bmj.com/site/about/guidelines.xhtml

A HEALTH SYSTEMS APPROACH TO PREVENTION OF FEMALE GENITAL MUTILATION USING PERSON-CENTRED COMMUNICATION: IMPLEMENTATION RESEARCH PROJECT IN GUINEA, SOMALIA AND KENYA (A65993)

ANC PROVIDER SCREENING QUESTIONNAIRE (SCR)

Participant ID:

Project ID: Country ID: Facility ID:

To be completed by data collector:

Data Collector ID:

Signature:

Date:

Day		Month			Year			
					2	0		

For peer review only

For peer review only

A HEALTH SYSTEMS APPROACH TO PREVENTION OF FEMALE GENITAL MUTILATION USING PERSON-CENTRED COMMUNICATION: IMPLEMENTATION RESEARCH PROJECT IN GUINEA, SOMALIA AND KENYA (A65993)

ANC PROVIDER SCREENING QUESTIONNAIRE (SCR)

Participant ID:

Project ID: Country ID: Facility ID:

A	6	5	9	9	3

9. What was the format of the training? (Check all that apply)

1. ☐ Classroom lessons
2. ☐ Workshops
3. ☐ Digital format (E-learning videos; smart phone app)
4. ☐ During clinical practice under supervision of a mentor
5. ☐ Other, specify ____

7. ☐ Not applicable

10. During your pre- or post- graduate training, did you receive any formal training on communication or counselling?

1. ☐ Yes.
2. ☐ No.
3. ☐ I don't know

11. During you pre or post graduate training, did you receive any formal training on person-centred care?

1. ☐ Yes.
2. ☐ No.
3. ☐ I don't know

12. Have you ever cut the genitals of a girl (<=18 years old) for non-health reasons?

1. ☐ Yes.
2. ☐ No.
3. ☐ I don't know

For peer review only - http://bmjopen.bmj.com/site/about/guidelines.xhtml

A HEALTH SYSTEMS APPROACH TO PREVENTION OF FEMALE GENITAL MUTILATION USING PERSON-CENTRED COMMUNICATION: IMPLEMENTATION RESEARCH PROJECT IN GUINEA, SOMALIA AND KENYA (A65993)

ANC PROVIDER QUESTIONNAIRE (HCP)

Participant ID:

Project ID: Country ID: Facility ID:

A	6	5	9	9	3

To be completed by data collector:

Data Collector ID: *Date:*

Signature:

Day		Month			Year			
					2	0		

1. **Have you ever heard about female genital mutilation?**
   ☐ Yes
   ☐ No
2. **Do the women in your community undergo female genital mutilation?**
   ☐ Yes
   ☐ No
   ☐ I don't know
3. **Do you know of the WHO classification for female genital mutilation?**
   ☐ Yes
   ☐ No. Skip to Q5
4. **Please provide the WHO classification for the following FGM images (to include images)**
   a. **IMAGE of Type III FGM to be inserted here**
      i. ☐ **Type I**
      ii. ☐ **Type II**
      iii. ☐ **Type III**
      iv. ☐ **Type IV**
      v. ☐n**Don't know**
   b. **IMAGE of Type I FGM to be inserted here**
      i. ☐ **Type I**
      ii. ☐ **Type II**
      iii. ☐ **Type III**

To be completed by data collector:

Data Collector ID: *Date:*

Signature:

Day		Month			Year			
					2	0		

For peer review only - http://bmjopen.bmj.com/site/about/guidelines.xhtml

A HEALTH SYSTEMS APPROACH TO PREVENTION OF FEMALE GENITAL MUTILATION USING PERSON-CENTRED COMMUNICATION: IMPLEMENTATION RESEARCH PROJECT IN GUINEA, SOMALIA AND KENYA (A65993)

ANC PROVIDER QUESTIONNAIRE (HCP)

Participant ID:

Project ID: Country ID: Facility ID:

iv. ☐ Type IV

v. ☐nDon't know

c. **IMAGE of Type II FGM to be inserted here**

i. ☐ **Type I** ii. ☐ **Type II**

A 6 5 9 9 3

iii. ☐ **Type III**

iv. ☐ **Type IV**

v. ☐n**Don't know**

d. IMAGE of Type III FGM to be inserted here

i. ☐ **Type I**

ii. ☐ **Type II**

iii. ☐ **Type III**

iv. ☐ **Type IV**

v. ☐n**Don't know**

e.

5. Do you know of any health complications arising from female genital mutilation?

☐ Yes

☐ No. Skip to Q6

6. Is female genital mutilation illegal in your country (specify actual study country)?

1. ☐ Yes
2. ☐ No
3. ☐ I don't know

7. Are you aware of any existing WHO tools/guidance on female genital mutilation and its complications?

1. ☐ Yes. *If yes, please specify*……………………………………………………….
2. ☐ No

To be completed by data collector:

Data Collector ID:	*Date:*		
Signature:	Day	Month	Year
			2 0

For peer review only - http://bmjopen.bmj.com/site/about/guidelines.xhtml

A HEALTH SYSTEMS APPROACH TO PREVENTION OF FEMALE GENITAL MUTILATION USING PERSON-CENTRED COMMUNICATION: IMPLEMENTATION RESEARCH PROJECT IN GUINEA, SOMALIA AND KENYA (A65993)

ANC PROVIDER QUESTIONNAIRE (HCP)

Participant ID: | | | | |

Project ID: A 6 5 9 9 3 Country ID: Facility ID:

8. When you treat or attend a girl or a woman with genital mutilation, how confident are you that you have enough knowledge to provide good quality health care? *Rate between 1 – 4*

1. ☐ Not confident at all
2. ☐ Not very confident
3. ☐ Fairly confident
4. ☐ Fully confident

9. How confident are you in your FGM knowledge to communicate on FGM prevention?
Rate between 1 – 4

1. ☐ Not confident at all
2. ☐ Not very confident
3. ☐ Fairly confident
4. ☐ Fully confident

For each of the following statements please state if you agree/disagree or don't know.

10. A girl who has not undergone FGM is unclean.

1. ☐ Agree
2. ☐ Disagree
3. ☐ I don't know

11. A girl without FGM cannot be married within her community.

1. ☐ Agree
2. ☐ Disagree
3. ☐ I don't know

12. A girl who has not undergone FGM is a disgrace to her family's honour.

1. ☐ Agree
2. ☐ Disagree

To be completed by data collector:

Data Collector ID: *Date:*

Signature:

Day		Month			Year			
					2	0		

For peer review only

A HEALTH SYSTEMS APPROACH TO PREVENTION OF FEMALE GENITAL MUTILATION USING PERSON-CENTRED COMMUNICATION: IMPLEMENTATION RESEARCH PROJECT IN GUINEA, SOMALIA AND KENYA (A65993)

ANC PROVIDER QUESTIONNAIRE (HCP)

Participant ID:

Project ID: Country ID: Facility ID:

3. ☐ I don't know

13. Health care providers who perform FGM are violating medical ethics.

1. ☐ Agree
2. ☐ Disagree
3. ☐ I don't know

14. Health care providers who perform FGM should be punished.

1. ☐ Agree
2. ☐ Disagree

A 6 5 9 9 3

3. ☐ I don't know

15. FGM is a good practice

1. ☐ Agree
2. ☐ Disagree
3. ☐ I don't know

16. FGM is a violation of women's and girls' rights

1. ☐ Agree
2. ☐ Disagree
3. ☐ I don't know

17. FGM is a religious mandate

1. ☐ Agree
2. ☐ Disagree
3. ☐ I don't know

18. Pretend you had a daughter now who was at an age when cutting occurs, what would your intention to cut her be? 1. ☐ Intend to cut her

2. ☐ Do not intend to cut her
3. ☐ Undecided
4. Refused to answer

19. If a family brought their daughter to the clinic requesting genital cutting for non-health reasons, would you perform it?

To be completed by data collector:

Data Collector ID:	*Date:*		
Signature:	Day	Month	Year
			2 0

For peer review only - http://bmjopen.bmj.com/site/about/guidelines.xhtml

A HEALTH SYSTEMS APPROACH TO PREVENTION OF FEMALE GENITAL MUTILATION USING PERSON-CENTRED COMMUNICATION: IMPLEMENTATION RESEARCH PROJECT IN GUINEA, SOMALIA AND KENYA (A65993)

ANC PROVIDER QUESTIONNAIRE (HCP)

Participant ID:

Project ID: Country ID: Facility ID:

1. ☐ Yes
2. ☐ No
3. ☐ I don't know

Now I would like to ask you a few questions about how you solve problems that you face. Please state how much you agree or disagree with the statements that I read, , where 1=Strongly disagree; 2=Disagree; 3=Neither agree nor disagree; 4=Agree; 5=Strongly agree

20. I will be able to achieve most of the goals that I have set for myself.

1. ☐ **Strongly disagree**
2. ☐ **Disagree**
3. ☐ **Neither agree nor disagree**
4. ☐ **Agree**

A 6 5 9 9 3

5. ☐ **Strongly agree**
6. ☐ **Don't know**

21. When facing difficult tasks, I am certain that I will accomplish them.

1. ☐ **Strongly disagree**
2. ☐ **Disagree**
3. ☐ **Neither agree nor disagree**
4. ☐ **Agree**
5. ☐ **Strongly agree**
6. ☐ **Don't know**

22. In general, I think that I can obtain outcomes that are important to me.

1. ☐ **Strongly disagree**
2. ☐ **Disagree**
3. ☐ **Neither agree nor disagree**

To be completed by data collector:

Data Collector ID:	*Date:*								
Signature:	Day		Month			Year			
						2	0		

For peer review only - http://bmjopen.bmj.com/site/about/guidelines.xhtml

A HEALTH SYSTEMS APPROACH TO PREVENTION OF FEMALE GENITAL MUTILATION USING PERSON-CENTRED COMMUNICATION: IMPLEMENTATION RESEARCH PROJECT IN GUINEA, SOMALIA AND KENYA (A65993)

ANC PROVIDER QUESTIONNAIRE (HCP)

Participant ID:

Project ID: Country ID: Facility ID:

4. ☐ **Agree**
5. ☐ **Strongly agree**
6. ☐ **Don't know**

23. I believe I can succeed at most any endeavour to which I set my mind.

1. ☐ **Strongly disagree**
2. ☐ **Disagree**
3. ☐ **Neither agree nor disagree**
4. ☐ **Agree**
5. ☐ **Strongly agree**
6. ☐ **Don't know**

24. I will be able to successfully overcome many challenges.

1. ☐ **Strongly disagree**
2. ☐ **Disagree**
3. ☐ **Neither agree nor disagree**
4. ☐ **Agree**
5. ☐ **Strongly agree**

A 6 5 9 9 3

6. ☐ **Don't know**

25. I am confident that I can perform effectively on many different tasks.

1. ☐ **Strongly disagree**
2. ☐ **Disagree**
3. ☐ **Neither agree nor disagree**
4. ☐ **Agree**
5. ☐ **Strongly agree**
6. ☐ **Don't know**

To be completed by data collector:

Data Collector ID:	*Date:*		
Signature:	Day	Month	Year
			2 0

For peer review only

A HEALTH SYSTEMS APPROACH TO PREVENTION OF FEMALE GENITAL MUTILATION USING PERSON-CENTRED COMMUNICATION: IMPLEMENTATION RESEARCH PROJECT IN GUINEA, SOMALIA AND KENYA (A65993)

ANC PROVIDER QUESTIONNAIRE (HCP)

Participant ID: ☐☐☐☐

Project ID: ☐☐☐☐☐☐ Country ID: ☐☐☐☐ Facility ID: ☐☐☐☐☐

26. Compared to other people, I can do most tasks very well.

1. ☐ **Strongly disagree**
2. ☐ **Disagree**
3. ☐ **Neither agree nor disagree**
4. ☐ **Agree**
5. ☐ **Strongly agree**
6. ☐ **Don't know**

For peer review only

27. Even when things are tough, I can perform quite well.

1. ☐ **Strongly disagree**
2. ☐ **Disagree**
3. ☐ **Neither agree nor disagree**
4. ☐ **Agree**
5. ☐ **Strongly agree**
6. ☐ **Don't know**

A 6 5 9 9 3

28. Would you like to receive more training related to care for women and girls with FGM?

1. ☐ Yes
2. ☐ No
3. ☐ I don't know

29. If a pregnant woman is expected to have a girl, do you discourage her from having her daughter cut?

To be completed by data collector:

Data Collector ID: *Date:*

Signature:

Day		Month			Year			
					2	0		

For peer review only - http://bmjopen.bmj.com/site/about/guidelines.xhtml

A HEALTH SYSTEMS APPROACH TO PREVENTION OF FEMALE GENITAL MUTILATION USING PERSON-CENTRED COMMUNICATION: IMPLEMENTATION RESEARCH PROJECT IN GUINEA, SOMALIA AND KENYA (A65993)

ANC PROVIDER QUESTIONNAIRE (HCP)

Participant ID:

Project ID: Country ID: Facility ID:

1. ☐ Always
2. ☐ Often
3. ☐ Sometimes
4. ☐ Rarely
5. ☐ Never

30. If you heard of or saw a colleague performing female genital mutilation, what would you do? ***(Tick all that apply)***

1. ☐ I would report him/her to the authorities
2. ☐ I would discuss with him/her and explain to him/her that health care providers should not perform female genital mutilation
3. ☐ I would not get involved 4. ☐ I don't know

31. How often do you look for female genital cutting/excision when performing a gynecological examination of the vulva?

1. ☐ Always
2. ☐ Often
3. ☐ Sometimes
4. ☐ Rarely
5. ☐ Never

32. How often do you record the female genital mutilation in the women's medical file if you are aware that she has undergone FGM?

1. ☐ Always
2. ☐ Often
3. ☐ Sometimes
4. ☐ Rarely
5. ☐ Never

33. Would you like to receive more training on how to help patients to prevent FGM?

1. ☐ Yes
2. ☐ No
3. ☐ I don't know

A 6 5 9 9 3

34. I can put myself in others' shoes

1. ☐ Always
2. ☐ Often

To be completed by data collector:

Data Collector ID: *Date:*

Signature:

Day		Month			Year			
					2	0		

For peer review only - http://bmjopen.bmj.com/site/about/guidelines.xhtml

A HEALTH SYSTEMS APPROACH TO PREVENTION OF FEMALE GENITAL MUTILATION USING PERSON-CENTRED COMMUNICATION: IMPLEMENTATION RESEARCH PROJECT IN GUINEA, SOMALIA AND KENYA (A65993)

ANC PROVIDER QUESTIONNAIRE (HCP)

Participant ID: [ ][ ][ ][ ]

Project ID: [ ][ ][ ][ ][ ][ ]   Country ID: [ ][ ][ ][ ]   Facility ID: [ ][ ][ ][ ][ ]

3. ☐ Sometimes
4. ☐ Rarely
5. ☐ Never

35. I let others know I understand what they say

1. ☐ Always
2. ☐ Often
3. ☐ Sometimes
4. ☐ Rarely
5. ☐ Never

36. In conversations with my colleagues, I perceive not only what they say but what they don't say

1. ☐ Always
2. ☐ Often
3. ☐ Sometimes
4. ☐ Rarely
5. ☐ Never

37. I communicate effectively

1. ☐ Always
2. ☐ Often
3. ☐ Sometimes
4. ☐ Rarely
5. ☐ Never

38. I communicate with others as though they are my equals

1. ☐ Always
2. ☐ Often
3. ☐ Sometimes
4. ☐ Rarely
5. ☐ Never

A 6 5 9 9 3

To be completed by data collector:

Data Collector ID: *Date:*

Signature:

Day		Month			Year			
					2	0		

For peer review only - http://bmjopen.bmj.com/site/about/guidelines.xhtml

For peer review only

A HEALTH SYSTEMS APPROACH TO PREVENTION OF FEMALE GENITAL MUTILATION USING PERSON-CENTRED COMMUNICATION: IMPLEMENTATION RESEARCH PROJECT IN GUINEA, SOMALIA AND KENYA (A65993)

ANC PROVIDER QUESTIONNAIRE (HCP)

Participant ID:

Project ID: Country ID: Facility ID:

These next questions relate to your clinic setting:

39. Have you seen the posters on FGM at the clinic?

1. ☐ Yes
2. ☐ No
3. ☐ I don't know

40. Have you referred to the clinical handbook on FGM that is available in your clinic?

1. ☐ No
2. ☐ I don't know

41. Do you think it is feasible to provide FGM prevention counselling during ANC visits?

1. ☐ Yes
2. ☐ No
3. ☐ I don't know

Comments

-

To be completed by data collector:

Data Collector ID: *Date:*

Signature:

Day		Month			Year			
					2	0		

For peer review only - http://bmjopen.bmj.com/site/about/guidelines.xhtml
For peer review only

A HEALTH SYSTEMS APPROACH TO PREVENTION OF FEMALE GENITAL MUTILATION USING PERSON-CENTRED COMMUNICATION: IMPLEMENTATION RESEARCH PROJECT IN GUINEA, SOMALIA AND KENYA (A65993)

Participant ID:

Project ID: Facility ID:

FIRST ANC CLIENT EXIT QUESTIONNAIRE (EXT)

Country ID:

A 6 5 9 9 3

1. **How old are you? (years)**____
2. **What is your religion?**
   1. ☐ Muslim
   2. ☐ Christian
   3. ☐ Other
   4. ☐ None
   5. ☐ Refused
3. **What is the highest level of education you achieved?**
   1. ☐ None
   2. ☐ Primary
   3. ☐ Secondary
   4. ☐ University
   5. ☐ Other, *specify* ____
4. **Many women in your community have had their genitals cut when they were children, if you are comfortable telling me, can I ask if you have undergone this practice?**
   1. ☐ Yes
   2. ☐ No
   3. ☐ I don't know
   4. ☐ Refused
5. **How supportive are you of female genital mutilation?**
   1. ☐ Strongly opposed
   2. ☐ Somewhat opposed
   3. ☐ Neutral (Neither opposed or supportive)
   4. ☐ Somewhat supportive
   5. ☐ Strongly supportive

The following questions relate to your visit today. During your visit today:

6. **Did you see any FGM poster(s) in the waiting room?**
   1. ☐ Yes
   2. ☐ No
   3. ☐ I don't know

For peer review only

A HEALTH SYSTEMS APPROACH TO PREVENTION OF FEMALE GENITAL MUTILATION USING PERSON-CENTRED COMMUNICATION: IMPLEMENTATION RESEARCH PROJECT IN GUINEA, SOMALIA AND KENYA (A65993)

Participant ID:

Project ID: Facility ID:

7. Did the ANC provider ask if you have undergone FGM?

1. ☐ Yes
2. ☐ No
3. ☐ I don’t know

8. Did the ANC provider explain how FGM can harm your health?

1. ☐ Yes
2. ☐ No

Version 2 – 6th November 2019 1 **FIRST ANC CLIENT EXIT QUESTIONNAIRE (EXT)**

Country ID:

A 6 5 9 9 3

3. ☐ I don’t know

9. Did the ANC provider ask about your personal belief regarding FGM?

1. ☐ Yes
2. ☐ No
3. ☐ I don’t know

10. Did the ANC provider discuss why FGM should be prevented?

1. ☐ Yes
2. ☐ No
3. ☐ I don’t know

11. Did the ANC provider discuss how FGM could be prevented?

1. ☐ Yes
2. ☐ No
3. ☐ I don’t know

12. Did you have questions about FGM to ask the ANC provider?

1. ☐ Yes
2. ☐ No
3. ☐ I don’t know

13. Did you feel encouraged to ask questions about FGM?

1. ☐ Yes
2. ☐ No
3. ☐ I don’t know

14. Are you satisfied with how FGM was addressed during your visit with your ANC provider today?

For peer review only

For peer review only - http://bmjopen.bmj.com/site/about/guidelines.xhtml

A HEALTH SYSTEMS APPROACH TO PREVENTION OF FEMALE GENITAL MUTILATION USING PERSON-CENTRED COMMUNICATION: IMPLEMENTATION RESEARCH PROJECT IN GUINEA, SOMALIA AND KENYA (A65993)

Participant ID: ☐☐☐☐

Project ID: ☐☐☐☐☐☐ ☐☐☐☐

Facility ID: ☐☐☐☐☐

1. ☐ Yes
2. ☐ No
3. ☐ I don't know

15. What do you feel about FGM now as compared to before you came to the clinic today?

1. ☐ Same, no change
2. ☐ I feel more supportive of FGM now as compared to before I came
3. ☐ I feel less supportive of FGM now as compared to before I came
4. ☐ I do not know
5. ☐ Other, *specify* ______________________________________

16. Pretend you had a daughter now who was at an age when cutting occurs, what would your intention to cut her be?

1. ☐ Intend to cut her
2. ☐ Do not intend to cut her

17. Do you wish/want to be active in preventing FGM?

1. ☐ Yes
2. ☐ No
3. ☐ I don't know

For peer review only

Supplementary file 3: Additional analyses (appendices 1 – 3)

Appendix 1: Comparison baseline characteristics of ANC facilities

Characteristics	Facilities included in final analysis (n=163)	Facilities excluded* from final analysis (n=17)
Number of ANC providers	Mean 4 (SD: 3) Median 3 (1-14, IQR 3)	Mean 3 (SD: 3) Median 2 (1-9, IQR 1)
Average number of ANC clients/month	Mean 150 (SD: 127) Median 118 (3-664, IQR 141)	Mean 162 (SD: 147) Median 100 (25-600, IQR 200)
MoH supervisory visits in the past year	Mean 4 (SD: 3) Median 3 (0-18, IQR 2)	Mean 5 (SD: 4) Median 4 (0-12, IQR 4)
Size of catchment population served	Mean 36,754 (SD: 126,082) Median 15,972 (1,000-1,458,000, IQR 24,332)	Mean 11,737 (SD: 14,62) Median 7,800 (1,200-63,000, IQR 7,505
Presence of anti-FGM activities in the catchment area		
Yes	74 (45%)	9 (53%)
No	89 (55%)	8 (47%)
Presence of pro-FGM activities in the catchment area		
Yes	21 (13%)	2 (12%)
No	140 (86%)	15 (88%)
Don't Know	2 (1%)	-

*Total of ANC clinics not included: 16 clinics were excluded (7 intervention and 9 control) due to loss-to-follow up (LTFU) of ANC provider i.e., the clinics did not have at least one ANC provider present across all study time points while one ANC clinic in Kenya was never visited at subsequent time points due to issues with insecurity. An ANC provider from one of the clinics in Kenya that had been inaccessible due to insecurity attended the PCC training and was subsequently interviewed.

For peer review only

Appendix 2: Comparison of baseline characteristics of ANC providers

Characteristics	Providers recruited at Baseline (n=436)	Providers enrolled with complete data at Month 6 (n=232)	Providers not enrolled with no data at Month 6 (n=204)
Age	37 (20-65; SD: 10)	36 (20-65; SD: 10)	38 (21-62; SD: 10)
Years of professional experience	9 (1-39; SD: 7)	8 (1-39; SD: 7)	10 (1-36; SD: 8)
Sex			
Female	361 (83%)	193 (83%)	168 (82%)
Male	75 (17%)	39 (17%)	36 (18%)
Highest educational level			
Certificate	44 (3%)	21 (5%)	23 (11%)
Diploma	309 (71%)	158 (68%)	151 (74%)
Bachelors	64 (15%)	44 (19%)	20 (10%)
Masters & above	3 (0.7%)	1 (0.4%)	2 (1%)
Other[#]	16 (4%)	8 (3%)	8 (4%)
Current professional role/title			
Midwife	198 (45%)	103 (44%)	95 (47%)
Nurse	95 (22%)	51 (22%)	44 (22%)
Nurse-Midwife	94 (22%)	54 (23%)	40 (20%)
Other	49 (11%)	24 (10%)	25 (12%)
Received formal training on FGM during clinical training			
Yes	158 (36%)	85 (37%)	73 (36%)
No	275 (63%)	146 (63%)	129 (63%)
Don't Know	3 (0.7%)	1 (0.4%)	2 (1%)
Timing of clinical training on FGM			
Pre-service	63 (14%)	33 (14%)	30 (15%)
In-service	81 (19%)	45 (19%)	36 (18%)
Both pre- and in-service	14 (3%)	7 (3%)	7 (3%)
Received formal training on communication/counselling			
Yes	287 (66%)	149 (64%)	138 (68%)
No	149 (34%)	83 (36%)	66 (32%)
Received formal training on person-centered care			
Yes	227 (52%)	118 (51%)	109 (53%)
No	207 (47%)	131 (56%)	94 (46%)
Don't know	2 (0.5%)	1 (0.4%)	1 (0.5%)

For peer review only

Characteristics	Providers recruited at Baseline (n=436)	Providers enrolled with complete data at Month 6 (n=232)	Providers not enrolled with no data at Month 6 (n=204)
Undergone FGM			
Yes	226 (52%)	126 (54%)	100 (49%)
No	128 (29%)	63 (27%)	65 (32%)
Don't know	4 (0.9%)	2 (1%)	1 (0.5%)
Refused to answer	3 (0.7%)	2 (1%)	1 (0.55)
Conducted FGM			
Yes	35 (8%)	15 (7%)	20 (10%)
Conducted FGM on a girl <18 years			
Yes	32 (7%)	14 (6%)	18 (9%)

For peer review only

Appendix 3: Comparison of study outcomes between baseline vs. month 3 and month 3 vs. month 6 in the intervention arm

	Baseline (Intervention only)	Month 3 (Intervention only)	P-value	Month 3 (Intervention only)	Month 6 (Intervention only)	P-value
**Primary Outcomes**						
**ANC clients reporting that their provider implemented components of PCC for FGM prevention**						
Provider asked client if they have undergone FGM	48 (6%)	298 (37%)	<0.0001	298 (37%)	694 (78%)	<0.0001
Provider asked client about their (client's) personal beliefs regarding FGM	38 (5%)	239 (29%)	<0.0001	239 (29%)	616 (76%)	<0.0001
Provider discussed with client why FGM should be prevented	56 (7%)	243 (30%)	<0.0001	243 (30%)	629 (77%)	<0.0001
Provider discussed with client how FGM could be prevented	48 (6%)	224 (28%)	<0.0001	224 (28%)	592 (73%)	<0.0001
Client satisfied with how FGM was addressed by provider during clinic visit	176 (21%)	346 (43%)	<0.0001	346 (43%)	684 (84%)	<0.0001
Mean score of PCC approach (out of 5)	0.5 (0.4-0.5)	1.7 (1.5-1.8)	<0.0001	1.7 (1.5-1.8)	3.9 (3.8-4.0)	<0.0001
Mean score of PCC + appropriate FGM prevention & care (out of 8)	1.8 (1.6-2.1)	3.3 (2.8-3.8)	<0.0001	3.3 (2.8-3.8)	6.2 (5.9 – 6.6)	<0.0001
**ANC clinic preparedness to offer FGM prevention and care services**						
Clinics with ALL correct answers for facility preparedness	0 (0%)	42 (52%)	<0.0001	42 (52%)	56 (69%)	<0.01
Mean score of clinic preparedness (out of 4)	0.1 (0.01-0.2)	3.1 (2.9-3.4)	<0.0001	3.1 (2.9-3.4)	3.4 (3.2-3.6)	0.18
Providers using level 1 intervention package	1 (1%)	61 (58%)	<0.0001	61 (58%)	96 (91%)	<0.0001
Providers offering appropriate FGM-related prevention and care services	11 (11%)	20 (19%)	<0.0001	20 (19%)	52 (50%)	<0.0001
**Secondary Outcomes**						
Providers with correct FGM-related knowledge responses	0 (0%)	1 (3%)	0.47	1 (3%)	8 (8%)	0.06
Providers with appropriate interpersonal communication skills	49 (49%)	62 (59%)	0.08	62 (59%)	74 (70%)	0.11
Providers with high self-efficacy	85 (85%)	94 (90%)	0.18	94 (90%)	86 (82%)	0.17
Providers reporting less supportive attitudes towards FGM	67 (67%)	75 (71%)	0.26	75 (71%)	76 (72%)	0.50
Providers with high confidence scores	84 (83%)	81 (77%)	0.30	81 (77%)	103 (98%)	<0.001
Providers not supportive of FGM	91 (91%)	101 (96%)	0.16	101 (96%)	100 (96%)	1.0
Providers not supportive of medicalized FGM	98 (97%)	104 (99%)	0.36	104 (99%)	104 (99%)	0.75
**Other ANC Client Outcomes**						
Clients reporting less support for FGM after ANC clinic visit	194 (24%)	235 (29%)	0.01	235 (29%)	424 (52%)	<0.0001
Clients reporting that they were strongly opposed to FGM	367 (45%)	345 (43%)	0.38	345 (43%)	498 (61%)	<0.0001
Clients reporting that they intend to have their daughters cut	249 (30%)	184 (23%)	<0.0001	184 (23%)	96 (12%)	<0.0001
Clients reporting that they would prefer health care provider to cut daughters	141 (17%)	117 (14%)	0.003	117 (14%)	53 (7%)	<0.001
Clients wishing to be active in FGM prevention	530 (65%)	547 (68%)	0.22	547 (68%)	677 (83%)	<0.001

For peer review only

CONSORT 2010 checklist of information to include when reporting a cluster randomised trial

Section/Topic	Item No	Standard Checklist item	Extension for cluster designs	Page No *
**Title and abstract**				
	1a	Identification as a randomised trial in the title	Identification as a cluster randomised trial in the title	1
	1b	Structured summary of trial design, methods, results, and conclusions (for specific guidance see CONSORT for abstracts)[1,2]	See table 2	3
**Introduction**				
**Background and objectives**	2a	Scientific background and explanation of rationale	Rationale for using a cluster design	5-6
	2b	Specific objectives or hypotheses	Whether objectives pertain to the cluster level, the individual participant level or both	7
**Methods**				
**Trial design**	3a	Description of trial design (such as parallel, factorial) including allocation ratio	Definition of cluster and description of how the design features apply to the clusters	7
	3b	Important changes to methods after trial commencement (such as eligibility criteria), with reasons		N/A
**Participants**	4a	Eligibility criteria for participants	Eligibility criteria for clusters	7-8
	4b	Settings and locations where the data were collected		6-7
**Interventions**	5	The interventions for each group with sufficient details to allow replication, including how and when they were actually administered	Whether interventions pertain to the cluster level, the individual participant level or both	6
**Outcomes**	6a	Completely defined pre-specified primary and	Whether outcome measures pertain to the cluster level, the	10

For peer review only - http://bmjopen.bmj.com/site/about/guidelines.xhtml

		secondary outcome measures, including how and when they were assessed	individual participant level or both	
	6b	Any changes to trial outcomes after the trial commenced, with reasons		N/A
**Sample size**	7a	How sample size was determined	Method of calculation, number of clusters(s) (and whether equal or unequal cluster sizes are assumed), cluster size, a coefficient of intra-cluster correlation (ICC or *k*), and an indication of its uncertainty	10-11
	7b	When applicable, explanation of any interim analyses and stopping guidelines		12
**Randomisation:**				
**Sequence generation**	8a	Method used to generate the random allocation sequence		8-9
	8b	Type of randomisation; details of any restriction (such as blocking and block size)	Details of stratification or matching if used	8-9
**Allocation concealment mechanism**	9	Mechanism used to implement the random allocation sequence (such as sequentially numbered containers), describing any steps taken to conceal the sequence until interventions were assigned	Specification that allocation was based on clusters rather than individuals and whether allocation concealment (if any) was at the cluster level, the individual participant level or both	9
**Implementation**	10	Who generated the random allocation sequence, who enrolled participants, and who assigned participants to interventions	Replace by 10a, 10b and 10c	
	10a		Who generated the random allocation sequence, who enrolled clusters, and who assigned clusters to interventions	8

	10b		Mechanism by which individual participants were included in clusters for the purposes of the trial (such as complete enumeration, random sampling)	8
	10c		From whom consent was sought (representatives of the cluster, or individual cluster members, or both), and whether consent was sought before or after randomisation	8
**Blinding**	11a	If done, who was blinded after assignment to interventions (for example, participants, care providers, those assessing outcomes) and how		8-9
	11b	If relevant, description of the similarity of interventions		8-9
**Statistical methods**	12a	Statistical methods used to compare groups for primary and secondary outcomes	How clustering was taken into account	10-13
	12b	Methods for additional analyses, such as subgroup analyses and adjusted analyses		12-13
**Results**				
**Participant flow (a diagram is strongly recommended)**	13a	For each group, the numbers of participants who were randomly assigned, received intended treatment, and were analysed for the primary outcome	For each group, the numbers of clusters that were randomly assigned, received intended treatment, and were analysed for the primary outcome	15
	13b	For each group, losses and exclusions after randomisation, together with reasons	For each group, losses and exclusions for both clusters and individual cluster members	Figure 2

**Recruitment**	14a	Dates defining the periods of recruitment and follow-up		15
	14b	Why the trial ended or was stopped		N/A
**Baseline data**	15	A table showing baseline demographic and clinical characteristics for each group	Baseline characteristics for the individual and cluster levels as applicable for each group	15-16
**Numbers analysed**	16	For each group, number of participants (denominator) included in each analysis and whether the analysis was by original assigned groups	For each group, number of clusters included in each analysis	15-16
**Outcomes and estimation**	17a	For each primary and secondary outcome, results for each group, and the estimated effect size and its precision (such as 95% confidence interval)	Results at the individual or cluster level as applicable and a coefficient of intra-cluster correlation (ICC or k) for each primary outcome	17-19
	17b	For binary outcomes, presentation of both absolute and relative effect sizes is recommended		19
**Ancillary analyses**	18	Results of any other analyses performed, including subgroup analyses and adjusted analyses, distinguishing pre-specified from exploratory		19
**Harms**	19	All important harms or unintended effects in each group (for specific guidance see CONSORT for harms[3])		N/A
**Discussion**				
**Limitations**	20	Trial limitations, addressing sources of potential bias, imprecision, and, if relevant, multiplicity of analyses		22-23
**Generalisability**	21	Generalisability (external validity, applicability) of the trial findings	Generalisability to clusters and/or individual participants (as relevant)	23

For peer review only

**Interpretation**	22	Interpretation consistent with results, balancing benefits and harms, and considering other relevant evidence	23-24
**Other information**			
**Registration**	23	Registration number and name of trial registry	15
**Protocol**	24	Where the full trial protocol can be accessed, if available	15
**Funding**	25	Sources of funding and other support (such as supply of drugs), role of funders	In Funding Statement

** Note: page numbers optional depending on journal requirements*

For peer review only

BMJ Open

A cluster randomized trial of a health system strengthening approach applying person-centered communication for the prevention of female genital mutilation in Guinea, Kenya, and Somalia

Journal:	*BMJ Open*
Manuscript ID	bmjopen-2023-078771.R3
Article Type:	Original research
Date Submitted by the Author:	20-May-2024
Complete List of Authors:	Balde, Mamadou ; Cellulle de Recherche en Sante de la Reproduction en Guinee (CERREGUI)<br>Ndavi, Patrick; University of Nairobi College of Health Sciences, Department of Obstetrics & Gynecology<br>Oyaro, Vernon; World Health Organization, Department of Sexual and Reproductive Health and Research<br>Soumah, Anne-Marie; Cellulle de Recherche en Sante de la Reproduction en Guinee (CERREGUI)<br>Esho, Tammary; Amref International University<br>King'oo, James; Technical University of Kenya<br>Kemboi , Jackline; Amref Health Africa<br>Sall, Alpha; Cellulle de Recherche en Sante de la Reproduction en Guinee (CERREGUI)<br>Diallo, Aissatou; Cellulle de Recherche en Sante de la Reproduction en Guinee (CERREGUI)<br>Ahmed, Wisal; World Health Organization, Department of Sexual and Reproductive Health and Research<br>Stein, Karin; World Health Organization<br>Nosirov, Khurshed; World Health Organization, Department of Sexual and Reproductive Health and Research<br>Thwin, Soe Soe; World Health Organization, Department of Sexual and Reproductive Health and Research, including UNDP/UNFPA/UNICEF/WHO/World Bank Special Programme of Research, Development and Research Training in Human Reproduction (HRP)<br>Petzold, Max; University of Gothenburg Sahlgrenska Academy, Public Health and Community Medicine<br>Ahmed, Muna; Ministry of Planning and National Development, Central Statistics Department; MUFEIS Multidisciplinary Consultancy Firm , CEO<br>Diriye, Ahmed; Data and Research Solutions<br>Pallitto, C ; World Health Organization, Department of Sexual and Reproductive Health and Research
**Primary Subject Heading**:	Health services research
Secondary Subject Heading:	Evidence based practice, Reproductive medicine, Research methods, Communication, Complementary medicine

Keywords: EDUCATION & TRAINING (see Medical Education & Training), Patient-Centered Care, Primary Care < Primary Health Care, Primary Prevention, PUBLIC HEALTH, Behavior

I, the Submitting Author has the right to grant and does grant on behalf of all authors of the Work (as defined in the below author licence), an exclusive licence and/or a non-exclusive licence for contributions from authors who are: i) UK Crown employees; ii) where BMJ has agreed a CC-BY licence shall apply, and/or iii) in accordance with the terms applicable for US Federal Government officers or employees acting as part of their official duties; on a worldwide, perpetual, irrevocable, royalty-free basis to BMJ Publishing Group Ltd ("BMJ") its licensees and where the relevant Journal is co-owned by BMJ to the co-owners of the Journal, to publish the Work in this journal and any other BMJ products and to exploit all rights, as set out in our licence.

The Submitting Author accepts and understands that any supply made under these terms is made by BMJ to the Submitting Author unless you are acting as an employee on behalf of your employer or a postgraduate student of an affiliated institution which is paying any applicable article publishing charge ("APC") for Open Access articles. Where the Submitting Author wishes to make the Work available on an Open Access basis (and intends to pay the relevant APC), the terms of reuse of such Open Access shall be governed by a Creative Commons licence – details of these licences and which Creative Commons licence will apply to this Work are set out in our licence referred to above.

Other than as permitted in any relevant BMJ Author's Self Archiving Policies, I confirm this Work has not been accepted for publication elsewhere, is not being considered for publication elsewhere and does not duplicate material already published. I confirm all authors consent to publication of this Work and authorise the granting of this licence.

For peer review only

A cluster randomized trial of a health system strengthening approach applying person-centered communication for the prevention of female genital mutilation in Guinea, Kenya, and Somalia

Authors: *Prof. Mamadou Balde, MD[1*], Prof. Patrick Ndavi, MMed[2*], Dr. Vernon Mochache, PhD[3] Dr. Anne-Marie Soumah, MSc[1], Prof. Tammary Esho, PhD[4], James Munyao King'oo, MSc[5], Jackline Kemboi, MSc[2], Alpha Oumar Sall, MSc[1], Aissatou Diallo, MSc[1], Dr. Wisal Ahmed, PhD[3], Dr. Karin Stein, MD[3], Khurshed Nosirov, MCS[3], Dr. Soe Soe Thwin, PhD[3], Prof. Max Petzold, PhD[6], Muna Abdi Ahmed, MSc[7], Ahmed Diriye, MA[8], Dr. Christina Pallitto, PhD[3]*

Institutional Affiliations: [1]Centre for Research in Reproductive Health in Guinea, Conakry, Guinea; [2]Department of Obstetrics and Gynecology, University of Nairobi, Nairobi, Kenya; [3]Department of Sexual and Reproductive Health and Research, and the UNDP/UNFPA/UNICEF/WHO/World Bank Special Programme of Research, Development and Research Training in Human Reproduction (HRP), World Health Organization, Geneva, Switzerland; [4]Amref International University, Nairobi, Kenya; [5]Technical University of Kenya, Nairobi, Kenya; [6]School of Public Health and Community Medicine, Institute of Medicine, University of Gothenburg, Gothenburg, Sweden; [7] Somaliland Central Statistics Department, Hargeisa, Somalia; [8]Data and Research Solutions, Hargeisa, Somalia

** Joint first authors*

Correspondence to:

Dr. Christina Pallitto,

World Health Organization,

Department of Sexual and Reproductive Health and Research

20 Avenue Appia, 1211, Geneva, Switzerland

Telephone: +41 22 791 4745

Email: pallittoc@who.int

For peer review only

Abstract word count: 299

Text word count: 6,084

Tables: 4

Figures: 1

For peer review only

For peer review only - http://bmjopen.bmj.com/site/about/guidelines.xhtml

ABSTRACT

Introduction: There is limited evidence on effective health systems interventions for preventing female genital mutilation (FGM). This study tested a two-level intervention package at primary care applying person-centred communication (PCC) for FGM prevention.

Methods: A cluster randomized trial was conducted in 2020 - 2021 in 180 antenatal care (ANC) clinics in Guinea, Kenya, and Somalia. At baseline, all clinics received guidance and materials on FGM prevention and care; at month three, ANC providers at intervention sites received PCC training. Data were collected from clinic managers, ANC providers and clients at baseline, month three and month six on primary outcomes, including delivery of PCC counseling, utilization of level one materials, health facility preparedness for FGM prevention and care services, and secondary outcomes related to clients' and providers' knowledge and attitudes. Data were analyzed using multi-level and single-level logistic regression models.

Results: Providers in the intervention arm were more likely to deliver PCC for FGM prevention compared to those in the control arm, including inquiring about clients' FGM status (OR: 8.9, 95% CI: 6.9-11.5; p<0.001) and FGM-related beliefs (OR: 9.7, 95% CI: 7.5-12.5; p<0.001) and discussing why (OR: 9.2, 95% CI: 7.1-11.9; p<0.001) or how (OR: 7.7, 95% CI: 6.0-9.9; p<0.001) FGM should be prevented. They were more confident in their FGM-related knowledge (OR: 6.3, 95% CI: 1.4-28.9; p=0.02) and communication skills (OR: 1.7; 95% CI: 1.0-3.0; p=0.06). Intervention clients were less supportive of FGM (AOR: 5.4, 95% CI: 2.4-12.4; p<0.001] and had lower intentions of having their daughters undergo FGM (AOR: 0.3, 95% CI: 0.1-0.7; p=0.004) or seeking medicalized FGM (AOR: 0.2, 95% CI: 0.1-0.5; p<0.001) compared to those in the control arm.

Conclusion: This is the first study to provide evidence of an effective FGM prevention intervention that can be delivered in primary care settings in high prevalence countries.

For peer review only

Trial registration and date: PACTR201906696419769 (June 3rd, 2019)

SUMMARY BOX

- This hybrid-effectiveness implementation research study conducted in primary care public health facilities in three countries with high prevalence of female genital mutilation (FGM) assessed the role of health workers in providing FGM prevention communication in the context of routine antenatal care (ANC).
- It will be important to assess the effectiveness of the person-centred communication approach in other service delivery points, e.g., child immunization, and with other cadres of health workers, e.g., community health workers, to assess its effectiveness beyond ANC care.
- Many factors influence FGM-related decision-making, and while primary care health workers were found to be effective communicators, and the randomized design controlled for some external factors, the impact of a health sector intervention in conjunction with multi-sectoral initiatives requires futher investigation.
- To ensure participation of at least one ANC provider at each site through each time point, eligibility of health workers was based on clinic rotation schedules, which may have introduced a selection bias although the included and excluded providers did not appear to differ significantly.

For peer review only - http://bmjopen.bmj.com/site/about/guidelines.xhtml

INTRODUCTION

Multi-sectoral efforts are needed to achieve Sustainable Development Goal (SDG) 5.3 to eliminate the harmful practice of female genital mutilation (FGM) by 2030 in line with the United Nation's (UN) General Assembly resolution 67/146 (1), the World Health Assembly Resolution 61.16 (2) and the 2008 Interagency Statement (3), which call upon UN Member States to enact comprehensive and multi-disciplinary national action plans and strategies towards the elimination of the practice. Identifying effective strategies across sectors is an important step in ending FGM.

The health system, defined as all organizations, institutions and resources that produce actions whose primary purpose is to improve health(4), has an important role to play not only in managing complications of FGM but also in preventing the practice. Health care providers, specifically nurses and midwives who constitute most of the health workforce, are highly respected members of FGM practising communities and could positively contribute to abandonment efforts (5,6). However, there is currently limited evidence to guide health programming on FGM prevention (7). In addition, some health care providers are themselves supportive of this harmful practice, and might even perform it (i.e., FGM medicalization), despite national laws and medical ethics forbidding it (8–11). Developing evidence-based tools to build skills of health care providers and address their underlying beliefs could contribute to FGM abandonment efforts and complement existing resources on management of complications (12,13) to ensure comprehensive and high quality care.

For peer review only - http://bmjopen.bmj.com/site/about/guidelines.xhtml

Three countries (Guinea, Somalia, and Kenya) participated in a cluster randomized trial to test the effectiveness and implementation of a health system strengthening approach to FGM, which included the testing of an intervention to build skills of health workers on applying person-centered communication (PCC) for the prevention of FGM (14). Study countries were selected based on their high national and/or sub-national FGM prevalence. The national prevalence of FGM among women and girls aged 15 - 49 years is 98% in Somalia, 97% in Guinea and 21% in Kenya according to national population-based surveys. There are 20 hotspot counties/sub-national administrative units in Kenya with a prevalence of >80% (15), and this study focused on three of these counties. Likewise, the study countries have high rates of medicalized FGM, performed primarily by midwives, who make up between 71% to 93% of primary health care providers in the three study countries (16) hence the selection of nurses and midwives as the target group for this intervention.

The purpose of this study was to test a two-level intervention package to enable ANC providers to deliver person-centered FGM counseling to their clients.[1] This intervention package was informed by a theory of change that promotes health workers to be effective behavioral change agents because of their credibility (17) and positionality to influence the opinions, attitudes, beliefs, motivations and behaviors of their clients (18). We hypothesized that if ANC providers gained the necessary knowledge and skills to provide person-centered counseling (Level 2) and were given the opportunity to question their beliefs and attitudes together with an enabling environment (Level 1), they could positively influence the knowledge and attitudes of their clients to abandon the practice (Supplementary file 1).

The level one intervention consisted of making available national policy directives on the role of health care providers in providing FGM prevention and care services, WHO's FGM guidelines and clinical handbook as well as information, education, and communication (IEC) materials. These materials were distributed without any capacity building to accompany their distribution. Level two

For peer review only - http://bmjopen.bmj.com/site/about/guidelines.xhtml

consisted of an interactive training specifically targeting ANC providers to build their knowledge on FGM, enable them to question their FGM-related values and attitudes and build their skills on counseling for FGM prevention using person-centred communication (19), a component of person-centred care, which ensures that the perspectives and preferences of individuals, carers, families and communities are at the center of decisions and that they have the information and support needed to make decisions (20). ANC providers were trained to apply a series of structured steps in which they would: 'Assess' their client's views on FGM, address and challenge her 'Beliefs', encourage 'Change' and together with the client, 'Discuss and Decide' (ABCD).

METHODS

Study Design

This cluster randomized trial applied a type 2 hybrid, effectiveness-implementation design (21) to test the effectiveness of the delivery of a phased intervention package (Level 1 and 2) on knowledge, attitudes and practices among ANC health workers and their clients. This type of implementation research design assesses the effectiveness of the intervention and implementation factors in real world settings. The methodology, analysis plan and reporting conformed to the Consolidated Standards of Reporting Trial (CONSORT) 2010 statement: extension for cluster randomized trials checklist (22). Ethical approval for the master protocol was obtained from the World Health Organization (WHO) Ethical Review Committee (ERC) (#P151/03/2014). Each study country submitted country-specific protocols to local institutional review boards. Ethical approval was obtained in Kenya from the Kenyatta National Hospital/University of Nairobi ERC (P805/09/2019) and the National Commission for Science, Technology, and Innovation (NACOSTI/P/20/5721); in Somalia from the Department of Planning, Policy and Strategic Information, Unit of Research (MOHD/DG: 2/11526/2019); and in Guinea from the *Comité National d'Ethique Pour la Recherche en Santé* (CNERS) (105/CNERS/19).

For peer review only

For peer review only - http://bmjopen.bmj.com/site/about/guidelines.xhtml

Participants

Within each study country, two or three sub-national units (regions/counties) were purposively selected according to the following eligibility criteria: (1) FGM prevalence >50% among females 15 - 49 years old; (2) more than 15 ANC clinics, seeing on average 30 new ANC clients per month and (3) accessibility in terms of security. The unit of randomization was the ANC clinic to avoid having ANC providers in the same clinic in different study arms, which could lead to contamination. In intervention sites, all providers on duty were pre-screened. To ensure participation and follow-up throughout the trial, between one and three ANC providers on duty were enrolled based on a six-month clinic rotation schedule provided by the clinic manager. Ten new clients exiting their first ANC consultation with a participating provider were recruited at each data collection point.

Individual study participants gave verbal informed consent. Data collectors collected data from the ANC providers and their clients in a private and confidential setting. While personally identifiable information was collected from ANC providers to facilitate tracking during the follow-up data collection time points, data were de-identified prior to analysis. No personally identifiable information was collected from ANC clients who were unique at each time point. Participating ANC clients received the equivalent of 5 USD to compensate for their transport costs recognizing that participants consenting to participate might have changed their plans to accommodate the interviews. Given insecurity in carrying cash in Somalia, a mobile phone application was used to transfer the money to participants, an amendment to the original protocol, which was submitted to the ethical review committees.

Randomization and blinding

Based on Ministry of Health (MoH) facility administrative records, all public, primary care facilities (i.e., dispensaries and/or health centers) offering ANC services in the selected regions/counties the average number of new ANC clients seen in November and December 2019 was compiled to create

For peer review only - http://bmjopen.bmj.com/site/about/guidelines.xhtml

ordered listings of client loads at each of the sites by region/county. Clinics were matched into pairs based on client load so the two busiest would be randomized to different arms and so on. A uniform distribution was used for randomization using the uniform random number function in STATA 17 (StataCorp Inc., College Station, TX, USA). Study teams organized data collection and intervention trainings based on the randomization lists. Attempts were made to blind clinic managers, ANC providers and their clients to study arm allocation. Since both study arms received the level one intervention component at baseline, and the providers and managers at control sites were unaware of the training that took place at intervention sites, it is conceivable that they were not aware of their study arm. Presumably, intervention clients would assume they were the intervention arm, but they were also not aware of what might have been offered to other sites. ANC clients, however, were completely blinded as to study arm allocation since a distinct set of clients was interviewed at each time point, and they would not be aware of the training the provider had had. Field data collectors were also blinded to study arm allocation as much as possible, although some might have determined intervention arm during the study.

Procedures

Implementation of the study interventions and data collection occurred between August 2020 and September 2021 and was staggered by countries. In the intervention arm, data collection was undertaken at three time points, i.e., at baseline prior to implementing the level one intervention component; at month three, prior to implementing the level two intervention component and at month six. In the control arm, data collection was done at two time points, i.e., at baseline and at month six. Study instruments included one for ANC clients, one for health workers and a health facility checklist completed by clinic managers. Instruments were pretested among ANC clients and providers from non-participating sites in all countries, and country teams provided feedback on the structure and appropriateness of each question prior to finalizing the instruments.

For peer review only - http://bmjopen.bmj.com/site/about/guidelines.xhtml

A web-interface electronic data capture system was developed on the Kobo toolbox core system architecture (Kobo Toolbox, Harvard Humanitarian Initiative, Boston, Massachusetts, USA). User accounts were password-protected, and data sent to the server was encrypted in transit using SHA256 with RSA encryption that met the data security requirements. Personally identifiable information was not collected, and all records were anonymized with unique study numbers. Study instruments for ANC clients were translated from English into ten languages by research team members in consultation with language experts (French, Somali, Swahili, Soussou, Poular, Malinké, Keiyo, Maasai, Marakwet and Tugen) while those for ANC providers and clinic managers were translated into two languages (French and Somali). No backtranslation was performed. Field data collectors and their supervisors spoke the languages in which the questionnaires were administered. Data collection teams participated in a standardized training with WHO/HRP and the research institutions in each country. The level two intervention was implemented by master trainers in each country who had been trained remotely over a three-day period following the WHO PCC for FGM prevention facilitator's manual.

Outcomes

The primary study outcome was delivery of the "ABCD" approach by ANC providers measured by responses from their client using tools developed for this study based on previously validated instruments, including four constructs of the operational definition of person-centered communication (23). We also assessed ANC provider delivery of FGM care services and their utilization of the level one intervention components. Health facility preparedness to offer FGM prevention and care was assessed using a composite score developed for this study. (Supplementary file 2).The secondary self-efficacy outcome was assessed based on a score calculated from a validated tool for measuring general self-efficacy (24) while knowledge, attitudes, and practice (KAP) on FGM prevention and care were

For peer review only - http://bmjopen.bmj.com/site/about/guidelines.xhtml

measured using an unvalidated KAP questionnaire similar to one used in formative research in Guinea. Study instruments can be found in Supplementary file 3

Statistical analysis

To have sufficient power (80%) to detect a difference (significance level 5%) between intervention and control arms on the primary study outcome of delivery of the PCC intervention for FGM prevention, 180 ANC clinics, equally divided across the three study countries were recruited and randomized with 1800 new ANC clients (10 per clinic) recruited at baseline and 1800 at six-month follow-up. While similar interventions have resulted in 20% difference between groups (25), a 10% difference (based on an assumed 20% in the control arm and 30% in the intervention arm) was applied to ensure sufficient power to detect a 10% difference and considering the minimal levels of clinical efficacy for such an intervention to be practical. This sample size also allowed for a 10% non-response and/or loss to follow-up rate and accounted for a clustering effect of (ICC=0.20) at clinic level. A relatively high level of clustering was assumed in the sample size calculations to not underestimate the needed sample size. Region/county level was not included in the multilevel model due to the low number of included regions/counties per country (Kenya 3, Guinea 2, Somalia 3) and it would then not be possible to get an accurate estimate of the variance between clusters.

Data were analyzed using STATA 17 software following a per-protocol approach. Data from ANC providers and their clients were analyzed if the clinic had at least one provider with follow up data at all study time points, and in the intervention arm, if the ANC provider present had undergone training on PCC for FGM prevention at month three. Clinics where providers were lost to follow-up were not included in the final analyses. All facility checklists and ANC client exit interviews were conducted as intended except at sites not accessible due to security issues or closed or converted for care of COVID-19 patients during the pandemic. As the study was designed to pre-screen ANC providers at baseline and

For peer review only - http://bmjopen.bmj.com/site/about/guidelines.xhtml

include in the final analytic sample only those clinics and providers who were available at 3 and 6 months, an intention-to-treat approach was not feasible. Key characteristics of the participating facilities, providers and clients were summarized. Providers and clinics that were screened but not eligible are compared in Supplementary file 4.

Continuous variables are presented using mean values, and standard deviation (SD) while categorical variables are summarized as counts (N) with percentages (%). Differences in proportions were analysed for dichotomous outcomes using Fischer's exact test. For outcomes measured as summary scores, comparisons of mean scores are presented across study arms using t-test.

Initial analyses showed that the clustering was negligible at the provider level since most sites only included one provider in the study. Therefore, multilevel regression models were not used to compare outcomes among providers in intervention vs. control arms. However, analyses based on client level outcomes applied multilevel mixed effect logistic regression models to assess differences between the study arms. Multilevel analyses were attempted for the models in which ANC clients reported on provider actions, but given the complexity of the models, convergence problems arose leading to unreliable results. In these cases, results of ordinary models are presented. Linearity was assessed for the continuous covariates included in the regression models using the Box-Tidwell test in Stata.

At month six, a comparison of study outcomes between the intervention and control arms was used to determine the combined effect of both levels of the intervention package. Multilevel multivariable logistic regression analyses for ANC provider outcomes were adjusted for their sex, years of service, FGM status, FGM-related training, any specific training on communication/counseling and PCC, and whether the provider had conducted FGM in the past. Analyses related to ANC client outcomes were adjusted for their age, educational level, FGM status and exposure to level one IEC materials. These variables were determined a priori based on previously published literature. Analyses

For peer review only - http://bmjopen.bmj.com/site/about/guidelines.xhtml

related to provider actions as reported by clients were adjusted for client characteristics as it was not possible to definitively link a client with a particular provider. Unadjusted analyses are presented for outcomes that relate to composite measures based on ANC provider and client responses (e.g., provision of FGM prevention and care services).

To determine the separate effect of the two levels of the intervention package, additional sub-analyses were conducted restricted to the intervention arm. Changes from baseline to month 3 within the intervention arm were used to determine the effect of the level one intervention component while changes from month 3 to month 6 within the same study arm were used to determine the effect of the level two intervention component. The study was not powered for these sub-analyses, however, and these results are presented in Supplementary file 4.

In-country data managers monitored data quality. Periodic data audits were conducted by the WHO/HRP Quantitative Assessment and Data Management team to identify any data collection gaps and data discrepancies requiring follow up by in-country teams. Weekly data monitoring meetings were held between the in-country research teams and WHO/HRP staff during data collection periods to identify, document and resolve any data discrepancies. These were virtual due to the COVID-19 pandemic. Given that there was no prospective follow-up of clients, a Data Safety and Monitoring Board was not established. Instead, local research teams documented and reported any unintended harms and/or protocol deviations to the WHO/HRP study coordination team.

Patient and public involvement statement

Health care providers and members of communities where the practice of FGM is prevalent in the study countries were actively involved in the design and implementation of this study intervention. This included the formative research conducted in Guinea, which identified health care providers as integral members of FGM practicing communities who understand local community beliefs and norms,

For peer review only - http://bmjopen.bmj.com/site/about/guidelines.xhtml

making them effective change agents. The formative research also found that the health sector can support these health care providers to be effective change agents by incorporating FGM content within their training, ensuring accountability to legal and policy standards and promoting FGM abandonment as part of a multi-sectoral approach. Based on this formative work, the PCC training was developed and subsequently piloted among ANC providers in Kenya before being rolled out as part of the multi-country study.

Additionally, the research partners in Guinea, Kenya and Somalia actively engaged health care providers and community members as part of their in-country work towards FGM prevention. In Kenya, as part of mobilization of study participants, community health volunteers in the study counties talked about the study during their community sensitization sessions and invited pregnant women to attend routine ANC sessions where they could be approached for participation in the study. Both health care providers and pregnant women were provided with information about the study, including the burden of the intervention as to time, any risks involved in their participation, the voluntary nature of their participation, and were recruited only after providing informed consent.

At present, study dissemination meetings have been conducted in Kenya and Guinea that have involved the MoH, other stakeholders as well as representatives of health care providers and community members where the study was implemented. In these meetings, the in-country research partners have led the development of policy briefs identifying country-specific results relevant for local research needs, policy development and practice.

Role of the funders

Apart from WHO/HRP, the study funders had no role in study design or implementation. WHO/HRP, in collaboration with in-country research teams, developed the study protocol, provided data management and analytic support, and contributed to interpretation and manuscript writing.

For peer review only - http://bmjopen.bmj.com/site/about/guidelines.xhtml

WHO/HRP coordinated the successful implementation of this study. The data collection platform was developed and maintained by an outsourced vendor (First Data, LLC, Kenya); data management was coordinated by the local implementing partners (CERREGUI, DARS and University of Nairobi) and statistical data analysis was conducted by an external statistician (Dr. Max Petzold, Gothenburg University). All these functions were conducted with utmost integrity following ICH-GCP guidelines.

RESULTS

Recruitment and retention

Between August 2020 and September 2021, a total of 180 ANC clinics (i.e, 60 clinics per study country) were enrolled and randomized to intervention and control arms. There was some natural staggering of the start and subsequent data collection dates due to factors, such as weather, COVID-19, Ramadan, and national elections. Data collection periods ranged from three to six weeks in each country at each time point. The time elapsed between the end of one data collection period to the beginning of the next data collection period ranged from three to five months.

In the intervention arm, 216 providers and 900 clients (i.e.., 10 per clinic) were interviewed. Based on a review of clinic rotation schedule to ensure participation of at least one provider from each study clinic throughout the trial, 133 providers were enrolled. In the control arm, 220 providers and 900 clients were interviewed. (Figure 1). At month three, data were collected at 98% (n=88) of the intervention clinics as two clinics in Kenya were inaccessible due to insecurity. One hundred and thirty (98%) ANC providers (at least one from each site) and 880 first visit ANC clients completed the month three questionnaires prior to implementing the Level 2 intervention PCC. No data collection was conducted at the control sites. At month six, 91% (n=163) of ANC clinics (81, intervention and 82, control) had at least one ANC provider (intervention n=110 and control n=122) on duty who was

For peer review only - http://bmjopen.bmj.com/site/about/guidelines.xhtml

previously enrolled in the study. The client questionnaire was applied to 819 and 810 first visit ANC clients, respectively in the intervention and control sites.

Characteristics of study sites and participants

The 163 ANC clinics retained to the end of the study, had a mean of four ANC providers (standard deviation, SD: 3) and served on average 155 new ANC clients per month (SD: 127) with a mean catchment population of 36,754 people (SD: 126,082). In 55% (n=89) of clinics, the clinic manager reported that there were no activities promoting FGM prevention in the facilities' catchment area (*Table 1*). These characteristics were not different from the 17 ANC clinics that were enrolled at baseline but that subsequently were not included in the final analysis (Supplementary file 4).

Of the 232 ANC providers who contributed data for analysis at month six, 83% (n=193) were female and their mean age was 36 years (SD: 10 years). They had an average of eight years professional experience (SD: 7 years) and 68% (n=158) had studied up to Diploma level (generally 3 years post-secondary education) with 90% (n=208) identifying as either midwives, nurses, or nurse-midwives. Health cadres were defined by national licensing requirements in each country. Among these providers, at baseline, 63% (n=146) reported that they had not received formal clinical training on FGM prevention and care (*Table 2*). Almost two-thirds (64%, n=149) reported that they had received training on communication/counselling while half (51%, n=118) had received training on person-centered care. Further, 54% (n=126) of female providers reported that they had undergone FGM while overall, 94% (n=217) of providers reported that they had never performed FGM. These characteristics were not different when compared to the ANC providers who were on duty in the 180 ANC clinics enrolled at baseline (Supplementary file 4). The mean age of the 1,800 clients exiting their first ANC visits at baseline was 26 years (SD: 6 years), 47% (n=846) reported not having received any education, and 73% (n=1,320) reported that they had undergone FGM. These characteristics were similar to the 880 and

For peer review only - http://bmjopen.bmj.com/site/about/guidelines.xhtml

1,630 first visit ANC clients interviewed at month three (intervention arm only) and month six, respectively (*Table 3*).

To evaluate potential bias from differential selection of providers receiving the intervention, we assessed differences in baseline characteristics between the 133 ANC providers from intervention facilities who were screened at baseline and received PCC training at month three (i.e., included in the analytic sample) versus the 97 who were screened and did not receive the intervention (i.e., excluded from analytic sample). The reasons for this included the fact that some of the providers had been transferred from the study clinics or could not be released to attend the training so as not to affect service delivery. Both groups were similar in terms of sex, educational level, professional cadre, as well as whether they had undergone or recently performed FGM. However, included providers tended to be slightly younger (by two years on average) and less likely to be of Muslim religion, although the question on religion was not administered for the Somalia sample since all respondents were assumed to be Muslim (Supplementary file 4).

ANC providers implementation of ABCD elements of the PCC approach

Table 4 presents the analysis of study outcomes by arm at month six. Compared to ANC providers in the control arm, those in the intervention arm were nearly nine times as likely to ask their clients if they had undergone FGM (OR: 8.9, 95% CI: 6.9-11.5; p<0.001), nearly ten times as likely to ask their clients' personal beliefs regarding FGM (OR: 9.7, 95% CI: 7.5-12.5; p<0.001), more than nine times as likely to discuss with their clients why FGM should be prevented (OR: 9.2, 95% CI: 7.1-11.9; p<0.001) and nearly eight times as likely to discuss with their clients how FGM could be prevented (OR: 7.7, 95% CI: 6.0-9.9; p<0.001). Further, ANC clients in the intervention arm were nearly seven times as likely to report that they were satisfied with how FGM had been addressed by their provider during the clinic visit compared to those in the control arm (OR: 6.6, 95% CI: 5.1-8.4; p<0.001). In the

For peer review only - http://bmjopen.bmj.com/site/about/guidelines.xhtml

intervention arm, the mean score of implementing the ABCD elements of the PCC approach was more than twice as likely (OR: 2.1, 95% CI: 1.6-2.6; p<0.001) to be higher in the intervention [3.9 (3.8-4.0)] compared to the control arm [1.6 (1.5-1.8)].

ANC clinic preparedness to provide FGM prevention and care services

A significantly higher proportion of ANC clinics in the intervention arm had all correct repornses related to facility preparedness to provide FGM prevention and care services compared to those in the control arm (68% vs. 27%, p<0.001). Additionally, ANC clinics in the intervention arm had a significantly higher mean score for preparedness compared to those in the control arm [3.4 (95% CI: 3.2-3.6) vs. 2.6 (95% CI: 2.4-2.9; p<0.001)].

ANC providers utilizing level one intervention components

A higher proportion of ANC providers in the intervention arm reported having utilized the level one intervention package components compared to those in the control arm (83% vs. 56%, p<0.001). In multivariable analyses, ANC providers in the intervention arm were nine times as likely to report having utilized the level one intervention package components compared to those in the control arm (AOR: 9.3, 95% CI: 4.2-20.8; P<0.001).

ANC providers offering appropriate FGM prevention and care services

At month six, based on a cumulative score to specific questions on provision of appropriate FGM-related prevention and care services, a higher proportion of ANC providers in the intervention arm reported that they had provided FGM prevention and care services correctly compared to those in the control arm (45% vs. 34%, p=0.03).

ANC providers' confidence, self-efficacy, and communication skills

A higher proportion of ANC providers in the intervention arm reported being confident in their knowledge to provide FGM prevention and care services compared to those in the control arm (98% vs.

For peer review only - http://bmjopen.bmj.com/site/about/guidelines.xhtml

89%, p=0.005). In multivariable analysis, ANC providers in the intervention arm had more than six times the odds of reporting being confident in their knowledge to provide FGM prevention and care services compared to those in the control arm (AOR: 6.3, 95% CI: 1.4-28.9; p=0.02). Self-efficacy was generally high (scores 7.4 – 7.8 out of 8) with no significant difference in high scores between study arms (85% vs. 82%, p=0.36 and OR: 0.8, 95% CI: 0.4-1.6); p= 0.50).

ANC providers' knowledge, attitudes and support for FGM/medicalized FGM

The mean correct scores for FGM-related knowledge were higher among ANC providers in the intervention arm compared to the control arm (2.5, 95% CI: 2.2-2.8 vs. 1.9, 95% CI: 1.7-2.2; p=0.005) but 8% vs. 2% (p=0.16) had correct responses on the FGM-related knowledge questions, showing low knowledge overall, and particularly on the FGM typology. Providers had similarly unsupportive attitudes towards FGM in both groups and similarly unsupportive attitudes about medicalized FGM with most providers reporting that they did not support FGM (82% vs. 85%, p=0.73) and/or medicalized FGM (72% vs. 73, p=0.94%).

ANC clients' support for FGM, intention to have their daughters undergo FGM and being involved in FGM prevention efforts

Compared to those in the control arm, a higher proportion of ANC clients in the intervention arm reported being less supportive of FGM after their month six clinic visit (52% vs. 29%, p<0.001). In multivariable analysis, ANC clients in the intervention arm had more than twice the odds of reporting that they were strongly opposed to FGM (AOR: 2.4, 95% CI: 1.1-5.2; p=0.023, ICC: 0.61). When asked about their support for FGM after the ANC visit compared to before, clients in the intervention arm had more than five times the odds of being less supportive of FGM compared to those in the control arm (OR: 5.4, 95% CI: 2.4-12.4; p<0.001, ICC:0.66). ANC clients in the intervention clinics had lower odds of intending to have their daughters undergo FGM (OR: 0.3, 95% CI: 0.1-0.7; p=0.004, ICC: 0.60) or of

For peer review only - http://bmjopen.bmj.com/site/about/guidelines.xhtml

wanting a health care provider to perform FGM (OR: 0.2, 95% CI: 0.1-0.5; p<0.001, ICC: 0.54) and higher odds of reporting that they wished to be active in FGM prevention (OR: 3.2, 95% CI: 1.6-6.2, p=0.001, ICC: 0.50).

To understand the impact of the level one intervention relative to the level two intervention, a comparison of study outcomes restricted to the intervention arm was done between baseline and month three and between months three and six (Supplementary file 4). Although not statistically powered for this analyses, we found that a significantly higher proportion of ANC clients in the intervention arm reported that their provider had asked about the different PCC components at month three versus basleine and at month six versus month three. Similarly, a significantly higher proportion of ANC clinics in the intervention arm were prepared to provide FGM-related prevention and care services at month three compared to baseline and at month six compared to month three. No statistically signiofiocant differences were seen in the proportion of ANC providers with the secondary outcomes apart from high confidence scores seen between month six and month three. Finally, ANC client outcomes were significantly higher among intervention clients in month three versus baseline and in month six versus month three.

DISCUSSION

The results of this cluster randomized trial show that an intervention to strengthen health facility preparedness while building skills of ANC providers to communicate using a person-centred counselling technique on FGM prevention was effective. ANC providers exposed to the intervention had increased confidence, improved FGM-related knowledge, and effective delivery of FGM prevention and care services. Additionally, ANC clients who had received care from these providers were less supportive of FGM and had reduced intentions to perform FGM on their daughters. This study provides evidence of a practical intervention to engage health care providers in FGM abandonment efforts whilst also providing

For peer review only - http://bmjopen.bmj.com/site/about/guidelines.xhtml

quality care to FGM survivors. This study provides evidence of how to effectively build the capacity of health care providers at primary care to address FGM(26), an area identified as a critical gap during the formative research.

The PCC training modules strengthened ANC providers' skills on FGM prevention and care and helped to clarify their beliefs and attitudes, which are key drivers of FGM (27). We did not find notable changes in knowledge and attitudes among ANC providers. The knowledge scores overall were low, and upon further investigation, it appears that questions on typology captured through visually drawn images on a tablet device were consistently answered incorrectly. These results perhaps show measurement and knowledge limitations but do not necessarily relate to service provision or quality of care. Attitudes in the intervention and control groups were generally unsupportive of FGM and do not appear to be heavily impacted by the training intervention. Exposure to the intervention package also did not improve ANC providers' self-efficacy towards FGM prevention and care. This may be related to the lack of support for FGM and/or its medicalization and high self-efficacy among nearly all providers throughout the study in both study arms, a finding that was also noted in formative research conducted in Guinea (28,29). In the formative phase, while the vast majority of health workers were opposed to the practice, 38% also felt that FGM limited promiscuity and 7% believed that it was a good practice, showing ambivalence and complexity in attitudes about FGM among health providers. Other studies have found that some providers support the perpetuation of the practice and even planned to have their own daughters undergo FGM or to perform it on their clients(30).

The findings in this study underscore the importance of addressing values and attitudes of both providers and clients as a means of achieving positive behavioral change. Changes observed among ANC providers were sustained across the study duration and ultimately, and importantly, resulted in

For peer review only - http://bmjopen.bmj.com/site/about/guidelines.xhtml

reported changes in attitudes and intentions of their clients. However, this study design did not allow us to determine whether the attitudinal changes observed among ANC clients were sustained after their clinic visit or translated into positive change in FGM prevention.

The application of these study results into programming will need to consider several factors. Firstly, the study sites were primary care facilities located in high FGM prevalence settings. The results of this intervention may not be generalizable to settings where FGM is less prevalent or to settings other than primary care. Secondly, first ANC visits are not typical of other health visits since the consultation is generally longer with a greater focus on health promotion messaging. While this is an ideal setting for implementing such an intervention, its application to other health settings and among other population groups is not known. During scale up, if the PCC approach is applied among clients seeking other sexual and reproductive health services or parents bringing their children to child immunization and wellness visits, it will be important to consider time requirements for the delivery of the 'ABCD' steps, especially in high volume clinic settings.

Thirdly, while the study found a positive impact of the PCC training on health care providers' delivery of person-centred FGM prevention counselling, the continuity and quality of FGM prevention counselling in the long-term is not known. Specifically, it will be important to assess subsequently whether providers will continue to provide prevention counselling on an ongoing basis, whether they will share their learnings with family and community members and whether clients will follow through with their intentions to not have their daughters undergo FGM. It may be important to include a supervisory mentorship component to ensure implementation of this intervention (31) in order to strengthen PCC communication practice and quality.

Limitations

For peer review only - http://bmjopen.bmj.com/site/about/guidelines.xhtml

The implementation of this multi-country study was not without challenges and limitations. First, initiation of field data collection activities was delayed by the global COVID-19 pandemic in 2020 – 2021 and required some modification to trainings of the data collection teams, the master trainers and the ANC providers receiving the PCC intervention. This may have impacted the overall effectiveness of the intervention.

Second, to attempt to ensure participation of at least one provider at each site, all providers were pre-screened at baseline and clinic rotation schedules determined enrollment into the study. Selection bias might have been introduced through this process. The exploratory analysis to assess for selection and attrition bias from the pre-screen step, did not reveal significant differences between included and excluded health workers except for slightly lower age (Supplementary file 4),and a per protocol analysis wasrequired, but it is possible that differences in other unmeasured factors related to the clinics and providers might have biased the results. Findings from a process evaluation conducted as part of this study will provide additional insights on the feasibility, acceptability, appropriateness, and fidelity of the intervention implementation in these contextual settings to inform further implementation and scale up.

Third, we did not perform adjustment for multiple testing in our analysis given that the different tests are interpreted separately and no overall conclusion will be stated. Given that the null hypotheses of no differences are true, we estimate that the overall type one error rate is higher than the individual test level of 0.05. In terms of assumptions regarding clustering, sample size was calculated based on an ICC of 0.20. However, the observed ICC:s were all above 0.50 leading to statistically conservative conclusions of the non-significant results due to being under-powered to find an association.

Finally, we acknowledge that there are many factors that could impact FGM-related decision-making and a positive and impactful interaction with a respected health care provider might not be

For peer review only - http://bmjopen.bmj.com/site/about/guidelines.xhtml

sufficient to lead to actual changes in community behavior. However, the study design enabled us to compare similar sites to identify the relative effect of this approach since both intervention and control sites would be exposed to similar factors, and clients at these sites would face similar complexities in decision-making.

Conclusion

In conclusion, this study highlights the importance of addressing the values and beliefs of health care providers working at primary care level, who are subject to social norms around FGM that may conflict with medical ethics and national laws and policies as an intermediary step in preventing FGM. Empowering these health care providers with communication skills and engaging them as opinion leaders can be impactful in changing their clients' attitudes towards FGM. In conjunction with FGM prevention activities in other sectors, this intervention can contribute to positive change if brought to scale.

For peer review only - http://bmjopen.bmj.com/site/about/guidelines.xhtml

DECLARATIONS

Contributors

WA and CP conceptualized the study and prepared the protocol in collaboration with VM, KS, PN, TE, MDB, AMS, AD(1) and MAA. MDB, AMS, AOS, PN, TE, JMK, AD(1) and MAA provided oversight over study implementation while AD(2), JK and SA monitored data quality in countries and KN and SST monitored data quality across countries. VM prepared the first draft of the manuscript with input from WA and CP, the responsible officer of the study at WHO/HRP. MP developed the statistical analysis plan and conducted data analysis. KS coordinated the development of the PCC for FGM prevention training. KS, PN, TE, JMK, JK, MDB, AMS, AOS, AD(1), AD(2), SA, and MAA contributed to and reviewed the manuscript for proper intellectual content. All authors read and approved the final draft of this manuscript.

Declaration of interests

The authors declare that they have no competing interests.

Data sharing

De-identified dataset will be retained in the WHO HRP electronic archival system. Any use of the de-identified analytic dataset for secondary research purposes will be governed by the WHO data use regulation. Request for data dictionary and for dataset may be sent to pallittoc@who.int

Acknowledgements

The authors would like to acknowledge the funders, the WHO country office colleagues, Dr Bernadette Dramou, Dr Cécé-Vieux Kolie, Dr Joyce Lavussa, Ms Matilda Cherono, Ms Asia Ahmed Osman in the study countries as well as MoH and national FGM stakeholders in the development of the research protocol, implementation of study interventions and field data collection. We also thank Professor Joanna Schellenberg of the London School of Hygiene and Tropical Medicine and Dr Christina

For peer review only - http://bmjopen.bmj.com/site/about/guidelines.xhtml

Atchison of Imperial College London for their input in conceptualizing the study as well as Dr Leyla Hussein for supporting pilot testing of the PCC intervention.

Funding

This work received funding from the Governments of Norway and the United Kingdom of Great Britain and Northern Ireland as well as the UNDP-UNFPA-UNICEF-WHO-World Bank Special Programme of Research, Development and Research Training in Human Reproduction (HRP), a cosponsored programme executed by the World Health Organization (WHO).

Ethics approval:

The following research ethics committees approved the protocol:

1. World Health Organization (WHO) Ethical Review Committee (ERC) (#P151/03/2014).

2. Kenya: Kenyatta National Hospital/University of Nairobi ERC (P805/09/2019) and the National Commission for Science, Technology, and Innovation (NACOSTI/P/20/5721)

3. Somalia: the Department of Planning, Policy and Strategic Information, Unit of Research (MOHD/DG: 2/11526/2019)

4. Guinea: the Comité National d'Ethique Pour la Recherche en Santé (CNERS) (105/CNERS/19).

Disclaimer

The named authors alone are responsible for the views expressed in this publication and do not necessarily represent the decisions or the policies of the UNDP-UNFPA-UNICEF-WHO-World Bank Special Programme of Research, Development and Research Training in Human Reproduction (HRP) or the World Health Organization (WHO).

For peer review only

For peer review only - http://bmjopen.bmj.com/site/about/guidelines.xhtml

For peer review only

REFERENCES

1. United Nations General Assembly, Intensifying global efforts for the elimination of female genital mutilations : resolution/ / adopted by the United Nations General Assembly, 5 March 2013, A/RES/67/146, available at: https://www.refworld.org/docid/51e67bc64.html [accessed 4 May 2022]
2. World Health Assembly, WHA resolution 61.16. 2008. United Nations: New York. 2008. Available at https://apps.who.int/gb/ebwha/pdf_files/WHA61-REC1/A61_REC1-en.pdf , New York. 2008.
3. Eliminating female genital mutilation: an interagency statement-OHCHR, UNAIDS, UNDP, UNECA, UNESCO, UNFPA, UNHCR, UNICEF, UNIFEM, WHO. WHO: Geneva.World Health Organization, 2008.
4. World Health Organization. The World Health Report: 2000 :health systems : improving performance [Internet]. Geneva: World Health Organization; 2000. Available from: https://apps.who.int/iris/handle/10665/42281
5. Pallitto CC, Ahmed W. The role of the health sector in contributing to the abandonment of female genital mutilation. Med (N Y) [Internet]. 2021 May 14 [cited 2023 May 15];2(5):485–9. Available from: https://pubmed.ncbi.nlm.nih.gov/35590230/
6. Kimani S, Esho T, Kimani V, Muniu S, Kamau J, Kigondu C, et al. Female Genital Mutilation/Cutting: Innovative Training Approach for Nurse-Midwives in High Prevalent Settings. Obstet Gynecol Int [Internet]. 2018 [cited 2023 May 15];2018. Available from: https://pubmed.ncbi.nlm.nih.gov/29736171/
7. Johansen REB, Diop NJ, Laverack G, Leye E. What works and what does not: a discussion of popular approaches for the abandonment of female genital mutilation. Obstet Gynecol Int [Internet]. 2013 [cited 2023 May 15];2013:1–10. Available from: https://pubmed.ncbi.nlm.nih.gov/23737795/
8. Njue C, Askew I. Medicalization of female genital cutting among the Abagusii in Nyanza Province, Kenya. Reprod Health [Internet]. 2004 Jan 1 [cited 2023 May 15]; Available from: https://knowledgecommons.popcouncil.org/departments_sbsr-rh/32
9. Shell-Duncan B, Gathara D, Moore Z. Female genital mutilation/cutting in Kenya: Is change taking place? Descriptive statistics from four waves of Demographic and Health Surveys. Reprod Health [Internet]. 2017 Jan 1 [cited 2023 May 15]; Available from: https://knowledgecommons.popcouncil.org/departments_sbsr-rh/596
10. Umar AS, Oche OM. Medicalization of female genital mutilation among professional health care workers in a referral hospital, north-western Nigeria. J Reprod Biol Health. 2014;2(1):2.
11. Doucet MH, Pallitto C, Groleau D. Understanding the motivations of health-care providers in performing female genital mutilation: an integrative review of the literature. Reprod Health [Internet]. 2017 Mar 23 [cited 2023 May 15];14(1):46. Available from: https://pubmed.ncbi.nlm.nih.gov/28335771/
12. World Health Organization. Female Genital Mutilation: A Resource Kitkit for the Health Sector. WHO: Geneva, 2021, available at https://srhr.org/fgmresources/https://srhr.org/fgmresources/ - Google Search [Internet]. [cited 2023 May 15].
13. World Health Organization. Reproductive Health and Research. WHO guidelines on the management of health complications from female genital mutilation. :47.
14. Ahmed W, Mochache V, Stein K, Ndavi P, Esho T, Balde MD, et al. A hybrid, effectiveness-implementation research study protocol targeting antenatal care providers to provide female

For peer review only - http://bmjopen.bmj.com/site/about/guidelines.xhtml

genital mutilation prevention and care services in Guinea, Kenya and Somalia. BMC Health Serv Res [Internet]. 2021 Dec 1 [cited 2023 May 15];21(1). Available from: https://pubmed.ncbi.nlm.nih.gov/33522926/

15. National Bureau of Statistics. Republic of Kenya. Kenya Demographic and Health Survey 2014. 2015 [cited 2023 May 15]; Available from: www.DHSprogram.com.
16. NHWA Web portal [Internet]. [cited 2023 May 15]. Available from: https://apps.who.int/nhwaportal/Home/Welcome?ReturnUrl=%2Fnhwaportal%2FHome%2FIndex
17. Boone Tim, Reilly Anthony J., Sashkin M. SOCIAL LEARNING THEORY Albert Bandura Englewood Cliffs, N.J.: Prentice-Hall, 1977. 247 pp., paperbound. Group & Organization Studies [Internet]. 1977 Sep 1;2(3):384–5. Available from: https://doi.org/10.1177/105960117700200317
18. Valente TW, Pumpuang P. Identifying opinion leaders to promote behavior change. Health Educ Behav [Internet]. 2007 Dec [cited 2023 May 15];34(6):881–96. Available from: https://pubmed.ncbi.nlm.nih.gov/17602096/
19. Person-centred communication for female genital mutilation prevention: facilitator's manual. WHO World Health Organization: Geneva, 2022
20. World Health Organization. WHO global strategy on people-centred and integrated health services: interim report [Internet]. Geneva: World Health Organization; 2015. Available from: https://apps.who.int/iris/handle/10665/155002
21. Peters DH, Adam T, Alonge O, Agyepong IA, Tran N. Republished research: Implementation research: what it is and how to do it. Br J Sports Med. 2014 Apr 23;48(8):731–6.
22. Campbell MK, Piaggio G, Elbourne DR, Altman DG. Consort 2010 statement: Extension to cluster randomised trials. BMJ (Online). 2012 Nov 3;345(7881).
23. Epstein RM, Franks P, Fiscella K, Shields CG, Meldrum SC, Kravitz RL, et al. Measuring patient-centered communication in patient-physician consultations: theoretical and practical issues. Soc Sci Med [Internet]. 2005 Oct [cited 2023 May 15];61(7):1516–28. Available from: https://pubmed.ncbi.nlm.nih.gov/16005784/
24. Chen G, Gully SM, Eden D. Validation of a New General Self-Efficacy Scale. Organ Res Methods [Internet]. 2001 Jan 1;4(1):62–83. Available from: https://doi.org/10.1177/109442810141004
25. Keeley RD, Burke BL, Brody D, Dimidjian S, Engel M, Emsermann C, et al. Training to use motivational interviewing techniques for depression: a cluster randomized trial. J Am Board Fam Med [Internet]. 2014 Sep 1 [cited 2023 May 15];27(5):621–36. Available from: https://pubmed.ncbi.nlm.nih.gov/25201932/
26. Balfour J, Abdulcadir J, Say L, Hindin MJ. Interventions for healthcare providers to improve treatment and prevention of female genital mutilation: a systematic review. BMC Health Serv Res [Internet]. 2016 Aug 19 [cited 2023 May 15];16(1). Available from: https://pubmed.ncbi.nlm.nih.gov/27542732/
27. Kimani S, Okondo C, Muteshi-Strachan J, Guyo J. Quality of services offered to women with female genital mutilation across health facilities in a Kenyan County. BMC Health Serv Res [Internet]. 2022 Dec 1 [cited 2023 May 15];22(1). Available from: https://pubmed.ncbi.nlm.nih.gov/35525954/
28. Balde MD, O'Neill S, Sall AO, Balde MB, Soumah AM, Diallo BA, et al. Attitudes of health care providers regarding female genital mutilation and its medicalization in Guinea. PLoS One

For peer review only - http://bmjopen.bmj.com/site/about/guidelines.xhtml

[Internet]. 2021 May 1 [cited 2023 May 15];16(5). Available from: https://pubmed.ncbi.nlm.nih.gov/33983949/
29. Balde MD, Soumah AM, Diallo A, Sall AO, Mochache V, Ahmed W, et al. Involving the health sector in the prevention and care of female genital mutilation: results from formative research in Guinea. Reprod Health. 2022 Dec 1;19(1).
30. Kaplan A, Hechavarría S, Bernal M, Bonhoure I. Knowledge, attitudes and practices of female genital mutilation/cutting among health care professionals in The Gambia: a multiethnic study. BMC Public Health [Internet]. 2013 [cited 2023 May 15];13(1). Available from: https://pubmed.ncbi.nlm.nih.gov/24040762/
31. Pantoja T, Opiyo N, Lewin S, Paulsen E, Ciapponi A, Wiysonge CS, et al. Implementation strategies for health systems in low-income countries: an overview of systematic reviews. Cochrane Database Syst Rev [Internet]. 2017 Sep 12 [cited 2023 May 15];9(9). Available from: https://pubmed.ncbi.nlm.nih.gov/28895659/

For peer review only

For peer review only - http://bmjopen.bmj.com/site/about/guidelines.xhtml

TABLES & FIGURES:

Figure 1: Study CONSORT Diagram

Table 1: Characteristics of ANC clinics included in month six analyses

Table 2: Characteristics of ANC providers included in the month six analyses

Table 3: Characteristics of ANC clients interviewed at each time point

Table 4: Analysis of study outcomes

SUPPLEMENTARY FILES

Supplementary file 1: Theory of change framework

Supplementary file 2: Measurement of study outcomes

Supplementary file 3: Data collection instruments

Supplementary file 4: Additional analyses (Appendices 1 – 3)

For peer review only

Table 1: Characteristics of ANC clinics included in month six analyses

Characteristics	Overall (n=163*)	Intervention (n=82)	Control (n=81)
Number of ANC providers	Mean 4 (SD: 3) Median 3 (1-14, IQR 3)	Mean 4 (SD: 2) Median 3 (1-11, IQR 3)	Mean 4 (SD: 3) Median 3 (1-14, IQR 4)
Average number of ANC clients/month	Mean 150 (SD: 127) Median 118 (3-664, IQR 141)	Mean 148 (SD: 121) Median 117 (3-500, IQR 143)	Mean 152 (SD: 133) Median 120 (3-664, IQR 140)
MoH supervisory visits in the past year	Mean 4 (SD: 3) Median 3 (0-18, IQR 2)	Mean 4 (SD: 3) Median 4 (1-18, IQR 1)	Mean 4 (SD: 3) Median 3 (0-12, IQR 2
Size of catchment population served	Mean 36,754 (SD: 126,082) Median 15,972 (1,000-1,458,000, IQR 24,332)	Mean 23,649 (SD: 35,873) Median 16,022 (1,000-290,000, IQR 22,361	Mean 50,020 (SD: 174,739) Median 15,551 (1,000-1,458,000, IQR 25,544
Presence of anti-FGM activities in the catchment area			
Yes	74 (45%)	43 (52%)	31 (38%)
No	89 (55%)	39 (48%)	50 (62%)
Presence of pro-FGM activities in the catchment area			
Yes	21 (13%)	12 (15%)	9 (11%)
No	140 (86%)	68 (83%)	72 (89%)
Don't Know	2 (1%)	2 (2%)	0 (0%)

* Total of 17 ANC clinics not included: 16 clinics were excluded (7 intervention and 9 control) due to loss-to-follow up (LTFU) of ANC provider i.e., the clinics did not have at least one ANC provider present across all study time points while one ANC clinic in Kenya was never visited at subsequent time points due to issues with insecurity. An ANC provider from one of the clinics in Kenya that had been inaccessible due to insecurity attended the PCC training and was subsequently interviewed.

For peer review only - http://bmjopen.bmj.com/site/about/guidelines.xhtml

Table 2: Characteristics of ANC providers included in the month six analyses

Characteristics	Overall (n=232)	Intervention (n= 115)	Control (n=117)
Age	Mean 36 (SD: 10) Median 34 (20-65, IQR 15)	Mean 35 (SD: 10) Median 33 (20-59, IQR 14)	Mean 37 (SD:11) Median 35 (20-65, IQR 16)
Years of professional experience	Mean 8 (SD: 7) Median 6 (1-39, IQR 7)	Mean 8 (SD:7) Median 6 (1-30, IQR 8)	Mean 8 (SD:7) Median 6 (1-39, IQR 7)
Sex			
Female	193 (83%)	95 (83%)	98 (84%)
Highest educational level			
Certificate	21 (5%)	12 (10%)	9 (8%)
Diploma	158 (68%)	72 (63%)	86 (74%)
Bachelors	44 (19%)	27 (24%)	17 (15%)
Masters & above	1 (0.4%)	0 (0%)	1 (1%)
Other#	8 (3%)	4 (3%)	4 (3%)
Current professional role/title			
Midwife	103 (44%)	53 (46%)	50 (43%)
Nurse	51 (22%)	25 (22%)	26 (22%)
Nurse-Midwife	54 (23%)	27 (24%)	27 (23%)
Other	24 (10%)	10 (9%)	14 (12%)
Received formal training on FGM during clinical training			
Yes	85 (37%)	44 (38%)	41 (35%)
No	146 (63%)	71 (62%)	75 (64%)
Don't Know	1 (0.4%)	0 (0%)	1 (1%)
Timing of clinical training on FGM			
Pre-service	33 (14%)	18 (16%)	15 (13%)
In-service	45 (19%)	22 (19%)	23 (20%)
Both pre- and in-service	7 (3%)	4 (4%)	3 (3%)
Received formal training on communication/counselling			
Yes	149 (64%)	76 (66%)	73 (62%)
No	83 (36%)	39 (34%)	44 (38%)
Received formal training on person-centered care			
Yes	118 (51%)	58 (50%)	60 (51%)
No	113 (56%)	56 (49%)	57 (49%)
Don't know	1 (0.4%)	1 (1%)	0 (0%)
Undergone FGM			

For peer review only - http://bmjopen.bmj.com/site/about/guidelines.xhtml

Yes	126 (54%)	65 (57%)	61 (52%)
No	63 (27%)	27 (24%)	36 (31%)
Don't know	2 (1%)	2 (2%)	0 (0%)
Refused to answer	2 (1%)	1 (1%)	1 (1%)
Conducted FGM			
Yes	15 (7%)	9 (8%)	6 (5%)
Conducted FGM on a girl <18 years			
Yes	14 (6%)	8 (7%)	6 (5%)

For peer review only - http://bmjopen.bmj.com/site/about/guidelines.xhtml

Table 3: Characteristics of ANC clients interviewed at each time point

Characteristics	ANC clients interviewed at Baseline			ANC clients interviewed at Month 3	ANC clients interviewed at Month 6		
	**Overall (n=1800)**	**Intervention (n=900)**	**Control (n=900)**	**Intervention only (n=880)**	**Overall (n=1759)**	**Intervention (n=879)**	**Control (n=880)**
Age	Mean 26 (SD: 6) Median 25 (15-45, IQR 10)	Mean 25 (SD: 6) Median 25 (15-45, IQR 10)	Mean 26 (SD: 6) Median 25 (15-45, IQR 10)	Mean 26 (SD 6) Median 25 (15-45, IQR 10)	Mean 26 (SD: 6) Median 25 (15-45, IQR 9)	Mean 26 (SD: 6) Median 25 (15-45, IQR 9)	Mean 26 (SD: 6) Median 25 (15-45, IQR 10)
Highest educational level							
None	840 (47%)	407 (45%)	433 (48%)	439 (50%)	806 (46%)	384 (44%)	422 (47%)
Primary	484 (27%)	231 (26%)	253 (28%)	239 (27%)	553 (31%)	278 (32%)	275 (31%)
Secondary	331 (18%)	171 (19%)	160 (18%)	157 (18%)	306 (17%)	160 (18%)	146 (16%)
University	95 (5%)	61 (7%)	34 (4%)	25 (3%)	67 (4%)	34 (4%)	33 (4%)
Other[#]	50 (3%)	30 (3%)	20 (25)	20 (2%)	37 (2%)	23 (3%)	14 (2%)
Have you undergone FGM?							
Yes	1320 (73%)	677 (75%)	643 (71%)	645 (73%)	1321 (75%)	655 (75%)	666 (75%)
No	452 (25%)	209 (23%)	243 (27%)	224 (25%)	420 (24%)	206 (23%)	214 (24%)
Don't know	12 (1%)	10 (1%)	2 (0.2%)	5 (1%)	21 (1%)	13 (2%)	8 (1%)
Refused to answer	16 (1%)	4 (0.4%)	12 (1%)	6 (1%)	7 (0.4%)	5 (1%)	2 (0.2%)

For peer review only

For peer review only

For peer review only - http://bmjopen.bmj.com/site/about/guidelines.xhtml

Table 4: Analysis of study outcomes

**Primary Outcomes**					
**ANC clients reporting that their provider implemented components of PCC for FGM prevention approach**					
	**Intervention (n=819)**	**Control (n=810)**	**Adjusted OR# (95% CI)**	**P-value**	**ICC**
Provider asked client if they have undergone FGM	634 (77%)	245 (30%)	8.9 (6.9-11.5)	<0.001	N/A
Provider asked client about the client's personal beliefs regarding FGM	616 (75%)	217 (27%)	9.7 (7.5-12.5)	<0.001	N/A
Provider discussed with client why FGM should be prevented	629 (77%)	244 (30%)	9.2 (7.1-11.9)	<0.001	N/A
Provider discussed with client how FGM could be prevented	592 (72%)	232 (29%)	7.7 (6.0-9.9)	<0.001	N/A
Client satisfied with how FGM was addressed by provider during clinic visit	684 (84%)	348 (43%)	6.6 (5.1-8.4)	<0.001	N/A
			Difference in mean scores (95% CI)		
Mean score of implementing PCC approach (out of 5)	3.9 (3.8-4.0)	1.6 (1.5-1.7)	2.3 (2.1-2.5)	<0.001	N/A
Mean score of PCC + appropriate FGM prevention and care (out of 8)	6.2 (5.9-6.6)	3.7 (3.2-4.1)	2.6 (2.0-3.2)	<0.001	N/A
**ANC clinic preparedness to offer FGM prevention and care services**					
	**Intervention (n=82)**	**Control (n=81)**	**Adjusted OR[&] (95% CI)**	**P-value**	**ICC**
Clinics with ALL correct responses for preparedness	56 (68%)	22 (27%)	-	<0.001	N/A
Mean score of clinic preparedness (out of 4)	3.4 (3.2-3.6)	2.6 (2.4-2.9)	-	<0.001	N/A
	**Intervention (n=115)**	**Control (n=117)**	**Adjusted OR[&] (95% CI)**	**P-value**	**ICC**
Providers using level 1 intervention package	96 (83%)	65 (56%)	9.3 (4.2-20.8)	<0.001	N/A
**Secondary Outcomes***					
Providers with appropriate interpersonal communication skills	74 (64%)	68 (58%)	1.7 (1.0-3.0)	0.060	N/A
Providers with high self-efficacy	86 (75%)	99 (85%)	0.8 (0.4-1.6)	0.453	N/A
Providers reporting less supportive attitudes towards FGM	76 (66%)	85 (73%)	1.0 (0.5-1.8)	0.901	N/A
Providers with high confidence scores	103 (90%)	104 (89%)	6.3 (1.4-28.9)	0.018	N/A
Providers not supportive of FGM	100 (87%)	114 (97%)	0.8 (0.2-3.7)	0.726	N/A
Providers not supportive of medicalized FGM	104 (90%)	116 (99%)	1.1 (0.1-22.1)	0.938	N/A
Providers with correct FGM-related knowledge responses	8 (8%)	1 (2%)	5.0 (0.5-47.8)	0.16	N/A
Mean score of FGM-related knowledge (out of 6)	2.5 (2.2-2.8)	1.9 (1.7-2.2)	-	0.005	N/A
**Other ANC client outcomes****					
	**Intervention (n=819)**	**Control (n=810)**	**Adjusted OR[&] (95% CI)**	**P-value**	**ICC**
Clients reporting less support for FGM after ANC clinic visit	424 (52%)	237 (29%)	5.4 (2.4-12.4)	<0.001	0.66
Clients reporting that they were strongly opposed to FGM	498 (61%)	382 (47%)	2.4 (1.1-5.2)	0.023	0.62

For peer review only

For peer review only - http://bmjopen.bmj.com/site/about/guidelines.xhtml

Clients reporting that they intend to have their daughters cut	96 (12%)	209 (26%)	0.3 (0.1-0.7)	0.004	0.60
Clients reporting that they would prefer health care provider to cut daughters	53 (7%)	139 (17%)	0.2 (0.1-0.5)	<0.001	0.54
Clients wishing to be active in FGM prevention	677 (83%)	535 (66%)	3.2 (1.6-6.2)	0.001	0.50

ICC = Intra-cluster Correlation Coefficient

[#]Single-level multi-variable adjusted models

[&]Multi-level multi-variable adjusted models

*Provider outcomes adjusted for sex, years of service, FGM status, FGM-related training, any specific training on communication/counseling and PCC, and whether the provider had conducted FGM in the past

** Client outcomes adjusted for age, educational level, FGM status and exposure to level one IEC materials

For peer review only

For peer review only - http://bmjopen.bmj.com/site/about/guidelines.xhtml

For peer review only

For peer review only - http://bmjopen.bmj.com/site/about/guidelines.xhtml

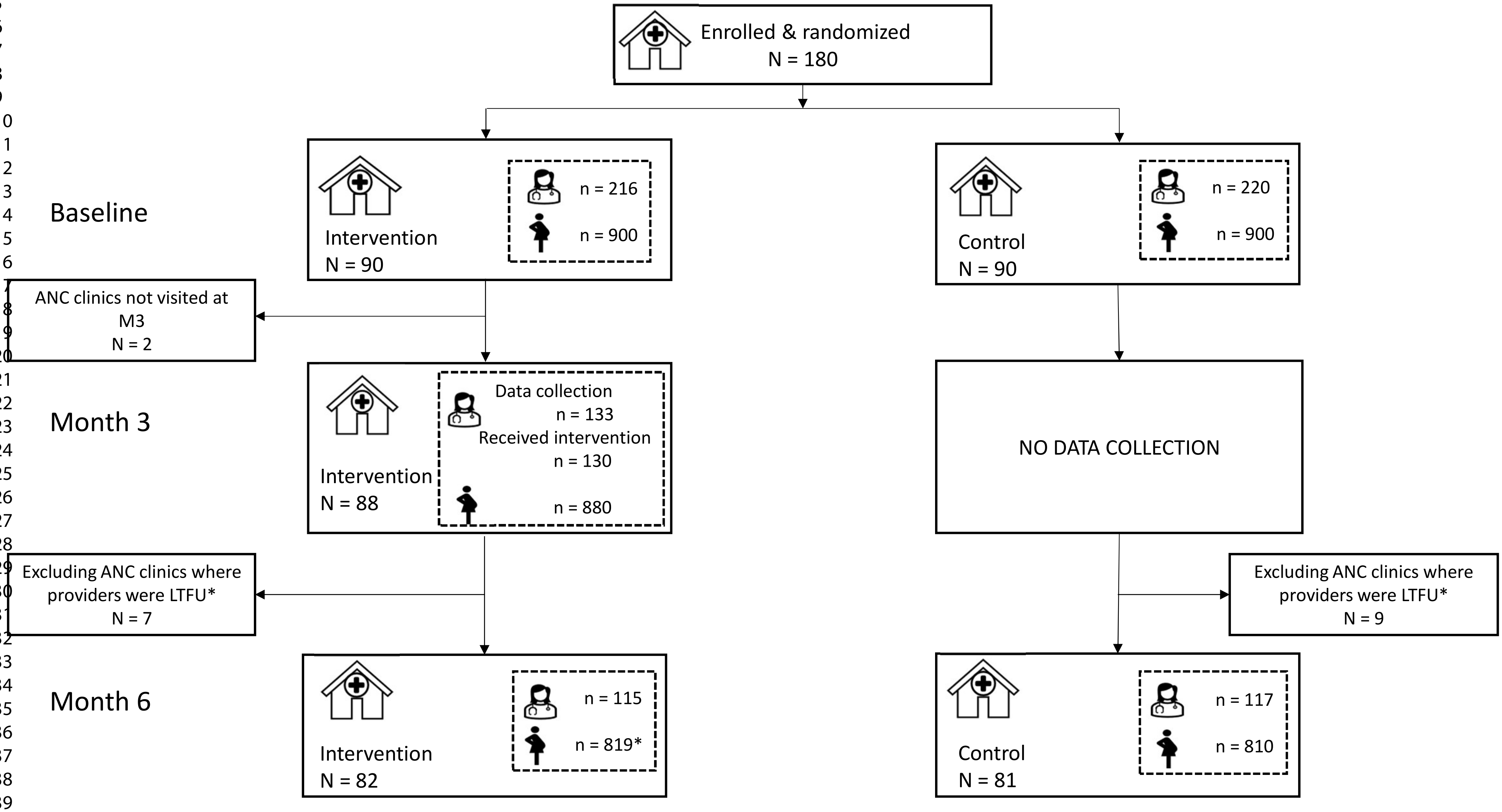
Enrolled & randomized
N = 180
Baseline
Intervention
N = 90
n = 216
n = 900
Control
N = 90
n = 220
n = 900
ANC clinics not visited at M3
N = 2
Month 3
Intervention
N = 88
Data collection
n = 133
Received intervention
n = 130
n = 880
NO DATA COLLECTION
Excluding ANC clinics where providers were LTFU*
N = 7
Excluding ANC clinics where providers were LTFU*
N = 9
Month 6
Intervention
N = 82
n = 115
n = 819*
Control
N = 81
n = 117
n = 810

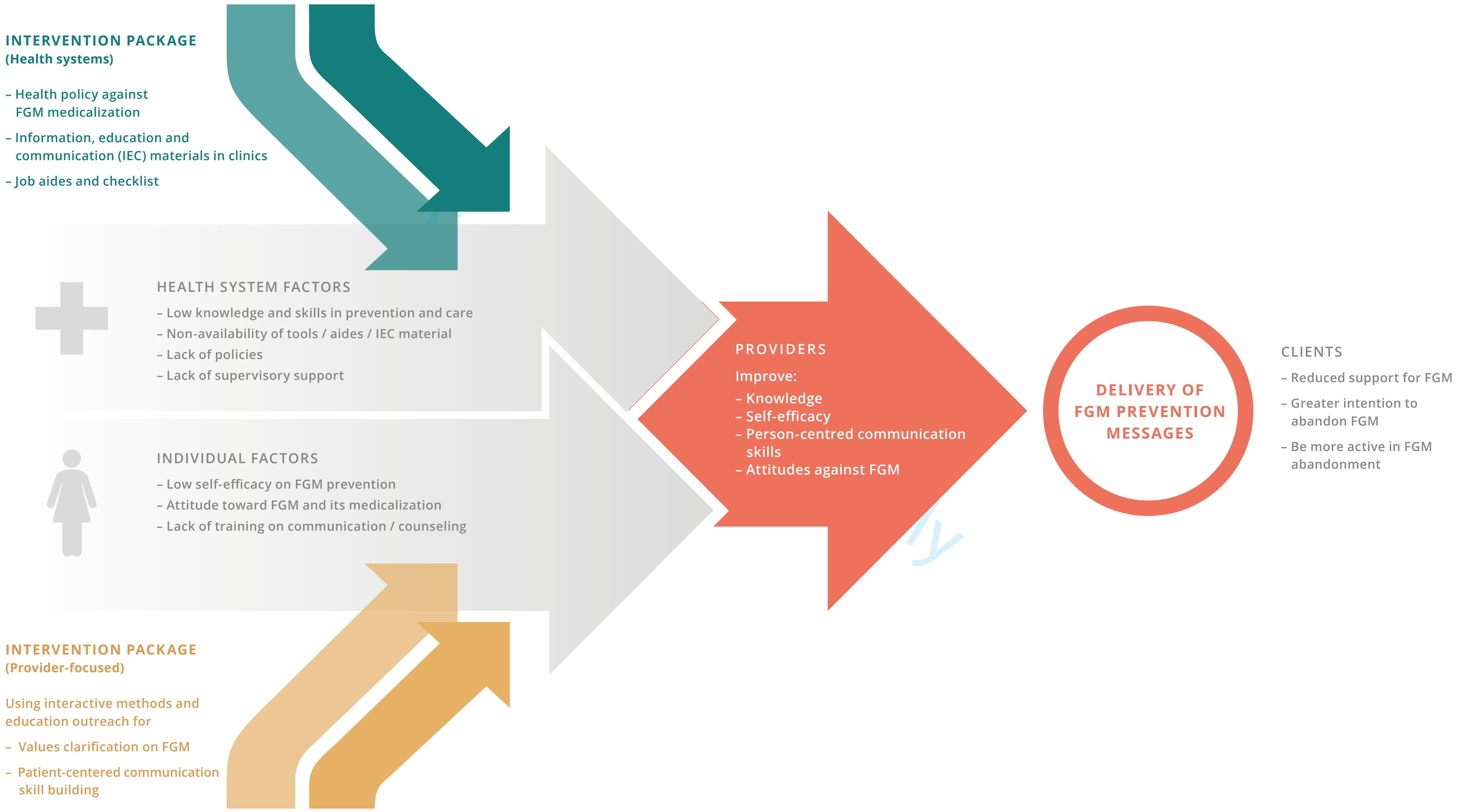
INTERVENTION PACKAGE
(Health systems)
– Health policy against FGM medicalization
– Information, education and communication (IEC) materials in clinics
– Job aides and checklist
HEALTH SYSTEM FACTORS
– Low knowledge and skills in prevention and care
– Non-availability of tools / aides / IEC material
– Lack of policies
– Lack of supervisory support
INDIVIDUAL FACTORS
– Low self-efficacy on FGM prevention
– Attitude toward FGM and its medicalization
– Lack of training on communication / counseling
INTERVENTION PACKAGE
(Provider-focused)
Using interactive methods and education outreach for
– Values clarification on FGM
– Patient-centered communication skill building
PROVIDERS
Improve:
– Knowledge
– Self-efficacy
– Person-centred communication skills
– Attitudes against FGM
DELIVERY OF FGM PREVENTION MESSAGES
CLIENTS
– Reduced support for FGM
– Greater intention to abandon FGM
– Be more active in FGM abandonment

Supplementary file 2 : Measurement of study outcomes

1. ***Primary Outcome:*** *Health facility preparedness to provide FGM prevention and care services.*

Outcome definition*: Cumulative score based on affirmative responses to Q9a, Q10a, Q11a & Q12a on the CHK form (see below).*

Q9. Is there an MoH policy on FGM posted on the wall?
Yes
No

Q9a. If yes, is it placed where health care providers can see/read it e.g., bulletin board?
Yes
No

Q10. Are there WHO FGM prevention posters on the wall of the consultation room and/or waiting room?
Yes
No

Q10a. If yes, are they placed in a place where ANC clients can see them?
Yes
No

Q11. Is there a WHO clinical handbook in the ANC consultation room?
Yes
No

Q11a If yes, is it placed where ANC providers can see/use it?
Yes
No

Q12. Is there an FGM ABCD guide in the ANC consultation room?
Yes
No

Q12a. If yes, is it placed where ANC providers can see/use it?
Yes
No

2. ***Primary outcome: ANC provider utilization of Level 1 package components***

Outcome definition*: Affirmative response on Q40 of HCP form (see below).*

Q40. Have you referred to the WHO Clinical Handbook on FGM?
Yes
No, available but not referred
No, not available
Don't know

3. ***Primary outcome: Provision of FGM-related care after PCC training***

Outcome definition: *Cumulative score based on affirmative responses (Provision of FGM-related care (after PCC training) either 'Always' or 'Often') on Q22, Q24 & Q25 on the HCP form (see below).*

Q22. How often do you discourage a pregnant woman expecting to have a girl, or one having a girl at the age of cutting, from having her daughter cut?
Always
Often
Sometimes
Rarely

For peer review only

Never
Rarely
Refused to answer

Q24. How often do you look for female genital mutilation when performing a gynecological examination of the vulva?

Always
Often
Sometimes
Rarely
Never
Rarely
Refused to answer

Q25. How often do you record female genital mutilation in the woman's medical file if you are aware that she has undergone FGM?

Always
Often
Sometimes
Rarely
Never
Rarely
Refused to answer

4. Primary Outcome: Delivery of PCC 'ABCD' package

Outcome definition: *Cumulative score based on affirmative responses on Q5, Q7, Q8, Q9 & Q12 on the EXT form.*

Q5. Did the ANC provider ask if you have undergone FGM?

Yes
No
Don't know
Refused

Q7. Did the ANC provider ask about your personal belief regarding FGM?

Yes
No
Don't know
Refused

Q8. Did the ANC provider discuss why FGM should be prevented?

Yes
No
Don't know
Refused

Q9. Did the ANC provider discuss how FGM could be prevented?

Yes
No
Don't know
Refused

Q12. Are you satisfied with how FGM was addressed during your visit with your ANC provider today?

Yes
No
Don't know
Refused

For peer review only

5. Secondary Outcome: Improved knowledge about FGM

Outcome definition: *Cumulative score based on correct responses to Q4 + affirmative responses to Q5 & Q7 of the HCP form.*

Q4. Please provide the WHO classification for the following images

Type I
Type II
Type III
Type IV
Don't Know
Other

Q5. Do you know of any health complications arising from female genital mutilation?

Yes
No

Q7. Are you aware of any existing WHO tools/guidance on FGM prevention and care?

Yes
No

6. Secondary Outcome: Improved interpersonal communication skills

Outcome definition: *Cumulative score based on positive responses ("Always or Often') to Q34, Q35, Q36, Q37, Q38 on the HCP form.*

Now I will ask you about your communication skills

34. I can put myself in others shoes

Always
Often
Sometimes
Rarely
Never
Rarely
Refused to answer

35. I let others know that I understand what they say

Always
Often
Sometimes
Rarely
Never
Rarely
Refused to answer

36. In conversations with my colleagues, I perceive not only what they say but what they don't say

Always
Often
Sometimes
Rarely
Never
Rarely
Refused to answer

37. I communicate effectively

Always
Often

For peer review only

Sometimes
Rarely
Never
Rarely
Refused to answer

38. I communicate with others as though they are my equals

Always
Often
Sometimes
Rarely
Never
Rarely
Refused to answer

7. *Secondary outcome: Improved self-efficacy*

Outcome definition: *Cumulative score based on positive responses (Agree or Strongly Agree) to Q26, Q27, Q28, Q29, Q30, Q31, Q32, Q33 on the HCP form.*

Now I would like to ask you a few questions about how you solve problems that you face. Please tell me how much you agree or disagree with the statements that I read to you

1 = Strongly disagree
2 = Disagree
3 = Neither agree nor disagree
4 = Agree
5 = Strongly agree

Q26. I will be able to achieve most of the goals that I have set for myself
Q27. When facing difficult tasks, I am certain that I will accomplish them
Q28. In general, I think that I can obtain outcomes that are important to me
Q29. I believe that I can succeed at almost any endeavor to which I set my mind
Q30. I will be able to successfully overcome many challenges
Q31. I am confident that I can perform effectively on many different tasks
Q32. Compared to other people, I can do most tasks very well
Q33. Even when things are tough, I can perform quite well

8. *Secondary outcome: Improved attitudes towards FGM*

Outcome definition*: Cumulative score based on positive responses to Q12, Q13, Q14, Q15, Q16, Q17, Q18 & Q19 on the HCP form.*

For each of the following statements please state if you:

1=Agree
2=Disagree
3=Don't know
4=Refused to answer

Q12. A girl who has not undergone FGM is unclean
Q13. A girl who has not undergone FGM cannot be married within her community
Q14. A girl who has not undergone FGM is a disgrace to her family's honor
Q15. Health care providers who provide FGM are violating FGM
Q16. Health care providers who provide FGM should be punished
Q17. FGM is a good practice
Q18. FGM is a violation of women and girls' rights
Q19. FGM is religious mandate

For peer review only

9. Tertiary outcome: ANC provider confidence in FGM knowledge to provide care

Outcome definition: *Positive responses ('Somewhat Confident' or 'Confident') to Q8 & Q9 on the HCP form*

Q8. When you treat or attend to a girl or woman with female genital mutilation, how confident are you that you have enough knowledge to provide good quality care?

1=Not confident
2=Somewhat confident
3=Confident
4=Refused to answer

Q9. How confident are you in your knowledge to communicate on FGM prevention?

1=Not confident
2=Somewhat confident
3=Confident
4=Refused to answer

10. Tertiary outcome: ANC provider support for FGM

Outcome definition: *Positive response ('Do not intend to cut her') to Q20 on the HCP form*

Q20. Pretend you had a daughter now who was at an age when cutting occurs, what would your intention to cut her be?

1=Intend to cut her
2=Do not intend to cut her
3=Don't know
4=Refused to answer

11. Tertiary outcome: ANC provider support for medicalized FGM

Outcome definition: *Correct response ('No') to Q21 on HCP form*

Q21. If a family brought their daughter to the clinic requesting genital cutting, for non-health reasons, would you perform it?

1=Yes
2=No
3=Don't know
4=Refused to answer

12. Tertiary outcome: ANC client change in support for FGM after ANC visit

Outcome definition: *Response to Q13 on EXT form*

Q13. What do you feel about FGM now as compared to before you came to the clinic today?

1= Same, no change
2=I feel more supportive of FGM now as compared to before I came
3=I feel less supportive of FGM now as compared to before I came
4=Don't know
5=Other
6=Refused to answer

13. Tertiary outcome: ANC client support or opposition to FGM

Outcome definition: *Response to Q14 on EXT form*

Q14. How supportive are you of female genital mutilation?

1=Strongly opposed
2=Somewhat opposed

For peer review only

3=Neutral
4=Somewhat supportive
5=Strongly supportive
6=Refused to answer

14. Tertiary outcome: ANC client intention to cut after ANC visit.
Outcome definition: *Response to Q16 on EXT form*
Q.16 Pretend you had a daughter now who was at an age where cutting occurs, what would your intention to cut her be?
1=Intend to cut her
2=Do not intend to cut her
3=Don't know
4=Refused to answer

15. Tertiary outcome: ANC client choice of who to cut their daughters.
Outcome definition: *Response to Q17 on EXT form*
Q17. If intending to cut, who would you prefer to do the cutting?
1=Traditional practitioner
2=Health care provider
3=Other
4=Refused to answer

16. Tertiary outcome: ANC client wish to be active in FGM prevention
Outcome definition: *Response to Q18 on EXT form*
Q.18 Do you wish/want to be active in preventing FGM?
1=Yes
2=No
3=Don't know
4=Refused to answer

For peer review only

A HEALTH SYSTEMS APPROACH TO PREVENTION OF FEMALE GENITAL MUTILATION USING PERSON-CENTRED COMMUNICATION: IMPLEMENTATION RESEARCH PROJECT IN GUINEA, SOMALIA AND KENYA (A65993)

ANC PROVIDER SCREENING QUESTIONNAIRE (SCR)

Participant ID:

A	6	5	9	9	3

Project ID:

Country ID: Facility ID:

Instructions: Observe and report findings from the health facility.

1. **MoH policy on FGM posted on the wall?**
☐ Yes
☐ No
1a. If yes, is it placed where health care providers can see/read it e.g. bulletin board?
☐ Yes
☐ No

2. **Are there FGM prevention posters on the wall of the waiting room?** ☐ Yes
☐ No
2a. If yes, is it placed in place where ANC clients can see it
☐ Yes
☐ No

3. **Is there WHO FGM Clinical Handbook in the ANC consultation room?** ☐ Yes
☐ No
3a. If yes, is it placed where ANC provider can see /use it?
☐ Yes
☐ No

4. **Is there FGM ABCD guide in ANC consultation room?**
☐ Yes
☐ No
4a. If yes, is it placed where ANC provider can see /use it
☐ Yes ☐
No

Instructions: Assess health facility factors that may facilitate/constrain intervention delivery by reviewing health facility administrative records and notes and by meeting with the health facility manager.

5. **Number of ANC providers** __________________
6. **Average number of ANC clients per month**__________________
7. **Number of ANC providers trained on PCC on FGM prevention**
☐ All (specify number trained): _________
☐ Some (specify number trained): _________
☐ None
8. **Indicate the number of MoH supervisory visits to the clinic in the past year**__________________

For peer review only

A HEALTH SYSTEMS APPROACH TO PREVENTION OF FEMALE GENITAL MUTILATION USING PERSON-CENTRED COMMUNICATION: IMPLEMENTATION RESEARCH PROJECT IN GUINEA, SOMALIA AND KENYA (A65993)

ANC PROVIDER SCREENING QUESTIONNAIRE (SCR)

Participant ID: | | | | |

A	6	5	9	9	3

Project ID: | | | | |

Country ID: Facility ID: | | | | | |

Version 2 – 18th October 2019 1

9. How frequently are staff meetings held?

☐ Monthly

☐ Every 2 to 4 months

☐ Every 6 to 12months

☐ Never

10. What is the size of the population served by this facility? (*specify number)* ________________

11. Are there country/region-specific FGM laws that are enforced?

☐ Yes

☐ No

12. Are there anti-FGM activities that target the population served by this health facility?

☐ Yes

☐ No

13. Are there pro-FGM activities that target the population served by this health facility?

☐ Yes

☐ No

Additional comments:

____

____

____

____

For peer review only

A HEALTH SYSTEMS APPROACH TO PREVENTION OF FEMALE GENITAL MUTILATION USING PERSON-CENTRED COMMUNICATION: IMPLEMENTATION RESEARCH PROJECT IN GUINEA, SOMALIA AND KENYA (A65993)

ANC PROVIDER SCREENING QUESTIONNAIRE (SCR)

Participant ID: | | | | |

A	6	5	9	9	3

Project ID: | | | | |

Country ID: | | | | | |

Facility ID:

Version 2 – 18th October 2019 2

1. **What is your age? ______________**
2. **What is your sex?**
   1. ☐ Female
   2. ☐ Male
3. **What is your religion?**
   1. ☐ Muslim
   2. ☐ Christian
   3. ☐ Other
   4. ☐ None
   5. ☐ Refused
4. **What is your occupation/designation?**
   1. ☐ Midwife
   2. ☐ Nurse
   3. ☐ Other, specify ____
5. **What is the highest education level of education you achieved?**
   1. ☐ Certificate
   2. ☐ Diploma
   3. ☐ Bachelors
   4. ☐ Masters or above
   5. ☐ Other, specify ____
6. **For how many years have you been working in your field? ________**
7. **During you clinical training, did you receive any formal training on female genital mutilation?**
   1. ☐ Yes.
   2. ☐ No. Go to section B
   3. ☐ I don't know. Go to section B
8. **When did you receive the training?**
   1. ☐ During my studies (pre-service training)
   2. ☐ After graduation/at work (in-service training)
   3. ☐ Both
   4. ☐ I don't know

   7. ☐ Not applicable

For peer review only - http://bmjopen.bmj.com/site/about/guidelines.xhtml

A HEALTH SYSTEMS APPROACH TO PREVENTION OF FEMALE GENITAL MUTILATION USING PERSON-CENTRED COMMUNICATION: IMPLEMENTATION RESEARCH PROJECT IN GUINEA, SOMALIA AND KENYA (A65993)

ANC PROVIDER SCREENING QUESTIONNAIRE (SCR)

Participant ID:

Project ID:

Country ID: Facility ID:

To be completed by data collector:

Data Collector ID:

Signature:

Date:

Day		Month			Year			
					2	0		

For peer review only

For peer review only

A HEALTH SYSTEMS APPROACH TO PREVENTION OF FEMALE GENITAL MUTILATION USING PERSON-CENTRED COMMUNICATION: IMPLEMENTATION RESEARCH PROJECT IN GUINEA, SOMALIA AND KENYA (A65993)

ANC PROVIDER SCREENING QUESTIONNAIRE (SCR)

Participant ID:

Project ID: Country ID: Facility ID:

A	6	5	9	9	3

9. What was the format of the training? (Check all that apply)

1. ☐ Classroom lessons
2. ☐ Workshops
3. ☐ Digital format (E-learning videos; smart phone app)
4. ☐ During clinical practice under supervision of a mentor
5. ☐ Other, specify ____

7. ☐ Not applicable

10. During your pre- or post- graduate training, did you receive any formal training on communication or counselling?

1. ☐ Yes.
2. ☐ No.
3. ☐ I don't know

11. During you pre or post graduate training, did you receive any formal training on person-centred care?

1. ☐ Yes.
2. ☐ No.
3. ☐ I don't know

12. Have you ever cut the genitals of a girl (<=18 years old) for non-health reasons?

1. ☐ Yes.
2. ☐ No.
3. ☐ I don't know

For peer review only - http://bmjopen.bmj.com/site/about/guidelines.xhtml

A HEALTH SYSTEMS APPROACH TO PREVENTION OF FEMALE GENITAL MUTILATION USING PERSON-CENTRED COMMUNICATION: IMPLEMENTATION RESEARCH PROJECT IN GUINEA, SOMALIA AND KENYA (A65993)

ANC PROVIDER QUESTIONNAIRE (HCP)

Participant ID:

Project ID: Country ID: Facility ID:

A	6	5	9	9	3

To be completed by data collector:

Data Collector ID: *Date:*

Signature:

Day		Month			Year			
					2	0		

1. **Have you ever heard about female genital mutilation?**
   ☐ Yes
   ☐ No
2. **Do the women in your community undergo female genital mutilation?**
   ☐ Yes
   ☐ No
   ☐ I don't know
3. **Do you know of the WHO classification for female genital mutilation?**
   ☐ Yes
   ☐ No. Skip to Q5
4. **Please provide the WHO classification for the following FGM images (to include images)**
   a. **IMAGE of Type III FGM to be inserted here**
      i. ☐ **Type I**
      ii. ☐ **Type II**
      iii. ☐ **Type III**
      iv. ☐ **Type IV**
      v. ☐n**Don't know**
   b. **IMAGE of Type I FGM to be inserted here**
      i. ☐ **Type I**
      ii. ☐ **Type II**
      iii. ☐ **Type III**

Version 2 – 6th November 2019 1

To be completed by data collector:

Data Collector ID: *Date:*

Signature:

Day		Month			Year			
					2	0		

For peer review only - http://bmjopen.bmj.com/site/about/guidelines.xhtml

A HEALTH SYSTEMS APPROACH TO PREVENTION OF FEMALE GENITAL MUTILATION USING PERSON-CENTRED COMMUNICATION: IMPLEMENTATION RESEARCH PROJECT IN GUINEA, SOMALIA AND KENYA (A65993)

ANC PROVIDER QUESTIONNAIRE (HCP)

Participant ID:

Project ID: Country ID: Facility ID:

iv. ☐ **Type IV**

v. ☐n**Don't know**

c. **IMAGE of Type II FGM to be inserted here**

i. ☐ **Type I ii.** ☐ **Type II**

A 6 5 9 9 3

iii. ☐ **Type III**

iv. ☐ **Type IV**

v. ☐n**Don't know**

d. **IMAGE of Type III FGM to be inserted here**

i. ☐ **Type I**

ii. ☐ **Type II**

iii. ☐ **Type III**

iv. ☐ **Type IV**

v. ☐n**Don't know**

e.

5. **Do you know of any health complications arising from female genital mutilation?**

☐ Yes

☐ No. Skip to Q6

6. **Is female genital mutilation illegal in your country (specify actual study country)?**

1. ☐ Yes
2. ☐ No
3. ☐ I don't know

7. **Are you aware of any existing WHO tools/guidance on female genital mutilation and its complications?**

1. ☐ Yes. *If yes, please specify*…………………………………………………….
2. ☐ No

To be completed by data collector:

Data Collector ID:	*Date:*		
Signature:	Day	Month	Year
			2 0

For peer review only - http://bmjopen.bmj.com/site/about/guidelines.xhtml

A HEALTH SYSTEMS APPROACH TO PREVENTION OF FEMALE GENITAL MUTILATION USING PERSON-CENTRED COMMUNICATION: IMPLEMENTATION RESEARCH PROJECT IN GUINEA, SOMALIA AND KENYA (A65993)

ANC PROVIDER QUESTIONNAIRE (HCP)

Participant ID: | | | | |

Project ID: | | | | | | | Country ID: | | | | Facility ID: | | | | | |

A 6 5 9 9 3

8. When you treat or attend a girl or a woman with genital mutilation, how confident are you that you have enough knowledge to provide good quality health care? *Rate between 1 – 4*

1. ☐ Not confident at all
2. ☐ Not very confident
3. ☐ Fairly confident
4. ☐ Fully confident

9. How confident are you in your FGM knowledge to communicate on FGM prevention?
Rate between 1 – 4

1. ☐ Not confident at all
2. ☐ Not very confident
3. ☐ Fairly confident
4. ☐ Fully confident

For each of the following statements please state if you agree/disagree or don't know.

10. A girl who has not undergone FGM is unclean.

1. ☐ Agree
2. ☐ Disagree
3. ☐ I don't know

11. A girl without FGM cannot be married within her community.

1. ☐ Agree
2. ☐ Disagree
3. ☐ I don't know

12. A girl who has not undergone FGM is a disgrace to her family's honour.

1. ☐ Agree
2. ☐ Disagree

To be completed by data collector:

Data Collector ID: *Date:*

Signature:

Day		Month			Year			
					2	0		

For peer review only

A HEALTH SYSTEMS APPROACH TO PREVENTION OF FEMALE GENITAL MUTILATION USING PERSON-CENTRED COMMUNICATION: IMPLEMENTATION RESEARCH PROJECT IN GUINEA, SOMALIA AND KENYA (A65993)

ANC PROVIDER QUESTIONNAIRE (HCP)

Participant ID: | | | |

| A | 6 | 5 | 9 | 9 | 3 |

Project ID: | | | |

Country ID: Facility ID: | | | | |

3. ☐ I don't know

13. Health care providers who perform FGM are violating medical ethics.

1. ☐ Agree
2. ☐ Disagree
3. ☐ I don't know

14. Health care providers who perform FGM should be punished.

1. ☐ Agree
2. ☐ Disagree
3. ☐ I don't know

15. FGM is a good practice

1. ☐ Agree
2. ☐ Disagree
3. ☐ I don't know

16. FGM is a violation of women's and girls' rights

1. ☐ Agree
2. ☐ Disagree
3. ☐ I don't know

17. FGM is a religious mandate

1. ☐ Agree
2. ☐ Disagree
3. ☐ I don't know

18. Pretend you had a daughter now who was at an age when cutting occurs, what would your intention to cut her be? 1. ☐ Intend to cut her

2. ☐ Do not intend to cut her
3. ☐ Undecided
4. Refused to answer

19. If a family brought their daughter to the clinic requesting genital cutting for non-health reasons, would you perform it?

To be completed by data collector:

Data Collector ID:	*Date:*								
Signature:	Day		Month			Year			
						2	0		

For peer review only - http://bmjopen.bmj.com/site/about/guidelines.xhtml
For peer review only

A HEALTH SYSTEMS APPROACH TO PREVENTION OF FEMALE GENITAL MUTILATION USING PERSON-CENTRED COMMUNICATION: IMPLEMENTATION RESEARCH PROJECT IN GUINEA, SOMALIA AND KENYA (A65993)

ANC PROVIDER QUESTIONNAIRE (HCP)

Participant ID:

Project ID: Country ID: Facility ID:

1. ☐ Yes
2. ☐ No
3. ☐ I don't know

Now I would like to ask you a few questions about how you solve problems that you face. Please state how much you agree or disagree with the statements that I read, , where 1=Strongly disagree; 2=Disagree; 3=Neither agree nor disagree; 4=Agree; 5=Strongly agree

20. I will be able to achieve most of the goals that I have set for myself.

1. ☐ **Strongly disagree**
2. ☐ **Disagree**
3. ☐ **Neither agree nor disagree**
4. ☐ **Agree**

A 6 5 9 9 3

5. ☐ **Strongly agree**
6. ☐ **Don't know**

21. When facing difficult tasks, I am certain that I will accomplish them.

1. ☐ **Strongly disagree**
2. ☐ **Disagree**
3. ☐ **Neither agree nor disagree**
4. ☐ **Agree**
5. ☐ **Strongly agree**
6. ☐ **Don't know**

22. In general, I think that I can obtain outcomes that are important to me.

1. ☐ **Strongly disagree**
2. ☐ **Disagree**
3. ☐ **Neither agree nor disagree**

To be completed by data collector:

Data Collector ID:	*Date:*		
Signature:	Day	Month	Year
			2 0

For peer review only - http://bmjopen.bmj.com/site/about/guidelines.xhtml

A HEALTH SYSTEMS APPROACH TO PREVENTION OF FEMALE GENITAL MUTILATION USING PERSON-CENTRED COMMUNICATION: IMPLEMENTATION RESEARCH PROJECT IN GUINEA, SOMALIA AND KENYA (A65993)

ANC PROVIDER QUESTIONNAIRE (HCP)

Participant ID:

Project ID: Country ID: Facility ID:

4. ☐ **Agree**
5. ☐ **Strongly agree**
6. ☐ **Don't know**

23. I believe I can succeed at most any endeavour to which I set my mind.

1. ☐ **Strongly disagree**
2. ☐ **Disagree**
3. ☐ **Neither agree nor disagree**
4. ☐ **Agree**
5. ☐ **Strongly agree**
6. ☐ **Don't know**

24. I will be able to successfully overcome many challenges.

1. ☐ **Strongly disagree**
2. ☐ **Disagree**
3. ☐ **Neither agree nor disagree**
4. ☐ **Agree**
5. ☐ **Strongly agree**

A 6 5 9 9 3

6. ☐ **Don't know**

25. I am confident that I can perform effectively on many different tasks.

1. ☐ **Strongly disagree**
2. ☐ **Disagree**
3. ☐ **Neither agree nor disagree**
4. ☐ **Agree**
5. ☐ **Strongly agree**
6. ☐ **Don't know**

To be completed by data collector:

Data Collector ID: *Date:*

Signature:

Day		Month			Year			
					2	0		

For peer review only

A HEALTH SYSTEMS APPROACH TO PREVENTION OF FEMALE GENITAL MUTILATION USING PERSON-CENTRED COMMUNICATION: IMPLEMENTATION RESEARCH PROJECT IN GUINEA, SOMALIA AND KENYA (A65993)

ANC PROVIDER QUESTIONNAIRE (HCP)

Participant ID:

Project ID: Country ID: Facility ID:

26. Compared to other people, I can do most tasks very well.

1. ☐ **Strongly disagree**
2. ☐ **Disagree**
3. ☐ **Neither agree nor disagree**
4. ☐ **Agree**
5. ☐ **Strongly agree**
6. ☐ **Don't know**

For peer review only

27. Even when things are tough, I can perform quite well.

1. ☐ **Strongly disagree**
2. ☐ **Disagree**
3. ☐ **Neither agree nor disagree**
4. ☐ **Agree**
5. ☐ **Strongly agree**
6. ☐ **Don't know**

A 6 5 9 9 3

28. Would you like to receive more training related to care for women and girls with FGM?

1. ☐ Yes
2. ☐ No
3. ☐ I don't know

29. If a pregnant woman is expected to have a girl, do you discourage her from having her daughter cut?

To be completed by data collector:

Data Collector ID: *Date:*

Signature:

Day		Month			Year			
					2	0		

A HEALTH SYSTEMS APPROACH TO PREVENTION OF FEMALE GENITAL MUTILATION USING PERSON-CENTRED COMMUNICATION: IMPLEMENTATION RESEARCH PROJECT IN GUINEA, SOMALIA AND KENYA (A65993)

ANC PROVIDER QUESTIONNAIRE (HCP)

Participant ID:

Project ID: Country ID: Facility ID:

1. ☐ Always
2. ☐ Often
3. ☐ Sometimes
4. ☐ Rarely
5. ☐ Never

30. If you heard of or saw a colleague performing female genital mutilation, what would you do? ***(Tick all that apply)***

1. ☐ I would report him/her to the authorities
2. ☐ I would discuss with him/her and explain to him/her that health care providers should not perform female genital mutilation
3. ☐ I would not get involved 4. ☐ I don't know

31. How often do you look for female genital cutting/excision when performing a gynecological examination of the vulva?

1. ☐ Always
2. ☐ Often
3. ☐ Sometimes
4. ☐ Rarely
5. ☐ Never

32. How often do you record the female genital mutilation in the women's medical file if you are aware that she has undergone FGM?

1. ☐ Always
2. ☐ Often
3. ☐ Sometimes
4. ☐ Rarely
5. ☐ Never

33. Would you like to receive more training on how to help patients to prevent FGM?

1. ☐ Yes
2. ☐ No
3. ☐ I don't know

A 6 5 9 9 3

34. I can put myself in others' shoes

1. ☐ Always
2. ☐ Often

To be completed by data collector:

Data Collector ID: *Date:*

Signature:

Day		Month			Year			
					2	0		

For peer review only

A HEALTH SYSTEMS APPROACH TO PREVENTION OF FEMALE GENITAL MUTILATION USING PERSON-CENTRED COMMUNICATION: IMPLEMENTATION RESEARCH PROJECT IN GUINEA, SOMALIA AND KENYA (A65993)

ANC PROVIDER QUESTIONNAIRE (HCP)

Participant ID:

Project ID: | Country ID: | Facility ID:

3. ☐ Sometimes
4. ☐ Rarely
5. ☐ Never

35. I let others know I understand what they say

1. ☐ Always
2. ☐ Often
3. ☐ Sometimes
4. ☐ Rarely
5. ☐ Never

36. In conversations with my colleagues, I perceive not only what they say but what they don't say

1. ☐ Always
2. ☐ Often
3. ☐ Sometimes
4. ☐ Rarely
5. ☐ Never

37. I communicate effectively

1. ☐ Always
2. ☐ Often
3. ☐ Sometimes
4. ☐ Rarely
5. ☐ Never

38. I communicate with others as though they are my equals

1. ☐ Always
2. ☐ Often
3. ☐ Sometimes
4. ☐ Rarely
5. ☐ Never

A 6 5 9 9 3

To be completed by data collector:

Data Collector ID: *Date:*

Signature:

Day		Month			Year			
					2	0		

For peer review only - http://bmjopen.bmj.com/site/about/guidelines.xhtml

For peer review only

A HEALTH SYSTEMS APPROACH TO PREVENTION OF FEMALE GENITAL MUTILATION USING PERSON-CENTRED COMMUNICATION: IMPLEMENTATION RESEARCH PROJECT IN GUINEA, SOMALIA AND KENYA (A65993)

ANC PROVIDER QUESTIONNAIRE (HCP)

Participant ID: | | | | |

| | | | | | | Project ID: | | | | | Country ID: | | | Facility ID: | | |

These next questions relate to your clinic setting:

39. Have you seen the posters on FGM at the clinic?

1. ☐ Yes
2. ☐ No
3. ☐ I don't know

40. Have you referred to the clinical handbook on FGM that is available in your clinic?

1. ☐ No
2. ☐ I don't know

41. Do you think it is feasible to provide FGM prevention counselling during ANC visits?

1. ☐ Yes
2. ☐ No
3. ☐ I don't know

Comments

-

____

____

____

____

____

To be completed by data collector:

Data Collector ID: *Date:*

Signature:

Day		Month			Year			
					2	0		

For peer review only - http://bmjopen.bmj.com/site/about/guidelines.xhtml

For peer review only

A HEALTH SYSTEMS APPROACH TO PREVENTION OF FEMALE GENITAL MUTILATION USING PERSON-CENTRED COMMUNICATION: IMPLEMENTATION RESEARCH PROJECT IN GUINEA, SOMALIA AND KENYA (A65993)

Participant ID:

Project ID: Facility ID:

FIRST ANC CLIENT EXIT QUESTIONNAIRE (EXT)

Country ID:

A 6 5 9 9 3

For peer review only

1. **How old are you? (years)**____
2. **What is your religion?**
    1. ☐ Muslim
    2. ☐ Christian
    3. ☐ Other
    4. ☐ None
    5. ☐ Refused
3. **What is the highest level of education you achieved?**
    1. ☐ None
    2. ☐ Primary
    3. ☐ Secondary
    4. ☐ University
    5. ☐ Other, *specify* ____
4. **Many women in your community have had their genitals cut when they were children, if you are comfortable telling me, can I ask if you have undergone this practice?**
    1. ☐ Yes
    2. ☐ No
    3. ☐ I don't know
    4. ☐ Refused
5. **How supportive are you of female genital mutilation?**
    1. ☐ Strongly opposed
    2. ☐ Somewhat opposed
    3. ☐ Neutral (Neither opposed or supportive)
    4. ☐ Somewhat supportive
    5. ☐ Strongly supportive

The following questions relate to your visit today. During your visit today:

6. **Did you see any FGM poster(s) in the waiting room?**
    1. ☐ Yes
    2. ☐ No
    3. ☐ I don't know

A HEALTH SYSTEMS APPROACH TO PREVENTION OF FEMALE GENITAL MUTILATION USING PERSON-CENTRED COMMUNICATION: IMPLEMENTATION RESEARCH PROJECT IN GUINEA, SOMALIA AND KENYA (A65993)

Participant ID:

Project ID: Facility ID:

7. Did the ANC provider ask if you have undergone FGM?

1. ☐ Yes
2. ☐ No
3. ☐ I don't know

8. Did the ANC provider explain how FGM can harm your health?

1. ☐ Yes
2. ☐ No

Version 2 – 6th November 2019 1 **FIRST ANC CLIENT EXIT QUESTIONNAIRE (EXT)**

Country ID:

A 6 5 9 9 3

3. ☐ I don't know

9. Did the ANC provider ask about your personal belief regarding FGM?

1. ☐ Yes
2. ☐ No
3. ☐ I don't know

10. Did the ANC provider discuss why FGM should be prevented?

1. ☐ Yes
2. ☐ No
3. ☐ I don't know

11. Did the ANC provider discuss how FGM could be prevented?

1. ☐ Yes
2. ☐ No
3. ☐ I don't know

12. Did you have questions about FGM to ask the ANC provider?

1. ☐ Yes
2. ☐ No
3. ☐ I don't know

13. Did you feel encouraged to ask questions about FGM?

1. ☐ Yes
2. ☐ No
3. ☐ I don't know

14. Are you satisfied with how FGM was addressed during your visit with your ANC provider today?

For peer review only

A HEALTH SYSTEMS APPROACH TO PREVENTION OF FEMALE GENITAL MUTILATION USING PERSON-CENTRED COMMUNICATION: IMPLEMENTATION RESEARCH PROJECT IN GUINEA, SOMALIA AND KENYA (A65993)

Participant ID:

Project ID: Facility ID:

1. ☐ Yes
2. ☐ No
3. ☐ I don't know

15. What do you feel about FGM now as compared to before you came to the clinic today?

1. ☐ Same, no change
2. ☐ I feel more supportive of FGM now as compared to before I came
3. ☐ I feel less supportive of FGM now as compared to before I came
4. ☐ I do not know
5. ☐ Other, *specify* ____

16. Pretend you had a daughter now who was at an age when cutting occurs, what would your intention to cut her be?

1. ☐ Intend to cut her
2. ☐ Do not intend to cut her

17. Do you wish/want to be active in preventing FGM?

1. ☐ Yes
2. ☐ No
3. ☐ I don't know

For peer review only

Supplementary file 3: Additional analyses (appendices 1 – 3)

Appendix 1: Comparison baseline characteristics of ANC facilities

Characteristics	Facilities included in final analysis (n=163)	Facilities excluded* from final analysis (n=17)
Number of ANC providers	Mean 4 (SD: 3) Median 3 (1-14, IQR 3)	Mean 3 (SD: 3) Median 2 (1-9, IQR 1)
Average number of ANC clients/month	Mean 150 (SD: 127) Median 118 (3-664, IQR 141)	Mean 162 (SD: 147) Median 100 (25-600, IQR 200)
MoH supervisory visits in the past year	Mean 4 (SD: 3) Median 3 (0-18, IQR 2)	Mean 5 (SD: 4) Median 4 (0-12, IQR 4)
Size of catchment population served	Mean 36,754 (SD: 126,082) Median 15,972 (1,000-1,458,000, IQR 24,332)	Mean 11,737 (SD: 14,62) Median 7,800 (1,200-63,000, IQR 7,505
Presence of anti-FGM activities in the catchment area		
Yes	74 (45%)	9 (53%)
No	89 (55%)	8 (47%)
Presence of pro-FGM activities in the catchment area		
Yes	21 (13%)	2 (12%)
No	140 (86%)	15 (88%)
Don't Know	2 (1%)	-

*Total of ANC clinics not included: 16 clinics were excluded (7 intervention and 9 control) due to loss-to-follow up (LTFU) of ANC provider i.e., the clinics did not have at least one ANC provider present across all study time points while one ANC clinic in Kenya was never visited at subsequent time points due to issues with insecurity. An ANC provider from one of the clinics in Kenya that had been inaccessible due to insecurity attended the PCC training and was subsequently interviewed.

For peer review only

Appendix 2: Comparison of baseline characteristics of ANC providers

**Characteristics**	**Providers recruited at Baseline (n=436)**	**Providers enrolled with complete data at Month 6 (n=232)**	**Providers not enrolled with no data at Month 6 (n=204)**
Age	37 (20-65; SD: 10)	36 (20-65; SD: 10)	38 (21-62; SD: 10)
Years of professional experience	9 (1-39; SD: 7)	8 (1-39; SD: 7)	10 (1-36; SD: 8)
Sex			
Female	361 (83%)	193 (83%)	168 (82%)
Male	75 (17%)	39 (17%)	36 (18%)
Highest educational level			
Certificate	44 (3%)	21 (5%)	23 (11%)
Diploma	309 (71%)	158 (68%)	151 (74%)
Bachelors	64 (15%)	44 (19%)	20 (10%)
Masters & above	3 (0.7%)	1 (0.4%)	2 (1%)
Other[#]	16 (4%)	8 (3%)	8 (4%)
Current professional role/title			
Midwife	198 (45%)	103 (44%)	95 (47%)
Nurse	95 (22%)	51 (22%)	44 (22%)
Nurse-Midwife	94 (22%)	54 (23%)	40 (20%)
Other	49 (11%)	24 (10%)	25 (12%)
Received formal training on FGM during clinical training			
Yes	158 (36%)	85 (37%)	73 (36%)
No	275 (63%)	146 (63%)	129 (63%)
Don't Know	3 (0.7%)	1 (0.4%)	2 (1%)
Timing of clinical training on FGM			
Pre-service	63 (14%)	33 (14%)	30 (15%)
In-service	81 (19%)	45 (19%)	36 (18%)
Both pre- and in-service	14 (3%)	7 (3%)	7 (3%)
Received formal training on communication/counselling			
Yes	287 (66%)	149 (64%)	138 (68%)
No	149 (34%)	83 (36%)	66 (32%)
Received formal training on person-centered care			
Yes	227 (52%)	118 (51%)	109 (53%)
No	207 (47%)	131 (56%)	94 (46%)
Don't know	2 (0.5%)	1 (0.4%)	1 (0.5%)

For peer review only

Characteristics	Providers recruited at Baseline (n=436)	Providers enrolled with complete data at Month 6 (n=232)	Providers not enrolled with no data at Month 6 (n=204)
Undergone FGM			
Yes	226 (52%)	126 (54%)	100 (49%)
No	128 (29%)	63 (27%)	65 (32%)
Don't know	4 (0.9%)	2 (1%)	1 (0.5%)
Refused to answer	3 (0.7%)	2 (1%)	1 (0.55)
Conducted FGM			
Yes	35 (8%)	15 (7%)	20 (10%)
Conducted FGM on a girl <18 years			
Yes	32 (7%)	14 (6%)	18 (9%)

For peer review only

Appendix 3: Comparison of study outcomes between baseline vs. month 3 and month 3 vs. month 6 in the intervention arm

	Baseline (Intervention only)	Month 3 (Intervention only)	P-value	Month 3 (Intervention only)	Month 6 (Intervention only)	P-value
**Primary Outcomes**						
**ANC clients reporting that their provider implemented components of PCC for FGM prevention**						
Provider asked client if they have undergone FGM	48 (6%)	298 (37%)	<0.0001	298 (37%)	694 (78%)	<0.0001
Provider asked client about their (client's) personal beliefs regarding FGM	38 (5%)	239 (29%)	<0.0001	239 (29%)	616 (76%)	<0.0001
Provider discussed with client why FGM should be prevented	56 (7%)	243 (30%)	<0.0001	243 (30%)	629 (77%)	<0.0001
Provider discussed with client how FGM could be prevented	48 (6%)	224 (28%)	<0.0001	224 (28%)	592 (73%)	<0.0001
Client satisfied with how FGM was addressed by provider during clinic visit	176 (21%)	346 (43%)	<0.0001	346 (43%)	684 (84%)	<0.0001
Mean score of PCC approach (out of 5)	0.5 (0.4-0.5)	1.7 (1.5-1.8)	<0.0001	1.7 (1.5-1.8)	3.9 (3.8-4.0)	<0.0001
Mean score of PCC + appropriate FGM prevention & care (out of 8)	1.8 (1.6-2.1)	3.3 (2.8-3.8)	<0.0001	3.3 (2.8-3.8)	6.2 (5.9 – 6.6)	<0.0001
**ANC clinic preparedness to offer FGM prevention and care services**						
Clinics with ALL correct answers for facility preparedness	0 (0%)	42 (52%)	<0.0001	42 (52%)	56 (69%)	<0.01
Mean score of clinic preparedness (out of 4)	0.1 (0.01-0.2)	3.1 (2.9-3.4)	<0.0001	3.1 (2.9-3.4)	3.4 (3.2-3.6)	0.18
Providers using level 1 intervention package	1 (1%)	61 (58%)	<0.0001	61 (58%)	96 (91%)	<0.0001
Providers offering appropriate FGM-related prevention and care services	11 (11%)	20 (19%)	<0.0001	20 (19%)	52 (50%)	<0.0001
**Secondary Outcomes**						
Providers with correct FGM-related knowledge responses	0 (0%)	1 (3%)	0.47	1 (3%)	8 (8%)	0.06
Providers with appropriate interpersonal communication skills	49 (49%)	62 (59%)	0.08	62 (59%)	74 (70%)	0.11
Providers with high self-efficacy	85 (85%)	94 (90%)	0.18	94 (90%)	86 (82%)	0.17
Providers reporting less supportive attitudes towards FGM	67 (67%)	75 (71%)	0.26	75 (71%)	76 (72%)	0.50
Providers with high confidence scores	84 (83%)	81 (77%)	0.30	81 (77%)	103 (98%)	<0.001
Providers not supportive of FGM	91 (91%)	101 (96%)	0.16	101 (96%)	100 (96%)	1.0
Providers not supportive of medicalized FGM	98 (97%)	104 (99%)	0.36	104 (99%)	104 (99%)	0.75
**Other ANC Client Outcomes**						
Clients reporting less support for FGM after ANC clinic visit	194 (24%)	235 (29%)	0.01	235 (29%)	424 (52%)	<0.0001
Clients reporting that they were strongly opposed to FGM	367 (45%)	345 (43%)	0.38	345 (43%)	498 (61%)	<0.0001
Clients reporting that they intend to have their daughters cut	249 (30%)	184 (23%)	<0.0001	184 (23%)	96 (12%)	<0.0001
Clients reporting that they would prefer health care provider to cut daughters	141 (17%)	117 (14%)	0.003	117 (14%)	53 (7%)	<0.001
Clients wishing to be active in FGM prevention	530 (65%)	547 (68%)	0.22	547 (68%)	677 (83%)	<0.001

For peer review only

CONSORT 2010 checklist of information to include when reporting a cluster randomised trial

Section/Topic	Item No	Standard Checklist item	Extension for cluster designs	Page No *
**Title and abstract**				
	1a	Identification as a randomised trial in the title	Identification as a cluster randomised trial in the title	1
	1b	Structured summary of trial design, methods, results, and conclusions (for specific guidance see CONSORT for abstracts)[1,2]	See table 2	3
**Introduction**				
**Background and objectives**	2a	Scientific background and explanation of rationale	Rationale for using a cluster design	5-6
	2b	Specific objectives or hypotheses	Whether objectives pertain to the cluster level, the individual participant level or both	7
**Methods**				
**Trial design**	3a	Description of trial design (such as parallel, factorial) including allocation ratio	Definition of cluster and description of how the design features apply to the clusters	7
	3b	Important changes to methods after trial commencement (such as eligibility criteria), with reasons		N/A
**Participants**	4a	Eligibility criteria for participants	Eligibility criteria for clusters	7-8
	4b	Settings and locations where the data were collected		6-7
**Interventions**	5	The interventions for each group with sufficient details to allow replication, including how and when they were actually administered	Whether interventions pertain to the cluster level, the individual participant level or both	6
**Outcomes**	6a	Completely defined pre-specified primary and	Whether outcome measures pertain to the cluster level, the	10

		secondary outcome measures, including how and when they were assessed	individual participant level or both	
	6b	Any changes to trial outcomes after the trial commenced, with reasons		N/A
**Sample size**	7a	How sample size was determined	Method of calculation, number of clusters(s) (and whether equal or unequal cluster sizes are assumed), cluster size, a coefficient of intra-cluster correlation (ICC or *k*), and an indication of its uncertainty	10-11
	7b	When applicable, explanation of any interim analyses and stopping guidelines		12
**Randomisation:**				
**Sequence generation**	8a	Method used to generate the random allocation sequence		8-9
	8b	Type of randomisation; details of any restriction (such as blocking and block size)	Details of stratification or matching if used	8-9
**Allocation concealment mechanism**	9	Mechanism used to implement the random allocation sequence (such as sequentially numbered containers), describing any steps taken to conceal the sequence until interventions were assigned	Specification that allocation was based on clusters rather than individuals and whether allocation concealment (if any) was at the cluster level, the individual participant level or both	9
**Implementation**	10	Who generated the random allocation sequence, who enrolled participants, and who assigned participants to interventions	Replace by 10a, 10b and 10c	
	10a		Who generated the random allocation sequence, who enrolled clusters, and who assigned clusters to interventions	8

For peer review only

	10b		Mechanism by which individual participants were included in clusters for the purposes of the trial (such as complete enumeration, random sampling)	8
	10c		From whom consent was sought (representatives of the cluster, or individual cluster members, or both), and whether consent was sought before or after randomisation	8
**Blinding**	11a	If done, who was blinded after assignment to interventions (for example, participants, care providers, those assessing outcomes) and how		8-9
	11b	If relevant, description of the similarity of interventions		8-9
**Statistical methods**	12a	Statistical methods used to compare groups for primary and secondary outcomes	How clustering was taken into account	10-13
	12b	Methods for additional analyses, such as subgroup analyses and adjusted analyses		12-13
**Results**				
**Participant flow (a diagram is strongly recommended)**	13a	For each group, the numbers of participants who were randomly assigned, received intended treatment, and were analysed for the primary outcome	For each group, the numbers of clusters that were randomly assigned, received intended treatment, and were analysed for the primary outcome	15
	13b	For each group, losses and exclusions after randomisation, together with reasons	For each group, losses and exclusions for both clusters and individual cluster members	Figure 2

For peer review only

**Recruitment**	14a	Dates defining the periods of recruitment and follow-up		15
	14b	Why the trial ended or was stopped		N/A
**Baseline data**	15	A table showing baseline demographic and clinical characteristics for each group	Baseline characteristics for the individual and cluster levels as applicable for each group	15-16
**Numbers analysed**	16	For each group, number of participants (denominator) included in each analysis and whether the analysis was by original assigned groups	For each group, number of clusters included in each analysis	15-16
**Outcomes and estimation**	17a	For each primary and secondary outcome, results for each group, and the estimated effect size and its precision (such as 95% confidence interval)	Results at the individual or cluster level as applicable and a coefficient of intra-cluster correlation (ICC or k) for each primary outcome	17-19
	17b	For binary outcomes, presentation of both absolute and relative effect sizes is recommended		19
**Ancillary analyses**	18	Results of any other analyses performed, including subgroup analyses and adjusted analyses, distinguishing pre-specified from exploratory		19
**Harms**	19	All important harms or unintended effects in each group (for specific guidance see CONSORT for harms[3])		N/A
**Discussion**				
**Limitations**	20	Trial limitations, addressing sources of potential bias, imprecision, and, if relevant, multiplicity of analyses		22-23
**Generalisability**	21	Generalisability (external validity, applicability) of the trial findings	Generalisability to clusters and/or individual participants (as relevant)	23

For peer review only

**Interpretation**	22	Interpretation consistent with results, balancing benefits and harms, and considering other relevant evidence	23-24
**Other information**			
**Registration**	23	Registration number and name of trial registry	15
**Protocol**	24	Where the full trial protocol can be accessed, if available	15
**Funding**	25	Sources of funding and other support (such as supply of drugs), role of funders	In Funding Statement

** Note: page numbers optional depending on journal requirements*

For peer review only
